# Low-intensity pulsed ultrasound-mediated nose-to-brain co-delivery of β-blockers and aPD-L1 enhances glioblastoma immunotherapy

**DOI:** 10.1038/s41467-026-76103-4

**Published:** 2026-07-30

**Authors:** Lei Dong, Zhengcheng Yun, Lin Gao, Yue Li, Ying Zhou, Yini Zhu, Meng Li, Leqian Ying, Xuhong Yang, Jiangtao Yue, Xueqing Yong, Wanqing Cheng, Jia Miao, Nuo Xu, Xinyu Zhang, Hui Yang, Tingting Liu, Gaolin Liang, Shenghong Ju, Haijun Zhang, Jinbing Xie

**Affiliations:** 1 Department of Oncology, Zhongda Hospital, Medical School of Southeast University, Nanjing, China; 2 Nurturing Center of Jiangsu Province for State Laboratory of AI Imaging & Interventional Radiology, Department of Radiology, Zhongda Hospital, Medical School of Southeast University, Nanjing, China; 3 State Key Laboratory of Mechanism and Quality of Chinese Medicine, Institute of Chinese Medical Sciences, University of Macau, Macau SAR, China; 4 Department of Microbiology and Immunology, Medical School of Southeast University, Nanjing, China; 5 Nursing Department, The Third People’s Hospital of Henan Province, School of Nursing, Zhengzhou University, Zhengzhou, China; 6 Nanjing Institute of Measurement and Testing Technology, Nanjing, China; 7 Department of Biochemistry and Molecular Biology, Medical School of Southeast University, Nanjing, China; 8 Zhongda Hospital, State Key Laboratory of Digital Medical Engineering, School of Public Health, Advanced Institute for Life and Health, Southeast University, Nanjing, China; 9 State Key Laboratory of Bioelectronics, School of Biological Science and Medical Engineering, Southeast University, Nanjing, China

**Keywords:** Nanoparticles, Permeation and transport, Neurophysiology, Targeted therapies

## Abstract

Intranasal delivery offers a direct route to the brain, circumventing the blood-brain barrier (BBB) and minimizing systemic toxicity. However, its efficiency is mainly limited by the nasal mucosal barrier (NMB). Here, low-intensity pulsed ultrasound (LIPUS) without depending on the microbubbles (MBs) to amplify energy, is directly used to reversibly open the NMB by disrupting tight junction proteins. A bionic nanovesicle (iRGD-anti-programmed cell death ligand 1 (aPD-L1) & carvedilol (β-blocker) @ macrophage-derived extracellular vesicles, iMPC) is designed to co-deliver carvedilol for β-receptor blockade to reduce T-cell exhaustion, and aPD-L1 to enhance T-cell anti-tumor activity in orthotopic glioblastoma (GBM) mice during the two-hour window for NMB opening. Consequently, compared to free aPD-L1, up to a 33.38-fold increase of aPD-L1 in the GBM region is obtained with LIPUS-mediated intranasal delivery of iMPC. Reactivating T cells significantly enhances immunotherapy, leading to a 40% tumor reduction, extended survival, and long-term immune memory in orthotopic GBM mice. Overall, the LIPUS-mediated NMB opening strategy notably enhances nose-to-brain drug delivery efficiency, offering a promising platform for treating brain diseases.

## Abstract (web summary)

Nasal delivery offers a delivery route to the brain that bypasses BBB, but is limited by the nasal mucosal barrier (NMB). Here, authors show that low-intensity pulsed ultrasound safely and reversibly opens the NMB, allowing nose-to-brain co-delivery of a β-blocker and aPD-L1 in a mouse model of glioblastoma.

## Introduction

Drug delivery faces significant challenges in treating various brain diseases, such as Alzheimer’s, Parkinson’s, and multiple sclerosis^1^. The nose-to-brain pathway provides a promising method to bypass the blood-brain barrier (BBB) and circumvent the hepatic and renal toxicity linked to systemic administration^2,3^. The nasal mucosal barrier (NMB) continues to restrict the efficiency of nose-to-brain drug delivery^1,2^. The NMB consists mainly of tight junction proteins, such as Claudin-1 and Occludin, in the nasal mucosal epithelium, along with the trigeminal bypass tight junction protein ZO-1^3,4^. Existing methods to address the NMB involve chemical permeation enhancers, which pose risks of mucosal damage and lack precise timing^3,5,6^.

Low-intensity pulsed ultrasound (LIPUS), a non-invasive physical energy technique, has shown the ability to temporarily open biological barriers^7^. Ultrasonic mechanical effects are generated by producing an electrical signal first, which is then amplified and fed to the ultrasonic horn^7–9^. Upon arriving at the horn, this electrical signal is transformed into mechanical waves by piezoelectric crystals via the transducer tip, and the waves are thereafter propagated to the target medium^8,9^. LIPUS can penetrate biological barriers due to its mechanical effects, including cavitation, viscous stress, and acoustic radiation^7–11^. For instance, drug permeation through the stratum corneum can be effectively enhanced under the acoustic energy of LIPUS^11^. Moreover, LIPUS has a promising therapeutic efficacy in clinical trials for brain diseases^12^. Microbubbles (MBs) amplify the acoustic energy of LIPUS to overcome 70-90% attenuation caused by the skull, enabling safe and reversible BBB opening^13^. Notably, NMB arises from the direct exposure of the nasal mucosa to the nasal cavity, devoid of physical skull barriers^14,15^. Currently, there are no studies documenting the direct use of LIPUS in NMB^7–9,15,16^. This study found that LIPUS can transiently loosen tight junctions of NMB via mechanical vibration, without requiring MBs-enhanced acoustic energy.

Immunotherapy, including anti-programmed cell death ligand 1 (aPD-L1), demonstrates significant potential in cancer treatment by inhibiting immune evasion and stimulating T-cell responses, thereby strengthening the immune system’s capacity to target and destroy tumors^17^. The limited effectiveness of immunotherapy in glioblastoma (GBM) is primarily attributed to the exhaustion of CD8^+^ T-cells within its immunosuppressive tumor microenvironment (TME), which makes them unresponsive to antigenic stimulation^17,18^. Most CD4^+^ T-cells in the TME of GBM act as regulatory T-cells (Tregs), contributing to immunosuppression^18–20^.

NE is reported to induce T-cell exhaustion by activating β-receptors on T-cells, thereby inhibiting their activity and weakening the immune system’s tumor-fighting capability^21^. It is noteworthy that adrenergic signaling simultaneously stimulates tumor proliferation and induces T-cell exhaustion, whereas cholinergic signaling maintains cancer stem cell properties^21–23^. These findings provide a theoretical basis for developing neural-targeting drugs such as β-adrenergic receptor blockers^21^. Thus, blocking the β-receptors of T cells to reduce T-cell exhaustion is expected to enhance GBM immunotherapy^17,21^.

Macrophage-derived extracellular vesicles (M-EVs) are ideal drug carriers owing to their natural biocompatibility, low immunogenicity, and ability to evade immune surveillance^24–26^. The membrane of M-EVs (EM) retains the CD47 protein, emitting a “don’t eat me” signal that inhibits recognition and clearance by the mononuclear phagocyte system^24,25^. In addition, study has shown that cleavage of iRGD, a cyclic nonpeptide targeting αvβ3 and αvβ5 integrins on tumor cells, in the TME exposed CendR motifs bound to neurohairpin-1 (NRP-1), increasing the tumor permeability of the nanoparticles and enhancing their accumulation at the tumor site^27,28^.

Polyaspartic acid (PASP) is a typical pH-sensitive polymer that primarily responds to environmental pH changes through the ionization properties of its side chains^29,30^. Under acidic conditions (pH 5.5–6.5), PASP’s negative charge rises due to the protonation of its side-chain carboxyl groups (-COOH/-COO⁻). This causes polymer chain swelling, inducing structural changes in the nanocarrier and releasing the loaded drug^31–33^. Conversely, at normal pH levels (such as pH 7.4, PASP maintains a relatively compact structure, inhibiting drug release^31^.

**Fig. 1:**
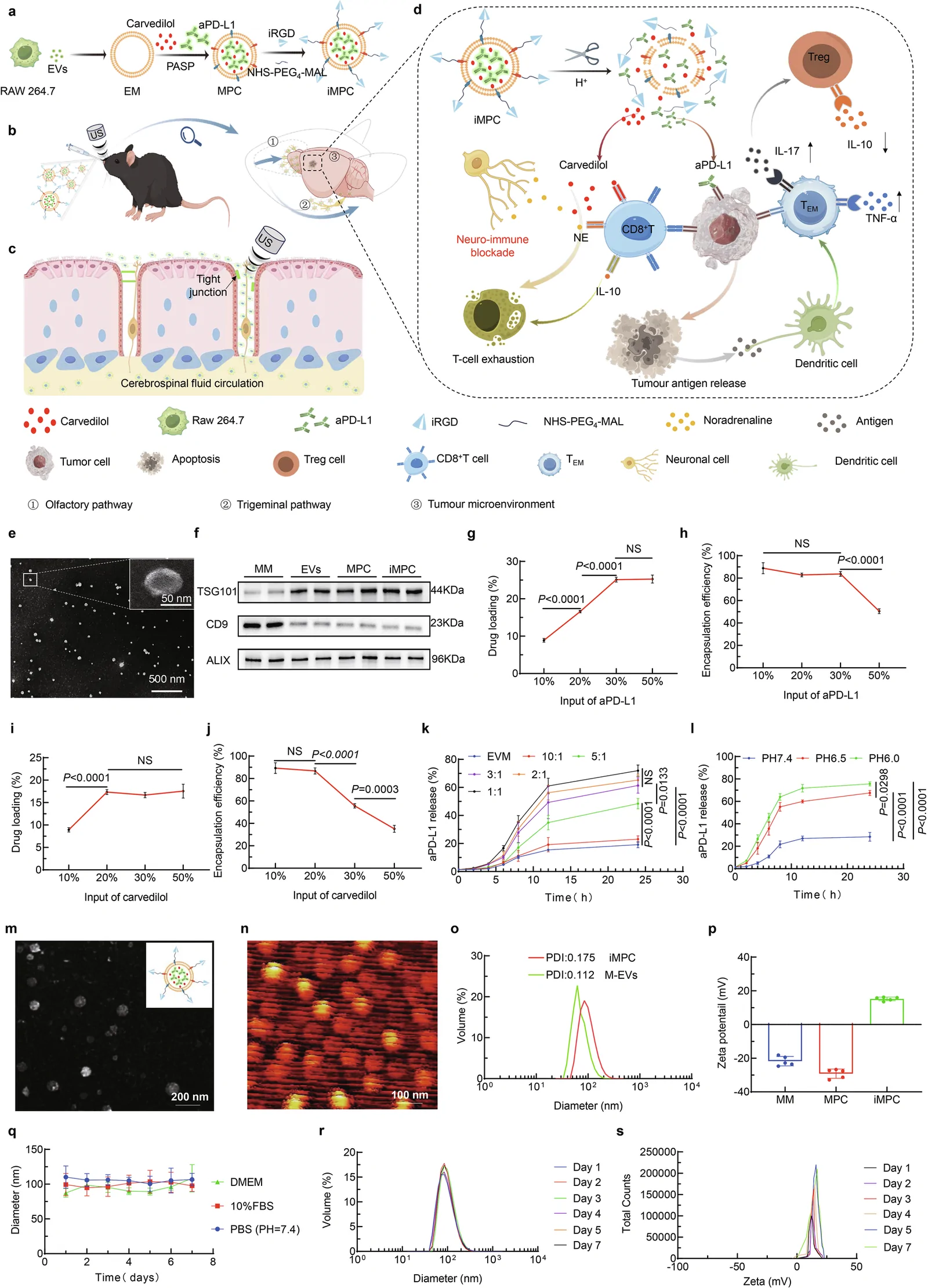
Construction and characterization of the bionic nanovesicle (iMPC). Panels a-d and m (insent schematic) were created by Figdraw. **a** Schematic diagram of iMPC synthesis (by figdraw.com). **b**, **c** Schematic representation of ultrasound-modulated nasal mucosal barrier (NMB) tight junctions promoting the transnasal delivery of iMPC to the brain (by figdraw.com). **d** Schematic diagram illustrating the principle of carvedilol blocking T-cell exhaustion combined with anti-programmed cell death ligand 1 (aPD-L1) to enhance immunotherapy for glioblastoma (GBM) (by figdraw.com). **e** Transmission electron microscopy image of extracellular vesicles from the macrophage cell line RAW 264.7. *n* = 3 samples. **f** Western blot (WB) detection of TSG101, CD9 and ALIX expression in macrophage membrane (MM), macrophage-derived extracellular vesicles (M-EVs), the bionic nanovesicle (aPD-L1&carvedilol@EM, MPC) and the bionic nanovesicle (iRGD-aPD-L1&carvedilol@EM, iMPC). *n* = 3 samples. **g** Drug loading of aPD-L1 in iMPC. **h** Encapsulation efficiency of aPD-L1 in iMPC. **i** Drug loading of carvedilol in iMPC. **j** Encapsulation efficiency of carvedilol in iMPC. **k** Release rate of aPD-L1 from biomimetic nanovesicles constructed with different mass ratios of RAW 264.7 extracellular vesicle membrane components and polyaspartic acid (PASP) in a solution with pH = 6.5 over different time periods. **l** Release efficiency of aPD-L1 in solutions with pH values of 7.4, 6.5, and 6.0. **m** Transmission electron microscopy image of iMPC. (The insent schematic was created by figdraw.com). *n* = 3 samples. **n** Atomic force microscopy image of iMPC. *n* = 3 samples. **o**, The average particle size of M-EVs is 51.34 ± 3.47 nm. The average particle size of iMPC is 102 ± 4.54 nm. **p** The average zeta potential of EVM, MPC and iMPC is −21.75 ± 2.81 mV, −29.18 ± 2.76 mV, and 15.21 ± 1.09 mV. **q** Stability testing of iMPC was conducted in 10% FBS, DMEM, and PBS (pH = 7.4 solutions over 7 days. **r**, **s** Changes in particle size and zeta potential of iMPC in a PBS (PH = 7.4 solution over 7 days. *n* = 4 samples per group in (**g–i**) and (**o–s**). Data are expressed as the mean ± SD. Statistical significances were determined using one-way ANOVA with Fisher’s LSD post‑hoc test. Comparisons were performed between different groups in (**g–i**). Not significant (NS) is *P* ≥ 0.05, and significant *P* values are shown. Source data are provided as a Source Data file.

Based on these facts, a strategy using LIPUS is designed to safely and reversibly create a 2-h NMB opening window. Notably, this approach employs LIPUS independently without MB-mediated energy amplification, thereby avoiding vascular damage and other side effects associated with conventional LIPUS/MBs combination therapy for BBB disruption^13,34^. Subsequently, a pH-responsive bionic nanovesicle (iRGD-aPD-L1&carvedilol@EM, iMPC) system based on EM is developed to co-deliver carvedilol (a β-adrenergic receptor inhibitor that mitigates T-cell exhaustion) and aPD-L1, working synergistically with LIPUS-mediated NMB opening (Fig. 1a-d). Reactivating T cells significantly improves immunotherapy, achieving a 40% tumor reduction, extended survival, and long-term immune memory in orthotopic GBM mice. LIPUS efficiently and temporarily opens the NMB, significantly improving nose-to-brain drug delivery and providing a promising method for treating brain diseases.

## Results

### Prognosis of GBM patients negatively correlates with NE-induced T cell exhaustion

Poor immunotherapeutic efficacy in GBM patients has been associated with a weaker immune response to exhausted T-cells in the TME^17,18,35^. Recent studies suggest NE plays a role in T-cell exhaustion^21^. Therefore, investigating the impact of NE on T cells in GBM patients could offer valuable insights for enhancing immunotherapy^17,22^. An analysis using the TCGA and GTEx databases via http://gepia2.cancer-pku.cn/ revealed significant overexpression of the *T-cell exhaustion gene set (HAVCR2, LAG3, PDCD1, CXCL13, LAYN, TIM3, CTLA4)* in GBM patients compared to healthy controls (Supplementary Fig. 1a, b).

A statistically significant association is observed between higher expression levels of these genes and poorer patient prognosis (Supplementary Fig. 1c). In addition, the *NE-related gene set (ADRA1B, ADRA1D, ADRA2A, ADRA2B, ADRA2C, DBH, PNMT, SLC6A2)* was overexpressed in GBM patients (Supplementary Fig. 1d). Similarly, a significant correlation exists between higher expression of this gene set and a worse prognosis (Supplementary Fig. 1e). A positive correlation exists between the expression of the *NE-related gene set* and the *T-cell exhaustion gene set* in GBM patients (Supplementary Fig. 1f). Conversely, the expression of the *Treg cell gene set (FOXP3-CTLA4-CCR8-TNFRSF9)* demonstrates a negative correlation with the prognosis of GBM patients, which is statistically significant (Supplementary Fig. 1g, h). Interestingly, the key *Tregs gene (FOXP3)* exhibits a positive correlation with the expression of the *NE-related gene set* (Supplementary Fig. 1g, h).

Furthermore, immunofluorescence triple staining was conducted on tumor tissues from GBM patients, both with and without NE inhibitor treatment (Supplementary Fig. 1i-k). The findings demonstrate a notable decrease in PD1 expression, a T-cell exhaustion marker, in tumor tissues of GBM patients treated with NE inhibitors compared with untreated patients (Supplementary Fig. 1i-k). These data suggest that in GBM patients, NE may promote tumor growth by inducing T-cell exhaustion^21^. Simultaneously, NE could exacerbate the formation of an immunosuppressive TME by activating Treg cells^19,20^. Blocking the effects of NE on T-cells has the potential to reprogram the TME to enhance the immunotherapy effect in GBM^22,35^.

### Elevated NE levels in the TME of the GBM stress model mice

Mice typically exhibit a marked NE increase in tumor tissue under stress^21,36^. By constructing stressed GBM mice model, we further validated the impact of NE on T-cell exhaustion^21,36^. The findings indicated that stressed GBM mice had notably elevated NE levels and increased tumor sizes in tumor tissue relative to untreated GBM mice (Supplementary Fig. 2a-c). Additionally, stressed GBM mice exhibited a shortened survival period and reduced body weight compared to untreated mice (Supplementary Fig. 2d, e). In the TME of stressed GBM mice, CD8^+^ T cell proportions decreased, whereas PD1 expression on these cells increased compared to untreated mice (Supplementary Fig. 2f-i). Triple immunofluorescence staining was conducted on tumor tissues from both stressed and native GBM mice (Supplementary Fig. 2j-l). The study found a significant increase in PD1 expression in the tumor tissues of stressed mice compared to native mice (Supplementary Fig. 2j-l). These data indicate that stressed GBM mice have increased NE content in tumor tissue, increased T-cell exhaustion, and larger tumor size.

### β-blockers reduce NE-induced T cell exhaustion

Recent research indicates that NE can cause T cell exhaustion^21^ through its interaction with β-adrenergic receptors (Supplementary Fig. 3a). In vivo experiments were performed to validate the impact of NE on T cells and assess the blocking efficacy of β-adrenergic receptor antagonists. The findings indicated that T cell activity was reduced in the NE-treated group compared to the untreated control group (Supplementary Fig. 3b). The NE plus carvedilol-treated group (NE + C) demonstrated significantly increased T cell activity compared to the NE-treated group (Supplementary Fig. 3b). When compared with the NE plus anti-PD-L1-treated group (NE + P), the group treated with NE, anti-PD-L1, and carvedilol (NE + P&C) displayed markedly increased T cell activity (Supplementary Fig. 3b). Additionally, T cell activity did not significantly differ between the NE+iMPC and NE + P&C treatment groups (Supplementary Fig. 3b). These findings indicate that NE can reduce T cell activity, and carvedilol effectively blocks this effect.

Analysis of the TCGA database revealed a positive correlation between *key genes of the cAMP/PKA/CREB signaling pathway (CREB1, CREBBP, ATF1, ATF2)* and *T cell exhaustion-related gene sets (HAVCR2, TIGIT, LAG3, PDCD1, CXCL13, LAYN)* in GBM patients (Supplementary Fig. 3c). Western blot (WB) analysis revealed that NE treatment significantly increased the expression levels of PKA and p-CREB in T cells compared to the control group (Supplementary Fig. 3d-g). In contrast, the NE combined with carvedilol (NE + C) treatment group exhibited a marked reduction in PKA and p-CREB expression in T cells relative to the NE-treated group (Supplementary Fig. 3h-k).

The impact of NE on the expression of T cell co-repressors LAG-3, Tim-3, and CTLA-4 was further examined using WB analysis^13,21,22^. The findings showed a significant increase in the expression levels of LAG-3, Tim-3, and CTLA-4 in the NE-treated group compared to the control group (Supplementary Fig. 3l-o). The group treated with both NE and carvedilol exhibited a marked reduction in the expression of co-inhibitory molecules compared to the group treated with NE alone (Supplementary Fig. 3p-s).

To further validate that NE induces T cell exhaustion by activating the cAMP/PKA/CREB signaling pathway, the specific pathway inhibitor KG501 was employed. Experimental results showed that T cells treated with NE + KG501 exhibited reduced expression levels of p-CREB, Tim-3, and CTLA-4 by 1.56-fold, 1.78-fold, and 4.86-fold, respectively, compared to the NE group (Supplementary Fig. 3t–w). These dates suggest that inhibition of the cAMP/PKA/CREB signaling pathway can attenuate NE-induced T cell exhaustion.

These findings suggest that NE activates the cAMP/PKA/CREB signaling pathway to induce T cell depletion, while β-adrenergic receptor blockade reduces NE-induced T cell exhaustion.

### iMPC has a high drug loading capacity, good stability, and pH responsiveness

The SFTE method^37^, which combines sonication, freeze-thaw cycles, and extrusion, was utilized to improve the loading capacity of iMPC for aPD-L1 and carvedilol. In our previous studies, efficient protein and aPD-L1 loading were achieved using this SFTE approach^37^. PASP-modified nanomaterials enhance acid responsiveness in acidic environments by undergoing protonation, which alters their molecular structure and increases solubility and degradation rates^29–31,38^. This property enables drug delivery systems to effectively target tumor or inflammation sites for localized therapy^29,32^.

By extracting the membrane components of M-EVs (EM) and fusing them with PASP, followed by surface modification with iRGD and co-loading of a β-blocker (carvedilol) and aPD-L1, iMPC was constructed (Fig. 1a). In combination with LIPUS-mediated NMB opening, iMPC enables efficient nose-to-brain delivery to enhance GBM immunotherapy (Fig. 1b−d). Characterization of iMPC was further evaluated. Transmission electron microscopy (TEM) shows that M-EVs exhibit a uniformly dispersed “cup-shaped” morphology with a particle size of about 50 nm (Fig. 1e). Particle size analysis revealed that M-EVs exhibited an average diameter of 51.34 ± 3.47 nm (Supplementary Fig. 4a).

To characterize the purity of M-EVs, we performed WB for key proteins of the macrophage membrane (MM) and EM. The results showed that M-EVs, MPC, and iMPC exhibited high expression of TSG101 and low expression of CD9 compared to the MM group, further confirming the purity of EVs (Fig. 1f). Calnexin, Histone H3, and cytochrome C levels were significantly lower in the M-EVs, MPC, and iMPC groups compared to the MM group (Supplementary Fig. 4b). These markers ruled out the possibility of contamination from other cellular components such as the endoplasmic reticulum, cell nucleus, and chromosomes. The nanovesicles derived from EM displayed superior chemotactic activity toward GBM cells (GL261) compared to those derived from MM. The targeting ability was further enhanced after iRGD modification of iMPC (Supplementary Fig. 4c, d).

Quantification using a multifunctional microplate reader revealed that at an aPD-L1 input ratio of 30%, the drug-loading capacity of iMPC was 25.12 ± 0.52%, with an encapsulation efficiency of 83.73 ± 1.74% (Fig. 1g, h). Further adjustment of the carvedilol input ratio showed that at a 20% carvedilol input ratio, the drug loading capacity of iMPC was 17.35 ± 0.44%, with an encapsulation efficiency of 86.75 ± 2.21% (Fig. 1i, j).

To enhance the acid responsiveness of iMPC, we further modified the proportion of PASP in EM^29–32^. The results demonstrated that in a pH 6.5 solution, when the input ratio of EM to PASP was 3:1, the responsive release of aPD-L1 increased by 3.21-fold compared to iMPC without PASP (Fig. 1k).

The efficiency of aPD-L1 release from iMPC was further compared at pH 7.4, 6.5, and 6.0. The results demonstrated that, compared to pH 7.4, the amount of aPD-L1 released from iMPC increased by 2.37-fold at pH 6.5 and by 2.66-fold at pH 6.0 (Fig. 1l). However, under acidic pH conditions, aPD-L1 was rapidly released (over 50%) within the 8 h (Fig. 1l). In the field of drug delivery, nanosystems are engineered for prolonged and sustained release^14,39^. Consequently, the iMPC drug-loaded nanosystem can be further designed and optimized to extend release duration, circumvent first-pass effects in the renal and hepatic systems, and thereby accommodate diverse administration routes^32,33^.

TEM and atomic force microscopy (AFM) revealed that iMPC were uniformly dispersed and spherical in shape (Fig. 1m, n). Dynamic light scattering analysis revealed that the average particle size was 102 ± 4.54 nm for iMPC and 51.34 ± 3.47 nm for M-EVs (Fig. 1o). The zeta potentials of EM, MPC, and iMPC were −21.75 ± 2.81 mV, −29.18 ± 2.76 mV, and 15.21 ± 1.09 mV, respectively (Fig. 1p). iMPC demonstrated remarkable stability over 7 days in DMEM, 10% fetal bovine serum (FBS), and PBS (pH 7.4, showing minimal alterations in particle size and zeta potential (Fig. 1q–s). These findings indicate that iMPC nanoparticles possess high drug-loading capacity, pH-responsive release behavior, and stability.

The particle size of iMPC nanoparticles increased significantly from 50 nm in M-EVs to 100 nm due to a hierarchical nanostructure formed by multi-step modification and loading^29,31,40^. Ultrasonic permeabilization converts M-EVs into hollow M-EVMs. The conjugated PASP molecular chains unfurl in aqueous environments, increasing steric hindrance and hydrodynamic diameter^29–32^. This transformation alters the vesicles from flexible native membranes to rigid polymer-membrane composites, raising the baseline particle size^31–33^. When aPD-L1 and carvedilol are ultrasonically loaded, they occupy the hollow lumen and bind to the inner membrane or PASP chains through hydrogen bonds and hydrophobic interactions, supporting the vesicle structure and further expanding particle size. Additionally, the iRGD peptide chains create a steric hydration layer in solution, significantly enhancing the nanoparticle’s hydrodynamic radius^27,28^.

### LIPUS-mediated NMB model opening

**Fig. 2:**
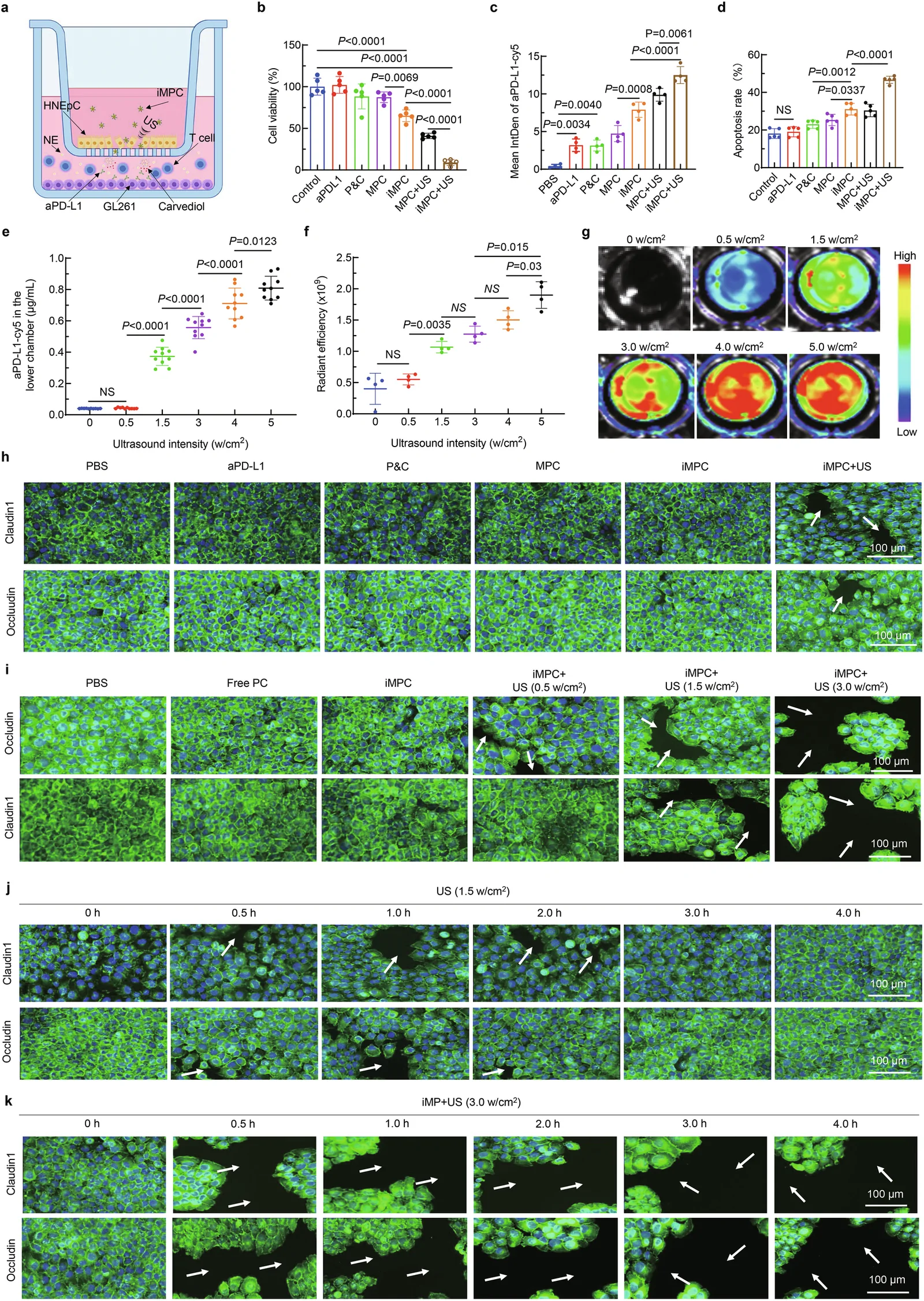
Low-intensity pulsed ultrasound (LIPUS)-triggered nasal mucosal barrier (NMB) model opening combined with the bionic nanovesicle (iMPC) inhibits GL261 cell growth in vitro. **a** was created by Figdraw. **a** Schematic diagram of an in vitro simulation experiment where LIPUS enhances the penetration of iMPC through the NMB, increasing T-cell suppression of GL261 cells (by figdraw.com). **b** Assessment of GL261 cell viability following LIPUS-mediated NMB model opening combined with iMPC treatment. **c** The fluorescence intensity of Cy5 NHS-labeled anti-programmed cell death ligand 1 (aPD-L1) in the lower chamber. **d** The early apoptosis rate, late apoptosis rate, and total apoptosis rate of GL261 cells after 24 h of different treatments. **e** Quantitative analysis of aPD-L1 in the lower chamber of the migration Chamber using a multifunctional microplate reader after LIPUS treatment at different intensities. **f**,** g** In vivo imaging system evaluation of the amount of iMPC penetration through the NMB model after 60 s of LIPUS exposure at intensities of 0, 0.5, 1.5, 3.0, 4.0, and 5.0 w/cm². **h** Immunofluorescence of Claudin-1 expression (blue: DAPI; green: Alexa Fluor 488-Claudin-1) after 1 h of different treatments. Immunofluorescence of Occludin expression (blue: DAPI; green: Alexa Fluor 488-Occludin) after 1 h of different treatments. *n* = 3 samples. **i** Comparison of the effects of ultrasound on the tight junction proteins Occludin and Claudin-1 at intensities of 0.5, 1.5, and 3.0 w/cm². *n* = 3 samples. **j** Immunofluorescence staining of Occludin and Claudin-1 was performed at 0, 0.5, 1, 2, 3, and 4 h after LIPUS exposure at 1.5 w/cm² for 60 s (blue: DAPI; green: Alexa fluor 488-Occludin or Claudin-1). *n* = 3 samples. **k** Immunofluorescence staining of Occludin and Claudin-1 was conducted at 0, 0.5, 1, 2, 3, and 4 h after LIPUS exposure at 3.0 w/cm² for 60 s (blue: DAPI; green: Alexa fluor 488-Occludin or Claudin-1. *n* = 3 samples. The area marked with a white arrow in (**h–k**) indicates the open section of the NMB model. *n* = 5 samples per group for data in (**b**). *n* = 4 samples per group for data in (**c**, **d** and f). *n* = 10 samples per group for data in **e**. Data are expressed as the mean ± SD. Comparisons were performed between different groups in (**a–f**). Statistical significances were determined using one-way ANOVA with Fisher’s LSD post‑hoc test. Not significant (NS) is *P* ≥ 0.05, and significant *P* values are shown. Source data are provided as a Source Data file.

In vitro experiments were conducted to mimic the LIPUS open NMB model and improve iMPC permeation efficiency. A transwell-based NMB model was developed and analyzed using Dunnett’s post hoc test (Fig. 2a). Compared to the untreated control group, the cell viability of GL261 cells treated with free iMPC decreased by 1.54-fold (Fig. 2b). In comparison with the free iMPC-treated group, the cell viability of GL261 cells in the US+iMPC-treated group was reduced by 7.24-fold (Fig. 2b). Fluorescence microscopy demonstrated a notable increase in aPD-L1 content in the lower chamber for the US+iMPC-treated group compared to the iMPC-treated group (Fig. 2c and Supplementary Fig. 4e).

Analysis of extracellular solutions from GL261 cells revealed enhanced fluorescence signals in the iMPC+GL261 group compared to the iMPC group, consistent with free aPD-L1-Cy5 fluorescence signals (Supplementary Fig. 4f). Confocal microscopy revealed that iMPC-released aPD-L1-Cy5 accumulated in the intercellular spaces of GL261-GFP cells (Supplementary Fig. 4g). These results indicate that iMPC release of aPD-L1 occurs in the intercellular spaces of tumor cells rather than inside tumor cells.

The impact of US+iMPC on GL261 cells was further assessed using scratch tests, flow-through apoptosis assays, and immunofluorescence assays. Scratch test results showed that, compared with free iMPC treatment, US+iMPC treatment resulted in a 4.05-fold decrease in GL261 cell migration rate (Supplementary Fig. 5a-c). GL261 cell migration remained largely unchanged in both the free aPD-L1 and P&C (free aPD-L1 & carvedilol) groups relative to the control group, primarily due to NMB hindering drug permeation (Supplementary Fig. 5a-c).

Additional research indicated that GL261 cell viability remained unchanged in the LIPUS-only group relative to the PBS group (Supplementary Fig. 5d). Compared to the Free P&C group, GL261 cell viability decreased by 59.93% in the iMPC group and by 85.92% in the iMPC+US group (Supplementary Fig. 5d). Compared with the LIPUS group, GL261 cell viability decreased by 87.48% in the iMPC+US group (Supplementary Fig. 5d). The data suggest that LIPUS at an intensity of 1.5 w/cm² does not impact the viability of GL261 cells.

Caspase-3 immunofluorescence and flow cytometry analyses revealed a 2. 29-fold increase in the apoptosis rate of GL261 cells following US+iMPC treatment compared to free iMPC treatment (Supplementary Fig. 5d, e). Likewise, caspase-3 expression and apoptosis rate in GL261 cells remained unchanged in both the free aPD-L1 and P&C groups compared to the control group (Supplementary Fig. 5d-f).

Flow cytometry analysis indicated that the early apoptosis rate of GL261 cells increased by 112.95% in the free PC group and by 192.17% in the iMPC+US group compared to the control group (Fig. 2d and Supplementary Fig. 5g-i). The late apoptosis rate of GL261 cells increased by 131.87% in the free PC group and by 279.32% in the iMPC+US group compared to the control group (Fig. 2d and Supplementary Fig. 5g-i). The total apoptosis rate of GL261 cells increased by 134.81% in the iMPC group and by 202.46% in the iMPC+US group compared to the free PC group (Fig. 2d and Supplementary Fig. 5g-i).

Flow cytometry was used to assess the impact of combining LIPUS with iMPC on preventing T cell exhaustion (Supplementary Fig. 6). The US+iMPC treatment group showed a higher T cell proportion in the lower chamber than other groups (Supplementary Fig. 7a, b). The CD3^+^CD8^+^ T cell proportion in the lower chamber of the US+iMPC group was 119.5% higher than in the iMPC-treated group (Supplementary Fig. 7a, b). The US+iMPC group exhibited a significant increase in the proportions of IFN-γ^+^CD8^+^ T cells and TNF-α^+^CD8^+^ T cells in the lower chamber compared to other treatment groups (Supplementary Fig. 7c, d).

Furthermore, in the US+iMPC group, the proportion of IFN-γ^+^CD8^+^ T cells rose by 178.09%, and TNF-α^+^CD8^+^ T cells increased by 194.74% in the lower chamber compared to the iMPC-treated group (Supplementary Fig. 7c, d). In the US+iMPC group, T cells exhibited notably lower expression levels of Tim-3 and PD-1 in the lower chamber compared to the iMPC treatment group alone (Supplementary Fig. 7e, f). The US+iMPC group exhibited an 84.2% reduction in PD-1^+^Tim-3^+^CD8^+^ T cells in the lower chamber compared to the iMPC-treated group (Supplementary Fig. 7g, h).

The transendothelial electrical resistance (TEER) values in the NMB model stabilized by the fifth day, as shown in Supplementary Fig. 8a. An in vivo imaging system (IVIS) assessment demonstrated that the aPD-L1 content in the lower chamber progressively rose with increased Ultrasound intensity (Fig. 2e−g). This conclusion was further validated by quantitative measurements using a microplate reader (Fig. 2e-g). TEER measurement results indicate that the TEER value of the NMB model gradually decreases with increasing Ultrasound intensity (Supplementary Fig. 8b). The findings suggest a significant correlation between the extent of NMB disruption and ultrasonic energy, highlighting the need to identify a safe ultrasound intensity range to ensure NMB damage reversibility.

We further investigate changes in tight junctions in the NMB model via immunofluorescence. The study found that US+iMPC treatment in the NMB model led to the disruption of tight junction proteins Claudin-1 and Occludin (Fig. 2h). Compared to other groups without US treatment, the TEER values for the NMB model in the US+iMPC group were significantly reduced (Supplementary Fig. 8c). The Free aPD-L1, P&C, MPC, and iMPC-treated groups showed no significant changes in the tight junction proteins Claudin-1 and Occludin, indicating that LIPUS, when not applied, does not significantly affect NMB (Fig. 2h). When other parameters (time 60 s, duty cycle 50%, frequency 1 MHz) remained constant, the disruption of Claudin-1 and Occludin became more pronounced with increasing Ultrasound intensity (Fig. 2i). This suggests that the degree of NMB disruption gradually increases as the ultrasonic energy is enhanced (Fig. 2i).

To further assess the reversibility of NMB disruption by LIPUS, immunofluorescence experiments and TEER test were performed. The results demonstrated that when the LIPUltrasound intensity was 1.5 w/cm², the NMB model could recover within 4 h (Fig. 2j and Supplementary Fig. 8d). In contrast, at a higher LIPUltrasound intensity of 3.0 w/cm², the disruption of tight junction proteins in the NMB model was irreversible (Fig. 2k and Supplementary Fig. 8e). These dates demonstrates that LIPUS at 1.5 w/cm² intensity can safely and reversibly disrupt NMB tight junction proteins, allowing iMPC to penetrate the NMB, reduce T-cell exhaustion, and improve the inhibition of GL261 cells.

Furthermore, compared with the group without iMPC treatment (−), the cell viability of HNEpC, bEnd.3, SH-SY5Y, PC12, and Neuro-2a cells in the iMPC-treated group (+) showed no significant changes (Supplementary Fig. 8f). This suggests that iMPC, as an anti-tumor immune-enhancing nanomedication, exhibits favorable biosafety.

Research has shown a strong association between the cGMP/PKG signaling pathway and tight junction protein levels^41^. WB analysis indicated a significant elevation in PKG protein levels in the US-treated group compared to the untreated group, with this increase showing a positive correlation with ultrasound intensity (Supplementary Fig. 9a, b). PKG protein levels in the iMPC+US (1.5 w/cm²) group were 3.59 times higher than those in the control group (Supplementary Fig. 9a, b). In the US-treated group, RhoA and Rock protein levels were significantly lower compared to the non-US-treated group, showing a negative correlation with Ultrasound intensity (Supplementary Fig. 9c, d).

In the iMPC+US (1.5 w/cm²) group, RhoA and Rock protein levels were reduced by 1.52-fold and 4.72-fold, respectively, compared to the control group (Supplementary Fig. 9c, d). The US-treated group showed a significant decrease in tight junction proteins (ZO-1, Claudin-1, and Occludin) compared to the non-US-treated group, with these reductions negatively correlating with Ultrasound intensity (Supplementary Fig. 9e-g). The iMPC+US (1.5 w/cm²) group showed a reduction in protein levels of ZO-1, Claudin-1, and Occludin by 2.43-fold, 3.71-fold, and 2.35-fold, respectively, compared to the control group (Supplementary Fig. 9e-g). These findings indicate that the US could interfere with NMB by triggering the cGMP/PKG signaling pathway.

### β-adrenergic receptor blockade combined with aPD-L1 enhances T-cell antitumor activity

CD4^+^ T cells can secrete IFN-γ and TNF-α in tumor environments^42,43^. IFN-γ and TNF-α enhance antigen-presenting cell activation and promote the proliferation and activation of cytotoxic CD8^+^ T cells^42,43^. We subsequently assessed the impact of combining β-blocker with aPD-L1 on the activity of CD4^+^ and CD8^+^ T cells using flow cytometry (Supplementary Fig. 10).

Results indicated a 3.97-fold increase in the proportion of CD45^+^ lymphocytes in the lower chamber of the transwell in the NE + PC group compared to the NE group (Supplementary Fig. 11a and e). The iMPC+US group showed no significant change in the proportion of CD45^+^ lymphocytes in the transwell’s lower chamber compared to the NE + PC group (Supplementary Fig. 11a, e). The iMPC+US group exhibited a 1.79-fold increase in the proportion of CD45^+^ lymphocytes in the lower chamber of the transwell compared to the iMPC group (Supplementary Fig. 11a, e).

Detection of CD3^+^CD8^+^ T cell proportions in the lower chamber section revealed a 3.08-fold increase in the NE + PC group compared to the NE group (Supplementary Fig. 11b, f), a 4.33-fold rise in IFNγ^+^CD8^+^ T cell proportion (Supplementary Fig. 11c, g) demonstrate a 4.36-fold increase in the proportion of TNF-α^+^CD8^+^ T cells (Supplementary Fig. 11d, h). The iMPC+US group did not exhibit significant changes in the proportions of CD3^+^CD8^+^ T cells, IFNγ^+^CD8^+^ T cells, and TNF-α^+^CD8^+^ T cells in the lower chamber compared to the NE + PC group (Supplementary Fig. 11d, f, g, h). The iMPC+US group exhibited a 1.73-fold higher proportion of CD3^+^CD8^+^ T cells compared to the iMPC group (Supplementary Fig. 11b, f), a 1.71-fold rise in the percentage of IFNγ^+^CD8^+^ T cells (refer to Supplementary Fig. 11c, g), and 1.72-fold rise in the percentage of TNF-α^+^CD8^+^ T cells (Supplementary Fig. 11d, h).

Analysis of CD3^+^CD4^+^ T cell ratios in the lower chamber showed a 4.01-fold increase in the NE + PC group (Supplementary Fig. 12a, d), a 5.15-fold increase in IFNγ^+^CD4^+^ T cell ratio (Supplementary Fig. 12b, e), with a 4.06-fold increase in the proportion of TNF-α^+^ CD4^+^ T cells (Supplementary Fig. 12c, f), compared to the NE group. The iMPC+US group did not exhibit significant changes in the proportions of CD3^+^CD4^+^, IFN-γ^+^CD4^+^, and TNF-α^+^CD4^+^ T cells in the lower chamber compared to the NE + PC group (Supplementary Fig. 12a-f). The iMPC+US group showed a 1.89-fold higher proportion of CD3^+^CD4^+^ T cells compared to the iMPC group (Supplementary Fig. 12a, d), a 4.71-fold increase in the proportion of IFN-γ^+^CD4^+^ T cells (Supplementary Fig. 12a, d), and a 3.28-fold higher proportion of TNF-α^+^ CD4^+^ T cells (Supplementary Fig. 12b, e, c, f).

These results indicate that combined β-blocker and aPD-L1 therapy enhances T cell antitumor activity. The proportion of CD4^+^ and CD8^+^ T cells with elevated TNF-α and IFN-γ secretion significantly increased. LIPUS-mediated NMB opening facilitates iMPC migration to the lower chamber, and releases carvedilol and aPD-L1 to boost T cell antitumor activity. This effect closely resembles the direct administration of β-blockers and aPD-L1 to the lower chamber.

### LIPUS improves iMPC nose-to-brain delivery efficiency in vivo

**Fig. 3:**
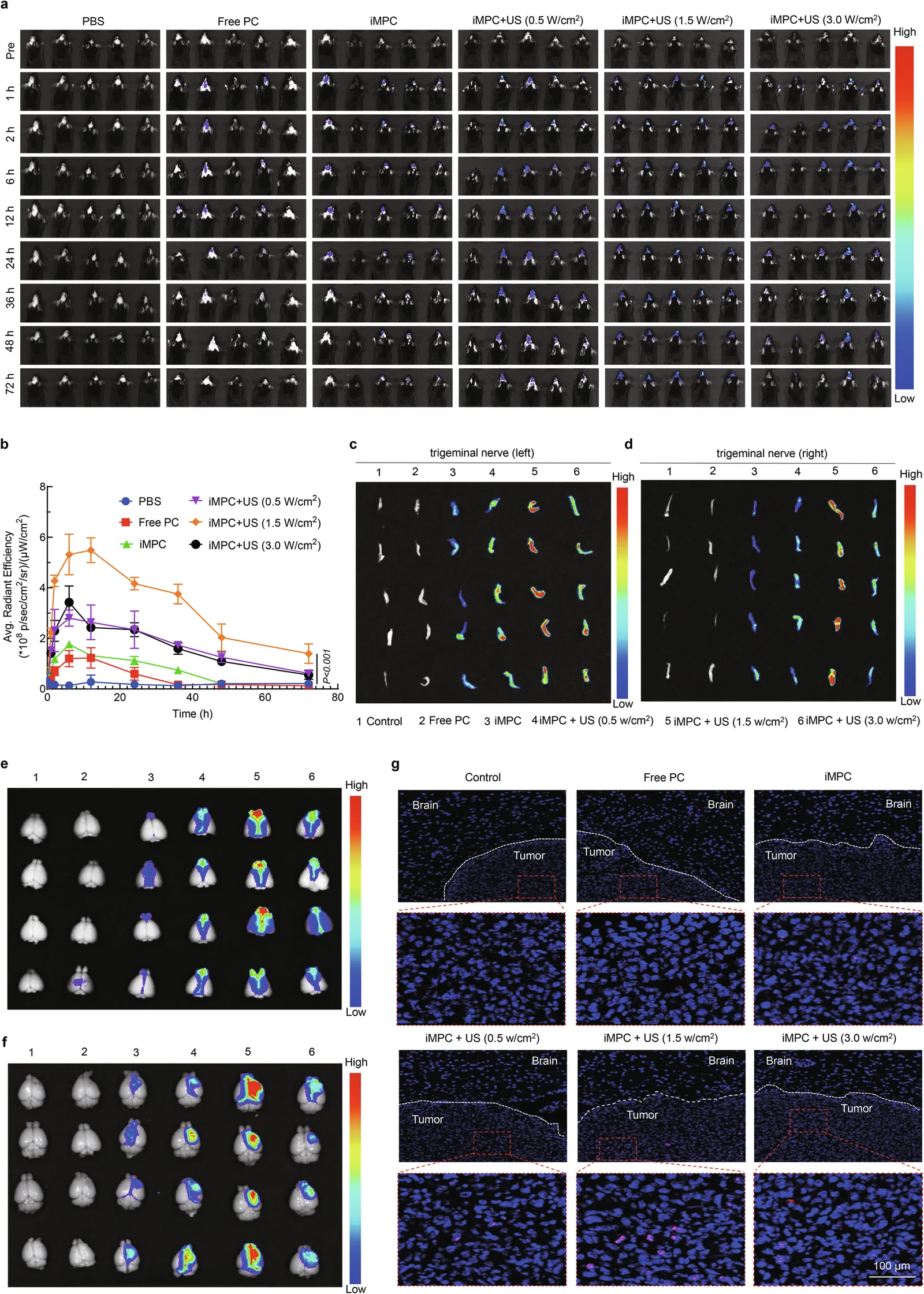
Low-intensity pulsed ultrasound (LIPUS) improves the bionic nanovesicle (iMPC) nose-to-brain delivery efficiency in vivo. **a**, **b** The distribution of Cy5 NHS-labeled anti-programmed cell death ligand 1 (aPD-L1) after various treatments. ImageJ software analyzes fluorescence signals. **c**, **d** The distribution of aPD-L1-Cy5 in the trigeminal nerve after 1 h of treatment. **e** The distribution of aPD-L1-Cy5 in the olfactory bulb after 2 h of treatment. **f**, **g** The distribution of aPD-L1-Cy5 in the brain tumor area after 6 h of treatment using both the small animal in vivo imaging system and frozen sectioning. *n* = 3 samples per group for data in (**g**). *n* = 4 samples per group for data in (**b**). Data are expressed as the mean ± SD. Source data are provided as a Source Data file.

Additional in vivo experiments assessed the efficiency of LIPUS-enhanced iMPC penetration across the NMB. IVIS imaging analyses demonstrated that the combination of LIPUS and iMPCs significantly improved iMPC penetration across the NMB and extended their retention time in the GBM microenvironment compared with iMPC treatment alone (Fig. 3a, b). In addition, iMPC had the highest nose-to-brain delivery efficiency at an ultrasound intensity was 1.5 w/cm², compared to 0.5 or 3.0 w/cm² (Fig. 3a, b).

Research indicates that the trigeminal bypass is the primary route for nose-to-brain drug delivery, accessing the olfactory bulb and brain ventricles^2,3,6,14^. IVIS observations at 1 h post-treatment indicated that the LIPUS combined with iMPC treatment group showed enhanced iMPC distribution on the trigeminal nerve surface compared to the iMPC treatment group (Fig. 3c, d and Supplementary Fig. 13a, b).

The LIPUS treatment at an intensity of 1.5 w/cm² resulted in the greatest iMPC accumulation on the isolated trigeminal nerve surface compared to intensities of 0.5 or 3.0 w/cm² (Fig. 3c, d and Supplementary Fig. 13a, b). The fluorescent signals (aPD-L1-Cy5) distributed on the surface of the right and left branches of the trigeminal nerve increased by 9.74 and 15.04-fold, respectively, in the US+iMPC group (1.5 w/cm²) compared with the free PC (aPD-L1 and carvedilol) group (Supplementary Fig. 13a, b).

Moreover, at 2 h post-treatment, the LIPUS exposure significant enhanced iMPC distribution on the surface of the isolated olfactory bulb compared to iMPC alone (Fig. 3e and Supplementary Fig. 13c). Notably, the LIPUS treatment at 1.5 w/cm² resulted in the highest iMPC content on the olfactory bulb surface (Fig. 3e and Supplementary Fig. 13c). The fluorescence signal (aPD-L1-Cy5) on the surface of the olfactory bulb increased 12.11-fold in the US+iMPC group (1.5 w/cm²) compared to the free PC-treated group (Supplementary Fig. 13c). By 6 h post-treatment, increased iMPC aggregation in the GBM region was observed in US+iMPC group compared with the iMPC treatment group (Fig. 3f and Supplementary Fig. 13d). Moreover, the LIPUS treatment at 1.5 w/cm² demonstrated the highest iMPC content in the GBM region (Fig. 3f and Supplementary Fig. 13d). The fluorescence signal (aPD-L1-Cy5) on the surface of the olfactory bulb increased 12.53-fold in the US+iMPC group (1.5 w/cm²) compared to the free PC-treated group (Supplementary Fig. 13d).

Frozen section analysis of brain tissue indicated that the LIPUS combined with iMPC treatment group showed increased iMPC infiltration in tumor tissue compared to the iMPC treatment group (Fig. 3 g). The LIPUS treatment at 1.5 w/cm² led to the greatest iMPC infiltration in tumor tissue (Fig. 3g). These results indicate that LIPUS can significantly improve the efficiency of iMPC penetration across the NMB into the brain, and iMPC possesses the ability to target and accumulate in GBM.

The study further assessed how different acoustic parameters, such as ultrasound intensity, center frequency, duty cycle, and duration, influence the efficiency of LIPUS-mediated nose-to-brain delivery of iMPC. The findings indicated that the iMPC-Cy5 concentration in tumor tissues of GBM mice subjected to LIPUS at 1.5 w/cm² was 3.55 times greater than in those treated with 0 w/cm² (Supplementary Fig. 13e). Modulating various LIPUS intensities revealed that 1.5 w/cm² maximized iMPC nose-to-brain delivery efficiency (Supplementary Fig. 13f). The iMPC-Cy5 level in tumor tissues of GBM mice subjected to LIPUS with a 30% duty cycle was 1.95 times greater than in those treated with a 10% duty cycle (Supplementary Fig. 13g). Treatment with LIPUS at a center frequency of 1.0 MHz led to a 1.97-fold increase in iMPC-Cy5 in tumor tissues compared to a frequency of 0.5 MHz (Supplementary Fig. 13h).

Further modulation of the LIPUS exposure duration revealed that the highest iMPC-Cy5 content in tumor tissues was achieved with an LIPUS duration of 60 s (Supplementary Fig. 13i). A decline in nose-to-brain delivery efficiency of iMPC was observed when the LIPUS duration was extended to 120 and 180 s, which may be attributed to thermal damage of the nasal mucosa induced by prolonged LIPUS exposure^7,11^. The study identifies the optimal acoustic parameters for LIPUS-mediated iMPC nose-to-brain delivery as a center frequency of 1.0 MHz, ultrasound intensity of 1.5 w/cm², a duty cycle of 30%, and a duration of 60 s.

### LIPUS opens NMB by disrupting the tight junction proteins

**Fig. 4:**
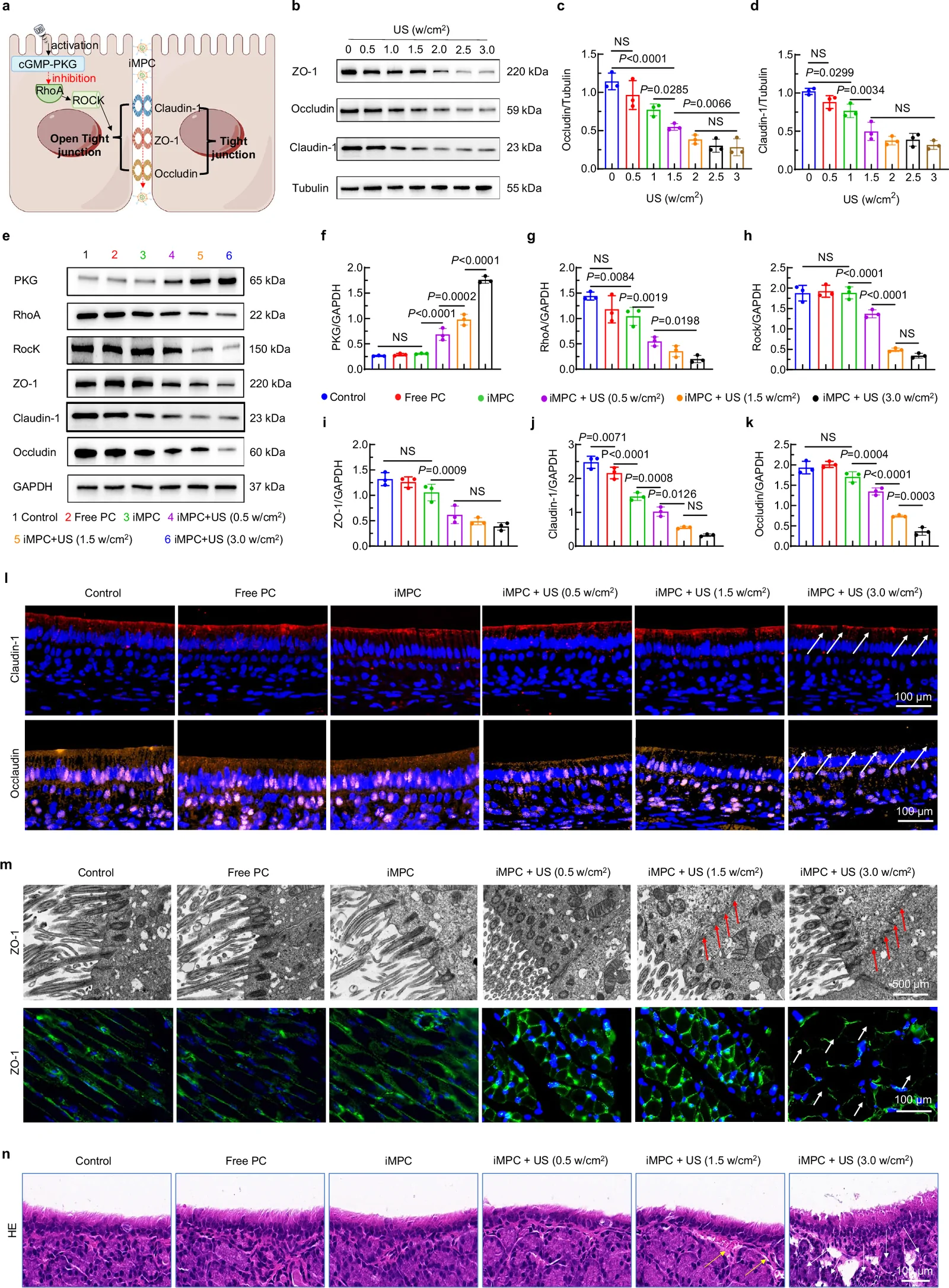
Low-intensity pulsed ultrasound (LIPUS) opens the nasal mucosal barrier (NMB) by disrupting the tight junction proteins. **a** was created by Figdraw. **a**, Schematic diagram of LIPUS activating the cGMP/PKG signaling pathway to regulate tight junction proteins in the NMB (by figdraw.com). **b–d** The effects of different ultrasonic intensities on tight junction proteins ZO-1, Occludin, and Claudin-1. **e–k** The effects of different treatments on the cGMP/PKG signaling pathway and tight junction proteins. **l** Changes in tight junction proteins Occludin and Claudin-1 (blue: DAPI; red: CY3-Claudin-1; yellow: SpRed-Occludin), *n* = 3 samples. **m** Alterations in tight junction proteins of the paracellular pathway (blue: DAPI; green: Alexa Fluor 488-ZO-1), *n* = 3 samples. **n** Inflammatory cell infiltration after various treatments, *n* = 3 samples. *n* = 3 samples per group for data in (**c**, **d** and **f–k**). Data are expressed as the mea*n* ± SD. Statistical significances were determined using one-way ANOVA with Fisher’s LSD post‑hoc test. Comparisons were performed between different groups in (**c**, **d** and **f–k**). Not significant (NS) is *P* ≥ 0.05, and significant *P* values are shown. Source data are provided as a Source Data file.

In addition to the physical effects of LIPUS on NMB results, studies have shown that the cGMP/PKG/RhoA/RocK signaling pathway^7,10,41^ is closely related to tight junction proteins ZO-1, Claudin-1, and Occludin (Fig. 4a). Research indicates that the Piezo1 ion channel responds to mechanical stimulation by converting LIPUS signals into intracellular calcium ion currents^7,10,44^. Enhanced calcium signaling stimulates the small GTPase RhoA and its downstream effector, Rho-associated protein kinase (ROCK)^43,45^. ROCK is essential for cellular functions, including contraction, morphological alterations, and migration. Ca²⁺ activates specific calmodulins, thereby activating the small GTPase RhoA, which leads to ROCK activation^45,46^. Ca²⁺ also enhances actin contractility by activating myosin light chain kinase^46,47^.

To further elucidate the mechanisms by which LIPUS opens the NMB, we assayed key proteins in this signaling pathway. WB analysis revealed that increasing LIPUltrasound intensity significantly augmented the degradation of ZO-1, Claudin-1, and Occludin in the nasal mucosal epithelium (Fig. 4b–d and Supplementary Fig. 13j). The destruction of NMB tight junction proteins by LIPUS was most significant when the ultrasound intensity was 2.0, 2.5 or 3.0 w/cm² (Fig. 4b–d and Supplementary Fig. 13j). LIPUS under these conditions disrupts NMB more strongly and may cause some safety concerns.

Following intranasal administration, drugs not only enter the brain via the “nose-brain pathway,” but a portion often enters the lungs or enters the systemic blood circulation through the capillaries of the nasal mucosa^3,4^. Compared with free carvedilol, the amount of carvedilol entering the bloodstream in the iMPC group did not decrease significantly (Supplementary Fig. 14a).

In contrast, compared with the free carvedilol or iMPC group, the amount of carvedilol entering the bloodstream was significantly reduced in the iMPC+US group (Supplementary Fig. 14a). The in vivo half-life of carvedilol was 6 h (Supplementary Fig. 14a). Compared with the free aPD-L1 or iMPC groups, the amount of aPD-L1 entering the bloodstream was significantly reduced in the iMPC+US group (Supplementary Fig. 14b). The in vivo half-life of aPD-L1 was 36 h (Supplementary Fig. 14b). These data indicate that carvedilol and aPD-L1 administered intranasally enter the circulation, with aPD-L1 persisting longer than carvedilol.

The distribution of carvedilol and aPD-L1 across the heart, liver, spleen, lungs, kidneys, and brain in various treatment groups showed that the iMPC+US group had significantly lower aPD-L1 levels in the liver and lungs compared to free aPD-L1 or iMPC alone, while the distribution in brain tumors was notably higher (Supplementary Fig. 15a, b). Compared with free carvedilol or iMPC, the iMPC+US group showed significantly reduced carvedilol distribution in the liver and lungs, while brain tumor distribution was significantly increased (Supplementary Fig. 15c, d).

Clearance rate measurements indicated that carvedilol was rapidly cleared from liver, kidney, and lung tissues within 6 h, while aPD-L1 exhibited a significantly delayed clearance, extending beyond 48 h (Supplementary Fig. 16a-l). These data indicate that US enhances iMPC brain penetration efficiency by opening the NMB, thereby delivering carvedilol and aPD-L1 to tumor regions. Compared to free carvedilol or the nanoparticle formulation, the clearance rate of free aPD-L1 or the nanoparticle formulation is slower.

Sustained LIPUS exposure often induces varying degrees of “thermal effects.”^8,9,11^ Thermal imaging results indicate that the temperature of the NMB model increases with rising Ultrasound intensity (Supplementary Fig. 17a, b). The NMB model attained a temperature of 29.3 °C after 60 s of continuous ultrasound exposure at an intensity of 1.5 w/cm² (Supplementary Fig. 17a, b). At an ultrasound intensity of 3.0 w/cm² with 60 s of continuous exposure, the NMB model reached a temperature of 50.1 °C (Supplementary Fig. 17a, b).

In vivo experiments demonstrated that the temperature in the mouse nasal mucosa region gradually increased with rising Ultrasound intensity (Supplementary Fig. 17c, d). Specifically, when Ultrasound intensity reached 3.0 w/cm² and was applied continuously for 60 s, the temperature in the mouse nasal mucosa region reached 50.6 °C (Supplementary Fig. 17c, d). At an Ultrasound intensity of 4.0 w/cm² with a 60-s exposure duration, the temperature in the mouse nasal mucosa region reached 54.7 °C (Supplementary Fig. 17c, d). These data indicate that sustained exposure to US at certain intensities may cause thermal injury to nasal mucosal tissue.

WB analysis indicated that CD68 levels in the nasal mucosal tissue of mice exposed to 1.5 w/cm² US were comparable to the control group (0 w/cm²), whereas exposure to 3.0 w/cm² US resulted in a 4.41-fold increase in CD68 levels (Supplementary Fig. 17e, f). HSP70 levels in the nasal mucosa of mice exposed to 1.5 w/cm² ultrasound showed no significant change compared to the control group (0 w/cm²), whereas exposure to 3.0 w/cm² resulted in a 2.83-fold increase (Supplementary Fig. 17g).

The US group (1.5 w/cm²) exhibited no significant differences in matrix metalloproteinase-2 (MMP-2) and matrix metalloproteinase-9 (MMP-9) levels in nasal mucosal tissue compared to the control group (0 w/cm²) (Supplementary Fig. 17h, i). MMP-2 and MMP-9 levels in the nasal mucosal tissue of the US (3.0 w/cm²) group rose by 1.71-fold and 3.38-fold, respectively (Supplementary Fig. 17h, i). These data indicate that US treatment at an intensity of 3.0 w/cm² induces “thermal injury” and inflammatory infiltration in the nasal mucosal tissue region of mice. LIPUS at 1.5 w/cm² intensity did not significantly alter mouse nasal mucosal tissue structure or cause inflammatory infiltration.

Further assessment of the effects of LIPUS on other key proteins of the cGMP/PKG/RhoA/RocK signaling pathway showed that these proteins were significantly correlated with NMB-related proteins. The US+iMPC-treated group showed a notable rise in PKG levels and a significant decrease in RhoA, RocK, ZO-1, Claudin-1, and Occludin levels in the nasal mucosal epithelium compared to the iMPC-treated group (Fig. 4e–k). In the LIPUS treatment groups, ultrasound intensities of 1.5 and 3.0 w/cm² significantly reduced the levels of RhoA, RocK, ZO-1, Claudin-1, and Occludin compared to the 0.5 w/cm² intensity group (Fig. 4e–k). Furthermore, there were no significant changes in the key proteins of the cGMP/PKG/RhoA/RocK signaling pathway and NMB tight junction protein levels in the free aPD-L1 and free PC treatment groups compared to the control group (Fig. 4e–k).

In vivo section staining and TEM were employed to examine the structural changes of NMB and confirm the impact of LIPUS on its structure. The study found that LIPUS treatment significantly disrupted the NMB structure, specifically the tight junction proteins Claudin-1 and Occludin, compared to the untreated group (Fig. 4l). The LIPUS treatment groups with ultrasound intensities of 1.5 and 3.0 w/cm² exhibited greater disruption of the tight junction proteins Claudin-1 and Occludin than the group with an ultrasound intensity of 0.5 w/cm² (Fig. 4l).

The trigeminal bypass serves as an essential route for nose-to-brain delivery, with its efficiency primarily constrained by crucial tight junction proteins like ZO-1^2,3,48^. Immunofluorescent and TEM images of mouse nasal mucosa revealed significant disruption of the trigeminal bypass in the LIPUS-treated group compared to the untreated group (Fig. 4m). In particular, the LIPUS-treated groups with ultrasound intensity of 1.5 and 3.0 w/cm² corresponded to more significant disruption of the trigeminal bypass compared to the LIPUS-treated group with ultrasound intensity of 0.5 w/cm² (Fig. 4m).

Hematoxylin and eosin (H&E) staining of the nasal mucosa revealed significant inflammatory cell infiltration when LIPUS at 3.0 w/cm² was combined with iMPC (Fig. 4n). LIPUS at an intensity of 3.0 w/cm² may excessively disrupt the NMB, heightening the risk of inflammatory cell infiltration. It highlights the primary reason for the greater efficiency of LIPUS in nose-to-brain delivery at 1.5 w/cm² rather than 3.0 w/cm².

The targeting efficiency of aPD‑L1 and carvedilol to GBM was compared between LIPUS‑mediated nasal‑to‑brain delivery of free aPD‑L1/carvedilol (PC + US group) and LIPUS‑mediated nasal‑to‑brain delivery of iMPC using the IVIS system. The study found that the PC + US group showed significantly improved targeted delivery of aPD-L1 to tumor tissues compared to the free PC group (Supplementary Fig. 18a, b). Furthermore, the iMPC+US group showed additional improvement in aPD-L1 tumor targeting compared to the PC + US group (Supplementary Fig. 18a, b). Quantitative analysis of aPD-L1 in brain tumor tissues via microplate reader revealed that the PC + US group achieved a 4.08-fold increase in tumor-targeted aPD-L1 delivery relative to the free PC group (Supplementary Fig. 18c). The iMPC+US group further enhanced aPD-L1 tumor targeting by 3.77-fold compared to the PC + US group (Supplementary Fig. 18c).

Fluorescence microscopy of tumor sections revealed that the PC + US group exhibited a 4.71-fold enhancement in aPD-L1-Cy5 fluorescence signal in GBM tumor tissues compared to the free PC group (Supplementary Fig. 18d, e). Compared with the PC + US group, the iMPC+US group showed a 4.01-fold elevation in aPD-L1-Cy5 fluorescence signal in GBM tumor tissues (Supplementary Fig. 18d, e). These findings indicate that LIPUS-mediated NMB opening promotes nose-to-brain delivery of free PC, whereas iMPC co-loading of aPD-L1 and carvedilol further enhances drug accumulation in GBM tumor tissues.

Further comparison of GBM targeting efficiency after iRGD-modified MPC revealed significantly enhanced tumor region signals in iMPC+US group compared to the MPC + US group (Supplementary Fig. 19a, b). Quantitative analysis using a multifunctional microplate reader revealed that aPD-L1 levels in the GBM mouse tumor region were 2.49 times higher in the MPC + US group compared to the Free PC group (Supplementary Fig. 19c). The iMPC+US group exhibited a 2.67-fold increase in aPD-L1 levels in the GBM mouse tumor region compared to the MPC + US group (Supplementary Fig. 19c). Confocal microscopy revealed significantly increased aPD-L1 distribution in tumor regions of iMPC+US GBM mice compared to MPC + US mice (Supplementary Fig. 19d). These findings demonstrate that iRGD-modified MPCs significantly enhance their targeting efficiency to GBM regions.

**Fig. 5:**
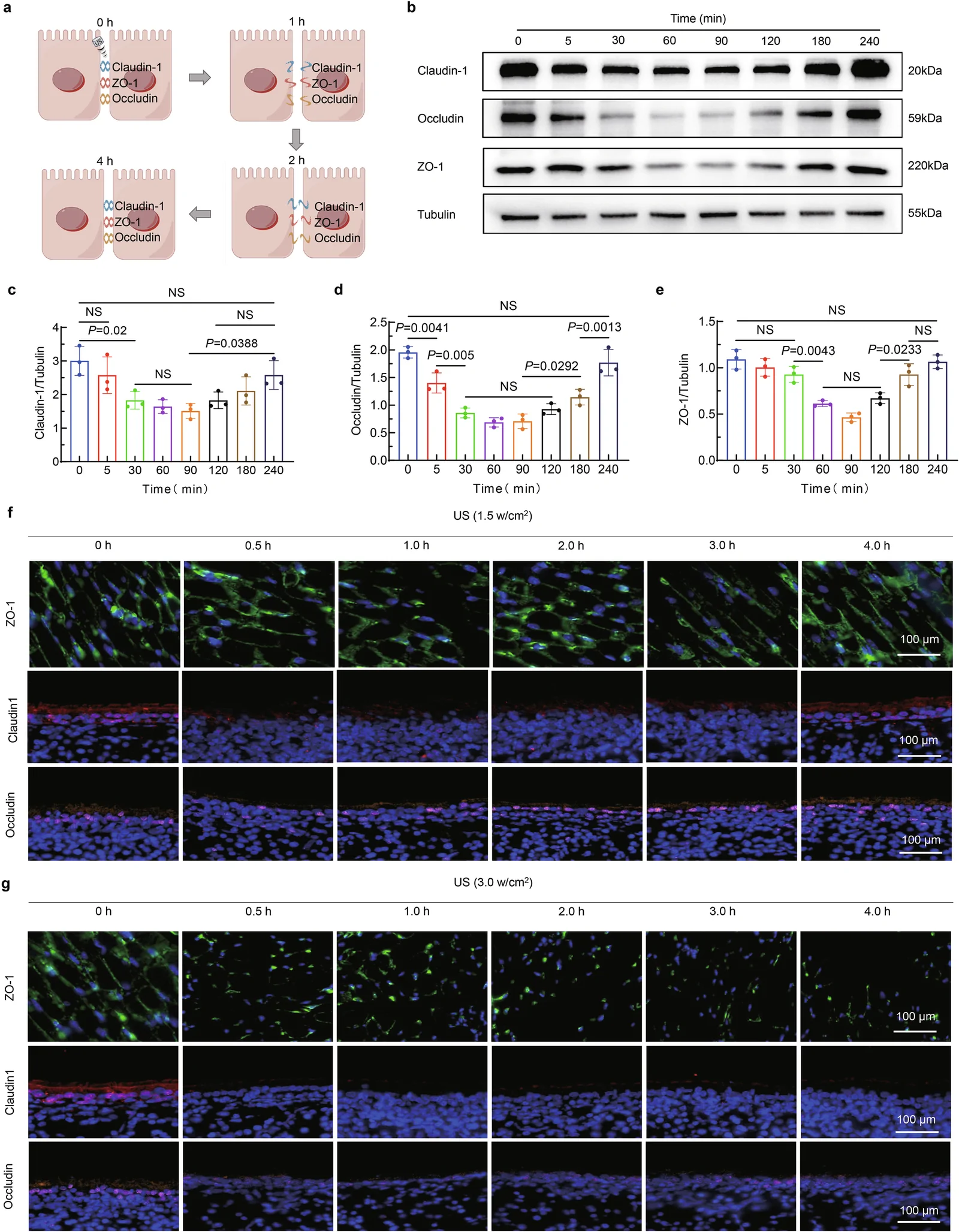
Low-intensity pulsed ultrasound (LIPUS) induces reversible opening of nasal mucosal barrier (NMB). **a** was created by Figdraw. **a** Diagram of LIPUS-mediated NMB opening (by figdraw.com). **b-e** Occludin, Claudin-1, and ZO-1 variations within 4 h after exposure to LIPUS at an intensity of 1.5 w/cm² for 60 s. **f**, **g** Occludin, Claudin-1, and ZO-1 changes within 4 h following LIPUS exposure at 1.5 w/cm² or 3.0 w/cm² for 60 s (blue: DAPI; red: CY3-Claudin-1; yellow: SpRed-Occludin), *n* = 3 samples. *n* = 3 samples per group for data in (**b–e**). Data are expressed as the mean ± SD. Statistical significances were determined using one-way ANOVA with Fisher’s LSD post‑hoc test. Comparisons were performed between different groups in (**c-e**). Not significant (NS) is *P* ≥ 0.05, and significant *P* values are shown. Source data are provided as a Source Data file.

We evaluated the impact of LIPUS at intensities of 1.5 w/cm² and 3.0 w/cm² on NMB-associated proteins to identify the optimal Ultrasound intensity range for safely and reversibly opening the NMB. The study found that LIPUS at 1.5 w/cm² intensity disrupted ZO-1, Claudin1, and Occludin, but these effects were reversible within 4 h. (Fig. 5a-f). After LIPUS treatment at an intensity of 3.0 w/cm², the disruption of ZO-1, Claudin1, and Occludin was not restored (Fig. 5g). This may be due to the fact that LIPUS with too high ultrasound intensity caused significant structural disruption of the NMB and was unable to restore the normal structure.

To assess the efficiency across the NMB of nanoparticle after LIPUS treatment (1.5 w/cm²), iMPC with diameters of 50, 100, 150, 200, and 300 nm were fabricated by adjusting extrusion membrane pore sizes (Supplementary Fig. 20a-e). IVIS imaging demonstrated that LIPUS-induced NMB opening (1.5 w/cm²) facilitated the transcytosis of nanoparticles ≤150 nm (Supplementary Fig. 20f, g). Among these, 50 and 100 nm iMPC exhibited the highest efficiency across the NMB (Supplementary Fig. 20f, g). Microplate reader quantification and ex vivo brain tissue imaging further confirmed the targeted accumulation of 50 and 100 nm iMPC in GBM regions (Supplementary Fig. 20h-i). Compared with 50 nm iMPC, LIPUS at 1.5 w/cm² decreased NMB penetration efficiency by 115.52% for 100 nm iMPC and 167.15% for 150 nm iMPC (Supplementary Fig. 20h–i). Compared to nasal-brain delivery of free iPMC at 50, 100, 150, and 200 nm, LIPUS-mediated nasal-brain delivery resulted in increases of 441.35%, 315.61%, 232.18%, and 124.98% (Supplementary Fig. 20j).

The results suggest that LIPUS enhances NMB opening through mechanical stress and modulates NMB opening in a safe and reversible manner by activating the cGMP/PKG/RhoA/ROCK signaling pathway, influencing the expression of tight junction proteins ZO-1, Claudin-1, and Occludin. LIPUS promotes iMPC (≤150 nm) cross NMB, with optimal efficiency observed in 50 nm and 100 nm iMPC. Furthermore, iMPC selectively accumulated in the GBM region, enabling responsive aPD-L1 release.

### LIPUS-mediated iMPC nose-brain delivery enhances GBM immunotherapy

**Fig. 6:**
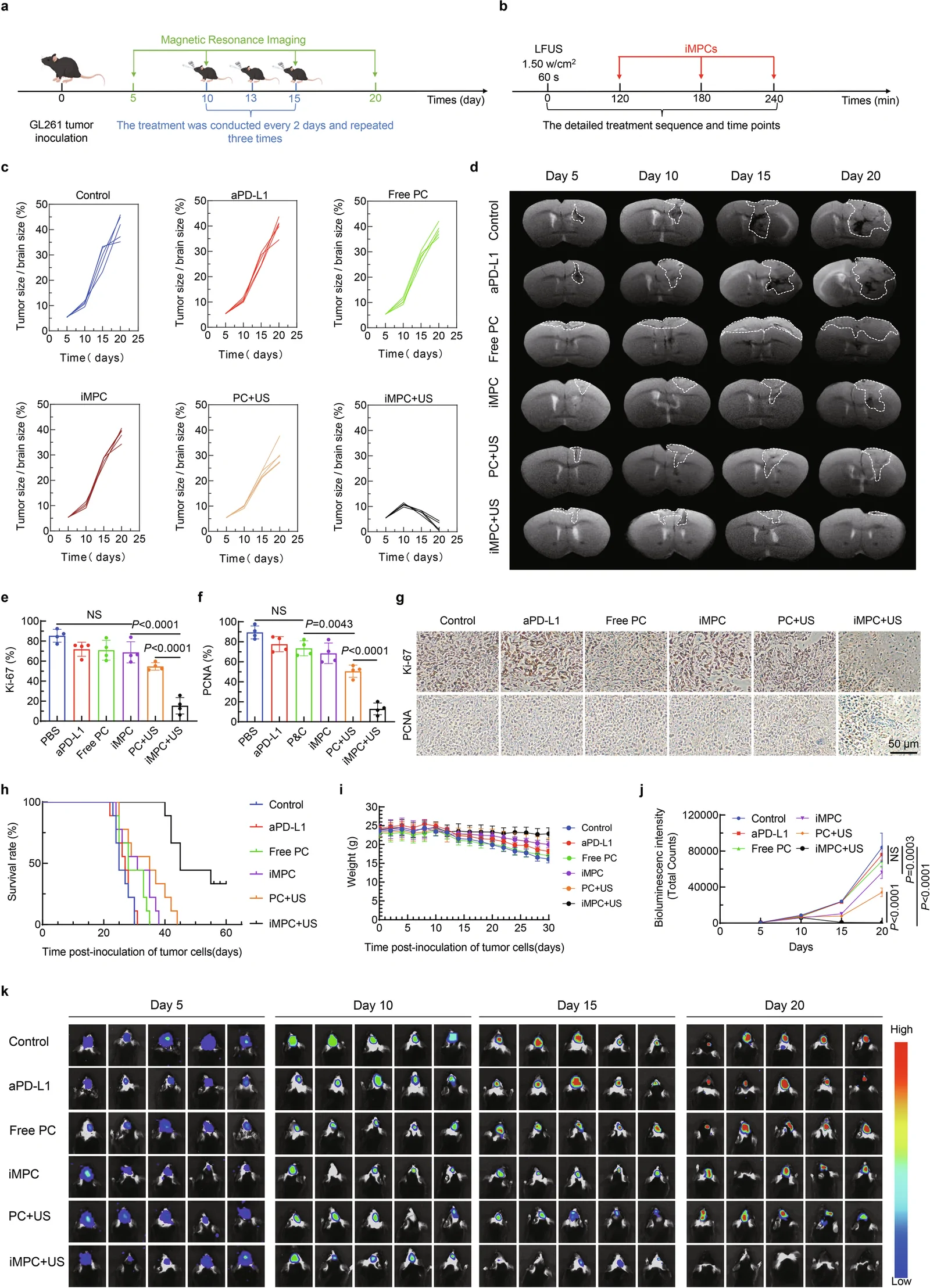
Antitumor efficacy on glioblastoma (GBM) mice. **a** was created by Figdraw. **a**, **b** Treatment schedule of the GL261 mouse brain tumor model (by figdraw.com). The detailed treatment sequence and time points. **c**, **d** Brain images of each group of mice obtained by T2 magnetic resonance imaging (MRI) with different treatments, where tumors are circled in white dashed lines. **e-g** Immunohistochemical images of tumor sections were examined at 15 days (Ki67 and PCNA, *n* = 5). **h** Survival curves of GBM tumor-bearing mice in different treatment groups (*n* = 10). Survival analysis was calculated using the log-rank test. **i** Body weight changes of GBM tumor-bearing mice in different treatment groups (*n* = 10). **j**, **k** Evaluation of antitumor effects of different treatments. *n* = 5 samples per group for data in (**c-f**) and (**i-k**). Data are expressed as the mean ± SD. Statistical significances were determined using one-way ANOVA with Fisher’s LSD post‑hoc test. Comparisons were performed between different groups in (**c-f**) and (**i-k**). Not significant (NS) is *P* ≥ 0.05, and significant *P* values are shown. *n *= 10 samples per group for data in (**h**). Survival analysis was compared using the log-rank test. Source data are provided as a Source Data file.

To further assess the antitumor efficacy of LIPUS-mediated iMPC nose-to-brain delivery, a detailed treatment plan was established (Fig. 6a, b). From the 10th day of GBM in situ mice model construction, one treatment was given every 2 days, totaling three treatments (Fig. 6a). The US+iMPC single-treatment regimen comprises LIPUS (acoustic intensity 1.5 w/cm^2^, duration 60 s, duty cycle 50%, frequency 1 MHz). Following LIPUS treatment, iMPC suspension was administered via intranasal instillation at 120, 180, and 240 min (Fig. 6b). Among them, the one-time treatment protocol of US+iMPC was that LIPUS (ultrasound intensity 1.5 w/cm^2^, time 60 s, duty cycle 50%, frequency 1 MHz) acted on the nasal mucosa of mice for 120, 180 and 240 min, and then iMPC suspension was given through the nose, respectively (Fig. 6b).

Tumor growth in GBM mice was monitored by magnetic resonance imaging (MRI) under different treatments. Untreated mice showed a 3.79-fold increase in tumor volume (Fig. 6c, d). Mice treated with Free aPD-L1, Free PC, and Free iMPC exhibited increases of 3.70-, 3.63-, and 3.80-fold, respectively. Mice treated with US + PC (free aPD-L1 and carvedilol) had a 3.08-fold increase, while those treated with US+iMPC showed a 5.34-fold decrease in tumor volume (Fig. 6c, d). These results suggest that LIPUS-mediated iMPC nose-to-brain delivery significantly inhibited GBM growth, even partially eliminating tumors compared to other treatments (Fig. 6c, d).

The proliferative activity of GBM following different treatments was evaluated by immunohistochemical staining of Ki-67 and PCNA. The findings indicated no notable differences in tumor proliferation among the Free aPD-L1, Free PC, or Free iMPC groups relative to the control group (Fig. 6e-g). The tumor proliferation rate in the US + PC group was reduced by 1.31-fold compared to the Free PC group (Fig. 6e-g). The US+iMPC group showed a 4.49-fold decrease in tumor proliferation rate compared to the iMPC group, suggesting that LIPUS improved the nose-to-brain delivery efficiency of iMPC, leading to enhanced tumor growth suppression (Fig. 6e-g). The tumor proliferation rate in the US+iMPC group was reduced by 3.57 times compared to the US + PC group, indicating that iRGD modification enhanced tumor-targeted drug delivery (Fig. 6e-g).

The therapeutic efficacy of LIPUS-mediated iMPC nose-to-brain delivery was further evaluated by statistically analyzing the survival period and body weight changes in GBM mice (Fig. 6h-i). The mean survival period of untreated control mice was 23.6 days, while those subjected to Free aPD-L1, Free PC, Free iMPC, and US + PC treatments exhibited mean survival periods of 24, 26, 28, and 31 days, respectively (Fig. 6h-i). Notably, among the 10 GBM mice treated with US+iMPC, two distinct outcomes were observed: the 6 mice that succumbed had a mean survival period of 44.8 days, while the remaining 4 mice survived beyond the 60-day observation period without mortality (Fig. 6h-i). Additionally, GBM mice treated with US+iMPC showed no significant changes in body weight (Fig. 6h-i). The findings suggest that GBM mice receiving LIPUS-combined iMPC treatment experienced significantly extended survival without significant alterations in body weight compared to other treatment groups.

A dual-luciferase-labeled GBM mouse model was developed and monitored dynamically with an IVIS to assess tumor progression. Immunofluorescence analysis indicated that the ideal multiplicity of infection (MOI) for GL261 cells transfected with Lent-EF1a-P2A-luciferase-CMV-coGFP-P2A-Puro lentivirus was 100 (Supplementary Fig. 21a). The CCK-8 assay determined the IC_50_ of puromycin for GL261 cells to be 2.5 µg/mL, facilitating the establishment of a stable GL261-Luc cell line co-expressing GFP and luciferase (Supplementary Fig. 21b, c). The ideal detection window for dual-luciferase-labeled GBM mice, derived from the GL261-Luc cell line, using IVIS was determined to be 6–14 min (Supplementary Fig. 21d, e).

IVIS monitoring indicated that neither the Free aPD-L1 nor the Free PC groups exhibited significant tumor growth inhibition compared to the control group (Fig. 6j, k). In GBM mice, tumor growth was notably suppressed in the US + PC group compared to the Free PC group, with even greater inhibition observed in the US+iMPC group (Fig. 6j, k). These results suggest that LIPUS-mediated iMPC nose-to-brain delivery has a significant anti-tumor effect in the GBM mouse model.

TUNEL apoptosis staining was further performed on the tumor tissues of GBM mice after different treatments to evaluate the apoptosis rate of the tumors. The study found that the apoptosis rates in tumor tissues increased by 1.84, 2.02, 2.01, 2.88, and 9.24 times in the Free aPD-L1, Free PC, iMPC, US + PC, and US+iMPC groups, respectively, compared to the control group (Supplementary Fig. 21f, g). Notably, the apoptosis rate of tumor tissues was 4.61-fold higher in the US+iMPC group compared with the iMPC group, respectively (Supplementary Fig. 21f, g). This may be due to the LIPUS-mediated NMB opening, which greatly improved the iMPC nose-to-brain delivery efficiency and tumor-targeting enrichment.

The G422-Luc cell line-derived GBM mice showed notably reduced tumor growth in the PC + US group relative to the Free PC group, as evidenced by IVIS antitumor assessment (Supplementary Fig. 22a, b). Tumor growth in the iMPC+US group was further inhibited relative to the PC + US group (Supplementary Fig. 22a, b). The survival period of GBM mice was extended to 35 days in the PC + US group and 60 days in the iMPC+US group, compared to the control group (Supplementary Fig. 22c).

Compared to other groups where body weight of GBM mice began to decrease on day 10, the iMPC+US group showed no significant weight loss (Supplementary Fig. 22d). Tumor tissue apoptosis rates were 2.19 times higher in the PC + US group and 7.47 times higher in the iMPC+US group compared to the control group (Supplementary Fig. 22e, f). The data suggest that LIPUS-enhanced iMPC nasal-to-brain drug delivery significantly suppresses tumor growth in GBM mice.

These results show that combining LIPUS-mediated nose-to-brain delivery with iMPC significantly boosts antitumor effects in GBM-bearing mice by inhibiting tumor growth, inducing apoptosis, extending survival time, and enhancing overall survival.

### LIPUS-mediated iMPC nose-to-brain delivery enhances T-cell antitumor activity

Transcriptome sequencing can identify dynamic alterations in gene expression within tumor tissues, highlighting changes in the activity of various cell types in the TME, including T cells, tumor-associated macrophages, fibroblasts, and other immune cells. To evaluate and predict changes in the proportion and activity of immune cells, including T cells, within the TME of GBM mice, we conducted transcriptome sequencing, flow cytometry, and immunofluorescence assays on tumor tissues from both control and US+iMPC groups.

**Fig. 7:**
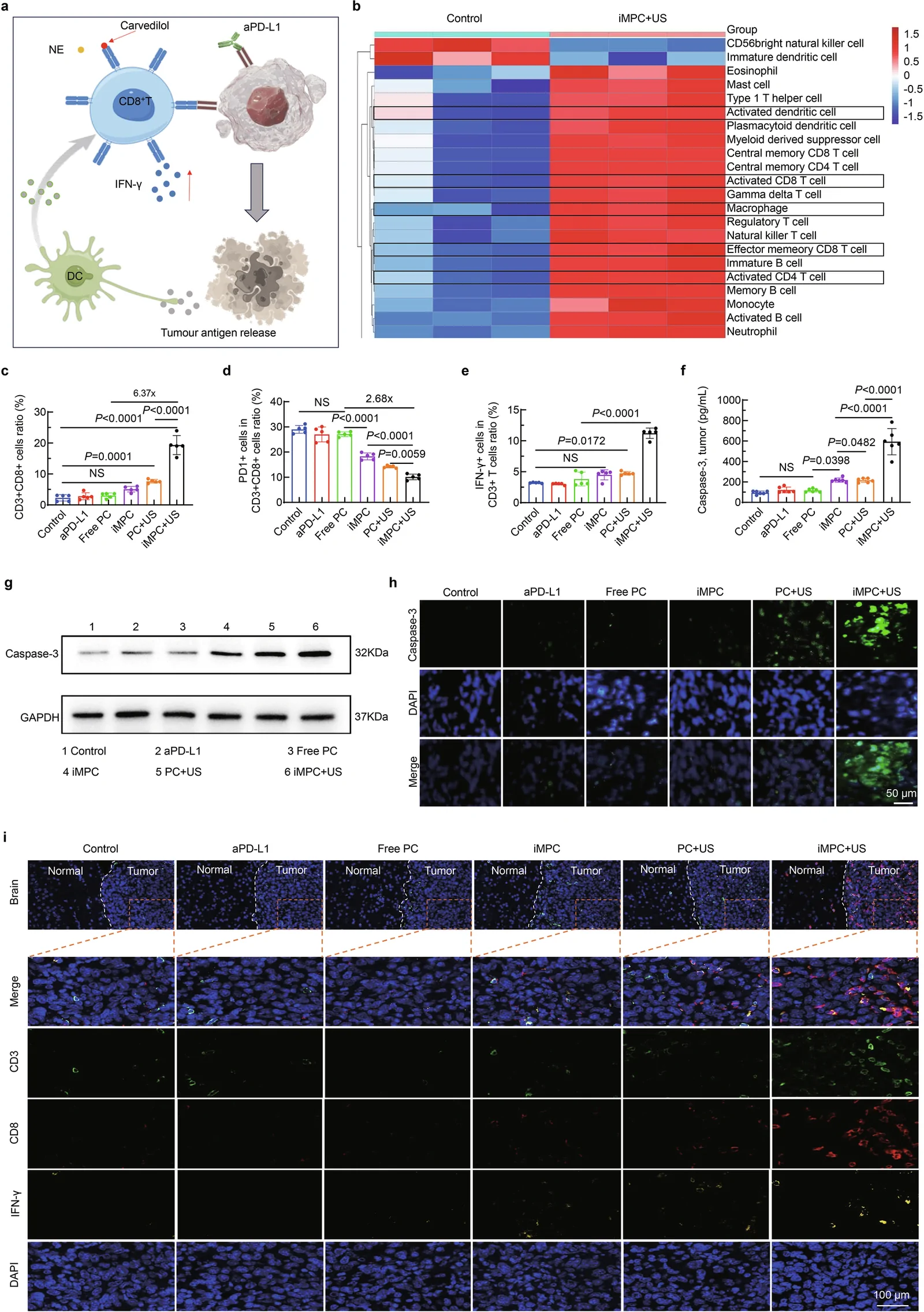
Evaluation of T cell antitumor activity in glioblastoma (GBM) mice. **a** was created by Figdraw. **a** Schematic diagram of carvedilol blocking norepinephrine-induced T-cell exhaustion combined with anti-programmed cell death ligand 1 (aPD-L1) to enhance immunotherapy for GBM (by figdraw.com). **b** Enrichment analysis of differentially expressed genes from transcriptome sequencing of tumor tissues. **c** The infiltration ratio of CD3^+^CD8^+^ T cells in tumor tissues after 5 days of different treatments. **d** The expression of PD1 in CD3^+^CD8^+^ T cells in tumor tissues after 5 days of different treatments. **e** The expression of Tumor-specific T cells in tumor tissues after 5 days of different treatments. **f** The expression of caspase-3 in tumor tissues by enzyme-linked immunosorbent assay (ELISA). **g** The expression of caspase-3 in tumor tissues, ImageJ software statistical analysis, *n* = 3 samples. **h** The expression of caspase-3 in tumor tissues, *n* = 3 samples. **i** Tumor-specific T cell infiltration in tumor tissues after 5 days of different treatments (blue: DAPI; green: Alexa Fluor 488-CD3; red: CY3-CD8; yellow: SpRed-IFN-γ), *n* = 3 samples. *n* = 5 samples per group for data in (**c-f**). Data are expressed as the mean ± SD. Statistical significances were determined using one-way ANOVA with Fisher’s LSD post‑hoc test. Comparisons were performed between different groups in (**c-f**). Not significant (NS) is *P* ≥ 0.05, and significant *P* values are shown. Source data are provided as a Source Data file.

Transcriptomic sequencing and flow cytometry analyses demonstrated that LIPUS-combined iMPC therapy in GBM mice activated various immune cell populations, such as T cells, compared to the control group (Fig.7a, b, Supplementary Fig. 23a-d, and Supplementary Fig. 24a-c). In GBM-bearing mice, the US + PC and US+iMPC groups showed significantly increased CD3^+^CD8^+^ T cell infiltration (3.34-fold and 8.47-fold, respectively) compared to the control, with no significant differences observed between the control and free aPD-L1, free PC, or iMPC groups (Fig.7c and Supplementary Fig. 25a). The US + PC and US+iMPC groups exhibited 2.51-fold and 6.37-fold increases, respectively, in CD3^+^CD8^+^ T cell proportions within GBM tumors compared to the free PC group (Fig.7c and Supplementary Fig. 25a). The findings demonstrate a notable increase in T cell populations within the TME of GBM mice treated with US+iMPC.

The study further assessed the proportion of PD1-expressing CD3^+^CD8^+^ T cells within the TME. Flow cytometry analysis showed no significant differences in the percentage of PD1^+^ CD3^+^CD8^+^ T cells within the TME of GBM mice treated with Free aPD-L1 or Free PC compared to the control group (Fig. 7d and Supplementary Fig. 25b). GBM mice treated with US + PC showed a 1.91-fold decrease in PD1^+^CD3^+^CD8^+^ T cell infiltration in tumor tissues compared to those treated with Free PC (Fig.7d and Supplementary Fig. 25b). More strikingly, a 2.68-fold decrease was observed in the US+iMPC treatment group compared to Free PC group (Fig. 7d and Supplementary Fig. 25b). Immunofluorescence multiplex staining demonstrated a notable reduction in the infiltration ratio of PD1^+^CD8^+^ T cells in tumor tissues of US+iMPC-treated GBM mice compared to other treatment groups (Supplementary Fig. 25c). The study shows that LIPUS-facilitated nose-to-brain delivery of iMPC significantly enhances the T cell ratio in the TME and reduces T cell exhaustion.

Tumor-specific T cells, the main T cell population responsible for tumor recognition and elimination, play a decisive role in determining the efficacy of tumor immunotherapy^17,22,35^. The study found no significant differences in the proportion of tumor-specific T cells (IFNγ^+^CD3^+^ T cells) within GBM tumor tissues among the free aPD-L1, free PC, and iMPC groups compared to the control group (Fig.7e and Supplementary Fig. 25d). However, the US+iMPC group exhibited a 2.93-fold increase in tumor-specific T cell infiltration relative to the free PC group (Fig. 7e and Supplementary Fig. 25d).

Caspase-3 levels in GBM mouse tumor tissues were quantitatively analyzed using enzyme-linked immunosorbent assay (ELISA). The study found no significant change in caspase-3 levels in tumor tissues of GBM mice treated with free aPD-L1 and free PC compared to the control group (Fig. 7f). The caspase-3 content in tumor tissues of GBM mice increased by 1.86-fold in the iMPC group, 1.84-fold in the US + PC group, and 5.08-fold in the US+iMPC group compared to the free PC group (Fig. 7f).

WB analyses demonstrated that GBM mouse tumor tissues treated with iMPC showed a significant increase in caspase-3 expression compared to those treated with free PC (Fig. 7g and Supplementary Fig. 25e). Furthermore, GBM mouse tumor tissues treated with US+iMPC exhibited significantly higher caspase-3 expression compared to those treated with free iMPC alone (Fig. 7g and Supplementary Fig. 25e).

Immunofluorescence staining revealed that GBM mice tumor tissues treated with US+iMPC exhibited notably higher caspase-3 expression than those in other treatment groups (Fig. 7h). These findings suggest that LIPUS-enhanced nose-to-brain delivery of iMPC promotes T cell-mediated destruction of tumor cells in the GBM region. Multiplex immunofluorescence analysis confirmed that GBM tumor tissues from US+iMPC-treated mice displayed significantly enhanced infiltration of tumor-specific T cells compared to all other treatment groups (Fig. 7i). These data indicate that LIPUS-mediated iMPC nose-to-brain delivery improves tumor-specific T cell infiltration in GBM. The findings suggest that LIPUS-facilitated nose-to-brain delivery of iMPC increases CD3^+^CD8^+^ T cell levels, decreases PD1-expressing CD3^+^CD8^+^ T cells, and boosts tumor-specific T cells in the TME of GBM mice.

### Enhanced activity of dendritic cells (DCs) and CD8^+^ T cells in cervical deep lymph nodes

Additional analysis of DC, CD4^+^ T cell, and CD8^+^ T cell activation in lymph nodes is provided in Supplementary Fig. 26. Flow cytometry of deep cervical lymph nodes showed an increase in DC proportions by 128.39% in the PC + US group and 154.90% in the iMPC+US group compared to the control group (Supplementary Fig. 27a, d). The iMPC group exhibited a 3.49-fold increase in the proportion of DCs with high CD80 expression compared to the control group (Supplementary Fig. 27b, e). Compared to the Free PC group, the proportion of DCs highly expressing CD80 increased 6.52-fold in the PC + US group and 9.21-fold in the iMPC+US group (Supplementary Fig. 27b, e). Compared with the iMPC group, the proportion of DCs highly expressing CD80 increased 1.42-fold in the PC + US group and 2.01-fold in the iMPC+US group (Supplementary Fig. 27b, e). The proportion of DCs with high CD86 expression increased by 119.16% in the PC + US group and by 144.52% in the iMPC+US group compared to the Free PC group (Supplementary Fig. 27c, f). These data indicate that LIPUS-mediated iMPC naso-cerebral delivery induces DCs activation in deep cervical lymph nodes.

Analysis of DCs activity in ipsilateral axillary lymph nodes showed a 1.25-fold increase in the PC + US group and a 1.50-fold increase in the iMPC+US group relative to the control group (Supplementary Fig. 28a, d). Importantly, none of the experimental groups, including iMPC+US, exhibited significantly higher CD86 or CD80 expression on DCs compared to the control group (Supplementary Fig. 28b, c, e, f). This indicates that LIPUS-mediated iMPC naso-cerebral delivery primarily influences local immune regulation within the brain tumor region.

Increased DC activity frequently stimulates T cells, necessitating further assessment of CD8^+^ and CD4^+^ T cell activity in deep cervical lymph nodes^40^. The study found that the T cell proportion increased by 146.18% in the PC + US group and by 173.69% in the iMPC+US group compared to the control group (Supplementary Fig. 29a, d). The PC + US group exhibited a 1.66-fold reduction in PD1^high^ CD8^+^ T cell proportion compared to the control group, while the iMPC+US group showed a 7.78-fold decrease (Supplementary Fig. 29b, e). Compared with the iMPC group, the proportion of PD1^high^ CD8^+^ T cells decreased 4.95-fold in the iMPC+US group (Supplementary Fig. 29b, e). The PC + US group exhibited a 1.66-fold reduction in PD-1^high^ CD8^+^ T cells, while the iMPC+US group showed an 8.83-fold decrease, compared to the control group (Supplementary Fig. 29c, f). The iMPC+US group exhibited a 5.57-fold reduction in the proportion of Tim3^high^ CD8^+^ T cells compared to the iMPC group (Supplementary Fig. 29c, f). These data indicate that LIPUS-mediated iMPC naso-cerebral delivery induces an increased proportion of T cells in deep cervical lymph nodes and enhanced CD8^+^ T cell activity.

The evaluation of CD4^+^ T cell activity in deep cervical lymph nodes revealed that the proportion of IFN-γ^high^ CD4^+^ T cells increased by 3.57 times in the PC + US group and 5.85 times in the iMPC+US group compared to the control group (Supplementary Fig. 30a, c). The iMPC+US group exhibited a 1.79-fold increase in the proportion of IFN-γ^high^ CD4^+^ T cells compared to the iMPC group (Supplementary Fig. 30a, c). The proportion of TNF-α^high^ CD4^+^ T cells increased by 2.27-fold in the PC + US group and 2.97-fold in the iMPC+US group compared to the control group (Supplementary Fig. 30b, d). The iMPC+US group exhibited a 1.39-fold increase in the proportion of IFN-γ^high^ CD4^+^ T cells compared to the iMPC group (Supplementary Fig. 30b, d). These data indicate that LIPUS-mediated iMPC nasal-brain delivery simultaneously enhances CD4^+^ T cell activity in deep cervical lymph nodes.

### Immunosuppressive TME is reactivated in GBM mice

**Fig. 8:**
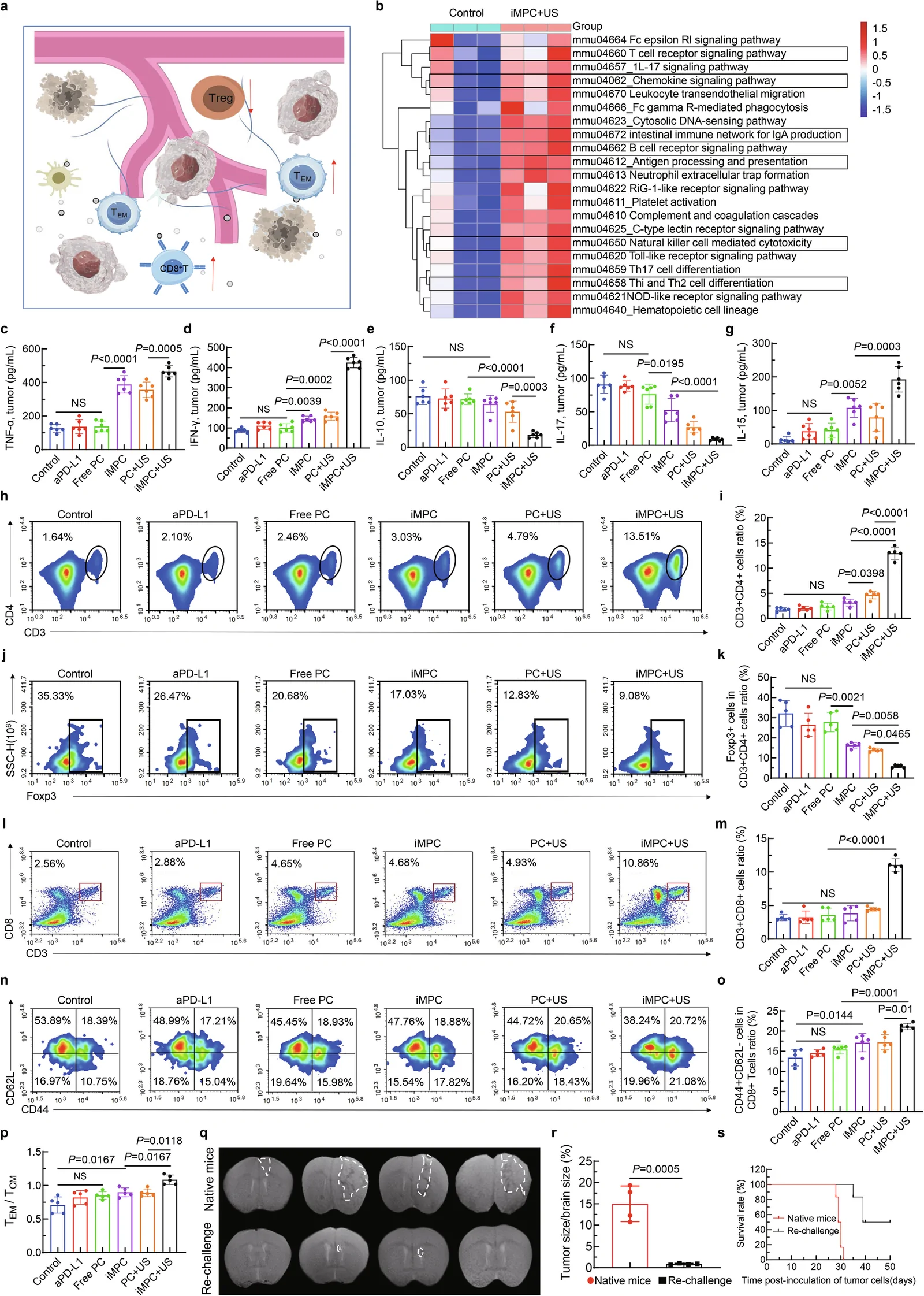
Reprogramming of the immunosuppressive tumor microenvironment (TME). **a** was created by Figdraw. **a** Schematic diagram of key immune cell changes in the tumor microenvironment (by figdraw.com). **b** Enrichment analysis on differentially expressed genes identified through transcriptome sequencing of tumor tissues to evaluate the activation status of multiple immune signaling pathways. **c-g** Changes in the levels of key cytokines such as TNF-α, IFN-γ, IL-10, IL-17, and IL-15 in the tumor microenvironment. **h, i** Changes in the proportion of CD3^+^CD4^+^ T cells in the tumor microenvironment after different treatments. **j**, **k** Changes in the Treg cell population in the tumor microenvironment following various treatments. **l**, **m** Alterations in the proportion of CD3^+^CD8^+^ T cells in the tumor microenvironment after different treatments. **n-p** The proportion of memory T cells in the tumor microenvironment after different treatments. **q-s** Brain images of mice from each group using T2 magnetic resonance imaging (MRI) after receiving different treatments, with tumors outlined in white dashed lines. The survival periods of GBM-bearing mice and mice undergoing tumor rechallenge. *n *= 6 samples per group for data in (**c-g**). *n* = 5 samples per group for data in (**h-p**). Data are expressed as the mean ± SD. Statistical significances were determined using one-way ANOVA with Fisher’s LSD post‑hoc test. Comparisons were performed between different groups in (**c-g** and **h-p**). Not significant (NS) denotes *P* ≥ 0.05, and significant *P* values are shown. *n* = 4 samples per group for data in (**q**, **r**). Statistical analysis between two groups was performed using unpaired two-tailed Student’s *t* test. n = 5 samples per group for data in **s**. Survival analysis was compared using the log-rank test. Source data are provided as a Source Data file.

To further evaluate the impact of LIPUS-combined iMPC on the immunosuppressive TME, transcriptome sequencing analysis was performed. The results demonstrated that LIPUS combined with iMPC could activate multiple anti-tumor immune signaling pathways in the TME of GBM-bearing mice (Fig. 8a-b and Supplementary Fig. 31a-f).

To assess the effects of LIPUS-facilitated nose-to-brain delivery of iMPC on the TME, inflammatory cytokines were quantitatively measured. ELISA analysis revealed that GBM mouse tumor tissues subjected to US+iMPC treatment showed a significant rise in pro-inflammatory cytokines (TNF-α, IFN-γ, and IL-15) and a notable reduction in immunosuppressive cytokines (IL-10 and IL-17) compared to other treatment groups (Fig. 8c-g). Compared to the free PC group, the US+iMPC group exhibited increases of 3.38-fold in TNF-α, 4.24-fold in IFN-γ, and 4.69-fold in IL-15, alongside reductions of 3.84-fold in IL-10 and 8.42-fold in IL-17 within GBM tumor tissues (Fig. 8c-g). These findings suggest that LIPUS-facilitated nose-to-brain delivery of iMPC successfully reactivates inflammatory responses in the TME of GBM mice.

Flow cytometry was employed to evaluate the immune activation status of the TME by analyzing the proportions of different immune cells in the tumor tissues of GBM mice. The study found that in GBM mice, US + PC treatment led to a 1.98-fold increase in CD3^+^CD4^+^ T cells and a 1.99-fold decrease in Tregs within the TME compared to the free PC group (Fig. 8h-k). Immunofluorescence multilabel staining indicated a notable decrease in Tregs cell infiltration in the tumor tissues of GBM mice treated with the US+iMPC group compared to other treatment groups (Supplementary Fig. 32a). These findings indicate that LIPUS-facilitated nose-to-brain delivery of iMPC increases CD3^+^CD4^+^ T cell levels in the GBM TME and simultaneously decreases immunosuppressive Tregs.

Previous research indicates that TME activation frequently leads to memory T cell generation^13,37,49^. The analysis of elevated pro-inflammatory cytokines TNF-α, IFN-γ, and IL-15 in the GBM TME, in relation to immune cells, indicates potential activation of effector memory T cells (T_EM_) (Supplementary Fig. 32b). Analysis of memory T cells in GBM mouse tumor tissues showed that the US+iMPC group had a 1.37-fold higher proportion of T_EM_ within the TME compared to the free PC group, with no notable change in TCM proportions (Fig.8l-o and Supplementary Fig. 32c). The T_EM_/T_CM_ ratio in tumor tissues of US+iMPC-treated GBM mice showed a 1.28-fold increase compared with the free PC group (Fig. 8p). Furthermore, this ratio was 1.21-fold higher in the US+iMPC group relative to the free iMPC group (Fig. 8p). The findings suggest that LIPUS-facilitated nose-to-brain delivery of iMPC enhances the immunosuppressive tumor microenvironment in GBM and notably increases the proportion of TEM cells.

Tumor rechallenge, where cured tumor model mice are re-implanted with tumor cells, is commonly used to assess the long-term immune memory formed by immunotherapy strategies^13^. To further evaluate the formation of long-term immune memory in GBM mice treated with LIPUS combined with iMPC, MRI was used to examine the tumor size of mice receiving different treatments (Fig. 8q-s). The results demonstrated that tumor growth was significantly suppressed, and survival was markedly prolonged in the US+iMPC group compared to native mice (untreated GBM mice) group (Fig. 8q-s). Immunofluorescence analysis demonstrated a notable increase in infiltrating memory T cells within tumor tissues of US+iMPC-treated mice compared to the native mice group (Supplementary Fig. 32d). The study demonstrates that LIPUS-facilitated nose-to-brain delivery of iMPC boosts T cell antitumor activity, counteracting the immunosuppressive TME in GBM mice and promoting long-term immune memory.

### LIPUS-mediated iMPC nose-brain delivery is a safe strategy for GBM

To further assess the safety of LIPUS-combined iMPC therapy, hematological and blood biochemical analyses were performed. The study found no significant changes in blood cell components—white blood cells, red blood cells, platelets, and hemoglobin—in GBM-bearing mice across various treatment groups, with all parameters staying within normal ranges (Supplementary Fig. 33a–j). Plasma levels of alanine aminotransferase (ALT), aspartate aminotransferase (AST), total bilirubin (TBIL), urea, and creatinine (Crea) in GBM mice across different treatment groups remained within normal physiological ranges (Supplementary Fig. 33k–p).

H&E staining of the heart, liver, spleen, lungs, and kidneys showed no significant tissue damage across all treatment groups (Supplementary Fig. 33q). These findings indicate that LIPUS-mediated nasal-to-brain delivery of iMPC represents a safe strategy for enhancing immunotherapy in GBM.

## Discussion

The nose-to-brain delivery route provides benefits over traditional systemic administration by bypassing the blood-brain barrier, minimizing systemic toxicity, and circumventing hepatic first-pass metabolism^7,14,50^. However, the NMB poses a significant challenge to efficient drug delivery^2,3^. Our study addresses this issue by using LIPUS to temporarily and reversibly open the NMB, enhancing the permeability of iMPC. Additionally, the use of pH-responsive iMPC ensures that the drugs are released in the acidic TME of GBM, further enhancing their efficacy. This method ensures that the drugs are delivered directly to the brain, achieving higher local concentrations and better therapeutic outcomes^17,18^.

In this study, LIPUS, without depending on the MBs to amplify energy, is directly used to reversibly open the NMB by disrupting tight junction proteins^8,10^. The NMB exhibits relatively loose architecture and higher tunability^4,7,14^. Through LIPUS application, the permeability of the NMB can be directly enhanced, thereby facilitating efficient drug delivery without relying on MBs-mediated enhancement^13,40^. This approach offers distinct advantages by simplifying the treatment protocol and eliminating MBs usage, consequently reducing operational complexity and potential side effects^8,10^. Overall, LIPUS-mediated nose-to-brain delivery significantly improves drug utilization efficiency while ensuring a more streamlined, comfortable, and controllable therapeutic procedure, demonstrating broad application prospects^8,14^.

Traditional methods to overcome NMB, such as mucoadhesive nanoparticles or enzymatic inhibitors, often cause chronic inflammation or compromise mucosal integrity^2–4,6,14^. LIPUS at 1.5 w/cm² intensity does not cause significant inflammation and enables NMB recovery within 4 h. This temporary and regulated opening preserves barrier function, reducing infection risk and other adverse effects^14^. The safety of this approach is further supported by hematological and blood biochemical analyses, which show no significant alterations in blood cell components or organ damage in GBM mice. Optimizing acoustic parameters for LIPUS-mediated iMPC nose-to-brain delivery is crucial to ensuring treatment efficacy and safety^7,10^. This study identifies optimal parameters as a 1.0 MHz center frequency, 1.5 w/cm² ultrasound intensity, a 30% duty cycle, and a 60-s treatment duration. These parameters strike a balance between maximizing the disruption of the NMB to facilitate iMPC penetration and minimizing potential irreversible damage to the tight junctions. The 1.0 MHz frequency allows for deeper tissue penetration, while the 1.5 w/cm² intensity ensures adequate disruption of the NMB without causing irreversible damage to surrounding tissues. A duty cycle of 30% provides a continuous yet controlled energy release, which enhances the effectiveness of iMPC delivery while maintaining safety. The 60-s exposure time optimizes the therapeutic effect while allowing for the reversibility of NMB disruption. This combination of acoustic parameters represents a significant advancement in non-invasive, targeted drug delivery strategies for brain tumors and other neurological disorders^7,10^.

Previous studies on β-blockers in GBM mainly focused on their direct anti-tumor effects, such as inhibiting proliferation, migration, or angiogenesis via β-adrenergic receptors (β-ARs) on cancer cells^18,21,23^. Our study emphasizes the previously underexplored immunomodulatory impact of β-blockers on tumor-infiltrating lymphocytes (TILs), specifically CD8^+^ T cells and Tregs^19–22,51^. Flow cytometry and multiplex cytokine analysis revealed that carvedilol-loaded iMPC significantly reduced the proportion of exhausted T cells (from 29.32% to 9.44%) while decreasing Tregs infiltration (from 35.33% to 9.08%) in GBM tissues. The role of β-adrenergic signaling in tumor progression and immune evasion involves activating cAMP-dependent pathways that inhibit cytotoxic T-cell activity. By achieving spatially controlled release of carvedilol within the TME, our strategy effectively blocks β-ARs on both T cells and immunosuppressive Tregs. This inhibition reduces the secretion of immunosuppressive cytokines (e.g., IL-17, IL-10) and suppresses downstream neuro-signaling cascades, such as the PKA/CREB pathway, which are aberrantly activated in T cells^18,22,35^.

In GBM mice model, a strategy using LIPUS-mediated pH-responsive bionic nanovesicles (iMPC) for nose-to-brain delivery was proposed to enhance immunotherapy. LIPUS is used to reversibly modulate the NMB, creating a two-h window for enhanced nose-to-brain delivery. The iMPC co-delivers β-blockers (carvedilol) and aPD-L1 to mitigate T-cell exhaustion and improve T-cell antitumor activity^17,21,22^. Our findings indicate that this integrated method notably enhances aPD-L1 concentration in the brain, reactivates CD8^+^ T cells, achieves a 40% tumor elimination rate in orthotopic GBM mice, and results in extended survival and long-term immune memory establishment. Notably, our findings reveal that the targeted release of β-blockers not only mitigates T-cell exhaustion but also disrupts multiple neuro-signaling pathways in GBM, a dual mechanism with significant therapeutic implications (Supplementary Fig. 34a-e)^17^. Future studies should investigate whether this immunomodulatory effect is β-AR subtype-specific (e.g., β1 vs. β2) or involves cross-talk with other neurotransmitter pathways^18,21^. Additionally, exploring the interplay between β-AR inhibition, neuroimmune modulation, and tumor metabolism may uncover novel targets for combinatorial GBM therapies^17,22^.

The success of LIPUS in enhancing the delivery of iMPC to the brain holds significant promise for treating other neurological disorders beyond GBM^3,6,7,39^. Numerous neurological disorders, including Alzheimer’s, Parkinson’s, and multiple sclerosis, encounter common obstacles in drug delivery due to the BBB and NMB^3,4,52^. LIPUS can transiently open the NMB, potentially enabling the direct delivery of various therapeutic agents, such as small molecules, peptides, and gene therapies, to the brain. This approach could potentially revolutionize the treatment of these conditions by improving drug bioavailability and efficacy while reducing systemic side effects^1,3^. Future studies should explore the application of LIPUS in combination with targeted nanocarriers for the treatment of various neurological disorders, paving the way for new therapeutic paradigms^7^.

To further enhance the effectiveness and safety of LIPUS in modulating the NMB, future improvements in US equipment are essential^8,10^. Current US devices used in clinical settings may not be optimized for the specific requirements of NMB modulation^10^. Advanced US systems with higher precision and real-time feedback mechanisms could enable more precise control over the intensity and duration of LIPUS exposure^8,10^. Additionally, the development of wearable US devices could allow for more frequent and consistent treatments, potentially extending the therapeutic window and improving patient compliance^11^. Integrating artificial intelligence (AI) algorithms to optimize ultrasound parameters based on individual patient data could further personalize the treatment, maximizing therapeutic benefits while minimizing side effects^53^.

In conclusion, we propose a strategy utilizing LIPUS to induce the opening of transient NMB. The ability to safely and reversibly modulate the NMB using LIPUS represents a significant advancement in targeted brain therapy. In addition, this study presents a groundbreaking approach to enhancing GBM immunotherapy by combining LIPUS with pH-responsive bionic nanovesicles for nose-to-brain delivery. This approach mitigates T-cell exhaustion, enhances their anti-tumor efficacy, and results in notable tumor suppression and extended survival in GBM mice. LIPUS-facilitated nose-to-brain nanoparticle delivery presents a promising therapeutic strategy for GBM and potentially other brain disorders^1,3,54^. LIPUS is expected to provide a method for bypassing physiological barriers like the blood-retinal barrier, placental barrier, and mucus barrier, thereby aiding in the treatment of multiple diseases^55–57^. As a physical energy wave form, LIPUS does not exhibit drug-drug interactions, has broad applicability, and does not induce long-term alterations in biological barrier structures, thereby providing safer and more stable therapeutic outcomes.

## Methods

### Ethics statements

All procedures involving human participants adhered to the ethical standards set by the Life Science Ethics Review Board of Zhengzhou University (ZZUIRB2024-191). For studies involving human tumor tissue sections, participants’ gender and/or sex are determined based on self-report. The risks to participants in this study do not exceed those of routine clinical medical procedures, and this research is categorized as a minimal-risk study with no additional therapeutic risks. All personal information and relevant research data of participants are strictly kept confidential. Tumor pathological specimens are identified only by anonymized study codes rather than patient names. No personally identifiable information will be released to external third parties beyond the research team without explicit participant consent. All research staff and study sponsors are obligated to protect participant privacy and maintain strict confidentiality. Original research archives are securely stored in dedicated locked cabinets at the School of Nursing and Health, Zhengzhou University, with access restricted exclusively to authorized researchers. For the purpose of ethical supervision and research compliance, authorized personnel from governmental regulatory bodies and institutional ethical review committees may access relevant participant records in accordance with official regulations. Certain questionnaire items may induce transient emotional discomfort among participants, who retain the right to decline to answer any uncomfortable questions at any time. When the research findings are published, strict anonymization and confidentiality protocols will be fully implemented to ensure that no personally identifiable participant information is disclosed.

C57BL/6J and BALB/c mice, both male and female (6–8 weeks, 20–22 g), were obtained from Jiangsu Huachuang Sino Pharma Tech Co., Ltd. The mice were co-housed in the specific pathogen-free (SPF) care facility with free access to food and water under a 12 h light/dark cycle. All animal experiments adhered to relevant laws and received approval from the Institutional Animal Care and Use Committee at Southeast University School of Medicine (No. 20220316049). None of the tumors reached a volume of 100 mm^3^ (the maximum tumor size). In survival analysis, endpoints for euthanasia include not only death but also moribund conditions in mice, such as unresponsiveness to stimuli, immobility, or inability to eat or drink. This study strictly adheres to the “Sex and Gender Equity in Research—SAGER—guidelines.” For experiments involving animal testing, males and females were randomly assigned.

### Materials

Carvedilol was purchased from Sigma-Aldrich (PHR1265, Poznań, Poland). aPD-L1 was purchased from Shanghai Taoshu Bioscience Co. (T77189, TargetMol, USA). NHS-PEG4-MAL was purchased from Xi’an Rui Xi Biotechnology Co., Ltd. (R-1083-2k, Xian, China). The PASP was purchased from Alamanda Polymers (Huntsville, AL, USA). Fetal Bovine Serum (KX-A1202) was sourced from Inner Mongolia Wanrui Biotechnology Co., Ltd., Neimenggu, China. A 24-well chamber was purchased from SAINING LIFE SCIENCES Co., Ltd. (1102000, Zhejiang, China). FEP cell culture bags were purchased from Yocon Biotechnology Co., Ltd. (FC0102, Beijing, China). A T75 cell culture flask was purchased from PakGent Bioscience Co., Ltd. (CL-FT075P, Suzhou, China).

Multicolor immunofluorescence staining of tumor tissue is supported by Zhongmo Biology. Mito-Tracker Green was purchased from KeygenBioTECH Co., Ltd. (KGE2704-50, Jiangsu, China). Gel/PCR Extraction Kit was purchased from Kunming YoungGen Biotechnology Co., Ltd. (DP101, Kunming, China). Fetal Bovine Serum (S660JJ) was obtained from Shanghai BasalMedia Technologies Co., Ltd, China. The stereotaxic apparatus and small animal anesthesia machine were obtained from Beijing Zhongshi Dichuang Technology Development Co., Ltd., while the Hi-FiGuard DNA Polymerase (MBR019020) was sourced from GENCEFE Biotech Co., Ltd., Wuxi, China. Recombinant Mouse GM-CSF (C-6His) was obtained from Novoprotein, Shanghai, China. Taq DNA Polymerase (Glycerol-Free) was purchased from Novoprotein (E001-03, Shanghai, China). L-7102 Protein Loading Buffer (5×) was purchased from Shanghai Biolinkedin biotechnology Co., Ltd (L-7102, Shanghai, China). L-2201 Protein A Plus Agarose gel was purchased from Shanghai Biolinkedin biotechnology Co., Ltd (L-2201, Shanghai, China).

The Mouse IL-17E ELISA kit (EMC00.48) was obtained from Neobioscience Technology Co., Ltd. The Interleukin 6 (IL-6) ELISA mouse kit (SU-BN20188) was sourced from Beijing Chengzhikewei Biotechnology Co., Ltd. D-Luciferin potassium salt (XY2091) and Cy5-NHS (XY2071) were acquired from Nanjing Starleaf Biological Technology Co. The GAPDH mouse monoclonal antibody (LF205S) was procured from Shanghai Epizyme Bio-Pharmaceutical Technology Co., Ltd. FITC-Goat Anti-Rabbit IgG (High Avidity) was obtained from Guangzhou Biolight Biotechnology Co., Ltd (GAB3101, Guangzhou, China). 1,2-Distearoyl-sn-glycero-3-phosphorylcholine (DSPC) was obtained from AVT Pharmaceutical Tech Co., Ltd. (S01005, Shanghai, China). High glucose medium (BC-M-005-500mL DMEM), 1640 medium (BC-1640M-005-500mL DMEM), 1% penicillin–streptomycin, fetal bovine serum (BC-SE-FBS01), and 0.25% trypsin-EDTA (BC-CE-005) were sourced from BioChannel Biological Technology Co., Ltd.

The Cell Counting Kit-8 (CCK-8) was obtained from ZUNYAN Biological Technology Co., Ltd (ZYCD002-0100, Nanjing, China). A 0.1 ml qPCR 96-well plate (AG12102) was obtained from Accurate Biotechnology (Hunan) Co., Ltd., Changsha, China. The 2X Accurate Taq Premix (with dye) was obtained from Accurate Biotechnology (Hunan) Co., Ltd. (AG11009, Changsha, China). Fetal Bovine Serum (JYK-FBS-301) was sourced from Jin Yuan Kang Biotechnology Co., Ltd., Neimenggu, China. The Albumin Content Assay Kit (Bromocresol Green Colorimetry) was obtained from Beijing Boxbio Science & Technology Co., Ltd (AKPR040C, Beijing, China). The Glutamic Pyruvic Transaminase (GPT) Activity Assay Kit (AKAM006C) was obtained from Beijing Boxbio Science & Technology Co., Ltd, Beijing, China.

### Cell culture

Raw 264.7 (SCSP-5036), SH-SY5Y (SCSP-5014), bEnd.3 (SCSP-5267), and Neuro-2a (SCSP-5035) cells were sourced from the Chinese Academy of Science Cell Bank (Shanghai, China). The mouse GL261 (catalog: SAc0135) cell lines were purchased from Shanghai Fusheng Industrial Co.; Human nasal epithelial cell line HNEpC (IM‑H279, IMMOCELL, Xiamen, Fujian, China); sex not specified. G422 (IM-M041) cells were purchased from IMMOCELL (Xiamen, Fujian, China). PC12 (QuiCell-P305) cells were purchased from QuiCell (Shanghai, China). Cells were purchased from Nanning Rising Biotechnology Co., Ltd. (Nanning, Guangxi, China). The cell lines were consistently treated with a mycoplasma-removing agent (VivaCell, Shanghai, China) and tested negative for mycoplasma contamination. The bEnd.3, GL261, GL261-Luc, Neuro-2a, and G422 cells were cultured in high-glucose DMEM supplemented with 10% special grade Fetal Bovine Serum. PC12, SH-SY5Y, and Raw 264.7 cells were cultured in RPMI-1640 medium supplemented with 10% special grade Fetal Bovine Serum. HNEpC cells were maintained in a specific medium for HNEpC and incubated at 37 °C in an atmosphere with 5% CO_2_. The cells were suspended in CELLSAVING serum-free cryopreservation solution (cat C40100, New Cell & Molecular Biotech, China). Fetal Bovine Serum (FBS-CS100) was obtained from Premium, NEWZERUM Ltd., Shanghai, China.

### Isolation and culture of primary T-cells from mice

Spleens from C57BL/6 mice were collected and immersed in sterile phosphate-buffered saline (PBS). The spleens were mechanically disrupted using a 70 µm cell strainer to obtain a single-cell suspension. 5 mL of RBC lysis buffer (eBioscience, San Diego, CA, USA) was added, incubated at room temperature for 5 min, and centrifuged at 500 g for 5 min. CD8⁺ T cells were isolated with the EasySep™ Mouse CD8⁺ T Cell Isolation Kit (catalog #19853, Stemcell Technologies, Vancouver, Canada). The isolated T cells were cultured in RPMI 1640 medium with 10% fetal bovine serum, 1% penicillin–streptomycin, 1% L-glutamine, and 20 IU/mL recombinant interleukin-2 (IL-2) (11848-HNAE2, Sino Biological Inc., Beijing, China). Fetal Bovine Serum was sourced from BaiDi Biotechnology Co., Ltd. (F800-050, Zhejiang, China). The serum-containing mouse T-lymphocyte culture medium was obtained from Eynos Lifescience Biotechnology Co., Ltd. (C400100, Nanjing, China). Cells were cultured at 37 °C in a humidified environment with 5% CO₂.

### Examination of the TCGA and GTEx databases

Using the TCGA and GTEx databases accessed through http://gepia2.cancer-pku.cn/, we compared the expression profiles of *T-cell exhaustion-related genes (HAVCR2, LAG3, PDCD1, CXCL13, LAYN, TIM3, CTLA4)* between normal brain tissues and tumor tissues in GBM patients. The study assessed the relationship between the expression of *T-cell exhaustion-related genes (HAVCR2, LAG3, PDCD1, CXCL13, LAYN, TIM3, CTLA4)* and the prognosis of GBM patients. Furthermore, the expression and prognostic relevance of *NE-related genes (ADRA1B, ADRA1D, ADRA2A, ADRA2B, ADRA2C, DBH, PNMT, SLC6A2)* in GBM patients were analyzed. Additionally, the correlation between the expression of *Treg cell-related genes (FOXP3, CTLA4, CCR8, TNFRSF9)* and patient prognosis in GBM was investigated. The study investigated the association between *key genes in the cAMP/PKA/CREB signaling pathway (CREB1, CREBBP, ATF1, ATF2)* and a gene set related to *T-cell exhaustion (HAVCR2, TIGIT, LAG3, PDCD1, CXCL13, LAYN)* in GBM patients.

### Human GBM tissue multiplexed immunofluorescence staining

We retrospectively included formalin-fixed paraffin-embedded tumor samples in tissue microarray (TMA) format. All tissues were utilized in compliance with the Life Science Ethics Review Board of Zhengzhou University, protocol number ZZUIRB2024-191, which sanctioned patient consent forms or, in specific instances, a waiver of consent. The study presented no extra therapeutic risks. All patient information was kept confidential.

Tumor tissues from GBM patients who were either subjected to or not subjected to carvedilol treatment were processed for multiplex fluorescence staining. Consecutive 4 μm sections of GBM tumor tissue were deparaffinized, rehydrated, and underwent antigen retrieval with EDTA buffer (96 °C, pH 8.0, 1 h). Endogenous peroxidases were inhibited using 1% hydrogen peroxide in methanol at room temperature for 30 min. To prevent non-specific binding, 0.3% BSA in TBST was used for blocking antigens. DAPI dihydrochloride (4’,6-Diamidino-2-phenylindole) was obtained from Shandong Sparkjade Biotechnology Co., Ltd (SJ-MD0076, Shandong, China).

Each section was stained using standardized multiplex immunofluorescence panels to profile exhausted T cells, incorporating markers for DAPI (blue), CD3 (red, CY3), CD8 (green, Alexa Fluor 488), and PD-1 (yellow, SpRed). Primary antibodies were incubated for 1 h at room temperature, followed by a 1-h incubation with secondary antibodies under the same conditions. Residual HRP activity between secondary antibody incubations was eliminated by treatment with a solution containing benzoic hydrazide (0.136 mg) and hydrogen peroxide (50 µL). DAPI (BD Biosystems) was used for nuclear staining. Two control/index TMAs were concurrently stained to verify reproducibility.

Stained slides were scanned with a Vectra Polaris microscope (PerkinElmer), and the images were analyzed using InForm software (v2. 4.8, PerkinElmer). Technical support for multicolor immunofluorescence was provided by Wuhan Boerfu Biotechnology Co., Ltd.

### WB

To assess NE’s impact on protein expression in T cells, primary murine T cells were incubated for 24 h with NE (10 µM) alone, NE (10 µM) combined with carvedilol (10 µM), or NE (10 µM) combined with KG501 (6.89 µM). The proteins evaluated included phospho-PKA (Thr197), CREB, phospho-CREB, LAG-3, Tim-3, and CTLA-4, sourced from Cell Signaling (Danvers, MA, USA) and Proteintech (Wuhan, China). Cells were lysed on ice for 30 min using a lysis buffer composed of 50 mM Tris/HCl (pH 7.6), 150 mM NaCl, 5 mM MgCl₂, and 0.1% NP-40, freshly supplemented with 1* cOmplete™ EDTA-free protease inhibitor cocktail (Sigma, 11873580001), 1 mM DTT, 1 mM benzamidine, and phosphatase inhibitor cocktails 2 and 3 (Sigma, P5726 and P0044). The supernatants from centrifuged lysates were stored at −80 °C.

Protein concentration in purified samples was measured using a BCA protein assay kit. BCA protein concentration Kit was purchased from Coolaber Science & Technology Co., Ltd. (SK-1070-250T, Beijing, China). For immunoblotting, 15–20 μg of protein per sample was separated using the NuPAGE electrophoresis system. Proteins were transferred to membranes and detected with Sparkjade ECL star (ED0025-A, Shandong Sparkjade Biotechnology Co., Ltd., China). ImageJ software (USA) was used for quantification.

### M-EVs

The Raw264.7 murine macrophage cell line (C57BL/6J) was obtained from the Shanghai Institute of Life Sciences, Chinese Academy of Sciences. Cells were suspended in CELLSAVING serum-free cryopreservation solution (catalog number C40100, New Cell & Molecular Biotech, China). Raw264.7 cells were cultured in high-glucose DMEM medium (BC-SE-FBS01, BioChannel Biological Technology Co., Ltd., Nanjing, China) with 10% fetal bovine serum (FBS) (3023A, Umedium, Hefei, China) at 37 °C in a 5% CO₂ incubator. After 24 h, the medium was replaced with a complete medium containing 10% FBS, and cells were cultured for another 48 h.

The culture supernatant underwent sequential centrifugation at 300 g for 15 min, 3000 *g* for 30 min, 11,000 *g* for 1 h, and 170,000 *g* for 60 min. The mixture was filtered through a 0.22 μm membrane to eliminate cells and large particles. M-EVs underwent two rounds of centrifugation at 170,000 *g* for 60 min to remove proteins, were resuspended in phosphate-buffered saline (PBS), and stored at −80 °C for later analysis. Transmission electron microscopy (HITACHI FE-2000, Tokyo, Japan) was used to observe the morphological characteristics of M-EVs. Mastersizer 3000 (Malvern Instruments, UK) was used to determine the average particle-size distribution of M-EVs. Western blot analysis was conducted to identify M-EV markers, including TSG101, CD9, Calnexin, Histone H3, cytochrome C, and ALIX, using antibodies from Abcam, UK, confirming the successful isolation of M-EVs.

### M-EVs membrane (M-EVM)

M-EVM was obtained from M-EVs. Harvested M-EVs were resuspended in ice-cold Tris–Mg²⁺ buffer (pH 7.4, 0.01 M Tris, 0.001 M MgCl₂) and extruded 11 times through a polycarbonate membrane with a membrane extruder (Avanti Polar Lipids, USA) to disrupt them. The M-EV homogenate was combined with sucrose to achieve a 0.25 M concentration and then centrifuged at 2000 × *g* for 10 min at 4 °C. The supernatant was collected, washed with ice-cold TM buffer containing 0.25 M sucrose, and pelleted via centrifugation. The protein content of the purified M-EVM was measured using a BCA protein assay kit (abs9323, Absin, Shanghai, China). Additionally, the macrophage membrane (MM) was prepared using the same method.

### Preparation and characterization of iMPC

Nanovesicles (MPC) loaded with aPD-L1 and carvedilol were prepared using M-EVM via the sequential freeze-thaw extrusion (SFTE) method^24,37^, which involved ultrasonication (1 min on, 1 min off on ice, 6 cycles), freeze-thaw cycles (3 cycles), and repeated extrusion (11 passes). The mass ratios of M-EVM to PASP were set at 10:1, 5:1, 3:1, 2:1, and 1:1, respectively. PASP was conjugated to M-EVM through a reaction with EDC/NHS to activate carboxyl groups, followed by a 4–6 h incubation at room temperature. The resulting PASP-M-EVM complex was purified by dialysis to remove unreacted PASP and reagents. The PASP-M-EVM complex, aPD-L1, and carvedilol underwent six rounds of ultrasonication on ice. Ultrasonication parameters were set as follows: output power 35%, trigger interval 5 s, duration 1 min, duty cycle 30%, frequency 20 kHz, on ice. The mixture was extruded using a membrane extruder (Avanti Polar Lipids, USA) to form MPCs, which were then extracted and purified. During extrusion, sodium cholate (1.5%), cholesterol (0.02%), leupeptin (0.2 μg/mL), and NaCl (500 mM) were added to the reaction system to improve flexibility and minimize membrane protein degradation. The MPCs were dissolved in a non-controlled rate cell cryopreservation medium (Cyagen Biosciences, China) to preserve protein functionality and then underwent freeze-thaw cycles to enhance loading efficiency.

To conjugate the iRGD peptide (QYAOBIO, China Peptides Co., Ltd., China), NHS-PEG4-MAL (R-1083-2k, Xi’an Rui Xi Biotechnology Co., Ltd., Xian, China) was added to the MPC solution at a mass ratio of 0.025 relative to M-EVM. This mixture was allowed to react with surface M-EVM proteins via click chemistry for 4 h. Following purification by dialysis, the iRGD peptide was introduced at a mass ratio of 0.02 relative to M-EVM, and the reaction proceeded for an additional 2 h to yield iMPC.

aPD-L1 and carvedilol were conjugated with Cy5 NHS ester (146368-14-1, Xi’an Rui Xi Biotechnology Co., Ltd., China). Post-purification, the encapsulation efficiency (EE) and drug loading capacity (DLC) of aPD-L1 and carvedilol within the nanovesicles were assessed using a fluorescence microplate reader (Thermo Fisher Scientific Inc., USA). The encapsulation efficiency (%) was calculated as: (amount of drug loaded/total amount of drug added) * 100%. Drug loading efficiency (%) = (amount of drug loaded/total amount of drug-loaded nanovesicles) * 100%.

The efficiency of aPD-L1 release from iMPC was compared by varying the mass ratio of M-EVM to PASP (100%, 10:1, 5:1, 3:1, 2:1, 1:1) in PBS buffer at pH 6.5. Following the identification of the optimal M-EVM/PASP ratio in the iMPC, the efficiency of aPD-L1 release was assessed in PBS buffer across varying pH levels (6.0, 6.5, and 7.0). aPD-L1 was conjugated with Cy5, and its fluorescence intensity was assessed using a PerkinElmer multifunctional microplate reader (Boston, MA, USA). The morphology and dispersion of iMPC were analyzed using transmission electron microscopy (TEM; HITACHI FE-2000, Tokyo, Japan) and atomic force microscopy (AFM; Dimension Icon, Bruker, Billerica, MA, USA; tip model ScanAsyst-Air). Hydration particle sizes were measured using dynamic light scattering with the BeNano 1800 Zeta Pro (Dandong Bettersize Instruments Ltd., China). The ex vivo stability of iMPC was assessed by monitoring alterations in average particle size and zeta potential over 7 days in DMEM, 10% FBS, and PBS buffer at pH 7.4.

### Cell vitality

Primary mouse CD8⁺ T cells (1 * 10⁶/mL) were cultured in 96-well plates under previously described conditions. Six treatment groups were established: control (conventional culture), NE (10 µM), NE (10 µM)+carvedilol (10 µM), NE (10 µM)+aPD-L1 (10 µg/mL), NE (10 µM)+aPD-L1 (10 µg/mL)+carvedilol (10 µM), and NE (10 µM)+iMPC (40 µg/mL). Cell viability was evaluated after 24 h using a CCK-8 kit. A 10% CCK-8 reagent was added to the cultured cells and incubated at 37 °C for 1 h. Absorbance was then measured at 450 nm using a PerkinElmer microplate reader (Boston, MA, USA). The CCK-8 kit was purchased from Bioligo Biotechnology Co., Ltd. (JDB0101-001, Shanghai, China). Cell viability (%) was determined using the formula: (OD experimental – OD blank)/(OD control – OD blank) * 100%. The control group comprised untreated normal cells, while the blank group included only basal medium with 10% CCK-8 reagent.

### In vitro NMB model

Human nasal epithelial cells (HNEpCs) were obtained from the Shanghai Institute of Life Sciences, part of the Chinese Academy of Sciences. HNEpCs and GL261 cells, each at a density of 1*10⁶ cells/mL, were seeded into a 24-well Transwell® migration insert (pore size 400 nm; Millipore, MA, USA), with 500 µL of HNEpCs in the upper chamber and 500 µL of GL261 cells in the lower chamber. Cells were detached with 0.25% trypsin-EDTA (BioChannel Biological Technology Co., Ltd., Cat# BC-CE-005) and cultured for 72 h. Then, 1 * 10⁵ primary mouse T cells were placed in the lower chamber. Six treatment conditions were applied: control group, aPD-L1 group, P&C group, MPC group, iMPC group, and iMPC+US group. The aPD-L1 concentration was kept at 10 µg/mL, with carvedilol administered at 10 µM. After 12 h, T cells were harvested via centrifugation at 500 g for 5 min and subsequently stained with antibodies against CD3, CD8, IFN-γ, TNF-α, PD1, and Tim3 (BioLegend, USA; Cat# 100204, 100732, 430807, 986802, 143705, 134011). Flow cytometry (Becton–Dickinson) was used to analyze the proportions of CD3⁺CD8⁺ T cells, including subsets expressing IFN-γ, TNF-α, and PD1⁺Tim3⁺.

Following six different treatments, immunofluorescence staining was performed on the NMB model. The NMB model underwent three washes with pre-warmed PBS, each lasting 10 min. Fixation was carried out using 4% formaldehyde at room temperature for 20–30 min. The samples underwent three 10-min washes with PBS. Cells were permeabilized using 0.2% Triton X-100 (56515; Affymetrix, USA) for 5 min, then washed three times with PBS for 10 min each. Blocking was conducted at room temperature for 30 min using 5% BSA. Primary antibodies against Claudin-1 (28674-1-AP) or Occludin (27260-1-AP) from Proteintech, Wuhan, China, were applied and incubated overnight at 4 °C. Following three 10-min PBS washes, fluorescent secondary antibodies (Anti-Mouse Alexa Fluor® 488, A11029, 1:1000; Anti-Rabbit Alexa Fluor® 488, A11034, 1:1000) were incubated in the dark at room temperature for 30 min. The samples were washed again three times with PBS for 10 min each. Samples were air-dried after nuclei staining with Hoechst for 5 min. Imaging was conducted with a Leica TCS SP confocal fluorescence microscope.

The NMB model established using HNEpCs underwent six treatment conditions: control group (routine culture), Free PC group, iMPC group, iMPC+US (0.5 w/cm²) group, iMPC+US (1.5 w/cm²) group, and iMPC+US (3.0 w/cm²) group. The ultrasound parameters included a duty cycle of 30%, a center frequency of 1 MHz, and a duration of 60 s. Two hours later, Western blot analysis was conducted to detect the levels of PKG (AB199438), RhoA (AB187027), Rock (AB45171), ZO-1 (AB307799), Claudin-1 (AB211737), Occludin (AB216327), and GAPDH (AB181602), all sourced from Abcam, UK.

Evaluate the effect of LIPUS alone on GL261 cell viability. HNEpCs (1 * 10⁶ cells/mL) were seeded in the upper chamber of a 24-well Transwell® migration insert (400 nm pore size; Millipore, MA, USA), and an equal volume of GL261 cells (IMMOCELL, Xiamen, China) at the same density was placed in the lower chamber. The experiment was divided into five groups: PBS group, P&C (aPD-L1+carvedilol) group, US (1.5 w/cm²) group, iMPC group, and iMPC+US group. The aPD-L1 concentration was kept at 10 µg/mL, with carvedilol administered at 10 µM. The ultrasound parameters included a 30% duty cycle, a center frequency of 1 MHz, and a duration of 60 s. GL261 cell viability was evaluated using the CCK-8 assay.

Seven treatment conditions were applied: control group, aPD-L1 group, P&C group, MPC group, iMPC group, MPC + US group and iMPC+US group. The aPD-L1 concentration was kept at 10 µg/mL, with carvedilol administered at 10 µM. Adherent GL261 cells were digested with 0.25% trypsin (BioChannel Biological Technology Co., Ltd., Nanjing, China), and digestion was halted using culture medium. All cell lines were confirmed free of mycoplasma contamination and regularly treated with a mycoplasma removal agent (VivaCell, Shanghai, China). An Annexin V-FITC/PI apoptosis detection kit (abs50001, Absin, Shanghai, China) facilitated the analysis. Caspase-3 (Abcam) was detected via immunofluorescence staining. The cell migration rate was determined using the formula: Cell Migration Rate (%) = [(Initial Scratch Area − Current Scratch Area)/Initial Scratch Area]*100%.

The NMB model derived from HNEpCs tracked trans endothelial electrical resistance (TEER) values over an 8-day period using a commercial ohmmeter (EVOM, WPI, USA). The TEER value for each well was calculated using the formula: TEER(cells) = (TEER(total) − TEER(blank))/culture area.

Cy5-labeled iMPC were placed in the upper chamber of the NMB model migration apparatus. The fluorescence signal intensity in the lower chamber was measured and statistically analyzed using an IVIS imaging system after 60 s of LIPUS exposure at varying acoustic intensities (0, 0.5, 1.5, 3.0, 4.0, and 5.0 w/cm²). TEER values were measured using a commercial ohmmeter (EVOM, WPI, USA).

To further evaluate the reversibility of NMB opening, the NMB model was treated with LIPUS at intensities of 1.5 or 3.0 w/cm². Immunofluorescence staining for Claudin-1 and Occludin proteins was conducted at intervals of 0, 0.5, 1.0, 2.0, 3.0, and 4.0 h post-treatment, using DAPI to counterstain the cell nuclei. The disruption and recovery of the NMB were examined using a Leica TCS SP laser scanning confocal microscope. TEER values were measured using a commercial ohmmeter (EVOM, WPI, USA).

Further evaluate the safety of iMPC nanoparticle formulations. HNEpC, bEnd.3, SH-SY5Y, PC12, and Neuro-2a cells were seeded into 96-well plates at a concentration of 1 × 10⁶ cells/mL. Experimental groups were as follows: HNEpC group, iMPC+HNEpC group, bEnd.3 group, iMPC+bEnd.3 group, SH-SY5Y group, iMPC+SH-SY5Y group, PC12 group, iMPC+PC12 group, Neuro-2a group, and iMPC+Neuro-2a group. The iMPC concentration was 35 µg/mL. Cell viability was evaluated using the CCK-8 assay following a 24-h conventional culture.

Evaluate the effect of β-blockers combined with aPD-L1 on T cell activity via flow cytometry. HNEpCs were seeded at a density of 1*10⁶ cells/mL in 500 µL into the upper chamber of a 24-well Transwell® migration insert (400 nm pore size; Millipore, MA, USA), while an equal volume and density of GL261 cells (IMMOCELL, Xiamen, China) were placed in the lower chamber. Lymphocytes were isolated from the spleens of tumor-free C57BL/6J mice using lymphocyte isolation medium (P9000, Solarbio, China).

Place the extracted lymphocytes in the lower chamber. The experiment comprised six groups: control group, NE group (NE placed in the lower chamber at 10 µM), NE + C (10 µM NE and 10 µM carvedilol placed in the lower chamber), NE + PC (10 µM NE, 10 µg/mL aPD-L1, and 10 µM carvedilol placed in the lower chamber), iMPC (35 µg/mL, placed in the upper chamber), and iMPC+US (35 µg/mL iMPC placed in the upper chamber, 1.5 w/cm²). Other US parameters were as follows: duty cycle 30%, center frequency 1 MHz, and duration 60 s. After 24 h, the cell suspension in the lower chamber was collected and cells were extracted.

Cells were stained for 90 min using antibodies against CD45, CD3, CD4, CD8a, TNF-α (catalog numbers: 103112, 100204, 100537, 100732, 506307; BioLegend, USA), and IFN-γ (catalog number: 505807; BioLegend, USA). The staining for anti-TNF-α and anti-IFN-γ was conducted following the BioLegend intracellular staining protocol (424401; BioLegend, USA). Flow cytometric analysis was performed on stained cells to identify populations of CD3⁺CD8⁺ and CD3⁺CD4⁺ T cells, including those expressing TNF-α and IFN-γ, with 20,000 events collected for analysis. Flow cytometric analysis antibodies were sourced from Dakewe Biotech Co., Ltd., while analytical grade chemicals were procured from Sigma-Aldrich (USA).

### In vitro tumor targeting

A 500 µL suspension of GL261 cells, with a concentration of 1*10⁶ cells/mL, was placed in the lower chamber of a Transwell migration insert (100 nm pore size; Millipore, MA, USA). Following 24 h of standard culture, four nanovesicle types (MM, M-EVM, MPC, and iMPC), each pre-labeled with Cy5 NHS ester (Cy5 NHS, 146368-14-1, Xi’an Rui Xi Biotechnology Co., Ltd., China) at a concentration of 10⁵/mL, were introduced to the upper chamber. After 12 h of incubation, the GL261 cells in the lower chamber were washed twice with PBS. The distribution of various nanovesicles on GL261 cell surfaces was imaged using a Leica TCS SP confocal laser scanning microscope. Fluorescence signals were quantitatively analyzed using ImageJ software (ImageJ Software Inc., USA).

To confirm that iMPCs can deliver drugs into the intercellular spaces of GL261 cells, GL261-GFP cells were suspended at a density of 1 × 10⁶ cells/mL and seeded into 48-well plates. After 24 h of routine culture, the experiment was divided into four groups: the aPD-L1 group (without GL261-GFP cells), the iMPC group (loaded with aPD-L1-Cy5, without GL261-GFP cells), aPD-L1-Cy5 group (without GL261-GFP cells), and iMPC+GL261-GFP group (loaded with aPD-L1-Cy5). The concentration of aPD-L1 was kept at 10 µg/mL. After 24 h of conventional culture, the four cell suspensions were removed and dialyzed (200 kDa, Acmec, Shanghai, China) for 12 h. The dialyzed fluid was collected and analyzed for fluorescence signals using IVIS imaging. Confocal microscopy was employed to examine the intercellular distribution of aPD-L1-Cy5 in the iMPC+GL261-GFP group.

### Pharmacokinetics of iMPC in vivo

The pharmacokinetic behavior of carvedilol, aPD-L1, and various nano-formulations was studied by administering multiple nano-formulations intranasally to healthy C57 mice (*n* = 3). The administered dosages were 30 µg of aPD-L1 and 4 µg of carvedilol per 20 g of body weight. Ultrasound parameters included a 1.5 w/cm² intensity, 30% duty cycle, 1 MHz center frequency, and a 60-s duration. Blood samples were collected at 0, 1, 2, 4, 8, 12, 24, 36, 48, and 72 h following intranasal administration. Serum was extracted and subjected to acid treatment. Carvedilol and aPD-L1 concentrations were measured using a Tecan Safire fluorescence microplate reader (Lyon, France).

### Intra-body distribution and clearance rates in major organs of iMPC

To investigate the in vivo distribution of aPD-L1, 6-8-week-old C57 mice (20–22 g) were selected for the experiment, with males and females randomly assigned. The experiment involved nasal administration and was divided into four groups: control, aPD-L1, iMPC, and iMPC+US. LIPUS targeted the olfactory epithelium within the nasal cavity. aPD-L1 was labeled with Cy5-NHS (Starleaf Biological Technology Co, Nanjing, China). The aPD-L1 dosage was administered at 30 µg per 20 g of body weight. IVIS imaging, conducted 6 h later, identified fluorescent signals in the heart, liver, spleen, lungs, kidneys, and brain across all four groups. Fluorescence signal values were statistically analyzed using ImageJ software (ImageJ Software Inc., USA).

To investigate the in vivo distribution of aPD-L1, 6-8-week-old C57 mice (20–22 g) were selected for the experiment, with males and females randomly assigned. The experiment involved nasal administration and was divided into four groups: control, aPD-L1, iMPC, and iMPC+US. LIPUS targeted the olfactory epithelium within the nasal cavity. aPD-L1 was labeled with Cy5-NHS (Starleaf Biological Technology Co, Nanjing, China). The aPD-L1 dosage was administered at 30 µg per 20 g of body weight. Six hours later, IVIS imaging revealed fluorescent signals in the heart, liver, spleen, lungs, kidneys, and brain across all four groups. Fluorescence signal values were statistically analyzed using ImageJ software (ImageJ Software Inc., USA).

The in vivo distribution of carvedilol was examined by categorizing the experiment into four groups: control, carvedilol, iMPC, and iMPC+US. Carvedilol was labeled with Cy5-NHS (Starleaf Biological Technology Co., Nanjing, China). Carvedilol was administered at a dosage of 4 µg per 20 g of body weight. Six hours after administration, IVIS imaging revealed fluorescence in the heart, liver, spleen, lungs, kidneys, and brain of mice in all four groups. Fluorescence signal values were statistically analyzed using ImageJ software (ImageJ Software Inc., USA).

Further evaluate the clearance rates of carvedilol and aPD-L1 in the lungs, kidneys, and liver. Select 6-8-week-old C57 mice (20-22 g) for the experiment, randomly assigning males and females, and administer the drugs intranasally. LIPUS was administered to the olfactory epithelium in the mouse nasal cavity. Experimental groups included: carvedilol group, aPD-L1 group, iMPC group, and iMPC+US group. aPD-L1 was administered at 30 µg per 20 g of body weight, while carvedilol was given at 4 µg per 20 g of body weight. Carvedilol and aPD-L1 were conjugated with Cy5-NHS (Starleaf Biological Technology Co, Nanjing, China). Ultrasound parameters were set to 1.5 w/cm² with a 30% duty cycle, a center frequency of 1 MHz, and a duration of 60 s. Lungs, kidneys, and livers were collected and homogenized from mice at 0, 1, 2, 6, 12, 24, 48, and 72 h post-treatment. Fluorescence signal intensity was measured using IVIS and quantitatively analyzed with a PerkinElmer multifunctional microplate reader (Boston, MA, USA).

### Nose-to-brain delivery efficiency

Mice bearing orthotopic GBM models were intranasally administered with six different treatments: PBS, Free PC (aPD-L1 & carvedilol), iMPC, iMPC+US (0.5 w/cm²), iMPC+US (1.5 w/cm²), and iMPC+US (3.0 w/cm²). In the iMPC+US group, nasal mucosa was first subjected to US treatment, followed by intranasal administration of the iMPC suspension. LIPUS was administered to the olfactory epithelium in the mouse nasal cavity. aPD-L1 was administered at 30 µg per 20 g of body weight, while carvedilol was given at 4 µg per 20 g of body weight. Other US parameters were as follows: duty cycle 30%, center frequency 1 MHz, and duration 60 s10,14,16. aPD-L1 was labeled with Cy5. The LIPUS device was provided by the National Key Laboratory of Intelligent Imaging and Interventional Medicine of China.

Fluorescence intensity in the mouse brain was monitored using IVIS (ABL X5, Tanon, China) at pre-treatment and at 1, 2, 6, 12, 24, 36, 48, and 72 h post-treatment across six different treatments. At 2 h post-treatment, the trigeminal nerves were dissected and analyzed under IVIS to evaluate fluorescence intensity. Brain tissues were collected at 6 and 12 h post-treatment, and fluorescence signal intensity was assessed using IVIS followed by statistical analysis. Brain tissues were collected 12 h after treatment, and frozen sections of the tumor area were prepared. Nuclei were stained with DAPI, and aPD-L1-Cy5 distribution was analyzed using a Leica TCS SP confocal fluorescence microscope.

Additionally, US parameters were methodically adjusted to evaluate the efficiency of LIPUS-facilitated nose-to-brain delivery of iMPC. The US parameters were varied as follows: intensity (0 to 4.0 w/cm²), duty cycle (0–50%), center frequency (0–4.0 MHz), and application duration (0–180 s). aPD-L1 was labeled with Cy5. At 12 h post-LIPUS-mediated nasal administration of iMPC, brain tumor tissues were harvested, homogenized, and subjected to fluorescence intensity measurement and statistical analysis using IVIS.

Comparison of the efficiency of drug delivery to the tumor region between LIPUS-mediated intranasal-to-brain delivery of free PC (aPD-L1 and carvedilol) and iMPC. C57BL/6 mice were allocated into four treatment groups: control, Free PC (aPD-L1 and carvedilol), PC + US (ultrasound-mediated intranasal delivery of aPD-L1 and carvedilol), and iMPC+US (ultrasound-mediated intranasal delivery of iMPC). The administered doses were 30 µg/20 g for aPD-L1 and 4 µg/20 g for carvedilol. The US parameters were configured with an intensity of 1.5 w/cm², a duty cycle of 30%, a center frequency of 1 MHz, and a duration of 60 s. Additionally, aPD-L1 was labeled with Cy5. Fluorescence signal intensity in the brain was measured using an IVIS imaging system at pre-administration and at 2, 6, 12, 24, 48, and 72 h post-administration. Twelve hours after administration, brain tissues were dissected, and frozen tumor region sections were prepared. Nuclei were stained with DAPI, and aPD-L1-Cy5 distribution in the brain tumor was examined using a Leica TCS SP confocal laser scanning microscope. Brain tumor tissues were homogenized, and aPD-L1-Cy5 concentrations were quantitatively measured using a multifunctional microplate reader (PerkinElmer, Boston, MA, USA).

iMPC nanovesicles with particle sizes of 50, 100, 150, 200, and 300 nm were constructed, and the average particle size distribution of iMPC was measured using a Malvern particle size analyzer. aPD-L1 was labeled with Cy5. In a mouse orthotopic GBM model, nasal administration was conducted, and brain fluorescence signal intensity was measured using IVIS at pre-administration and 2, 6, 12, 24, 48, and 72 h post-administration. Six hours post-administration, brain tissues were isolated, and fluorescence signal intensity in the brain tumor region was assessed using IVIS. Fluorescence signal analysis was conducted using ImageJ software (ImageJ Software Inc., USA). Brain tumor tissues were homogenized for quantitative analysis of aPD-L1-Cy5 using a multifunctional microplate reader (PerkinElmer, Boston, MA, USA).

Further evaluation of tumor targeting by iRGD-modified iMPCs in vivo. C57 mice (6–8 weeks old, 20–22 g) were randomly assigned by sex for intranasal administration in the experiment. The experiments comprised four groups: PBS control, Free PC, MPC + US, and iMPC+US. aPD-L1 was labeled with Cy5-NHS (Starleaf Biological Technology Co, Nanjing, China). The dosage of aPD-L1 was administered at 30 µg for every 20 g of body weight. LIPUS was directed at the olfactory epithelium within the mouse nasal cavity. Ultrasound parameters included a 1.5 w/cm² intensity, 30% duty cycle, 1 MHz center frequency, and a 60-s duration. The efficiency of nasal-to-brain drug delivery was evaluated using IVIS imaging at intervals of 0, 1, 2, 6, 12, 24, 48, and 72 h post-treatment for all groups. Fluorescence signals were statistically analyzed using ImageJ software (ImageJ Software Inc., USA). Six hours after treatment, frozen sections were prepared, stained with DAPI, and imaged using a Zeiss LSM 510 laser-scanning fluorescence confocal microscope to observe aPD-L1-Cy5 distribution.

### LIPUS-induced thermal injury to the nasal mucosa

Further evaluation of the thermal effect of LIPUS on NMB. HNEpCs were seeded at 1*10⁶ cells/mL in 500 µL into the upper chamber of a 24-well Transwell® migration insert (400 nm pore size; Millipore, MA, USA). After conventional culture for 5 days, LIPUS treatment was applied at intensities of 0, 0.5, 1.0, 1.5, 2.0, and 3.0 w/cm². The other US parameters were as follows: duty cycle 30%, center frequency 1 MHz, and duration 60 s. Subsequently, temperature changes in the NMB were assessed using an infrared thermal imager (TIS65, FLUKE, USA) and statistically analyzed.

The olfactory epithelium in the nasal cavity of 6–8-week-old C57 mice (20–22 g) underwent LIPUS treatment at ultrasound intensities of 0, 0.5, 1.0, 1.5, 2.0, 2.5, 3.0, and 4.0 w/cm², with male and female mice randomly assigned. The US parameters included a 30% duty cycle, 1 MHz center frequency, and 60-s duration. Temperature changes in the nasal cavity’s olfactory region were then measured with an infrared thermal imager (TIS65, FLUKE, USA) and analyzed statistically. Western blot analysis was conducted to assess the expression of HSP70, CD68, MMP-2, MMP-9, and Tubulin in the nasal mucosa of the olfactory epithelium in C57 mice following LIPUS treatment at varying ultrasound intensities, using antibodies from Abcam, UK. Statistical analysis of gray values was conducted using ImageJ software (ImageJ Software Inc., USA).

### Disruption of NMB in vivo

To further evaluate the disruption of the NMB, nasal mucosa tissues from mice subjected to the aforementioned six different treatments were sectioned and subjected to immunofluorescence staining. Nasal tissues were dissected, rinsed with PBS, and fixed in 4% paraformaldehyde at 4 °C for 30 min. Following three 5-min PBS washes, the fixed tissues were sectioned into 10 µm slices using a cryostat microtome (HM400R, Microm, Heidelberg, Germany). The sections were mounted onto glass slides, kept clean, and air-dried. After three additional 5-min PBS washes, cells were permeabilized with 0.1% Triton X-100 at room temperature for 10 min. The sections were washed three times with PBS, each for 5 min. Blocking was carried out with 5% bovine serum albumin (BSA) for 1 h.

Primary antibodies against Claudin-1 (Abcam 14998, 1:200), Occludin (Abcam 216327, 1:200), and ZO-1 (Thermo Fisher Scientific 33-9100, 1:500) were incubated overnight at 4 °C. After three additional 5-min PBS washes, appropriate fluorescent secondary antibodies were applied. Claudin-1, Occludin, and ZO-1 were detected using Goat anti-Rabbit IgG (Alexa Fluor® 594, Thermo Fisher Scientific A-11037, 1:500 dilution), Goat anti-Rabbit IgG (Cy3, Jackson Immuno Research 111-165-144, 1:500 dilution), and Goat anti-Mouse IgG (Alexa Fluor® 488, Thermo Fisher Scientific A-11001, 1:500 dilution), respectively. The sections were washed three times with PBS, each for 5 min. DAPI was used to counterstain nuclei for 10 min. The sections were mounted using an anti-fade medium (Servicebio, G1401) and analyzed with a Leica TCS SP confocal laser scanning microscope.

Transmission electron microscopy (HITACHI FE-2000, Tokyo, Japan) revealed changes in the perineural structure, while hematoxylin and eosin (HE) staining assessed inflammatory cell infiltration in the nasal mucosa. Western blot analysis was conducted to assess protein level changes of PKG, RhoA, Rock, ZO-1, Claudin-1, Occludin, and GAPDH in nasal mucosal tissues across six treatment conditions, using specific antibodies from Affinity and Proteintech. ImageJ software (ImageJ Software Inc., USA) was utilized for quantitative analysis. The Ultrasound intensity was varied (0, 0.5, 1.0, 1.5, 2.0, 2.5, and 3.0 w/cm²) and applied to the nasal mucosa of mice for 60 s. Nasal mucosal tissues were isolated for Western Blot analysis to assess protein expression changes in ZO-1, Claudin-1, Occludin, and Tubulin, using antibodies from Proteintech, Wuhan, China.

Nasal mucosal tissue was assessed using hematoxylin and eosin (H&E) staining. Mice from each group were euthanized, followed by careful dissection and a 5-min PBS rinse of the nasal structures. The tissues were then refixed in 4% paraformaldehyde (PFA) for 24 h, followed by paraffin sectioning. H&E-stained sections were used to observe inflammatory cell infiltration in the nasal mucosal region. Images were captured with a Nikon E800 fluorescence microscope (Nikon, El Segundo, CA, USA).

To evaluate the reversibility of US-induced NMB disruption, LIPUS was administered with a 1.5 w/cm² intensity, 30% duty cycle, 1 MHz frequency, and 60-s duration. Nasal mucosal tissues were harvested at 0, 5, 30, 60, 90, 120, 180, and 240 min after LIPUS application. Protein expression changes of ZO-1, Claudin-1, Occludin, and Tubulin were analyzed using western blot, with antibodies sourced from Proteintech, Wuhan, China.

Nasal mucosal tissues were collected at intervals of 0, 0.5, 1.0, 2.0, and 3.0 h following LIPUS application. Immunofluorescence staining was conducted to assess the structural integrity of the NMB by analyzing the distribution of ZO-1, Claudin-1, and Occludin proteins. DAPI was used to counterstain cell nuclei.

### Mouse models and MRI

The Lent-EF1a-P2A-luciferase-CMV-coGFP-P2A-Puro lentivirus and polybrene were supplied by Banma Biotechnology Co., Ltd. in Changsha, China. GL261 cells at 50% confluence were transfected with Lent-EF1a-P2A-luciferase-CMV-coGFP-P2A-Puro lentivirus (titer: 7 × 10^8^ TU/mL) using an MOI of 100 and 4 µg/mL polybrene. Stable expression of GFP and luciferase in the GL261-luc cell line was achieved by screening with 3 µg/mL puromycin. A microliter syringe (Hamilton, USA) was used to intracranially inject a mixture of 4 μL matrix gel/PBS with 1*10^5^ GL261 or GL261-luc cells at coordinates 0.5 mm anterior to the fontanel, 1.5 mm right, and 2 mm deep. Tumor growth was monitored using a 7.0 T small animal MR scanner (Bruker, Germany) with T2-weighted and fast spin-echo sequences (TR/TE: 2000/50 ms, matrix: 256×256, FOV: 20 × 20 mm, slice thickness: 1.0 mm). Glioma-in-situ volume on T2WI images was quantified using ImageJ (ImageJ Software Inc., USA), and the relative tumor volume was calculated as the ratio of glioma-in-situ volume to the whole brain volume.

### Chronic restraint stress (CRs)

C57BL6 GBM mice were randomly allocated into control and stress groups, wherein mice in the stress group were subjected to daily restraint stress by being placed in well-venticated plastic restrainers that limited movement without causing compression for 6 h per day, to minimize non-experimental stress, all handling procedures were performed with extreme care, and potentially stress-inducing procedures (including injections and blood collection) were conducted under mild isoflurane anesthesia.

### IVIS

Tumor load in GL261-luc-bearing mice was quantified using IVIS. Tumor-bearing mice received an injection of D-luciferin (150 mg/kg, Starleaf Biological Technology Co., China), followed by imaging with the IVIS system (ABL X5, Tanon, China). The data were analyzed using Living Image 2.5 software (Caliper Life Sciences, USA).

### Antitumor activity in orthotopic GBM models

A microliter syringe (Hamilton, USA) was used to intracranially inject a mixture of 4 μL matrix gel/PBS containing 1*10^5^ GL261 cells at coordinates 0.5 mm anterior to the fontanelle, 1.5 mm right, and 2 mm deep. Meisen Matrigel (MS0102ZY) from Zhejiang Meisen Zhiyuan Biotechnology Co., Ltd. Fetal bovine serum was sourced from Umedium (3023A, Hefei, China). Day 0 was designated as the day of inoculation. Seven days later, the mice were randomly assigned and administered drug suspensions via the nasal route. The treatment groups included free aPD-L1, free PC (aPD-L1 and carvedilol), free iMPC, PC + US, and iMPC+US (based on a dosage of 30 µg/20 g aPD-L1 and 4 µg/20 g carvedilol). Three administrations were performed on Days 10, 13, and 15. In the PC + US and iMPC+US groups, ultrasound treatment (1.5 w/cm² intensity, 1 MHz center frequency, 30% duty cycle, 60 s duration) was administered to the mice’s nasal mucosa at 120, 180, and 240 min before intranasal delivery of free PC or iMPC. Tumor growth was monitored using magnetic resonance imaging. Mouse survival rates and body weights were documented, and the Log-rank test was used to assess the statistical significance of extended survival. All tumors remained below the maximum volume of 100 mm³.

A dual luciferase reporter GBM mouse model was developed by intracranially injecting 4 μL of a Matrigel/PBS mixture containing 1*10⁵ GL261-luc cells using a microliter syringe (Hamilton, USA) at stereotaxic coordinates: 0.5 mm anterior to the bregma, 1.5 mm lateral to the right, and 2 mm deep. The GelNest™ matrix gel (211212) was sourced from NEST Biotechnology, Wuxi, China. Inoculation was marked as Day 0. Seven days after inoculation, mice received an intraperitoneal injection of D-luciferin suspension (150 mg/kg; Starleaf Biological Technology Co., China). Bioluminescence signals in the mouse brain were monitored using an IVIS imaging system at pre-injection and at 2, 4, 6, 8, 10, 12, and 14 min post-injection to identify the optimal detection time window. Additionally, six different interventions were performed following the same protocol. Bioluminescence images were acquired using an IVIS system (ABL X5, Tanon, China) on days 5, 10, 15, and 20 after GL261-Luc cell inoculation. The data were analyzed using Living Image 2.5 software (Caliper Life Sciences, USA).

Brain tissue samples were obtained and fixed in 4% paraformaldehyde, followed by sequential dehydration in a graded series of alcohol solutions. Glioma sections were prepared in paraffin following the manufacturer’s protocol. Immunohistochemical staining utilized PCNA (AC058, PCNA Mouse mAb, Abclonal) and Ki67 (A26755PM, Ki67 Rabbit PolymAb®, Abclonal) antibodies, with technical assistance from Nanjing Youmeng Biotechnology Co., Ltd. Tumor sections underwent immunofluorescence staining using a TUNEL assay kit (Beyotime Biotech, Shanghai, China). ImageJ software was utilized for image analysis.

To systematically assess the effectiveness of LIPUS-mediated nasal-to-brain delivery of iMPC for GBM suppression, a GBM mouse model was developed using the G422-Luc cell line and BALB/c mice (6–8 weeks old, 20–22 g), with random assignment of both male and female subjects. The treatment regimen was identical to the previous protocol. Tumor growth progression was monitored via IVIS imaging to evaluate antitumor efficacy. Concurrently, daily measurements of body weight changes and survival status were recorded for mice in different treatment groups. Five days post-treatment, a TUNEL assay kit (Beyotime Biotech, Shanghai, China) was employed for immunofluorescence staining of tumor sections. ImageJ software was utilized for image analysis.

### Transcriptome sequencing (RNA-Seq)

Shanghai Meiji Bio-pharm Technology Co., Ltd. conducted transcriptome sequencing and analysis. Total RNA extraction from each sample utilized the TRIzol® Reagent Kit (Invitrogen, USA). RNA quality was evaluated using a NanoDrop ND-2000 spectrophotometer. Shanghai Majorbio Bio-pharm Technology Co., Ltd. performed RNA purification, reverse transcription, library construction, and quality control in strict accordance with Illumina kit protocols (Illumina, San Diego, CA). Following quality assessment using Qubit 4.0, sequencing was performed on the Illumina NovaSeq X PLUS platform. All raw sequencing data generated in this study have been deposited in the Genome Sequence Archive for Human (GSA-Human,) database under accession code CRA031945.

Raw sequencing data frequently include adapter sequences, low-quality reads, high N rate sequences (indicating ambiguous bases), and excessively short sequences, all of which can negatively impact subsequent analyses. To enhance bioinformatic analysis accuracy, raw data underwent quality filtering to produce high-quality clean data, enabling dependable downstream processing. The filtering process involved: 1) eliminating adapter sequences and reads lacking insert fragments due to self-ligation; 2) trimming bases with a quality score below 20 at the 3′ end, discarding reads with an average quality score under 10, and retaining others; 3) excluding reads with an N rate over 10%; and 4) removing sequences shorter than 20 bp post adapter and quality trimming. All filtering procedures were performed using the fastp software (https://github.com/OpenGene/fastp).

The cleaned data, post-quality control, were aligned to the reference genome to produce mapped data for further analyses, including transcript assembly and expression quantification. The alignment results underwent quality assessment, which included evaluating sequencing saturation, gene coverage, and the distribution of reads across genomic regions and chromosomes.

Transcript abundance reflects gene expression levels, with higher abundance indicating higher expression. In RNA-Seq analysis, gene expression levels are quantified by the read counts mapped to genomic regions. The expression levels of genes and transcripts were quantified using RSEM software to facilitate subsequent identification of differentially expressed genes/transcripts across samples and to further untangle gene regulatory mechanisms in conjunction with functional annotations of sequences.

Differential gene expression analysis was conducted on gene read counts from multiple samples (≥2) to identify differentially expressed genes (DEGs) and explore their functions. Differential expression analysis was performed using DESeq2 for datasets with biological replicates and DEGseq for those without, both under default parameters. Genes were deemed significantly differentially expressed if they satisfied both criteria: an FDR of less than 0.05 and an absolute log₂ fold change of at least 1.

### Functional enrichment analysis

Enrichment analysis aims to determine whether a set of genes is significantly enriched in a particular biological function node. The underlying principle involves extending the analysis from single-gene annotation to gene-set annotation. This approach enhances the reliability of research and helps identify the biological processes most relevant to a given biological phenomenon.

GO functional enrichment analysis was conducted using Goatools software, applying Fisher’s exact test for statistical evaluation. To manage the false positive rate, *p*-values were adjusted using four multiple testing correction methods: Bonferroni, Holm, Sidak, and False Discovery Rate (FDR). A GO term was deemed significantly enriched if the adjusted *p*-value (*p*-FDR) was ≤0.05.

Pathway enrichment analysis was conducted using KOBAS for Reactome pathways. This analysis uses a computational approach akin to GO functional enrichment analysis, utilizing Fisher’s exact test for statistical calculations. The Benjamini–Hochberg method was used to adjust for multiple testing, ensuring control of the false positive rate. Reactome pathways with a corrected *p*-value below 0.05 were deemed significantly enriched among differentially expressed genes.

Immune infiltration analysis was conducted using the QUANTISEQ tool. xCell is an ssGSEA-based algorithm that uses established gene signatures to estimate immune cell infiltration levels from RNA expression data of tissue samples. xCell analysis further untangled the detailed patterns of immune cell infiltration.

### TME

Tumor tissue levels of TNF-α, IFN-γ, IL-10, IL-17, and IL-15 were measured using specific ELISA kits. The TNF-α ELISA mouse kit (JM-02415M1) and the IL-15 ELISA mouse kit (YM-2908A1) were sourced from Nanjing, China, specifically from Jiangsu Jingmei Biological Technology Co., Ltd. and Nanjing Youmeng Biotechnology Co., Ltd., respectively. The IFN-γ ELISA mouse kit (JL10967) and the IL-10 ELISA mouse kit (JL20242) were obtained from Jianglai Biology Co., Ltd. in Shanghai, China. Additionally, the Caspase-3 ELISA mouse kit (F10011) was acquired from Shanghai Westang Biotechnology Co., Ltd. Tumor tissues were isolated, homogenized, and centrifuged for analysis. The supernatant was divided into aliquots and diluted using ELISA assay buffer as per the manufacturer’s guidelines. The GL261 Brain Cancer Mouse Tumor Antigen-Specific T Cell Assay Stimulation Kit (abs57014) was obtained from Absin Bioscience Inc, Shanghai, China.

Tumor tissue cell suspensions for T cell analysis were isolated using 0.5 μg/mL collagenase D (Roche, Switzerland), 0.5 μg/mL DNase I (Vazyme Biotec, China), and 3 μg/mL DNase I (Sigma-Aldrich, USA). Brain-infiltrating immune cells were then separated using lymphocyte isolation solution (Solarbio, China). Cells were stained for 90 min using the Zombie NIR™ Fixable Viability Kit and antibodies against CD3, CD4, CD8a, PD1, IFN-γ, CD62L, CD44 (catalog numbers: 423106, 100204, 100537, 100732, 143705, 430807, 104407, 103029; BioLegend, USA), and Foxp3 (126403; BioLegend, USA). Foxp3 staining followed the BioLegend intracellular staining protocol (424401; BioLegend, USA). Flow cytometric analysis was performed on stained cells to identify various T cell populations, including CD3⁺CD8⁺, PD1⁺CD3⁺CD8⁺, CD3⁺IFN-γ⁺, CD3⁺CD4⁺, Foxp3⁺CD3⁺CD4⁺, CD44⁺CD62L⁺, and CD44⁺CD62L⁻, with 20,000 events collected for analysis. Flow cytometric analysis antibodies were sourced from Dakewe Biotech Co., Ltd., while analytical grade chemicals were procured from Sigma-Aldrich (USA).

Assess the status of DCs and T cells in lymph nodes via flow cytometry. 5 days post-treatment, deep cervical and axillary lymph node tissues were harvested from GBM mice. Cell suspensions were isolated using 0.5 μg/mL collagenase D (Roche, Switzerland), 0.5 μg/mL DNase I (Vazyme Biotec, China), and 3 μg/mL DNase I (Sigma-Aldrich, USA). Cells were stained for 90 min using the Zombie NIR™ Fixable Viability Kit and antibodies against CD3, CD4, CD8a, CD11c, CD80, CD86, IFN-γ, TNF-α (BioLegend, USA; catalog numbers: 100204, 100537, 100732, 117305, 104714, 105026, 505808, 506307), and PD1 (143705, BioLegend, USA). Antibody staining for anti-IFN-γ, anti-TNF-α, and anti-PD1 was conducted following BioLegend’s intracellular staining protocol (424401; BioLegend, USA). Stained cells underwent flow cytometric analysis to identify various cell types, including CD3⁺ T cells, PD1⁺CD3⁺CD8⁺ T cells, Tim3⁺CD3⁺CD8⁺ T cells, TNF-α⁺CD4⁺ T cells, IFN-γ⁺CD4⁺ T cells, CD11c⁺ dendritic cells (DCs), CD86⁺CD11c⁺ DCs and CD80⁺CD11c⁺ DCs, with 20,000 events collected for analysis. Flow cytometric analysis antibodies were sourced from Dakewe Biotech Co., Ltd., while analytical grade chemicals were procured from Sigma-Aldrich (USA).

Tumor tissue sections underwent multiplex immunofluorescence staining. Quadruple staining with CD3, CD8, IFN-γ, and DAPI was employed to assess CD3⁺CD8⁺ T cell activity and infiltration. Triple staining (CD8, PD1, and DAPI for nuclear labeling) was applied to assess T cell exhaustion. Quadruple staining (CD3, CD4, Foxp3, and DAPI for nuclear labeling) was employed to evaluate Treg cell infiltration. Another quadruple staining (CD3, CD8, CD44, and DAPI for nuclear labeling) was conducted to assess memory T cell infiltration. Technical support for multicolor immunofluorescence was provided by Wuhan Ruisaiqi Biotechnology Co., Ltd.

In the tumor challenge study, C57BL/6 mice received an intracranial injection of 4 μL Matrigel/PBS mixed with 1*10⁵ GL261 cells using a 10 μL Hamilton syringe. The injection site was located 0.5 mm anterior to the bregma, 1.5 mm lateral to the right, and 2 mm deep. Subsequently, the mice underwent iMPC+US treatment. As a control, a GBM model group was established without treatment. Tumor progression was tracked using MRI up to day 50, and survival duration was documented. Survival prolongation was statistically analyzed using the Log-rank test.

### Safety evaluation

Post-experiment assays were conducted to assess the safety effects of different treatments on C57BL/6J mice. Histological changes in the heart, liver, spleen, lungs, and kidneys were examined using hematoxylin–eosin (HE) staining to evaluate the toxic effects on these major organs. Second, blood biochemical analysis targeted key hepatic and renal function indicators: alanine aminotransferase (ALT), aspartate aminotransferase (AST), total bilirubin (TBIL), serum creatinine (Crea), urea, and uric acid. Routine blood tests, including RBC, WBC, and PLT counts, along with leukocyte subset analysis, were conducted to assess the treatments’ effects on immune function and the hematopoietic system.

### Statistics and reproducibility

All statistical analyses were performed by SPSS version 19.0 (SPSS Inc., USA) and GraphPad Software (GraphPad Prism 9.3.0 for MacOS; GraphPad Software; GraphPad, Bethesda, MD). Statistical evaluation was performed with the Student’s *t* test and one-way analysis of variance (ANOVA). Survival was analyzed using log-rank tests. All values were described as the means ± standard deviation (SD), and differences were considered as statistically significant at *P* < 0.05. All experiments were repeated for at least three times and experimental findings were reproducible.

### Reporting summary

Further information on research design is available in the Nature Portfolio Reporting Summary linked to this article.

## Supplementary information


Supplementary Information
Reporting Summary
Transparent Peer Review file


## Source data


Source Data 1
Source Data 2


## Data Availability

The data that support the findings of this study are available in the supplementary material of this article. Source data are provided with this paper. All raw sequencing data generated in this study have been deposited in the Genome Sequence Archive for Human (GSA-Human,) database under accession code CRA031945. Source data are provided with this paper.
