# Supplementary material for: Low-intensity pulsed ultrasound-mediated nose-to-brain co-delivery of β-blockers and aPD-L1 enhances glioblastoma immunotherapy
Source: Nat Commun. 2026 Jul 30;17:9254. doi: 10.1038/s41467-026-76103-4 (PMC13526793; doi:10.1038/s41467-026-76103-4)
Supplement: Supplementary file 1 [file 41467_2026_76103_MOESM1_ESM.pdf]

Supplementary Information for

Low-intensity pulsed ultrasound-mediated nose-to-brain co-delivery of β-blockers and aPD-L1 enhances glioblastoma immunotherapy

Lei Dong[1, 2#], Zhengcheng Yun[2#], Lin Gao[2], Yue Li[3], Ying Zhou[4], Yini Zhu[4], Meng Li[5], Leqian Ying[1], Xuhong Yang[2], Jiangtao Yue[2], Xueqing Yong[6], Wanqing Cheng[2], Jia Miao[2], Nuo Xu[2], Xinyu Zhang[2], Hui Yang[7], Tingting Liu[8], Gaolin Liang[9], Shenghong Ju[2]*, Haijun Zhang[1]*, and Jinbing Xie[2]*

[1]Department of Oncology, Zhongda Hospital, Medical School, Southeast University, 87 Dingjiaqiao, Nanjing 210009, China.

[2]Nurturing Center of Jiangsu Province for State Laboratory of AI Imaging & Interventional Radiology, Department of Radiology, Zhongda Hospital, Medical School of Southeast University, Nanjing, 210009, China.

[3]State Key Laboratory of Mechanism and Quality of Chinese Medicine, Institute of Chinese Medical Sciences, University of Macau, Macau SAR, 999078.

[4]Department of Microbiology and Immunology, Medical School of Southeast University, Nanjing, 210009, Jiangsu, China.

[5]Nursing Department, The Third People's Hospital of Henan Province, School of Nursing, Zhengzhou University, Zhengzhou, Henan, 450000, China.

[6]Nanjing Institute of Measurement and Testing Technology, Nanjing 210049, China.

[7]Department of Biochemistry and Molecular Biology, Medical School of Southeast University, Nanjing, China.

[8]Zhongda Hospital, State Key Laboratory of Digital Medical Engineering, School of Public Health, Advanced Institute for Life and Health, Southeast University, Nanjing 210096, China.

[9]State Key Laboratory of Bioelectronics, School of Biological Science and Medical Engineering, Southeast University, 2 Sipailou Road, Nanjing, 210096, China.

#These authors contributed equally: Lei Dong and Zhengcheng Yun.

*Correspondence to: xiejb@seu.edu.cn (J. X.); haijunzhang@seu.edu.cn (H. Z.); jsh@seu.edu.cn (S. J.).

The PDF file includes:

Supplementary Figures 1 to 41

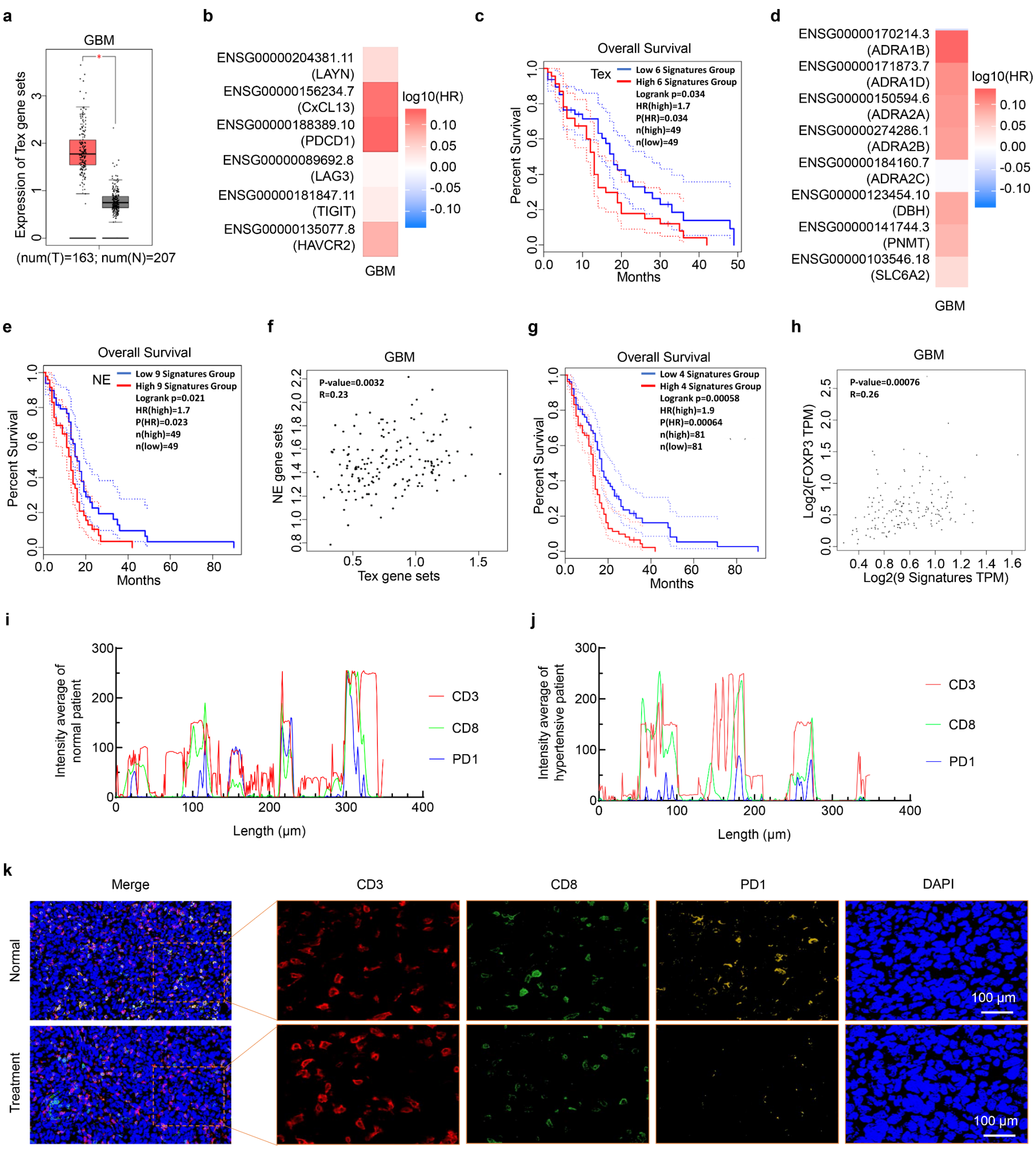

Supplementary Figure 1. Correlation analysis of prognosis of glioblastoma (GBM)

patients with T cell exhaustion and norepinephrine (NE) levels. The relationship between prognosis and T-cell exhaustion in GBM patients was analyzed using TCGA and GTEx databases through http://gepia2.cancer-pku.cn/. **a**, Compared to healthy individuals, the *T-cell exhaustion gene set (HAVCR2, LAG3, PDCD1, CXCL13, LAYN, TIM3, CTLA4)* is significantly overexpressed in GBM patients (T: GBM patients. N: healthy individuals). **b,** Expression of *T-cell exhaustion gene set (HAVCR2, TIGIT, LAG3, PDCD1, CXCL13, LAYN)* in tumor tissues of GBM patients. **c**, The high expression of the *T-cell exhaustion gene set (HAVCR2, TIGIT, LAG3, PDCD1, CXCL13, LAYN)* in GBM patients suggests a high prognostic risk. In GBM patients, higher expression of this gene set is associated with poorer patient prognosis, and the difference is statistically significant. **d-e**, Expression of the *norepinephrine-related gene set (ADRA1B, ADRA1D, ADRA2A, ADRA2B, ADRA2C, DBH, PNMT, SLC6A2)* in tumor tissues of GBM patients. In GBM patients, higher expression of this gene set is associated with poorer patient prognosis, and the difference is statistically significant. **f**, In GBM patients, there is a positive correlation between the expression of the *norepinephrine-related gene set (SLC6A2, PNMT, ADRA2C, ADRA2B, ADRA2A, ADRA1B, and ADRA1A)* and the *T-cell exhaustion gene set (HAVCR2, TIGIT, LAG3, PDCD1, CXCL13, LAYN)*. **g**, The expression of the *Treg cell exhaustion gene set (FOXP3-CTLA4-CCR8-TNFRSF9)* is negatively correlated with the prognosis of GBM patients, and this difference is statistically significant. **h**, *The key Treg cell gene FOXP3* is positively correlated with the expression of the *norepinephrine-related gene set (ADRA1B, ADRA1D, ADRA2A, ADRA2B, ADRA2C, DBH, PNMT, SLC6A2)*. **i-k**, Immunofluorescence triple staining was performed on tumor tissues from GBM patients who did (j) and did not (i) receive norepinephrine inhibitors (blue: DAPI; green: Alexa fluor 488-CD8; red: CY3-CD3; yellow: SpRed-PD1). Fluorescence co-localization analysis by ImageJ software. Source data are provided as a Source Data file.

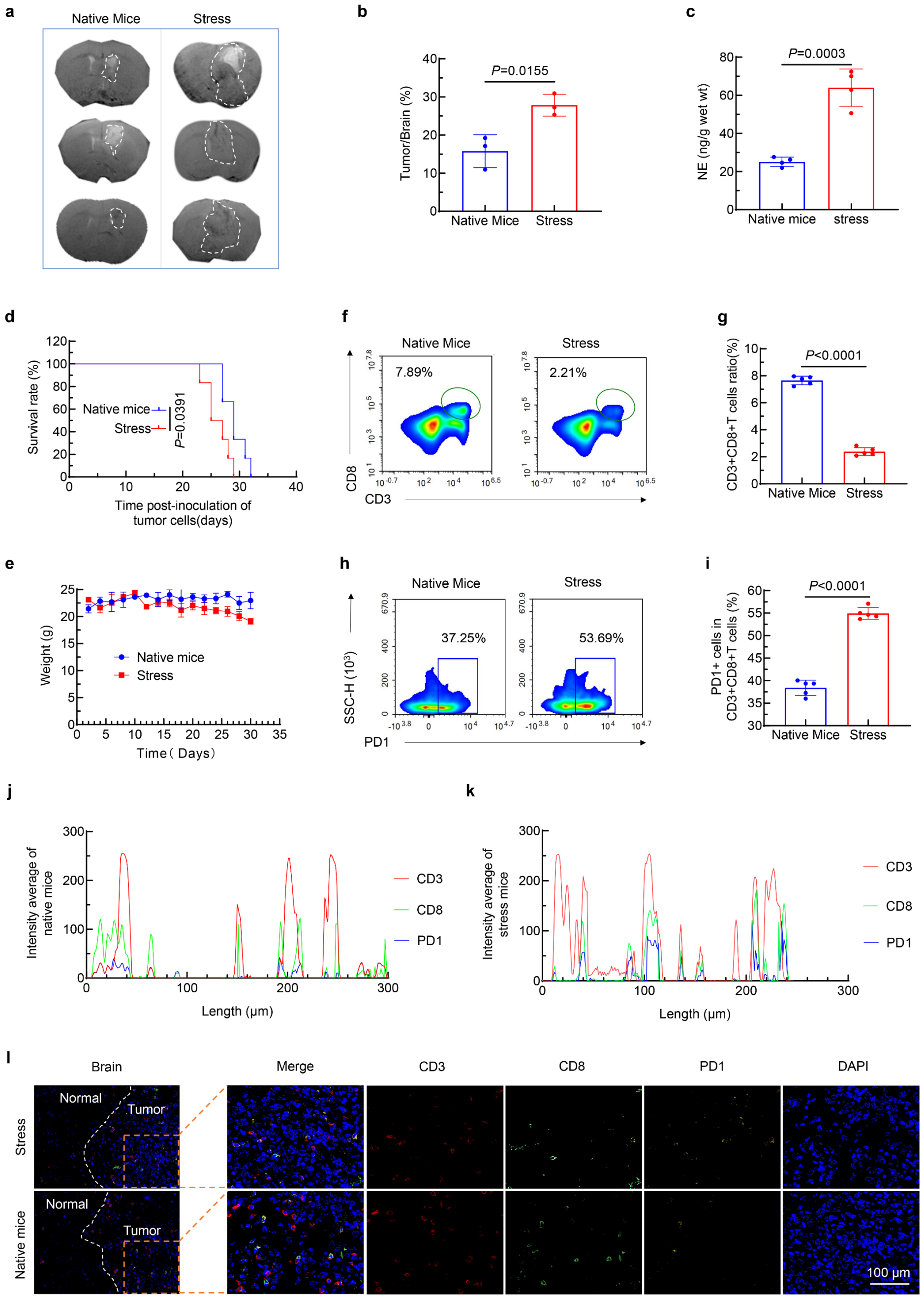

Supplementary Figure 2. Levels of norepinephrine (NE) and T-cell exhaustion in a glioblastoma (GBM) stress model of mice. a-b, T2 MRI images were used to evaluate the tumor size in GBM mice. **c**, An ELISA assay was used to detect norepinephrine content in the tumor tissues of GBM model mice and GBM stress model mice. **d-e**, Statistical analysis of survival duration and body weight changes in GBM model mice and GBM stress model mice (n = 10). Survival analysis was compared using the log-rank test. . **f-i**, Flow cytometry detection of the proportion of $CD3^{+}CD8^{+}$

T cells and the expression of PD1 in $CD3^+CD8^+$ T cells in tumor tissues of GBM model mice and GBM stress model mice. **j-l**, Triple immunofluorescence staining of tumor tissues from stressed and non-stressed GBM mouse models (blue: DAPI; green: Alexa fluor 488-CD8; red: CY3-CD3; yellow: SpRed-PD1). Fluorescence co-localization analysis by ImageJ software. n = 3 samples per group in **a-c**. Data are expressed as the mean ± SD. n = 3 samples per group in **a-c**. Data are expressed as the mean ± SD. n = 5 samples per group in **f-g and h-i**. Data are expressed as the mean ± SD. statistical analysis between two groups was performed using unpaired two-tailed Student's t-test. Comparisons were performed between different groups in **b-c, f-g and h-i**. Significant P values are shown. Source data are provided as a Source Data file.

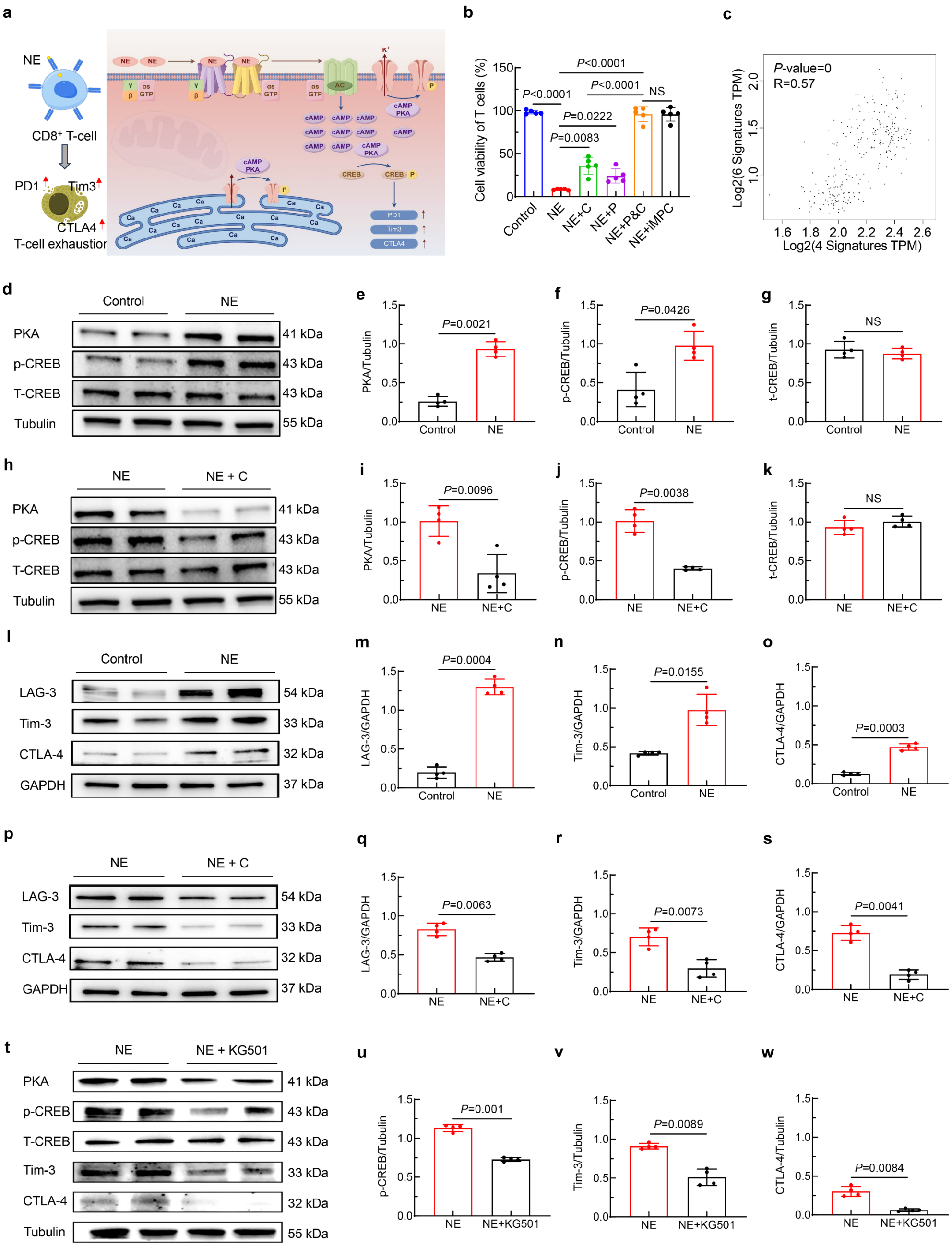

Supplementary Figure 3. β-blocker reduces norepinephrine (NE)-induced T-cell exhaustion. Panel a was created by Figdraw. **a**, Schematic diagram showing how carvedilol blocks norepinephrine-induced T-cell exhaustion (by figdraw.com). **b**, Co-culture of T cells and GL261 cells, with T cell viability assessed by CCK-8 after different treatments. **c**, Analysis of the TCGA database reveals a positive correlation between *key genes of the cAMP/PKA/CREB signaling pathway (CREB1-CREBBP-ATF1-ATF2)* and *T-cell exhaustion-related gene sets (HAVCR2-TIGIT-LAG3-PDCD1-CXCL13-LAYN)* in GBM patients, with p<0.0001 and R=0.57. **d-g**, Western blot (WB)

confirms that norepinephrine activates the cAMP/PKA/CREB signaling pathway. **h-k**, WB verifies that carvedilol can block the norepinephrine-activated cAMP/PKA/CREB signaling pathway. **l-o**, WB demonstrates that norepinephrine can activate the cAMP/PKA/CREB signaling pathway, leading to T-cell exhaustion. **p-s**, Carvedilol blocks norepinephrine-induced T-cell exhaustion. **t-w**, Specific inhibitor (KG501) of the cAMP/PKA/CREB signaling pathway can block norepinephrine-induced T-cell exhaustion. n = 5 samples per group in **b**. Data are expressed as the mean ± SD. Statistical significances were determined using one-way ANOVA with Fisher's LSD post-hoc test. n = 4 samples per group in **d-w**. Data are expressed as the mean ± SD. statistical analysis between two groups was performed using unpaired two-tailed Student's t-test. Comparisons were performed between different groups in b. Not significant (NS) is $P \geq 0.05$, and significant P values are shown. Source data are provided as a Source Data file.

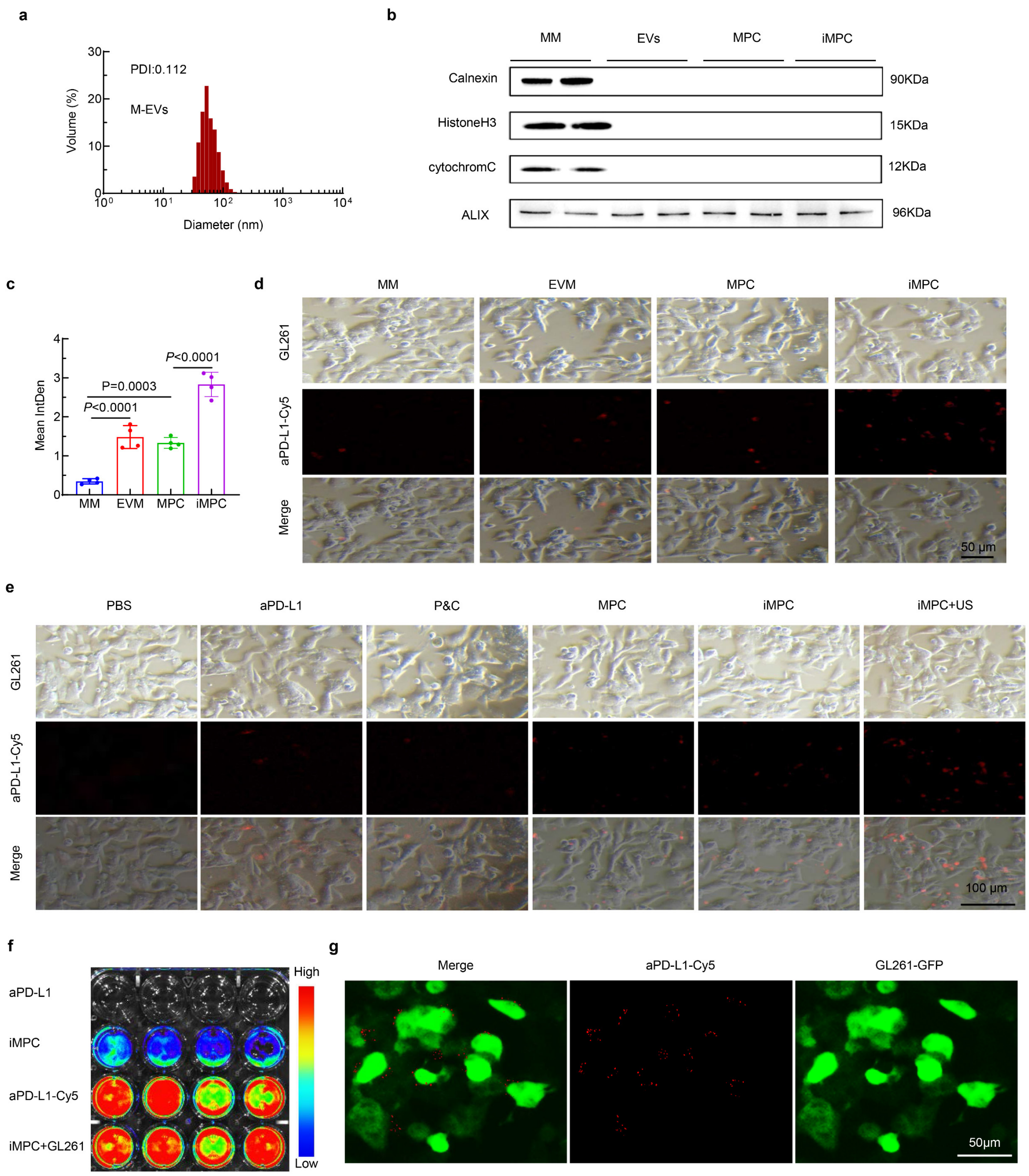

Supplementary Figure 4. Evaluation of the inhibitory effect on GL261 cells *in vitro*. **a,** Particle size distribution of macrophage extracellular vesicles (M-EVs). **b,** Expression levels of calnexin, histone H3, cytochrome C, and ALIX in macrophage membranes (MM), M-EVs, M-EVs loaded with aPD-L1 and carvedilol (MPC), and iRGD-modified MPC (iMPC). **c-d**, Comparison of the tropism towards GL264.7 cells between RAW264.7 cell membranes (MM), extracellular vesicle membranes (EM), biomimetic nanovesicles loaded with aPD-L1 and carvedilol (MPC), and peptide-modified biomimetic nanovesicles loaded with aPD-L1 and carvedilol (iMPC). **e**, Microscopic observation of the distribution of aPD-L1-Cy5 after different treatments

in the upper chamber of the Transwell. **f,** Extracellular aPD-L1-Cy5 fluorescence signal in GL261 cells. **g,** iMPCs release aPD-L1-Cy5 into the intercellular space of GL261-GFP cells. n = 4 biologically independent mice per group for data in **c**. Data are expressed as the mean ± SD. Statistical significances were determined using one-way ANOVA with Fisher's LSD post-hoc test. Comparisons were performed between different groups in **c**. Significant *P* values are shown. Source data are provided as a Source Data file.

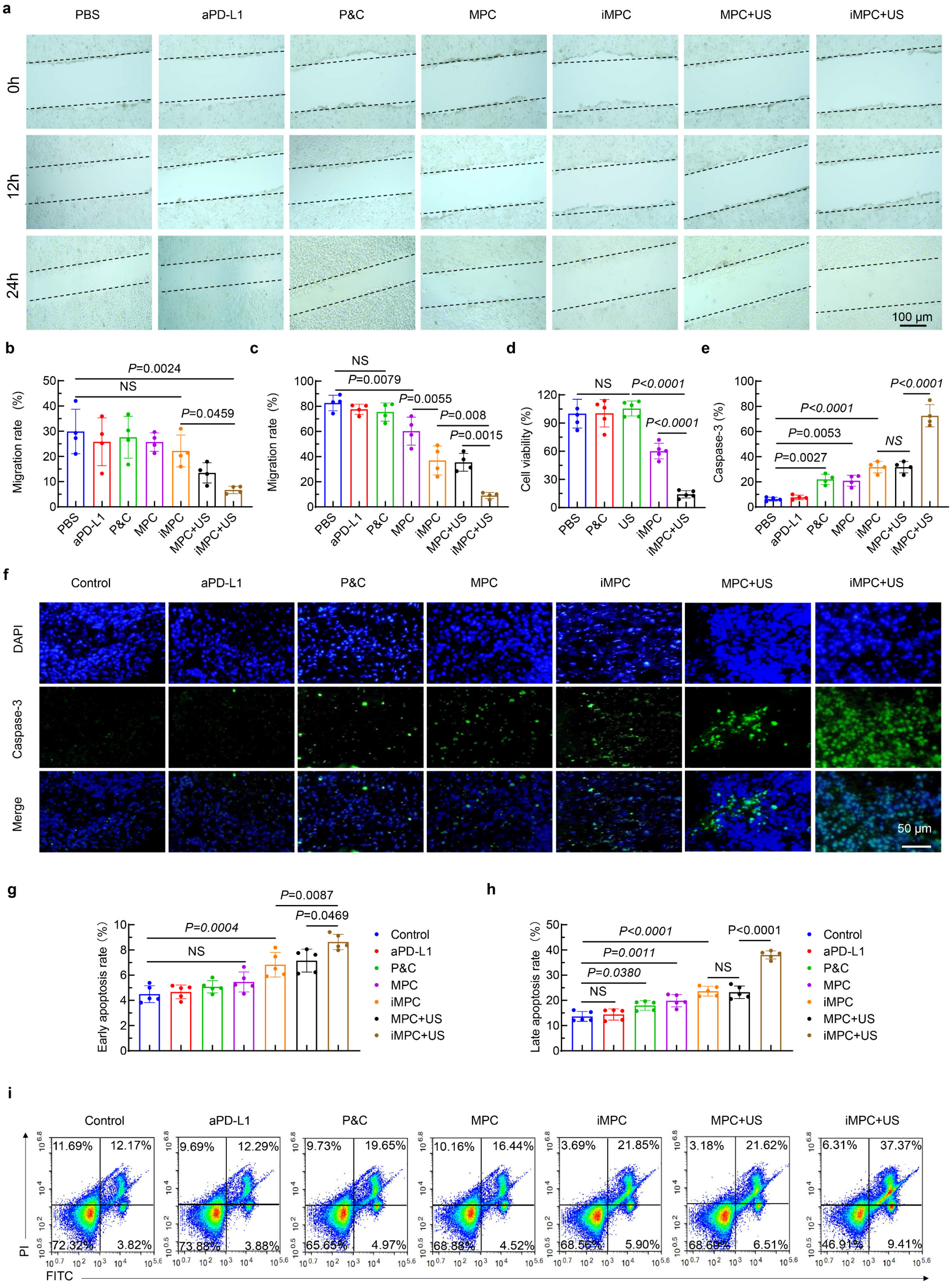

Supplementary Figure 5. Low-intensity pulsed ultrasound (LIPUS)-triggered nasal mucosal barrier opening combined with iMPC inhibits GL261 cell growth *in vitro*. a-c, Evaluation of the invasiveness of GL261 cells using a scratch assay after different treatments in the upper chamber of the Transwell. **d,** Effect of LIPUS on GL261 cell viability. **e-f,** Analysis of apoptosis in GL261 cells using the caspase-3 apoptosis fluorescence assay. **g-i**, The early apoptosis rate, late apoptosis rate, and total

apoptosis rate of GL261 cells after 24 hours of different treatments. n = 4 biologically independent mice per group for data in **b, c,** and **e**. n = 5 biologically independent mice per group for data in **d** and **g-h**. Data are expressed as the mean ± SD. Statistical significances were determined using one-way ANOVA with Fisher's LSD post-hoc test. Comparisons were performed between different groups in **b-e** and **g-h**. Not significant (NS) is $P \geq 0.05$, and significant P values are shown. Source data are provided as a Source Data file.

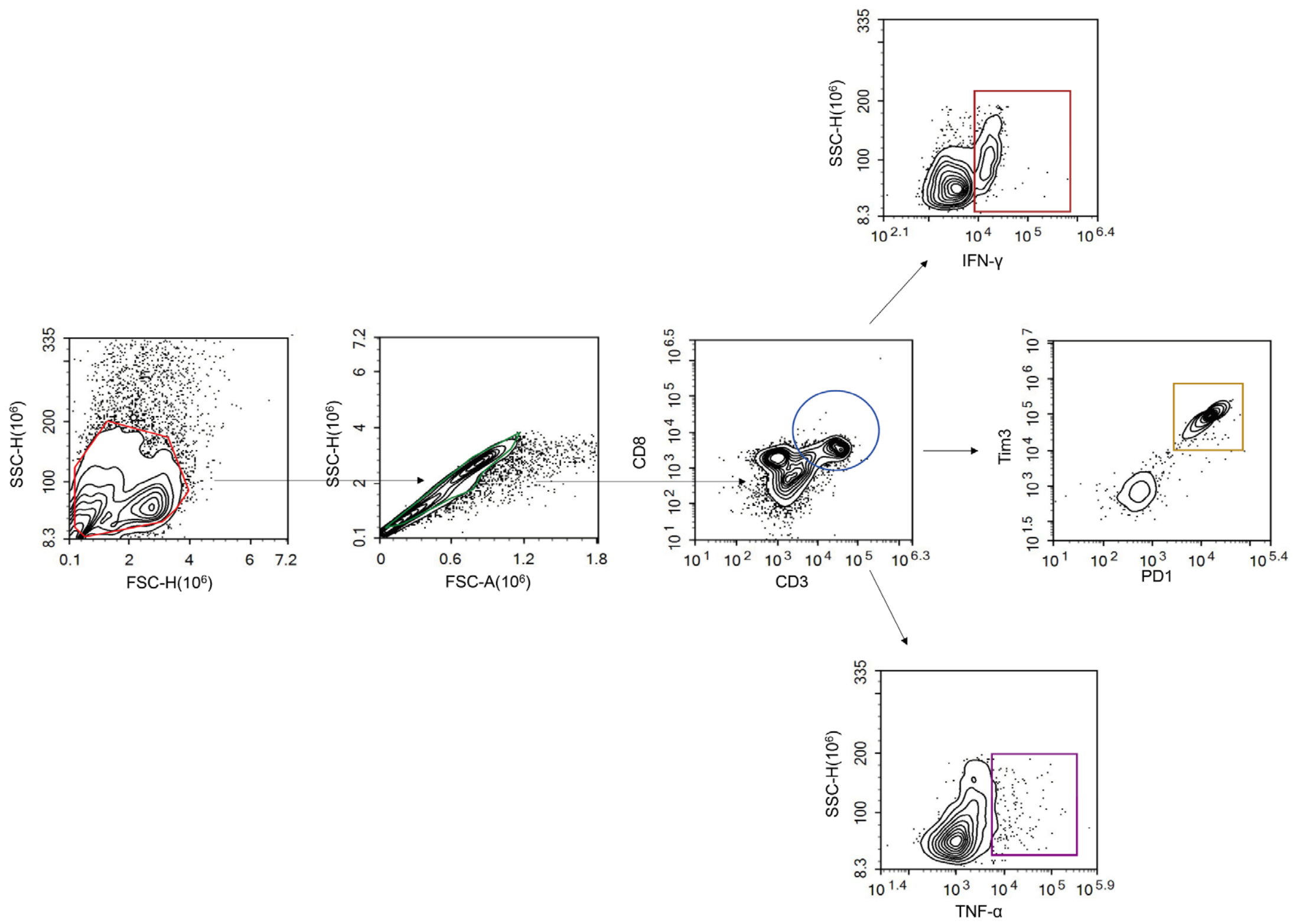

Supplementary Figure 6. Flow cytometry logical gating for CD3$^+$CD8$^+$ T cells and CD3$^+$CD8$^+$ T cells expressing IFN-γ, TNF-α, PD1, and Tim3. First, gating strategy for CD3$^+$CD8$^+$ T cells were isolated from the total cells. Subsequently, gating strategy for the CD3$^+$CD8$^+$ T cells expressing high levels of IFN-γ, TNF-α, PD-1 and TIM-3.

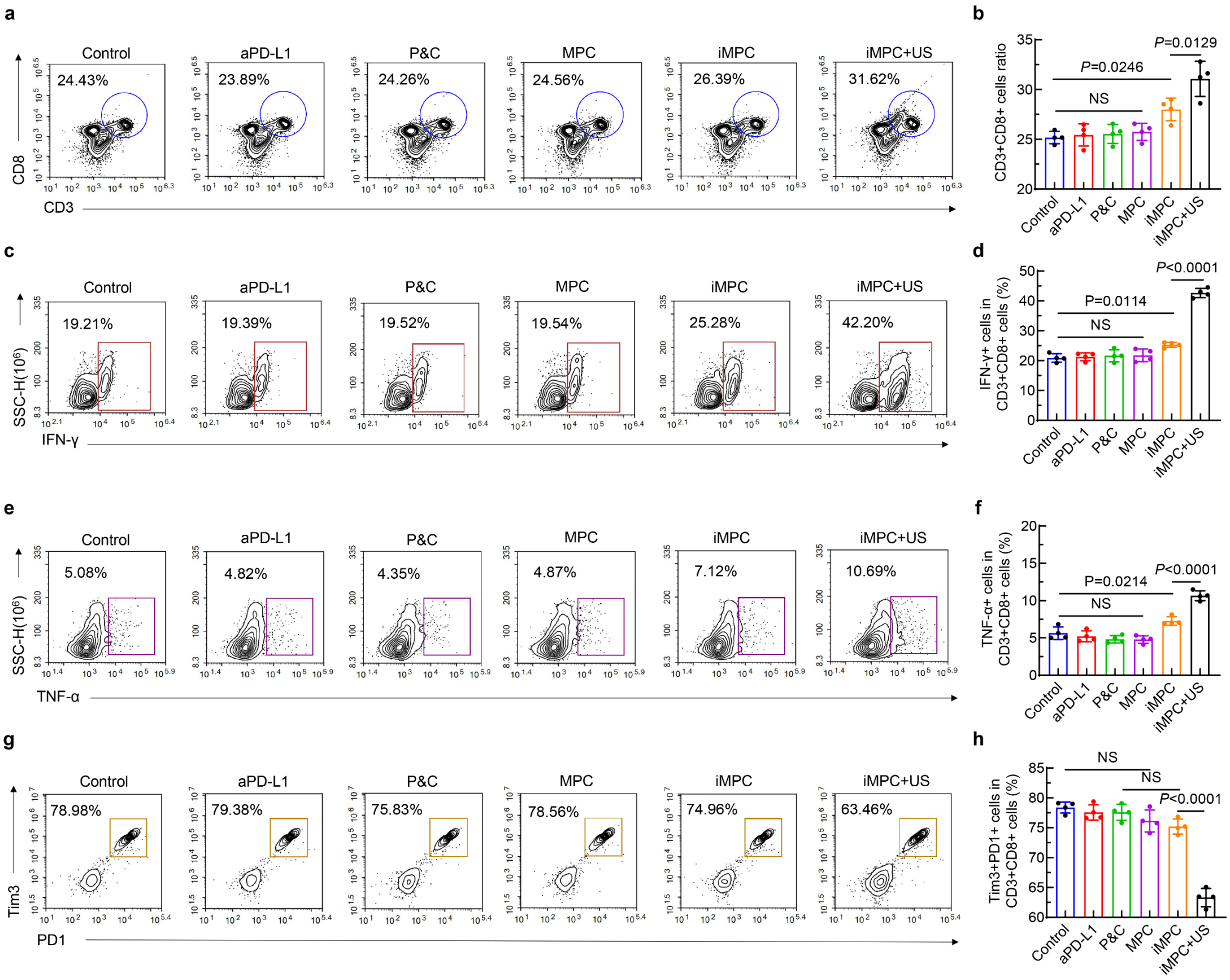

Supplementary Figure 7. Assess T cell activity using flow cytometry. a-b, The proportion of T-cells after 24 hours of treatment. **c-d**, The proportion of IFN-γ^{+} T-cells after 24 hours of different treatments. **e-f**, The proportion of TNF-α^{+} T-cells after 24 hours of different treatments. **g-h**, The co-expression ratio of PD1 and Tim3 in CD3^{+}CD8^{+} T cells. n = 5 biologically independent mice per group for data in **b, d, f, h**. Data are expressed as the mean ± SD. Statistical significances were determined using one-way ANOVA with Fisher's LSD post-hoc test. Comparisons were performed between different groups in **b, d, f, h**. Not significant (NS) is $P \geq 0.05$, and significant P values are shown. Source Data are provided as a Source Data file.

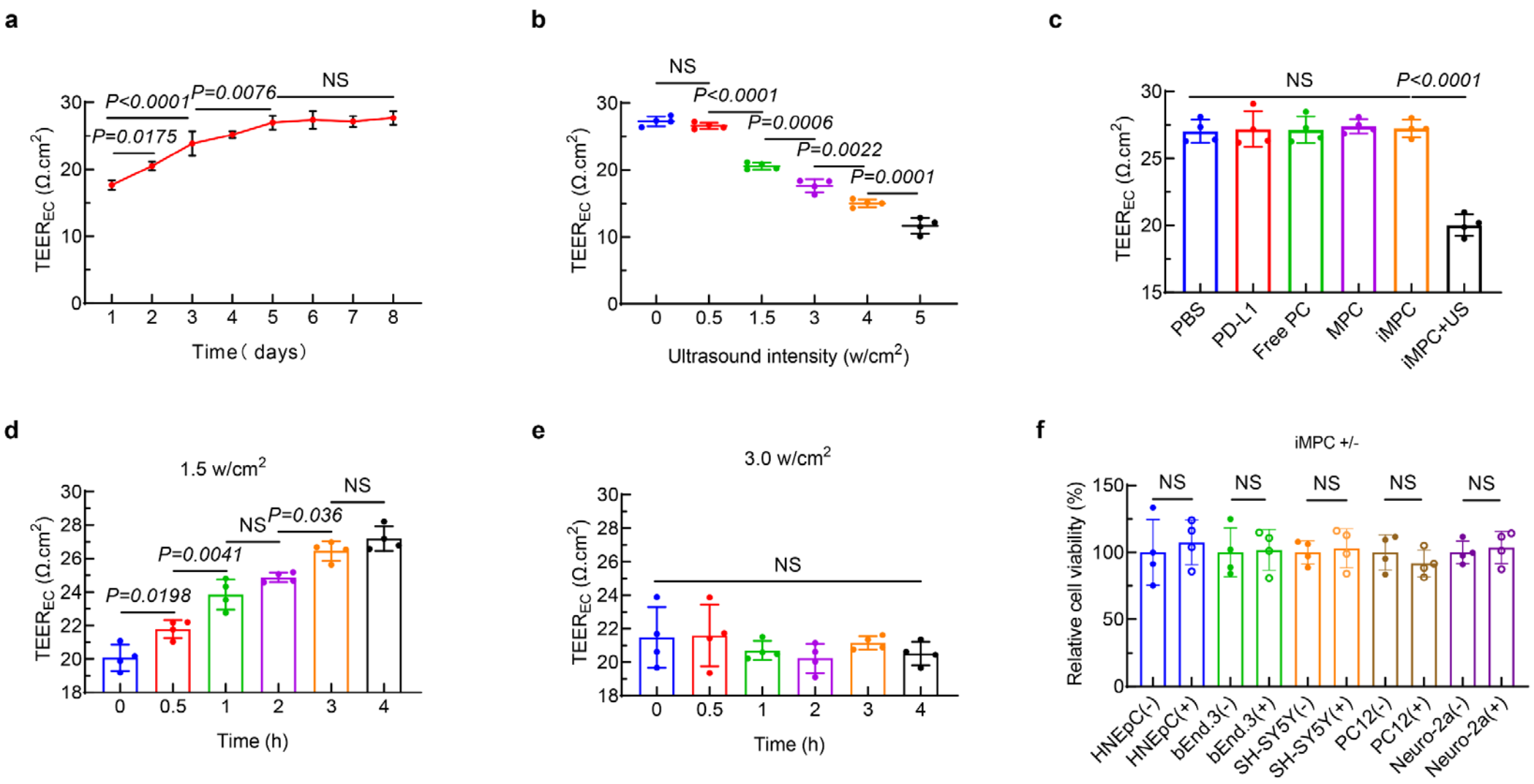

Supplementary Figure 8. Trans-epithelial electrical resistance (TEER) values in the nasal mucosal barrier (NMB) model. a, Changes in TEER values of the human nasal epithelial cell (HNEpC)-based NMB model (over 8 days). **b,** Changes in TEER values of the NMB model following treatment with different ultrasound intensities. **c,** Changes in TEER values of the NMB model after six different treatments. **d,** Ultrasonic intensity of 1.5 w/cm² was applied to NMB models, with changes in TEER values monitored over 4 hours. **e,** Ultrasonic intensity of 3.0 w/cm² was applied to NMB models, with changes in TEER values monitored over 4 hours. **f,** Cell viability of HNEpC, bEnd.3, SH-SY5Y, PC12, and Neuro-2a cells after treatment with iMPC nanoformulation. n = 4 biologically independent mice per group for data in **a-f**. Data are expressed as the mean ± SD. Statistical significances were determined using one-way ANOVA with Fisher's LSD post-hoc test. Comparisons were performed between different groups in **a-f**. Not significant (NS) is $P \geq 0.05$, and significant P values are shown. Source data are provided as a Source Data file.

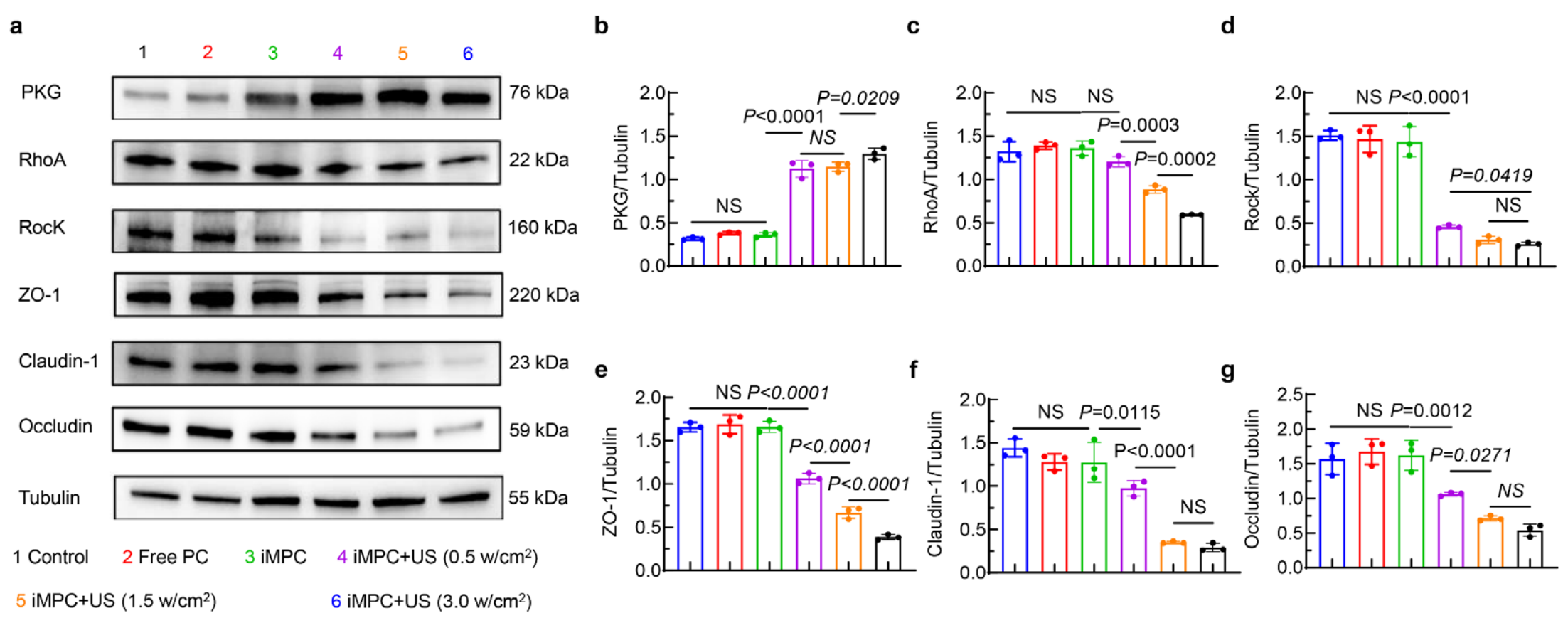

Supplementary Figure 9. Low-intensity pulsed ultrasound (LIPUS) disrupts tight junction proteins in the nasal mucosal barrier (NMB) model by activating the cGMP/PKG signaling pathway. a-g, The effects of different treatments on the cGMP/PKG signaling pathway and tight junction proteins. The gray value analysis was performed by using ImageJ software. n = 3 biologically independent mice per group for data in **b-g**. Data are expressed as the mean ± SD. Statistical significances were determined using one-way ANOVA with Fisher's LSD post-hoc test. Comparisons were performed between different groups in **b-g**. Not significant (NS) is $P \geq 0.05$, and significant P values are shown. Source data are provided as a Source Data file.

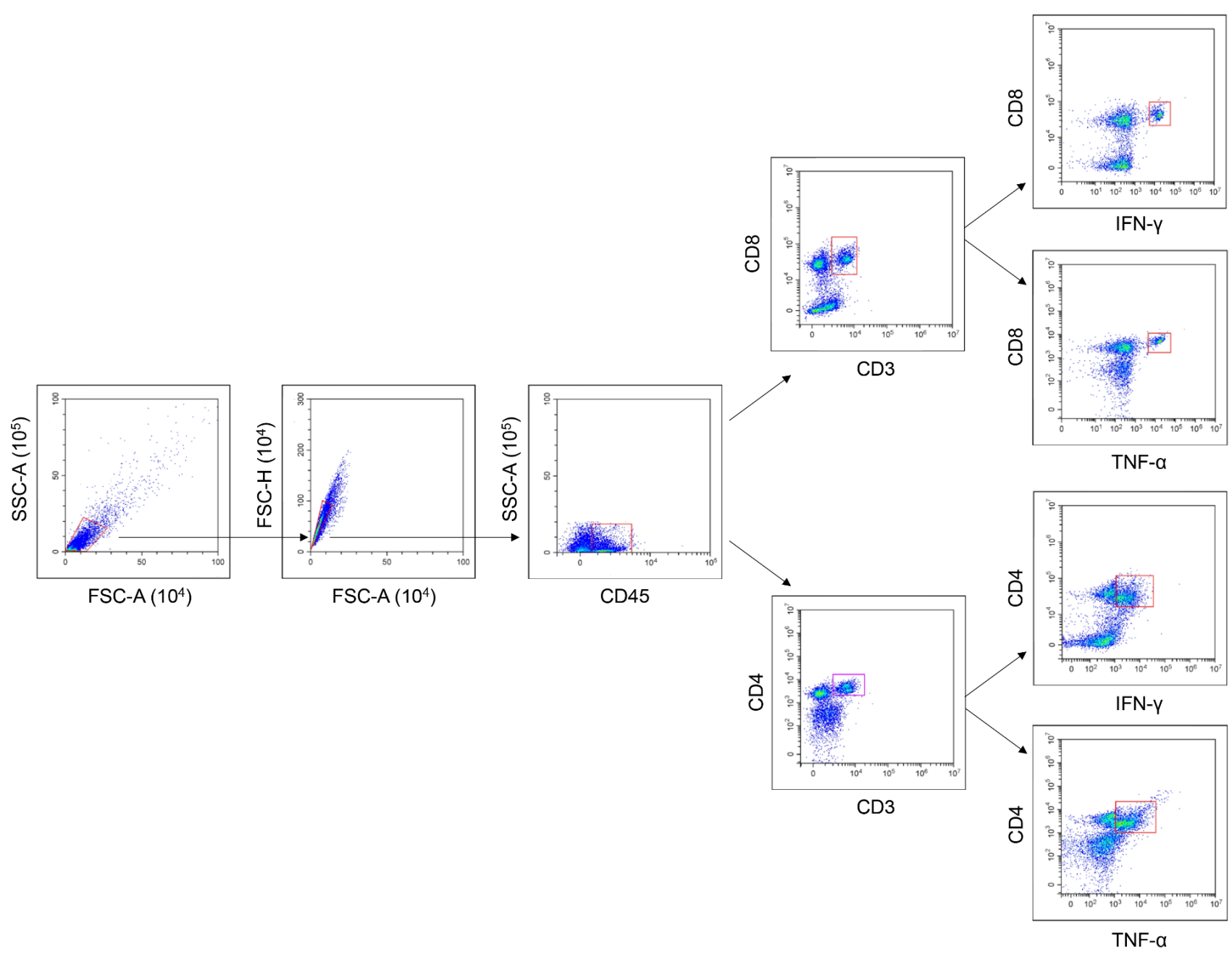

Supplementary Figure 10. Flow cytometry gating logic for CD3$^+$CD8$^+$ T cells and

$CD3^+CD4^+$ T cells. First, gating strategy for $CD3^+CD8^+$ T cells and $CD3^+CD4^+$ T cells were isolated from the total cells. Subsequently, gating strategy for the $CD3^+CD8^+$ T cells and $CD3^+CD4^+$ T cells expressing high levels of IFN-γ and TNF-α.

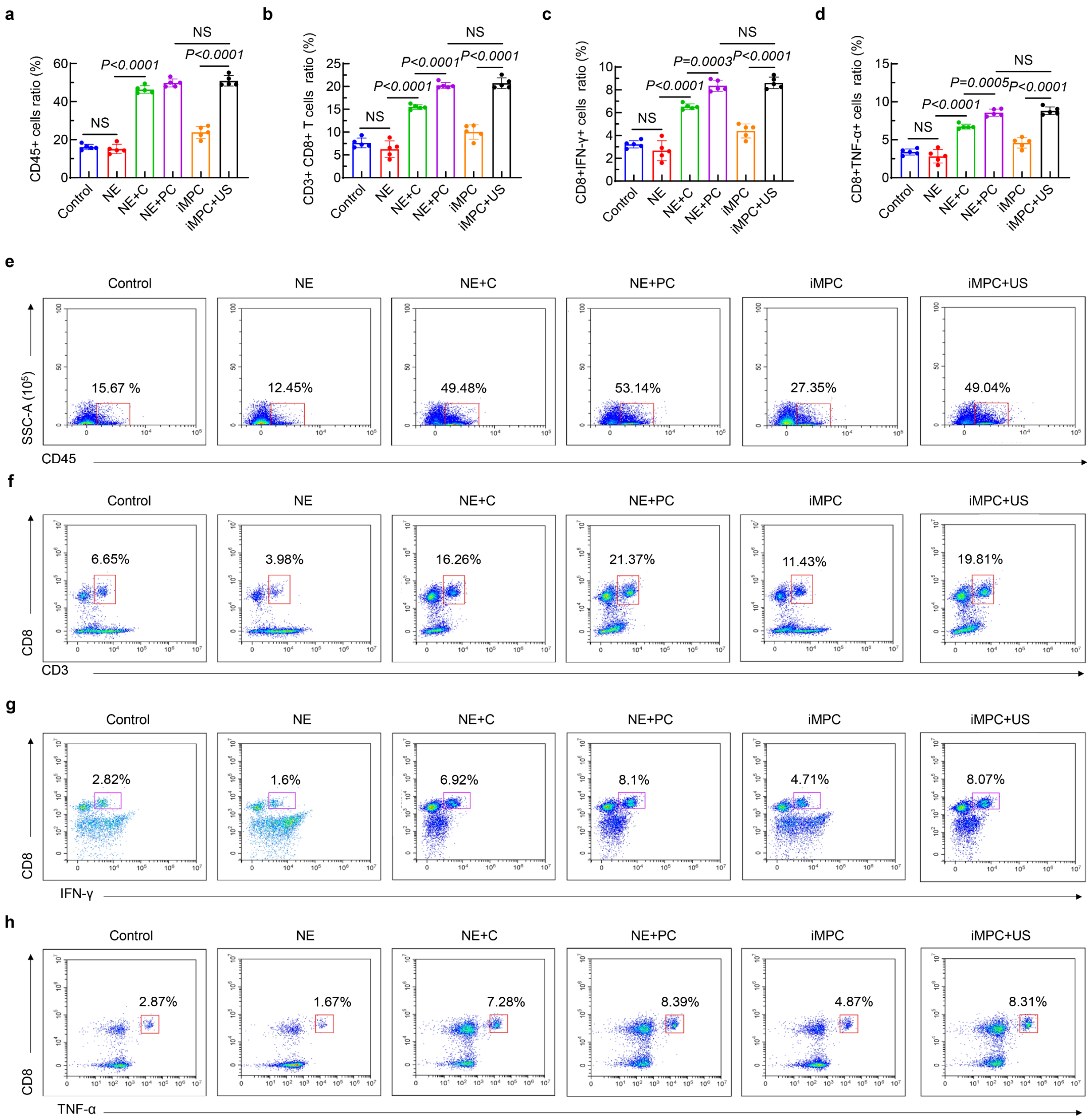

Supplementary Figure 11. β-adrenergic receptor blockade combined with aPD-L1 enhances the antitumor activity of $CD3^+CD8^+$ T cells. a, e, Proportion of $CD45^+$ lymphocytes after different treatments. **b, f,** Proportion of $CD3^+CD8^+$ T cells after different treatments. **c, g,** Proportion of IFN-γ^+CD8^+ T cells after different treatments. **d, h,** Proportion of TNF-α^+CD8^+ T cells after different treatments. n = 5 biologically independent mice per group for data in **a-d**. Data are expressed as the mean ± SD.

Statistical significances were determined using one-way ANOVA with Fisher's LSD post-hoc test. Comparisons were performed between different groups in **a-d**. Not significant (NS) is $P \geq 0.05$, and significant P values are shown. Source data are provided as a Source Data file.

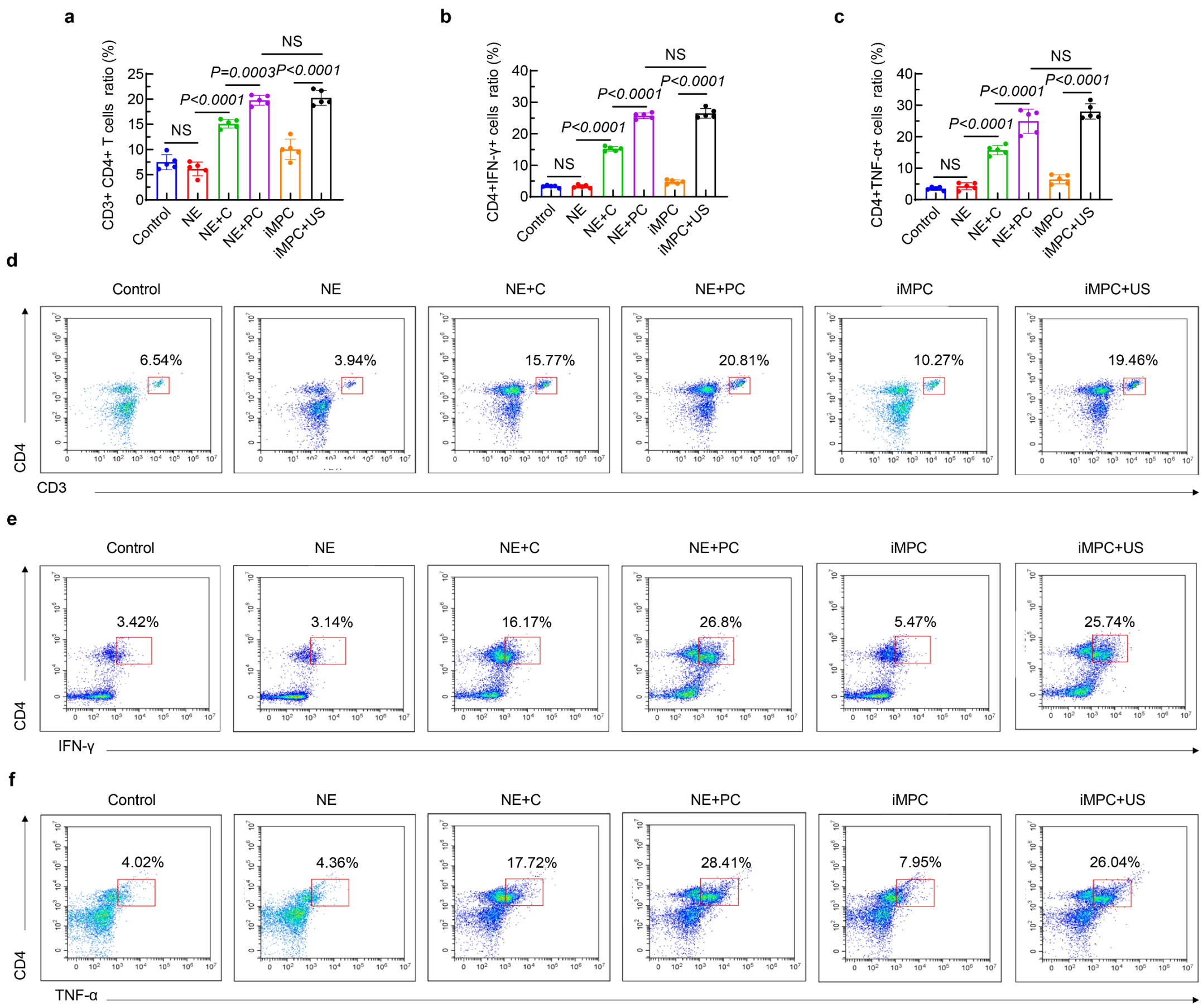

Supplementary Figure 12. β-adrenergic receptor blockade combined with aPD-L1 enhances $CD3^+CD4^+$ T cell activity. a, d, Proportion of $CD3^+CD4^+$ T cells after different treatments. **b, e,** Proportion of IFN-$\gamma^+$$CD4^+$ T cells after different treatments. **c, f,** Proportion of TNF-$\alpha^+$$CD4^+$ T cells after different treatments. n = 5 biologically independent mice per group for data in **a-c**. Data are expressed as the mean ± SD. Statistical significances were determined using one-way ANOVA with Fisher's LSD post-hoc test. Comparisons were performed between different groups in **a-c**. Not significant (NS) is $P \geq 0.05$, and significant P values are shown. Source data are provided as a Source Data file.

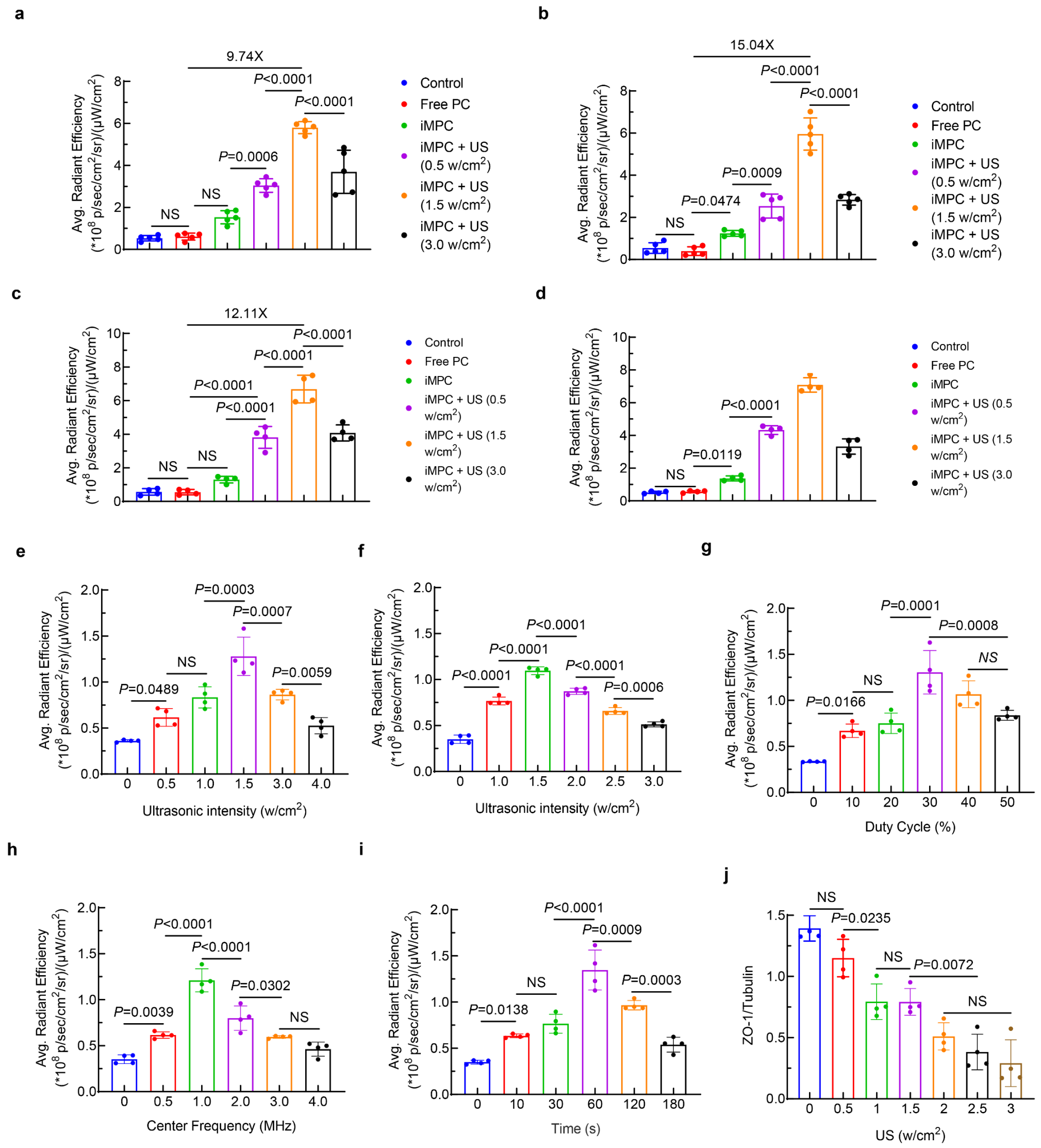

Supplementary Figure 13. In vivo imaging system (IVIS) for fluorescence signal quantification of aPD-L1-Cy5 in neural bypass, olfactory bulb, and tumor tissue. **a-b**, Quantitative analysis of aPD-L1-Cy5 fluorescence signal in the trigeminal neural bypass using ImageJ software 1 hour after intranasal administration. **c**, Quantitative analysis of aPD-L1-Cy5 fluorescence signal in the trigeminal neural bypass using ImageJ software 2 hours after intranasal administration. **d**, Quantitative analysis of aPD-L1-Cy5 fluorescence signal in the tumor area using ImageJ software 6 hours after intranasal administration. **e-f**, Fluorescence signals of iMPC-Cy5 in brain tumors after LIPUS treatment at different ultrasound intensities. **g,** Fluorescence signals of iMPC-Cy5 in brain tumors after LIPUS treatment with different duty cycles. **h,** Fluorescence signals of iMPC-Cy5 in brain tumors after LIPUS treatment at different center

frequencies. **i,** Fluorescence signals of iMPC-Cy5 in brain tumors after LIPUS treatment for different durations. **j**, ZO-1 reduces with increasing ultrasonic intensity. n = 5 biologically independent mice per group for data in **a-d**. n = 4 biologically independent mice per group for data in **e-j**. Data are expressed as the mean ± SD. Statistical significances were determined using one-way ANOVA with Fisher's LSD post-hoc test. Comparisons were performed between different groups in **a-j**. Not significant (NS) is $P \geq 0.05$, and significant P values are shown. Source data are provided as a Source Data file.

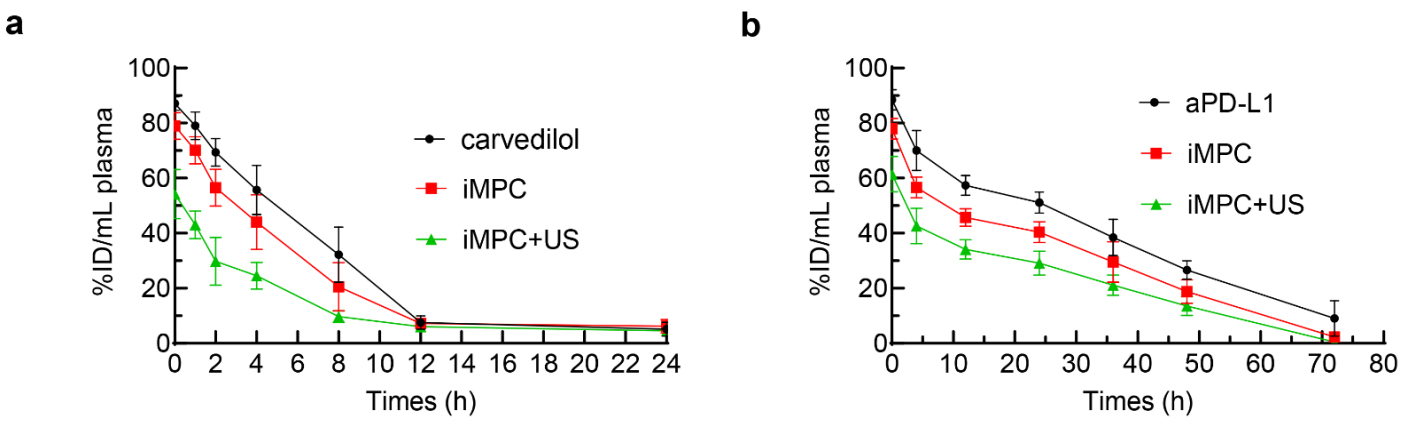

Supplementary Figure 14. Pharmacokinetic monitoring of carvedilol, aPD-L1, and iMPC nano-formulations. a. Percentage change in blood concentration of carvedilol. **b.** Percentage change in blood concentration of aPD-L1. n = 3 biologically independent mice per group for data in **a-b**. Data are expressed as the mean ± SD. Source data are provided as a Source Data file.

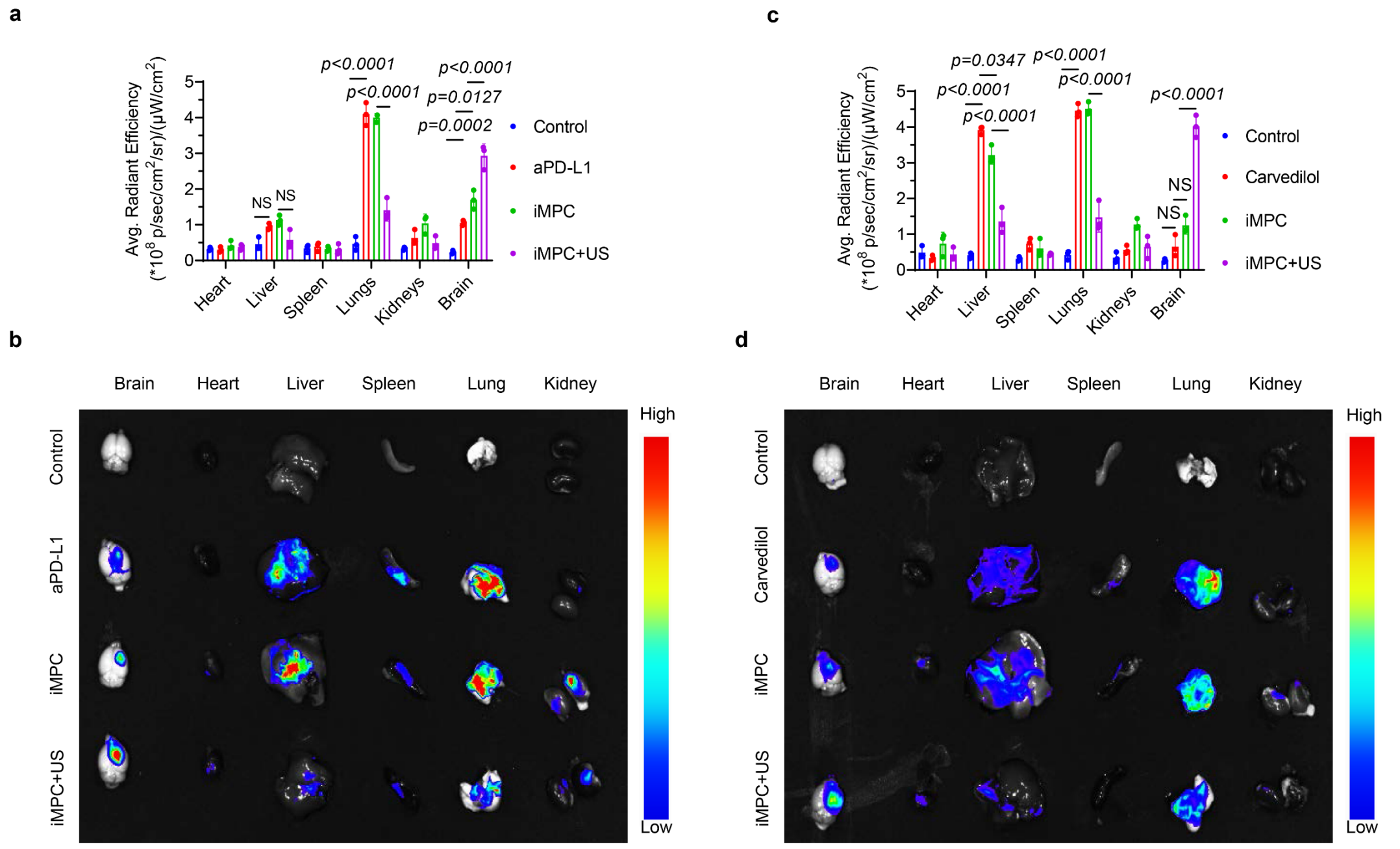

Supplementary Figure 15. Organ distribution of carvedilol, aPD-L1, and iMPC nano-formulations. a-b, Distribution of aPD-L1 and iMPC nano-formulations in heart, liver, spleen, lung, and kidney. **c-d,** Distribution of carvedilol and iMPC nano-formulations in heart, liver, spleen, lung, and kidney. n = 3 biologically independent mice per group for data in **a** and **c**. Data are expressed as the mean ± SD. Statistical significances were determined using one-way ANOVA with Fisher's LSD post-hoc test. Comparisons were performed between different groups in **a** and **c**. Not significant (NS) is $P \geq 0.05$, and significant P values are shown. Source data are provided as a Source Data file.

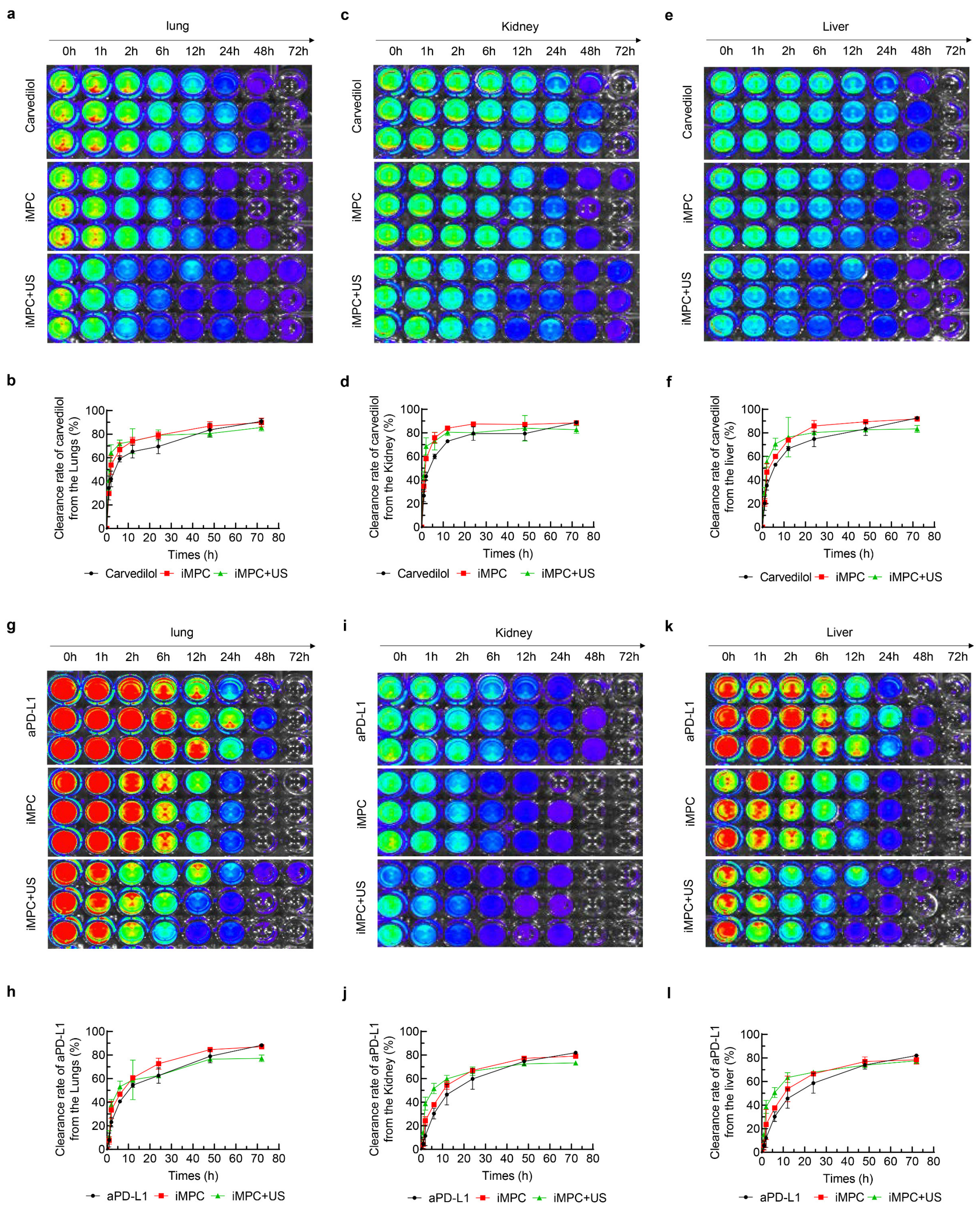

Supplementary Figure 16. Cardiac clearance of carvedilol, aPD-L1, and the iMPC nano-formulations. a-b, Pulmonary clearance of carvedilol and the iMPC nano-formulations. **c-d,** Renal clearance of carvedilol and the iMPC nano-formulations. **e-f,** Hepatic clearance of carvedilol and the iMPC nano-formulations. **g-h,** Pulmonary clearance of aPD-L1 and the iMPC nano-formulations. **i-j,** Renal clearance of aPD-L1 and the iMPC nano-formulations. **k-l,** Hepatic clearance of aPD-L1 and the iMPC nano-formulations. n = 3 biologically independent mice per group for data in **a-l**. n = 5

biologically independent mice per group for data in **a-l**. Data are expressed as the mean ± SD. Source data are provided as a Source Data file.

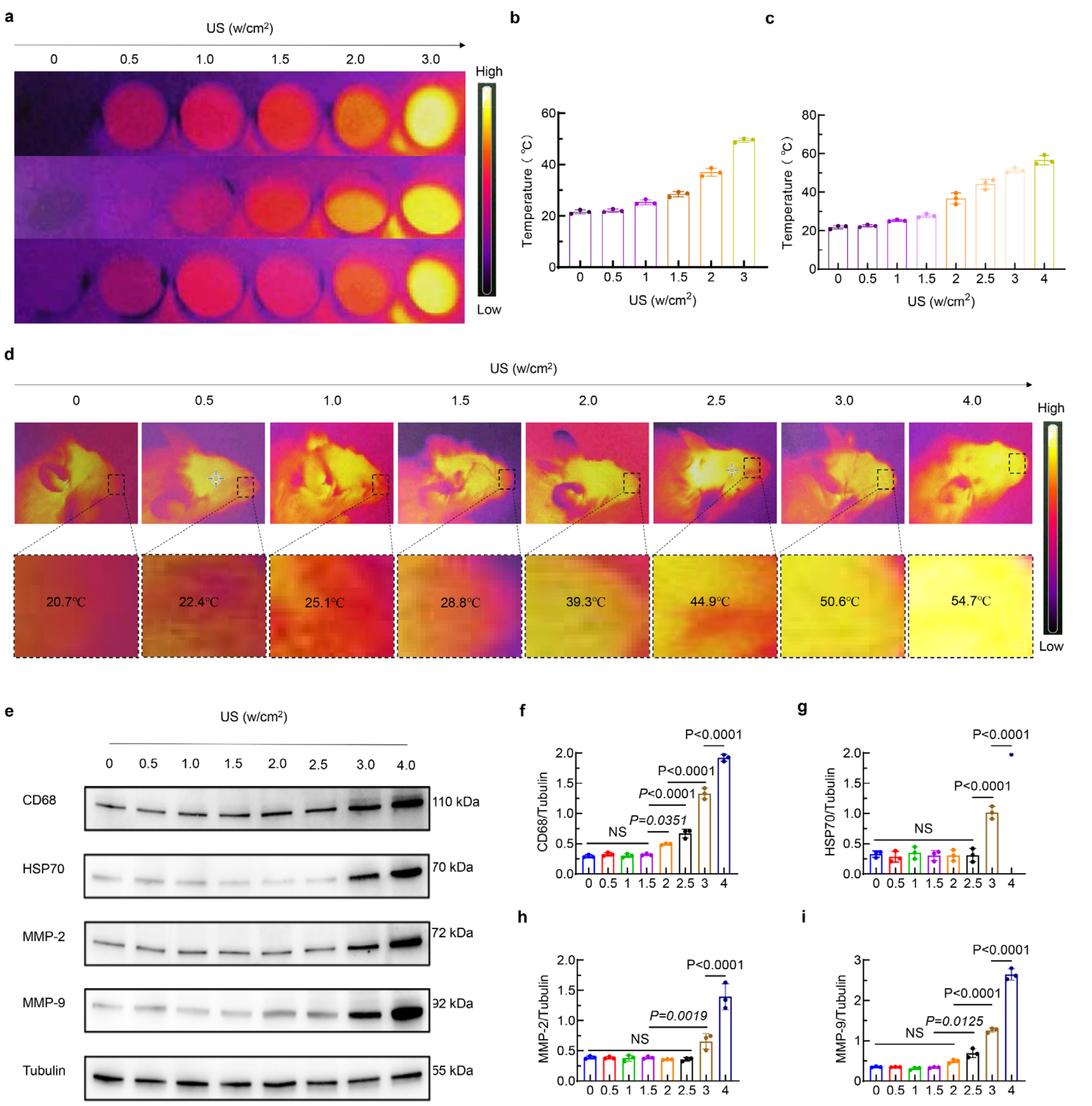

Supplementary Figure 17. Thermal injury of LIPUS on nasal mucosa. a-b, Temperature changes in the nasal mucosal barrier (NMB) model constructed from human nasal epithelial cells (HNEpC). **c-d,** Regional temperature changes in mouse nasal mucosa. **e-h,** Expression levels of HSP70, CD68, and MMP-9. Gray-scale analysis was performed using ImageJ software. n = 3 biologically independent mice per group for data in **a-c**. n = 3 biologically independent mice per group for data in **f-i**. Data are expressed as the mean ± SD. Statistical significances were determined using one-way ANOVA with Fisher's LSD post-hoc test. Comparisons were performed between

different groups in **a-c** and **f-i**. Not significant (NS) is $P \geq 0.05$, and significant P values are shown. Source data are provided as a Source Data file.

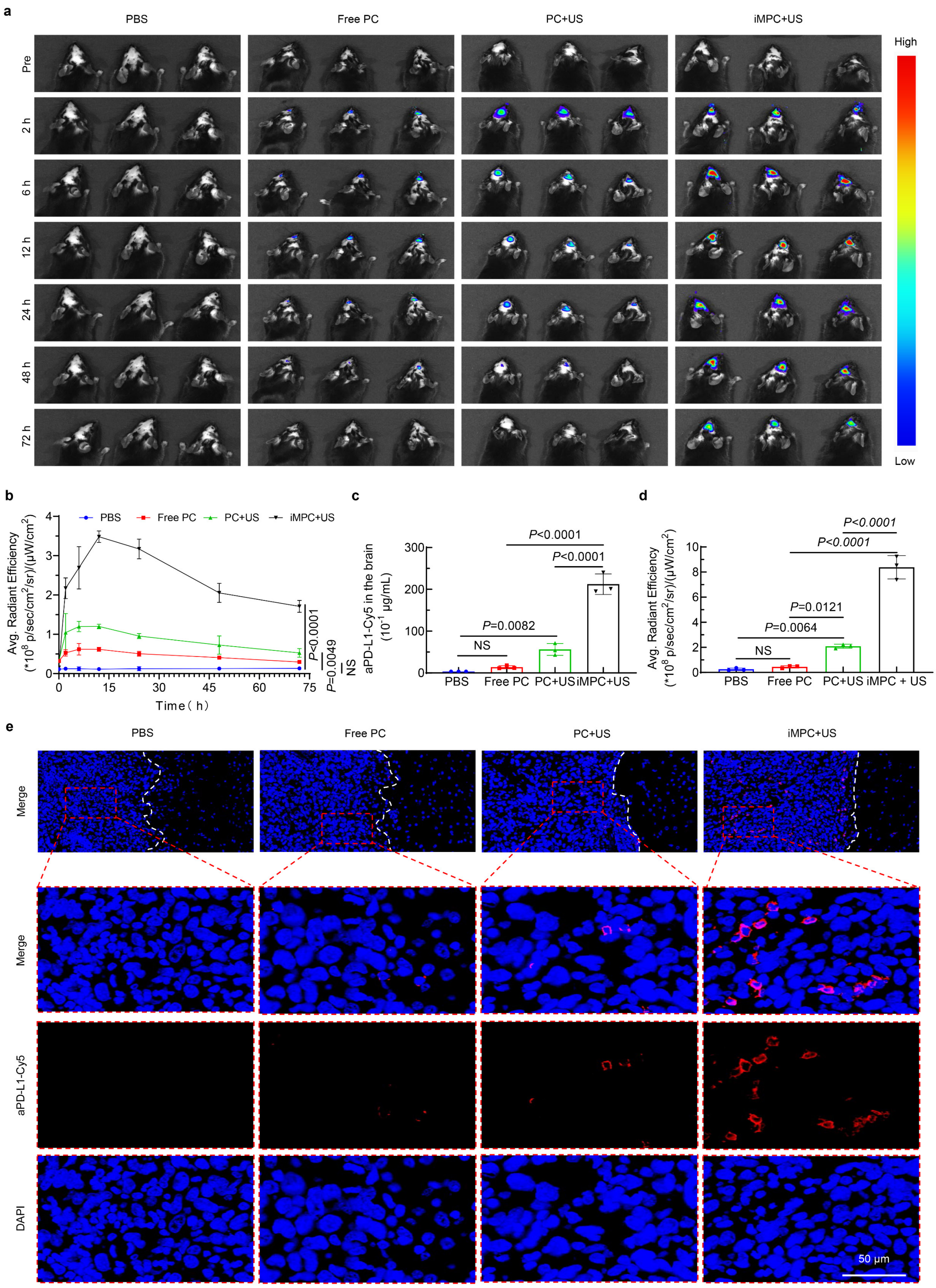

Supplementary Figure 18. Low-intensity pulsed ultrasound (LIPUS)-mediated efficient nose-to-brain delivery of drugs. a, *In vivo* imaging of GBM mice subjected

to four different treatments. **b,** Quantification of fluorescence signals. **c,** Fluorescence intensity of aPD-L1 in brain tissues, aPD-L1 labeled with Cy5 NHS. **d,** Fluorescence signals of aPD-L1 in tumor tissues of GBM mice. **e,** Immunofluorescence staining of tumor tissue sections from GBM mice. n = 3 biologically independent mice per group for data in **a-e**. Data are expressed as the mean ± SD. Statistical significances were determined using one-way ANOVA with Fisher's LSD post-hoc test. Comparisons were performed between different groups in **b-d**. Not significant (NS) is $P \geq 0.05$, and significant P values are shown. Source data are provided as a Source Data file.

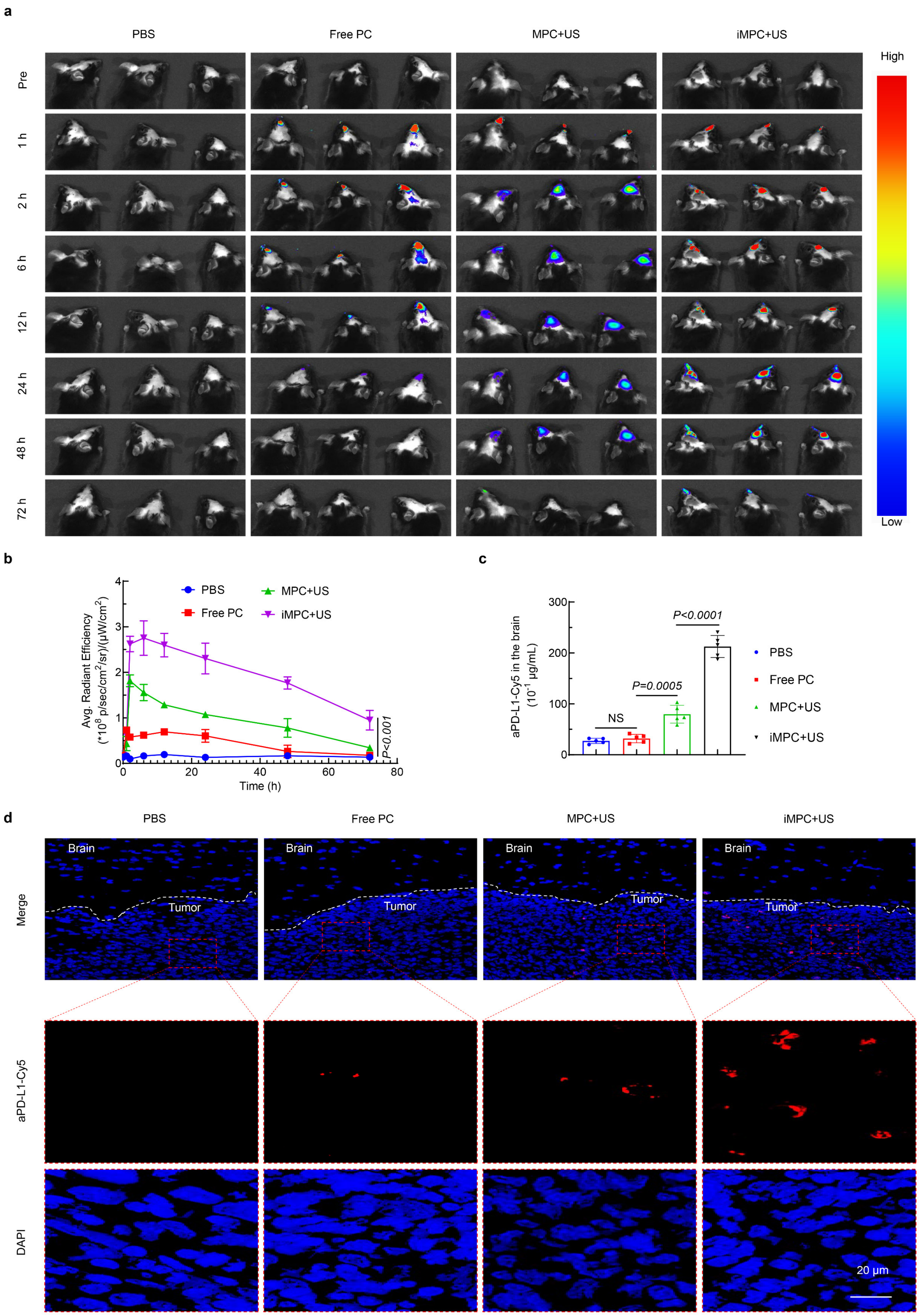

Supplementary Figure 19. iRGD-modified MPC enhances its targeted tumor delivery efficiency. a, *In vivo* imaging of GBM mice subjected to four different treatments. **b,** Quantification of fluorescence signals. **c,** Fluorescence intensity of aPD-L1 in brain tissues, aPD-L1 labeled with Cy5 NHS. **d,** Fluorescence signals of aPD-L1

in tumor tissues of GBM mice. n = 3 biologically independent mice per group for data in **a-b**. n = 5 biologically independent mice per group for data in **c**. Data are expressed as the mean ± SD. Statistical significances were determined using one-way ANOVA with Fisher's LSD post-hoc test. Comparisons were performed between different groups in **b-c**. Not significant (NS) is $P \geq 0.05$, and significant P values are shown. Source data are provided as a Source Data file.

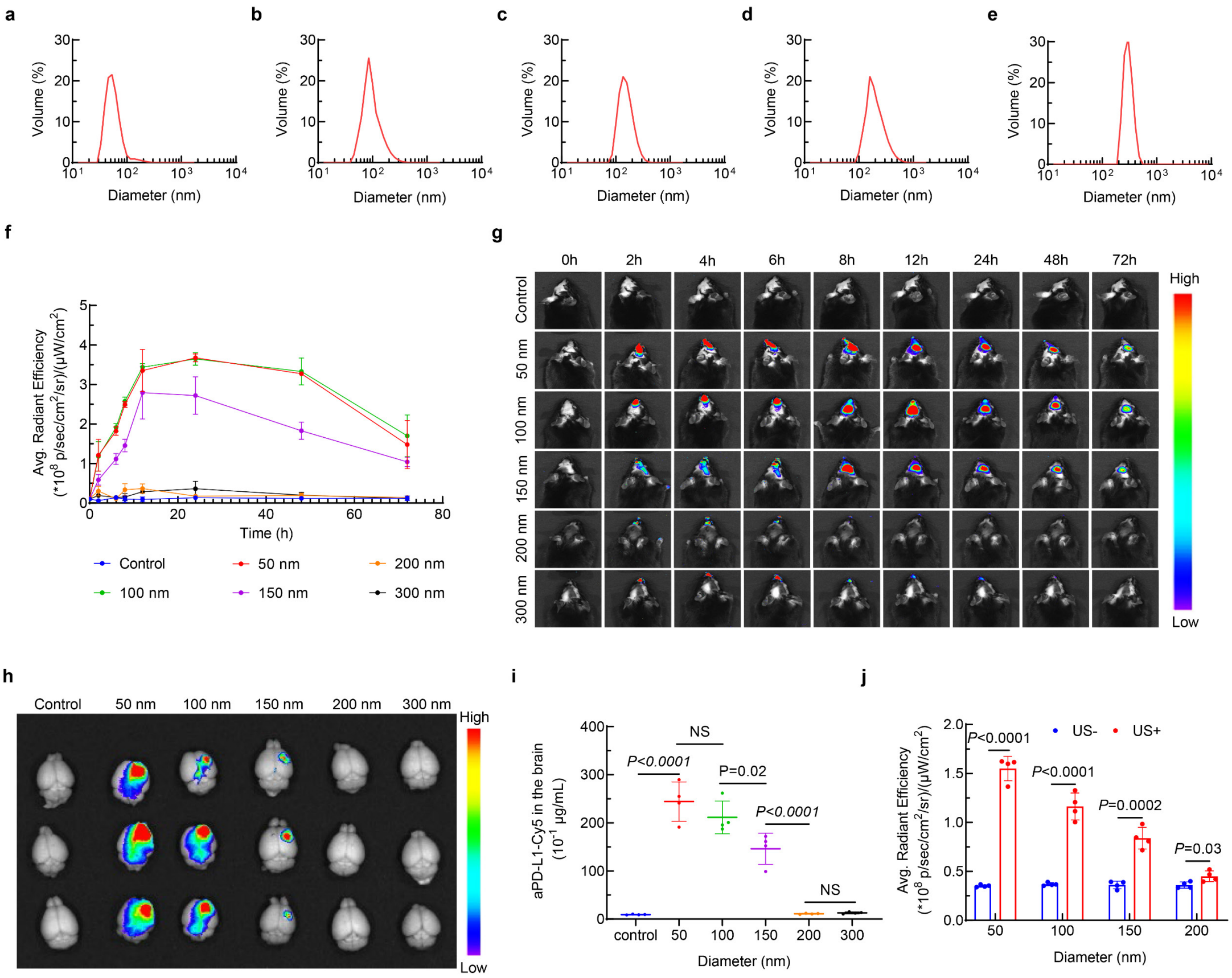

Supplementary Figure 20. Low-intensity pulsed ultrasound (LIPUS)-mediated nose-to-brain delivery efficiency of iMPC with different particle sizes. a-e, Particle size distribution diagrams of iMPC with sizes of 50, 100, 150, 200, and 300 nm, respectively. **f-g,** Distribution profiles of iMPC with different particle sizes over various time periods. **h,** aPD-L1-Cy5 distribution in brain tumors. **i,** Quantitative analysis of aPD-L1 in brain tissue using a microplate reader. **j,** Fluorescence signal of iMPC-Cy5 in brain tumors. n = 4 biologically independent mice per group for data in **i-j**. Data are expressed as the mean ± SD. Statistical significances were determined using one-way ANOVA with Fisher's LSD post-hoc test. statistical analysis between two groups was

performed using unpaired two-tailed Student's t-test. Comparisons were performed between different groups in **i-j**. Not significant (NS) is $P \geq 0.05$, and significant P values are shown. Source data are provided as a Source Data file.

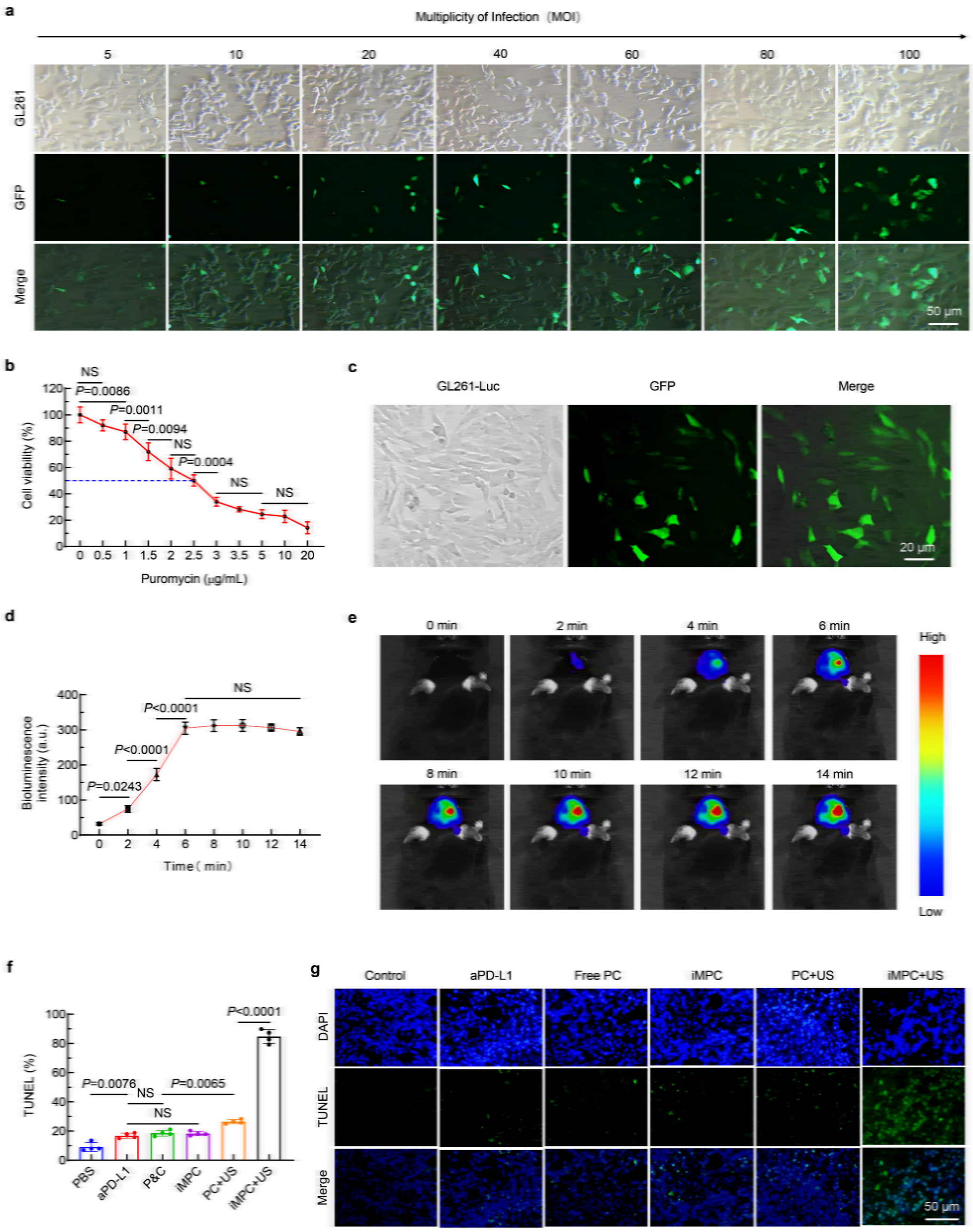

Supplementary Figure 21. Construction and screening of GL261-Luc cell line. a, The transfection efficiency of GL261 cells at different multiplicity of infection (MOI). **b**, The effect of different concentrations of puromycin on the viability of GL261 cells, with an IC50 of 2.5 μg/mL. **c**, Stable GFP-expressing GL261-Luc cell line. **d-e**, The stable bioluminescence signal time period (6-14 min) in GL261-Luc tumor-bearing

mice. **f-g**, TUNEL apoptosis detection in tumor tissues. Statistical analysis of fluorescence signal intensity by ImageJ software. n = 4 biologically independent mice per group for data in **b, d and f**. Data are expressed as the mean ± SD. Statistical significances were determined using one-way ANOVA with Fisher's LSD post-hoc test. Comparisons were performed between different groups in **b, d and f**. Not significant (NS) is $P \geq 0.05$, and significant P values are shown. Source data are provided as a Source Data file.

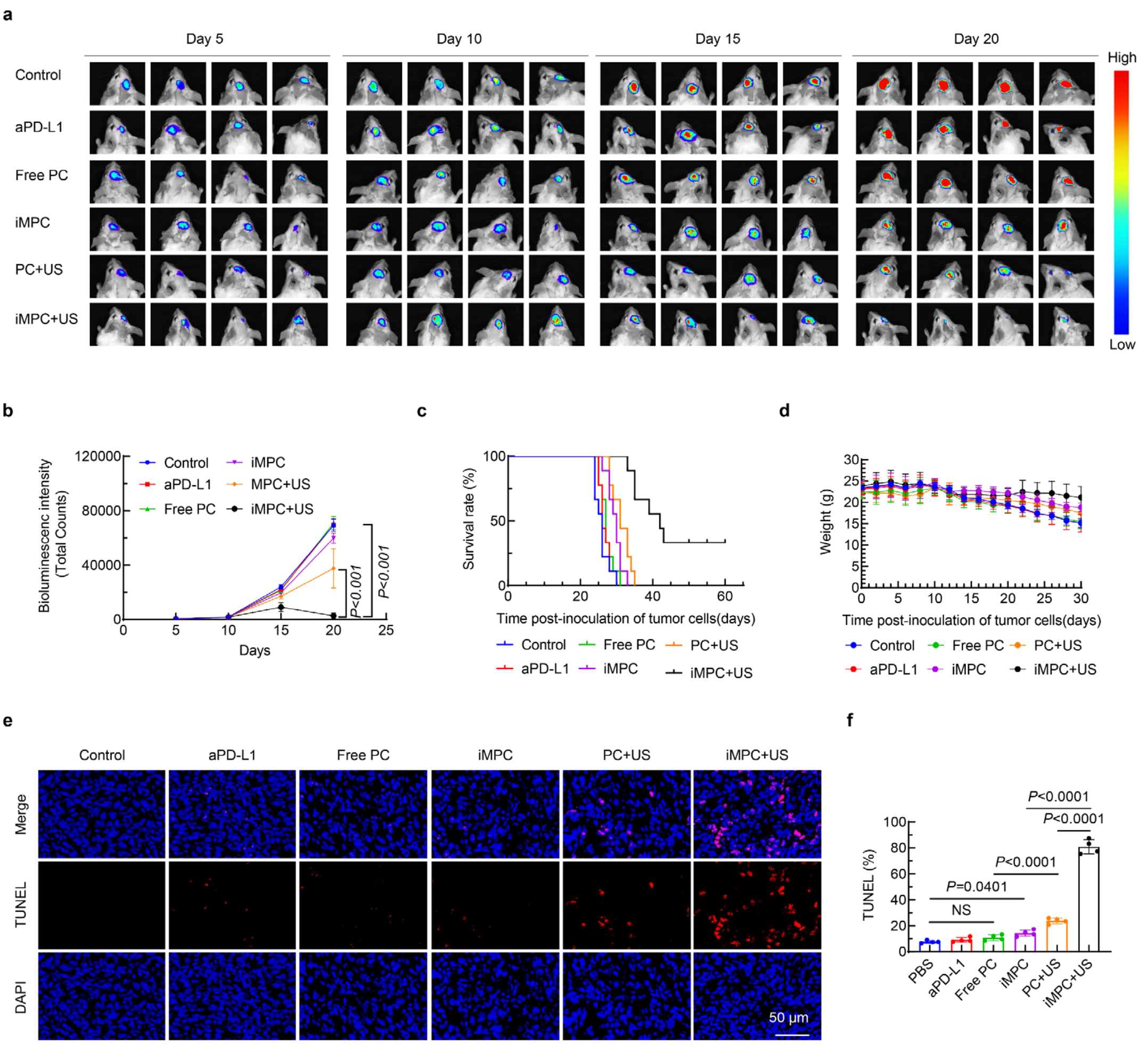

Supplementary Figure 22. Evaluation of antitumor efficacy. a-b, *In vivo* imaging system (*IVIS*) assessment of tumor growth inhibition in G422-Luc cell-implanted in situ glioblastoma (GBM) mouse models across different treatment groups. **c,** Survival curves of GBM tumor-bearing mice in different treatment groups (n = 10). Survival analysis was compared using the log-rank test. . **d,** Body weight changes of GBM mice

in different treatment groups (n = 10). **e-f,** Apoptotic proportion in tumor tissues of GBM mice across different treatment groups. n = 4 biologically independent mice per group for data in **a-b** and **f**. Data are expressed as the mean ± SD. Statistical significances were determined using one-way ANOVA with Fisher's LSD post-hoc test. Comparisons were performed between different groups in **a-b** and **f**. Not significant (NS) is $P \geq 0.05$, and significant P values are shown. n = 10 biologically independent mice per group for data in **c-d**. Survival analysis was compared using the log-rank test. Source data are provided as a Source Data file.

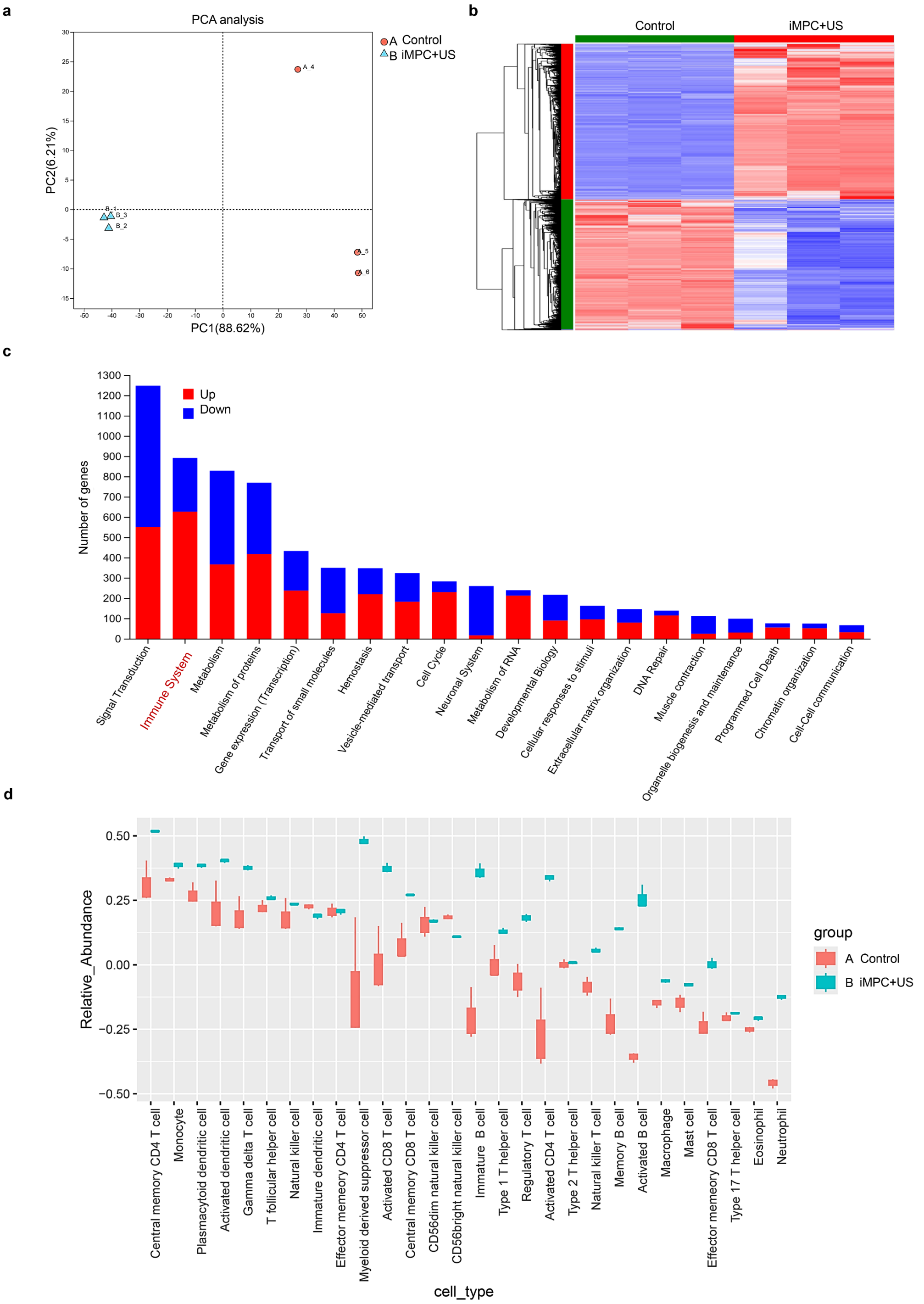

Supplementary Figure 23. Transcriptome sequencing analysis of tumor tissues. a, Analysis of individual gene variations in tumor tissues from two treatment groups. **b**, Analysis of differential gene expression levels in tumor tissues from two treatment groups. **c**, Enrichment analysis of differentially expressed genes to identify key pathway changes. **d**, Enrichment analysis of differentially expressed genes in immune system cell subtypes.

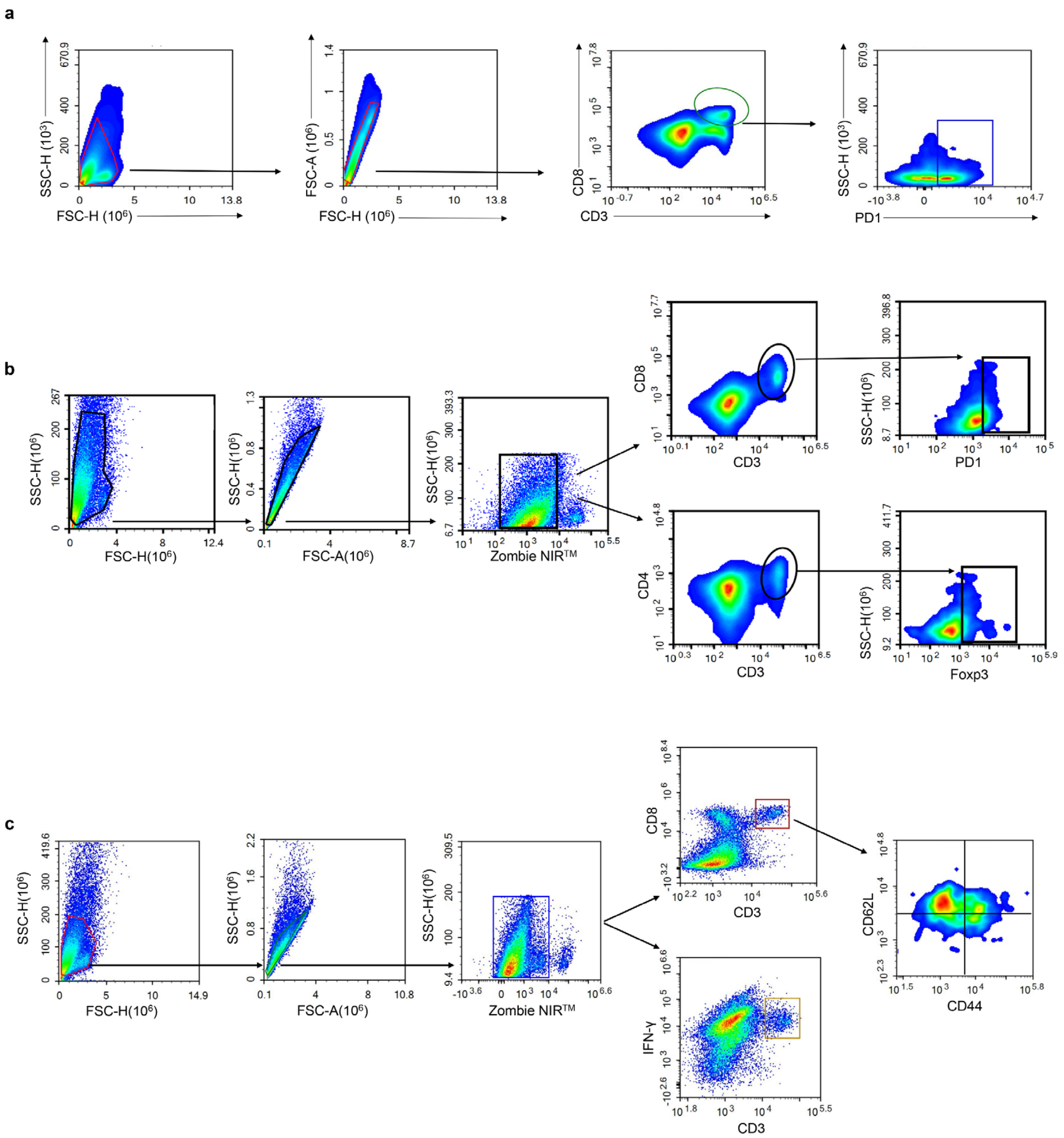

Supplementary Figure 24. Gating strategies for flow cytometry analysis of different immune cells. a, Gating strategy to identify $CD3^+CD8^+$ T cells and assess PD1 expression. **b**, Gating approach to analyze $CD3^+CD8^+$ T cells, $CD3^+CD4^+$ T cells, and Treg cells. **c**, Flow cytometry gating to determine $CD3^+CD8^+$ T cells, IFN-γ^+CD3^+ T cells, and memory T cells.

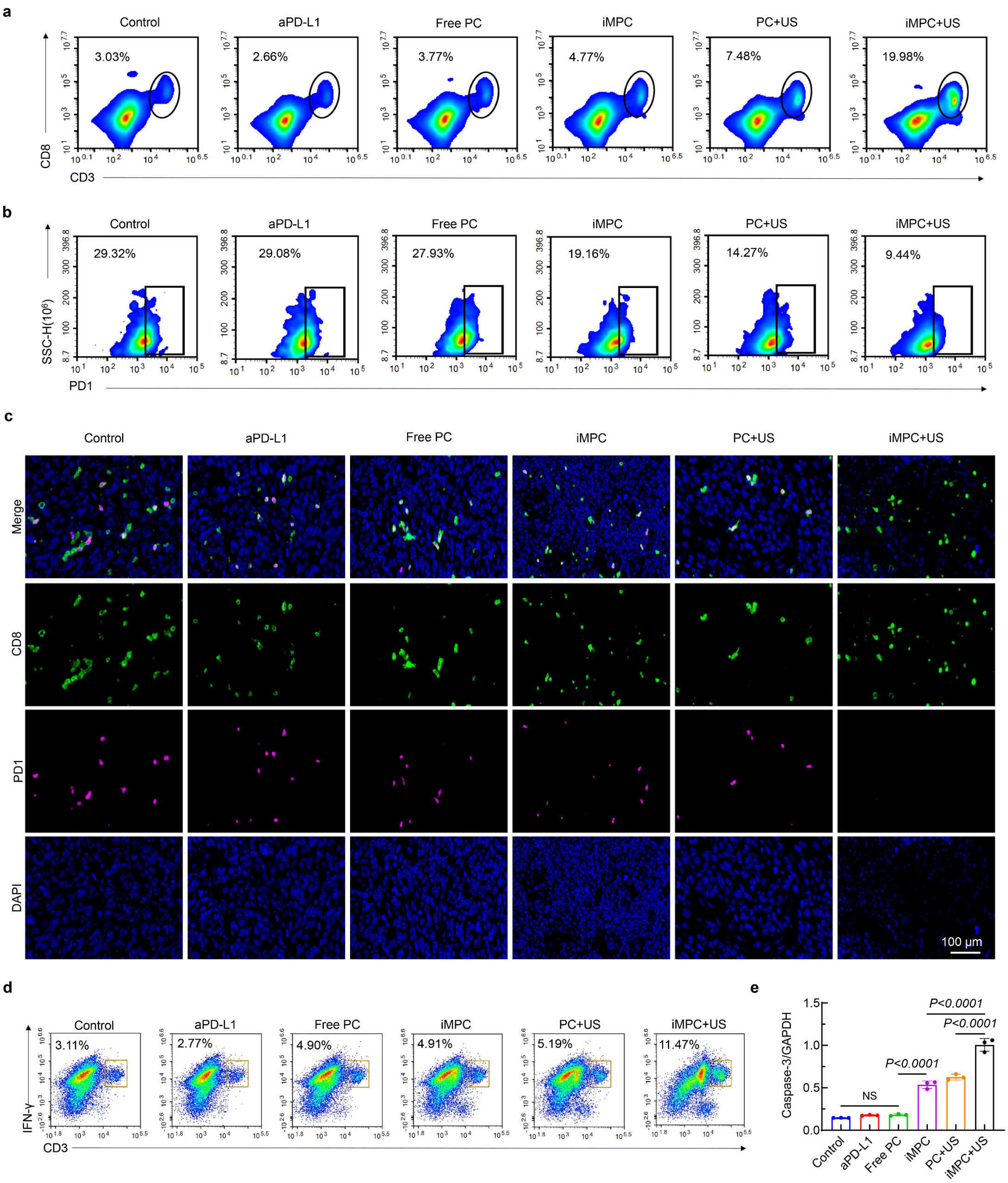

Supplementary Figure 25. Assessment of T cell activity by flow cytometry and immunofluorescence. a, The infiltration ratio of CD3$^+$CD8$^+$ T cells in tumor tissues after 5 days of different treatments. **b**, The expression of PD1 in CD3$^+$CD8$^+$ T cells in tumor tissues after 5 days of different treatments. **c,** Immunofluorescence analysis of CD3$^+$CD8$^+$ T cell infiltration and PD1 expression in tumor tissues (blue: DAPI; green: Alexa Fluor 488-CD8; red: CY3-PD1). **d,** The expression of Tumor-specific T cells in tumor tissues after 5 days of different treatments. **e,** The expression of caspase-3 in tumor tissues, ImageJ software statistical analysis. n = 5 biologically independent mice per group for data in **e**. Data are expressed as the mean ± SD. Statistical significances

were determined using one-way ANOVA with Fisher's LSD post-hoc test. Comparisons were performed between different groups in **e**. Not significant (NS) is $P \geq 0.05$, and significant P values are shown. Source data are provided as a Source Data file.

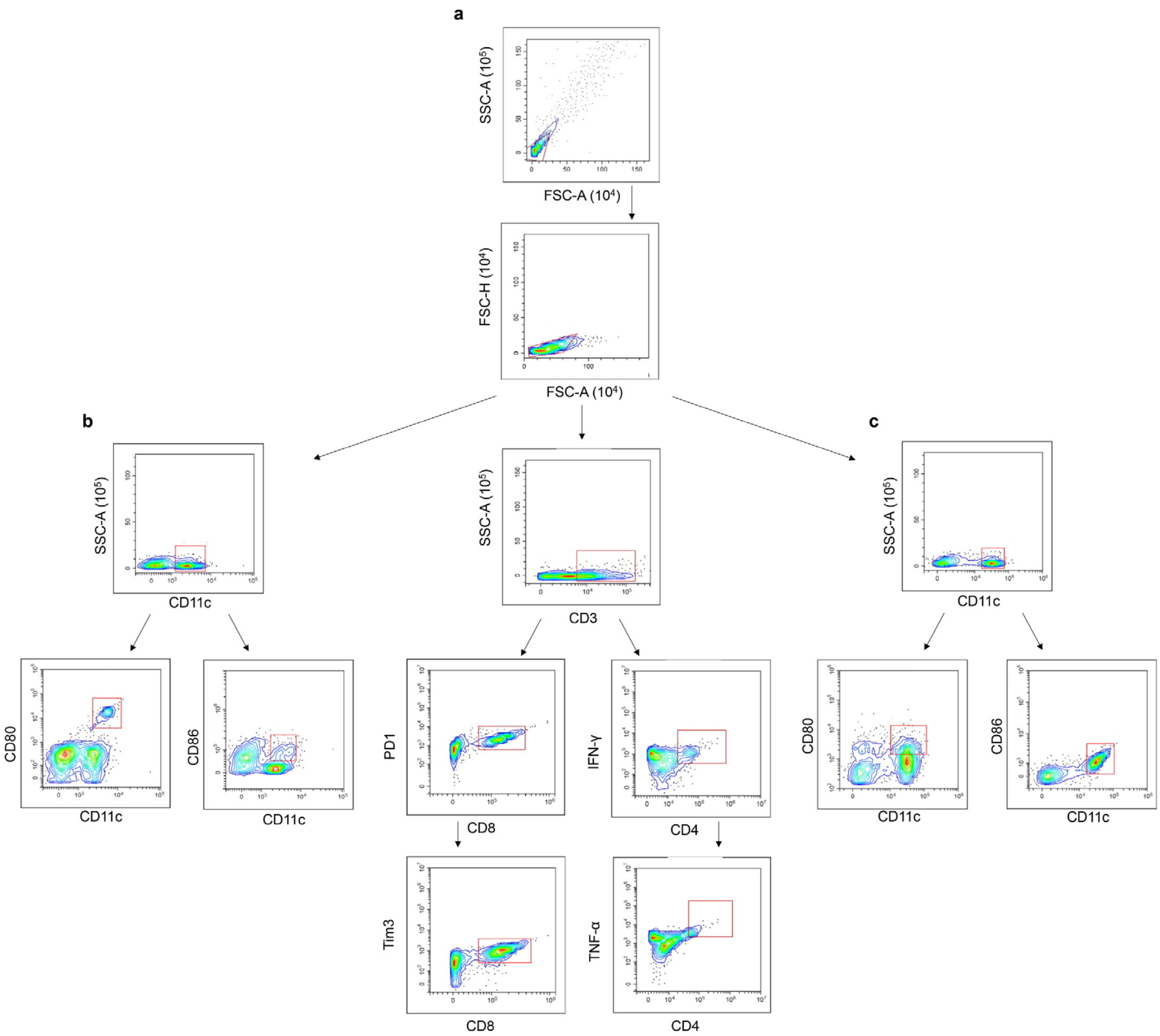

Supplementary Figure 26. Flow cytometry gating logic for dendritic cells (DCs), CD8^{+} T cells, and CD4^{+} T cells in cervical deep lymph nodes and axillary lymph nodes. **a**, Gating strategy for CD3^{+} T cells in deep cervical lymph nodes. Flow cytometry gating to determine PD1^{+}CD8^{+} T cells, Tim3^{+}CD8^{+} T cells, IFN-γ^{+}CD4^{+} T cells and TNF-α^{+}CD4^{+} T cells. **b**, Gating strategy for DCs in axillary lymph nodes. Flow cytometry gating to determine CD80^{+} DCs and CD86^{+} DCs. **c,** Gating strategy for DCs in deep cervical lymph nodes. Flow cytometry gating to determine CD80^{+} DCs and CD86^{+} DCs.

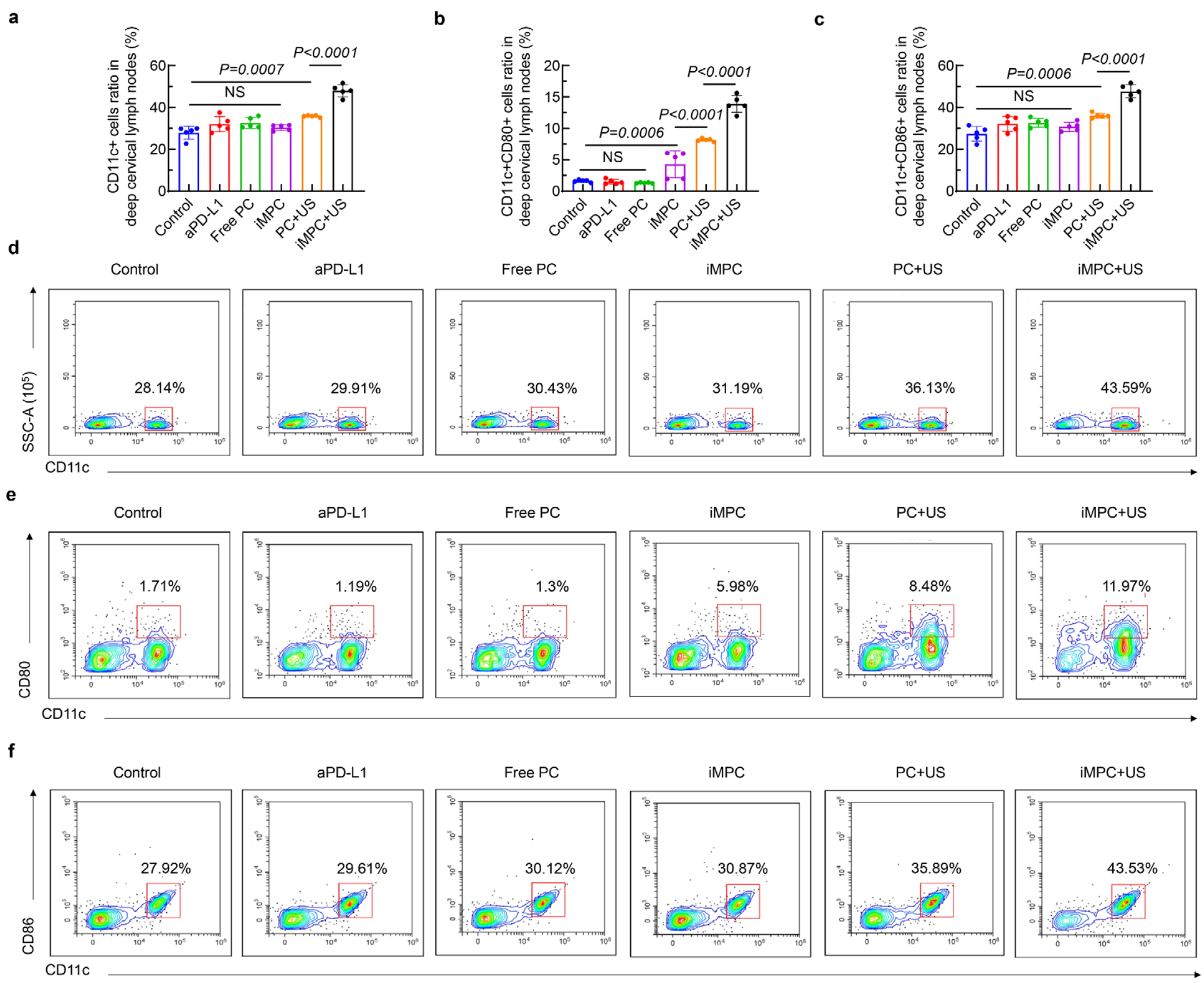

Supplementary Figure 27. The proportion of dendritic cells (DCs) in deep cervical lymph nodes increased, with enhanced activity. a, d, Proportion of DCs in deep cervical lymph nodes. **b, e,** Proportion of DCs highly expressing CD80 in deep cervical lymph nodes. **c, f,** Proportion of DCs highly expressing CD86 in deep cervical lymph nodes. n = 5 biologically independent mice per group for data in c-f and **a-c**. Data are expressed as the mean ± SD. Statistical significances were determined using one-way ANOVA with Fisher's LSD post-hoc test. Comparisons were performed between different groups in **a-c**. Not significant (NS) is $P \geq 0.05$, and significant P values are shown. Source data are provided as a Source Data file.

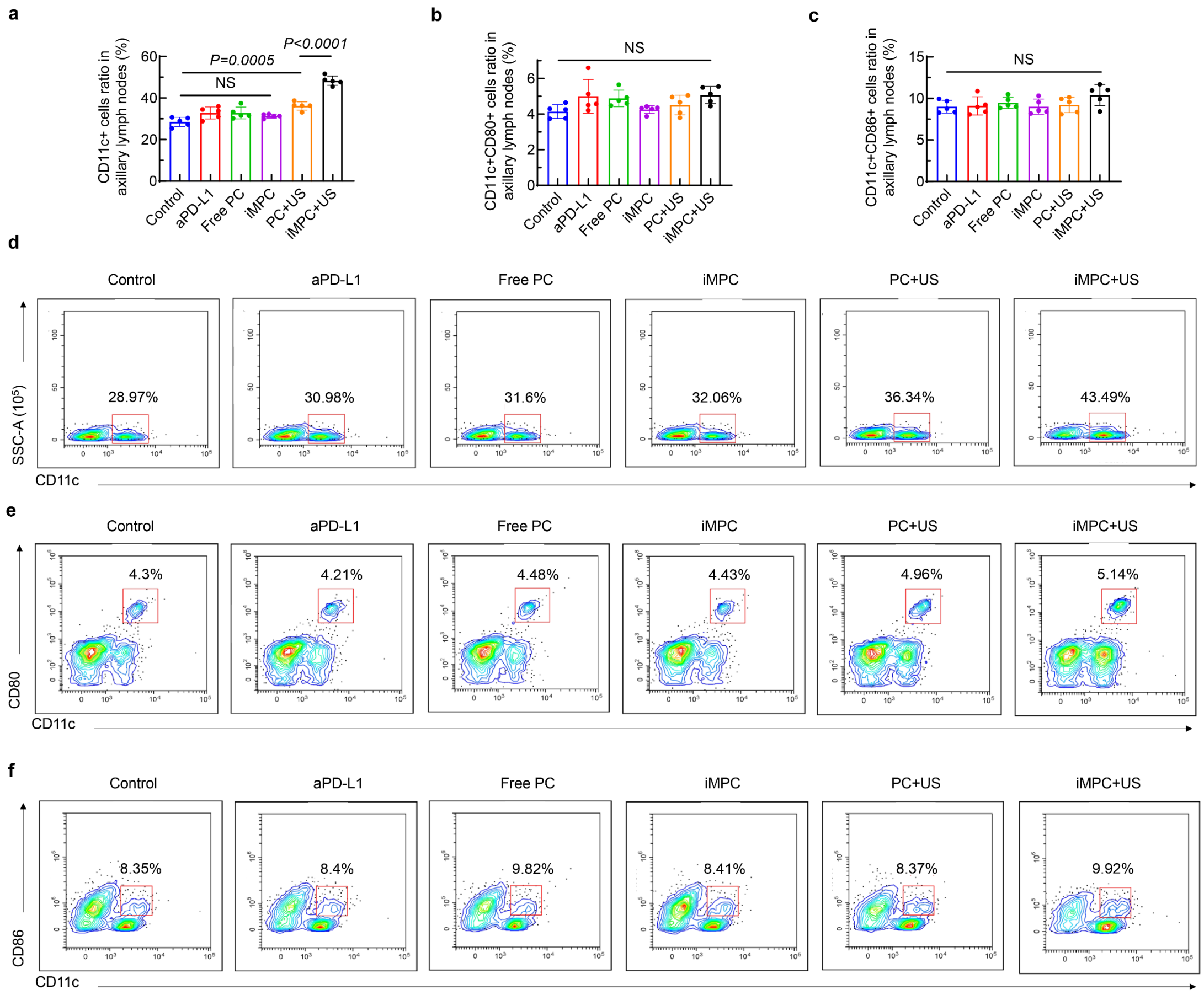

Supplementary Figure 28. The proportion of dendritic cells (DCs) in axillary lymph nodes increased. a, d, Proportion of DCs in axillary lymph nodes. **b, e,** Proportion of DCs highly expressing CD80 in axillary lymph nodes. **c, f,** Proportion of DCs highly expressing CD86 in axillary lymph nodes. n = 5 biologically independent mice per group for data in c-f and **a-c**. Data are expressed as the mean ± SD. Statistical significances were determined using one-way ANOVA with Fisher's LSD post-hoc test. Comparisons were performed between different groups in **a-c**. Not significant (NS) is $P \geq 0.05$, and significant P values are shown. Source data are provided as a Source Data file.

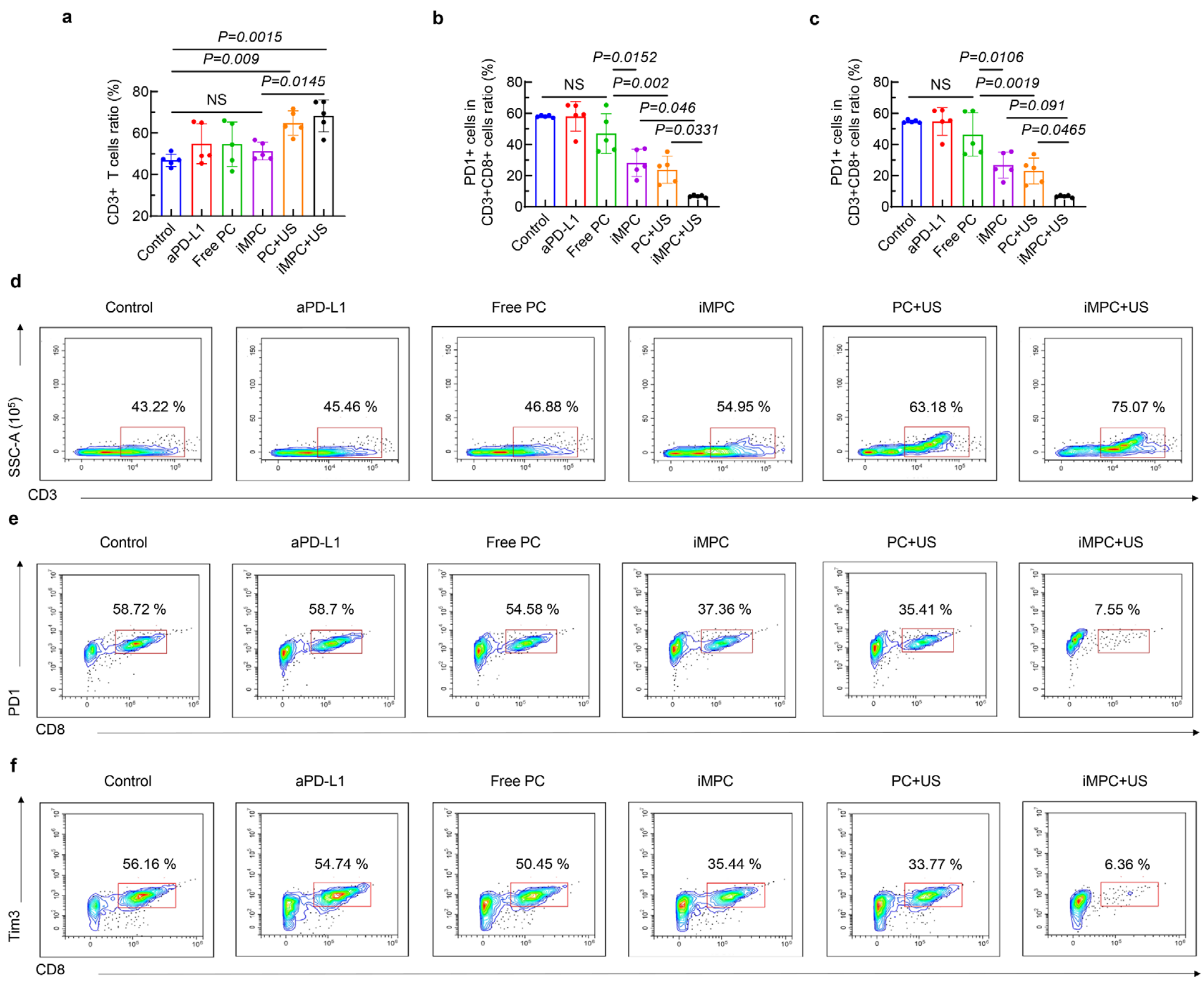

Supplementary Figure 29. The proportion of $CD3^+CD8^+$ T cells in deep cervical lymph nodes increased, with enhanced activity. a, d, Proportion of $CD3^+$ T cells in deep cervical lymph nodes. **b, e,** Proportion of PD1-expressing $CD3^+CD8^+$ T cells in deep cervical lymph nodes. **c, f,** Proportion of Tim3-expressing $CD3^+CD8^+$ T cells in deep cervical lymph nodes. n = 5 biologically independent mice per group for data in c-f and **a-c**. Data are expressed as the mean ± SD. Statistical significances were determined using one-way ANOVA with Fisher's LSD post-hoc test. Comparisons were performed between different groups in **a-c**. Not significant (NS) is $P \geq 0.05$, and significant P values are shown. Source data are provided as a Source Data file.

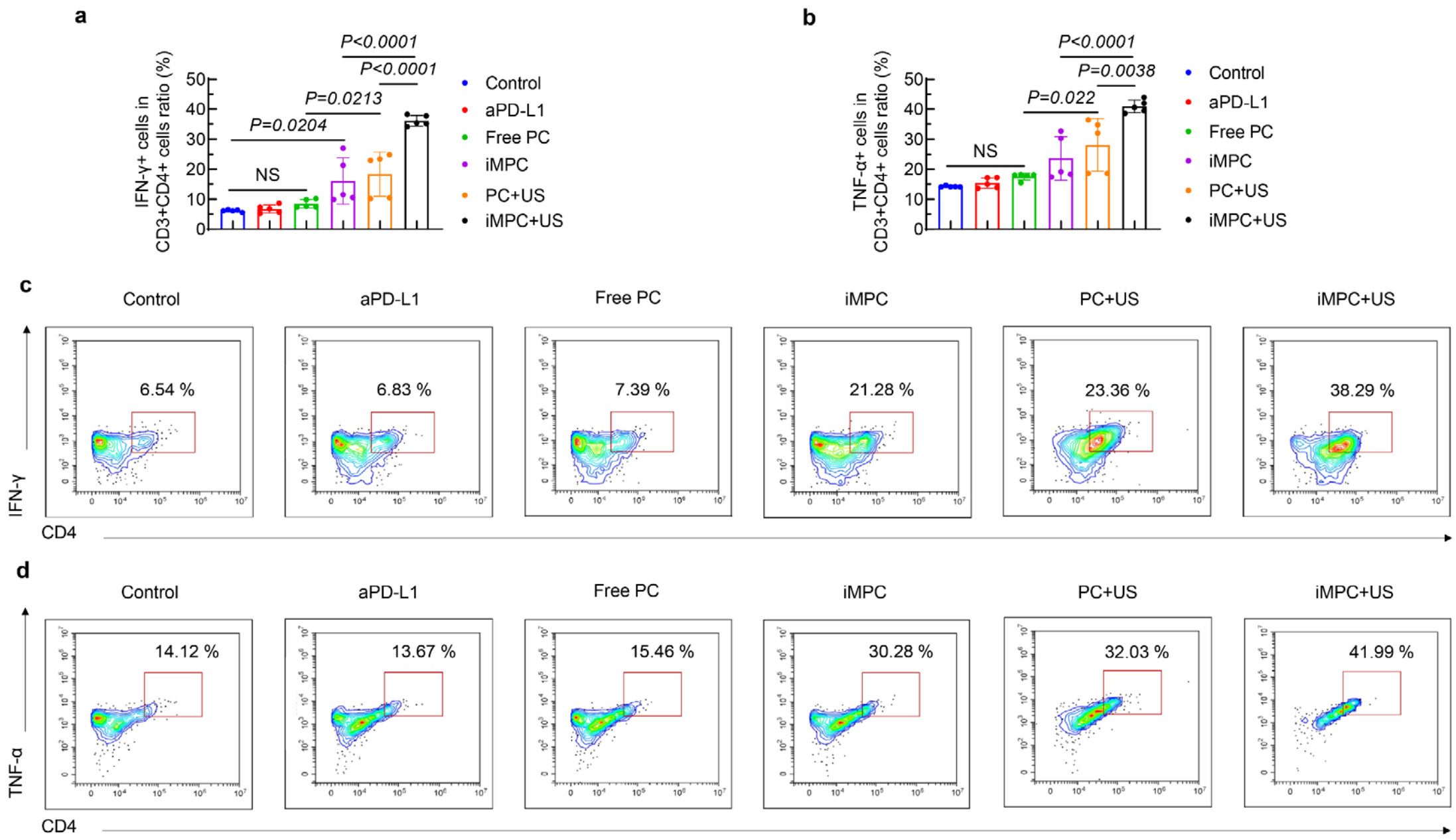

Supplementary Figure 30. The proportion of CD3$^+$CD4$^+$ T cells in deep cervical lymph nodes increased, with enhanced activity. a, c, Proportion of CD3$^+$CD4$^+$ T cells expressing IFN-γ in deep cervical lymph nodes. **b, d,** Proportion of CD3$^+$CD4$^+$ T cells expressing TNF-α in deep cervical lymph nodes. n = 5 biologically independent mice per group for data in c-f and **a-b**. Data are expressed as the mean ± SD. Statistical significances were determined using one-way ANOVA with Fisher's LSD post-hoc test. Comparisons were performed between different groups in **a-b**. Not significant (NS) is $P \geq 0.05$, and significant P values are shown. Source data are provided as a Source Data file.

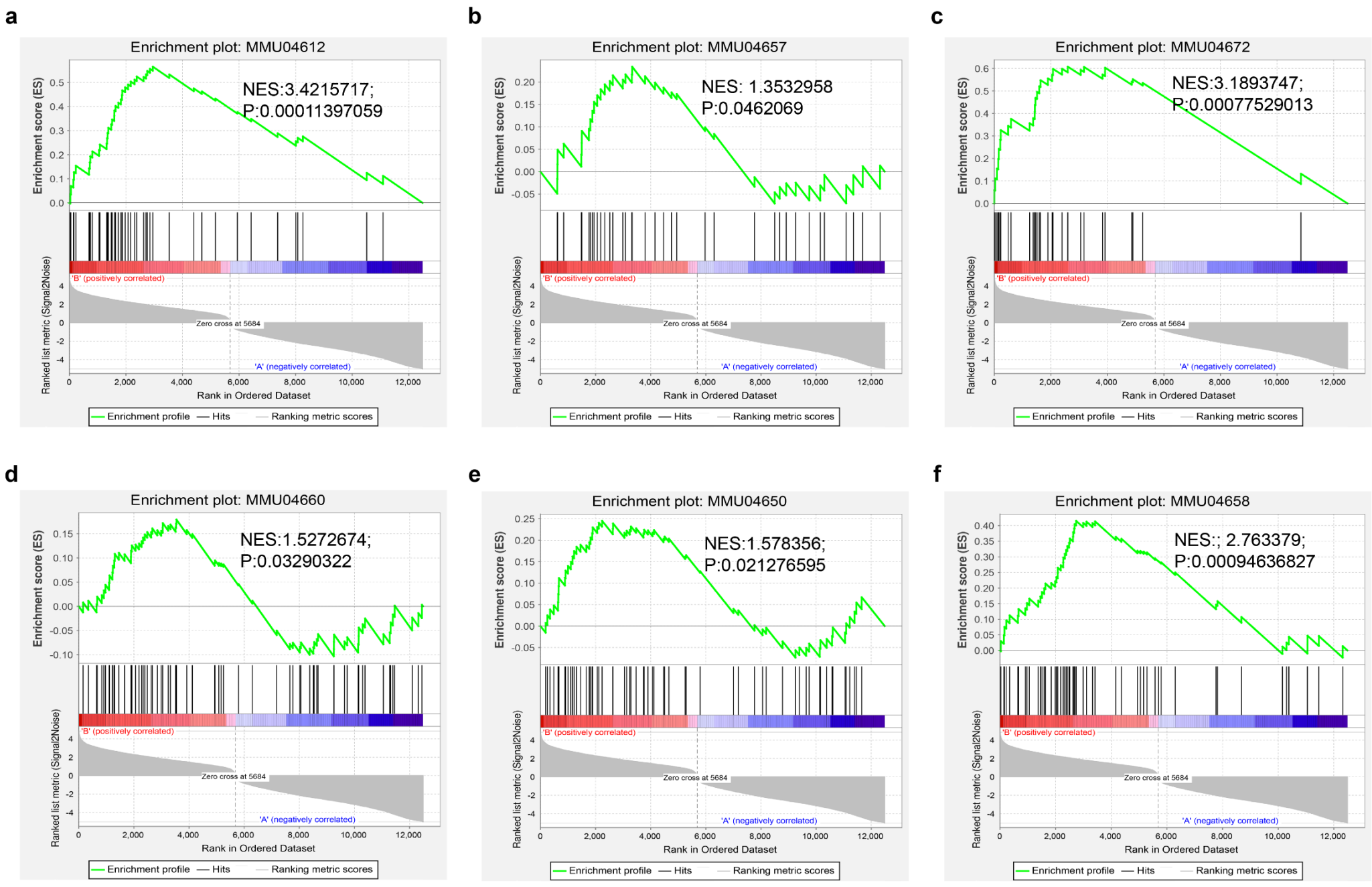

Supplementary Figure 31. Enrichment analysis of multiple immune signaling pathways for differentially expressed genes in tumor tissues from two treatment groups. a, mmu04612_Antigen processing and presentation. **b,** mmu04657_1L-17 signaling pathway. **c,** mmu04672 intestinal immune network for lgA production. **d,** mmu04660 T cell receptor signaling pathway. **e,** mmu04650 Natural killer cell mediated cytotoxicity. **f,** mmu04658 Thi and Th2 cell differentiation.

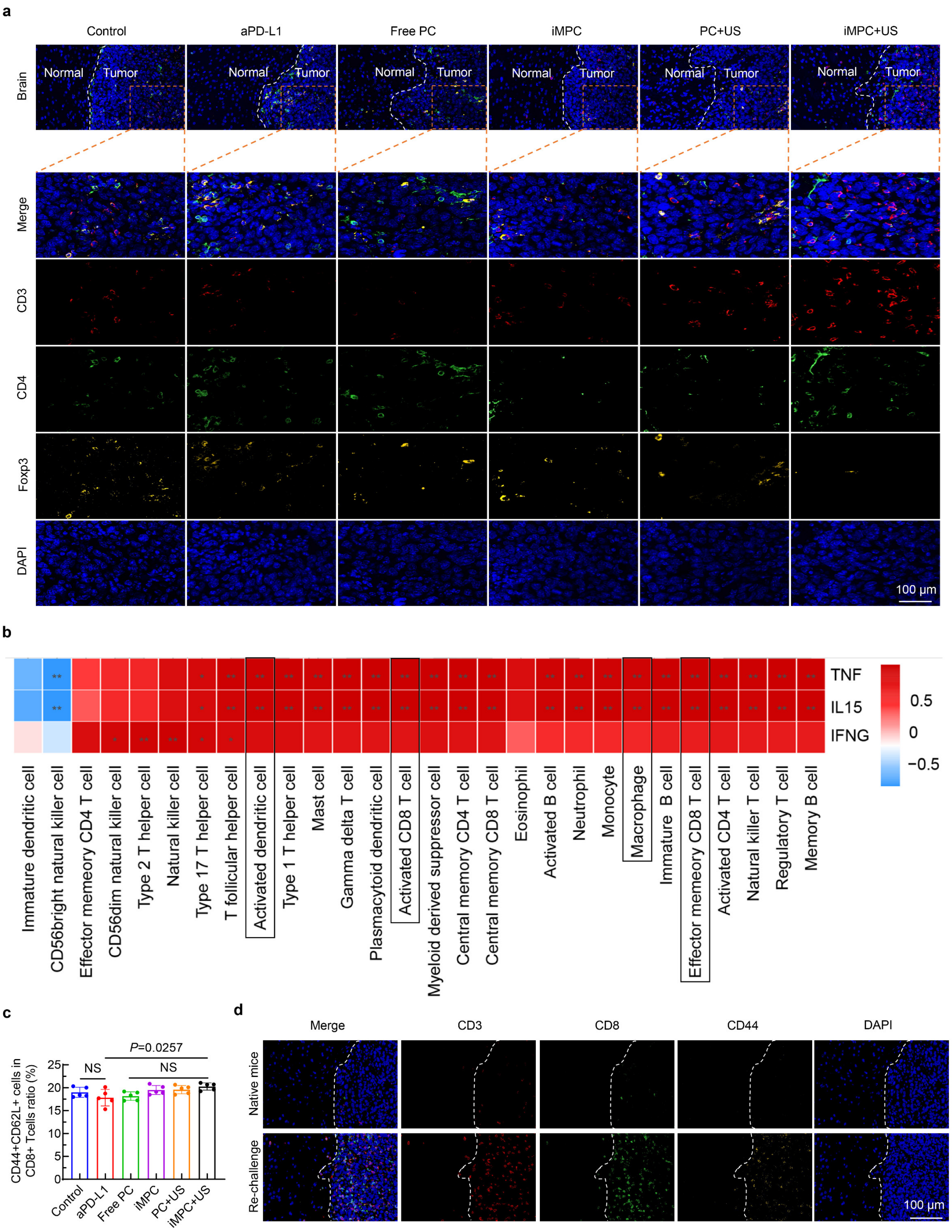

Supplementary Figure 32. Analysis of Treg cells and immune memory T cells infiltration. a, Analysis of Treg cell infiltration in tumor tissues (blue: DAPI; green: Alexa Fluor 488-CD4; red: CY3-CD3; yellow: SpRed-Foxp3). **b**, Correlation analysis between TNF-α, IFN-γ, IL-15, and various immune cells. **c,** Proportion of central memory T cells. **d**, Immunofluorescence analysis of memory T cell infiltration in tumor tissues (blue: DAPI; green: Alexa Fluor 488-CD8; red: CY3-CD3; yellow: SpRed-CD44). n = 5 biologically independent mice per group for data in **c**. Data are expressed

as the mean ± SD. Statistical significances were determined using one-way ANOVA with Fisher's LSD post-hoc test. Comparisons were performed between different groups in **c**. Not significant (NS) is $P \geq 0.05$, and significant P values are shown. Source data are provided as a Source Data file.

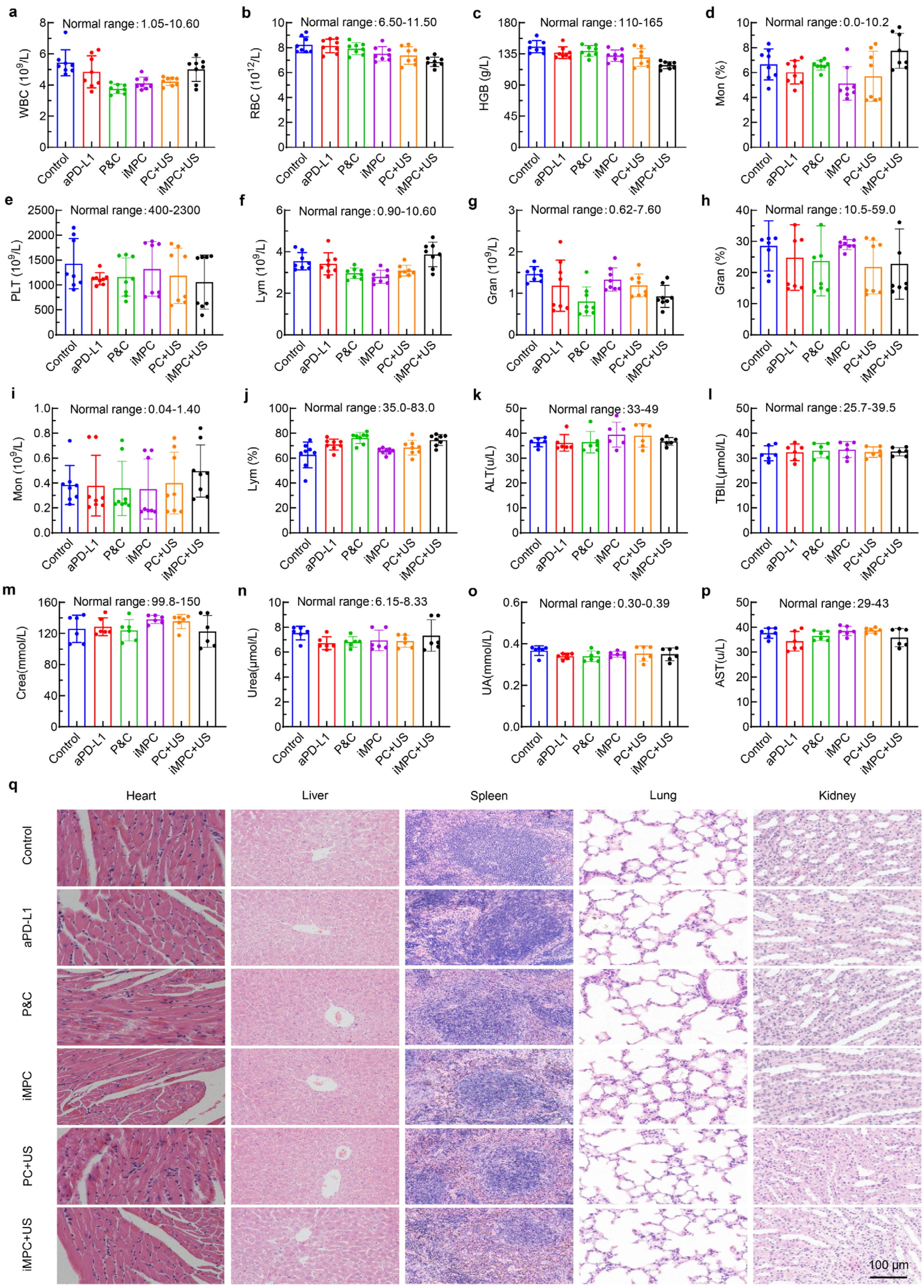

Supplementary Figure 33. Safety evaluation. a-j, Evaluation of blood counts. **k-p**, Blood biochemistry evaluation. **q**, HE staining of heart, liver, spleen, lung, and kidney tissues. n = 6 biologically independent mice per group for data in **a-p**. Data are expressed as the mean ± SD. Statistical significances were determined using one-way ANOVA with Fisher's LSD post-hoc test. Comparisons were performed between different groups in **a-p**. Not significant (NS) is $P \geq 0.05$, and significant P values are shown. Source data are provided as a Source Data file.

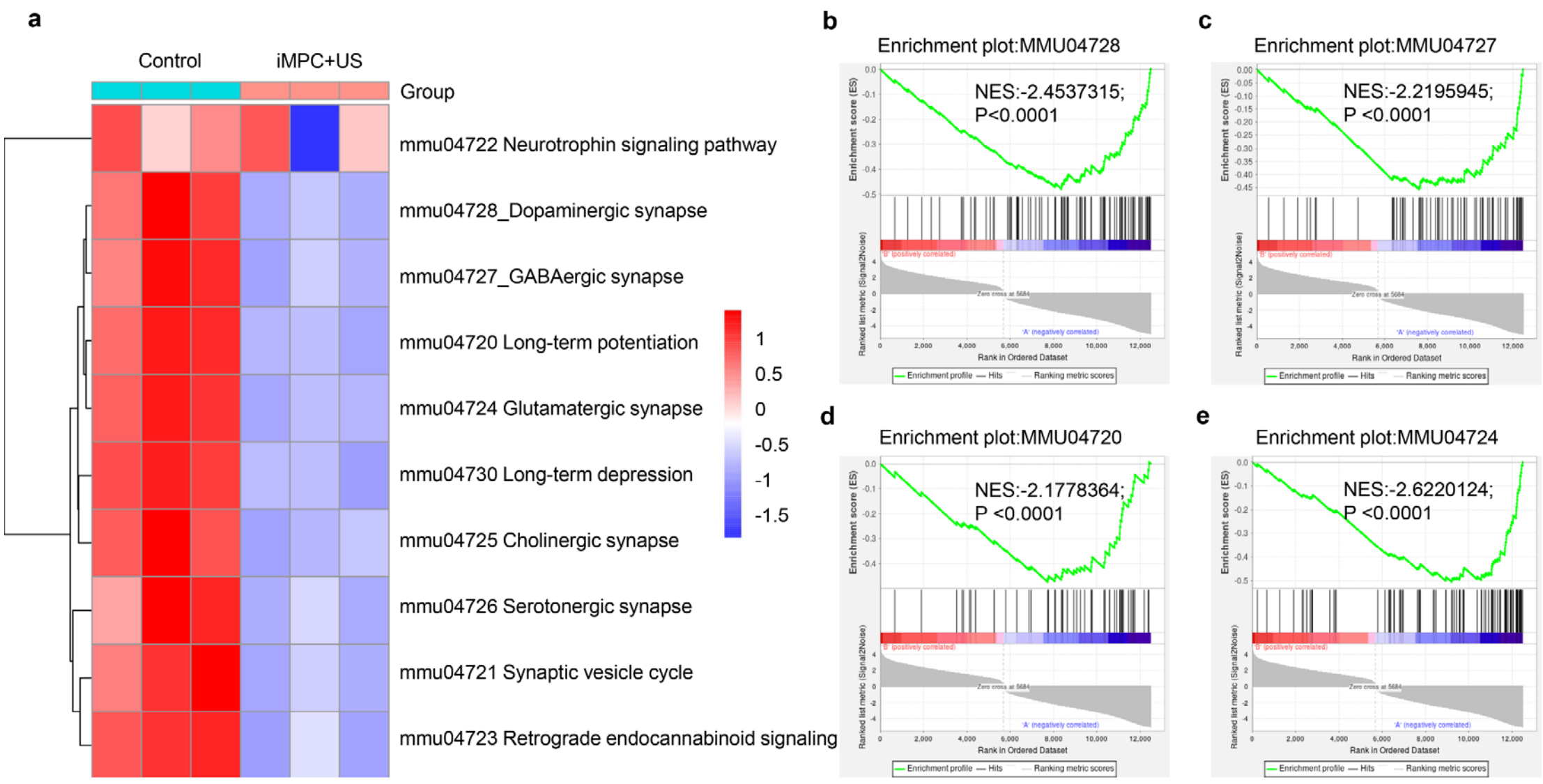

Supplementary Figure 34. Transcriptome sequencing analysis of differential genes. a, Enrichment analysis of multiple neural signaling pathways for differential genes in tumor tissues from two treatment groups. **b-e**, Combination of iMPC and LIPUS leads to the inhibition of multiple neural signaling pathways.

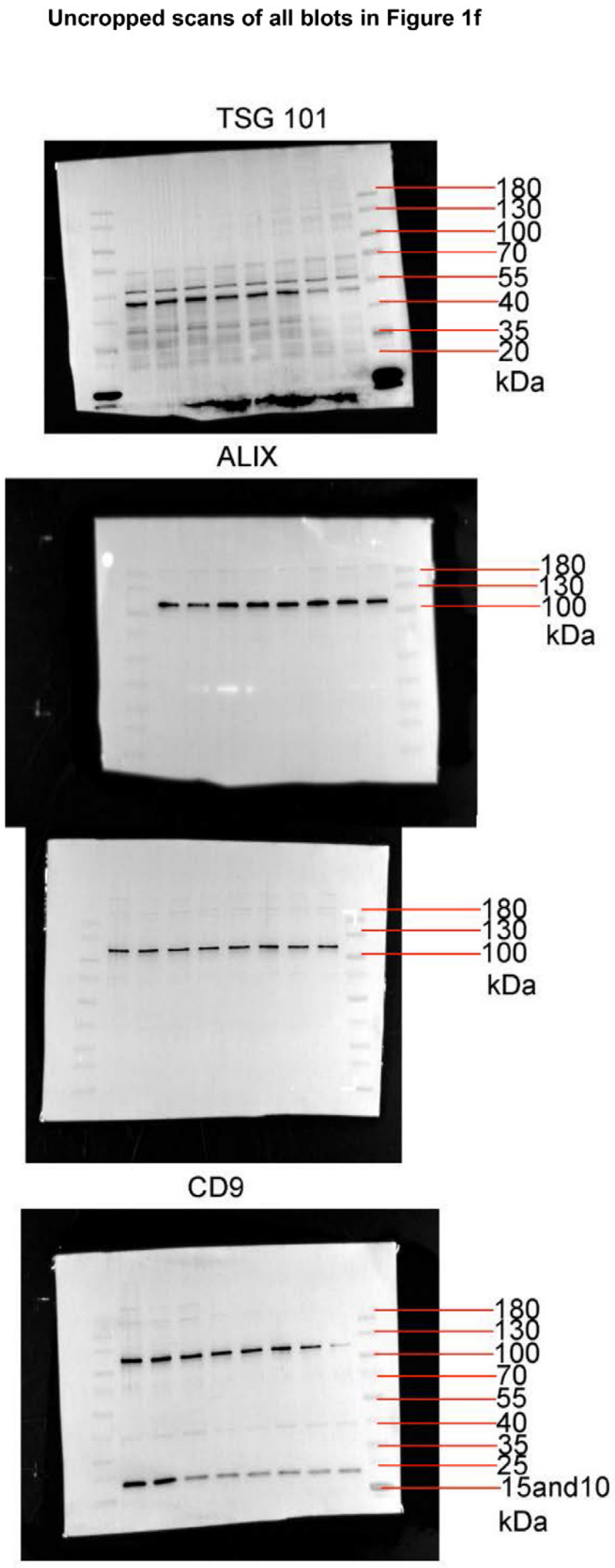

Supplementary Figure 35. Uncropped scans of all blots in Figure 1f.

Uncropped scans of all blots in Figure 4b, 4e

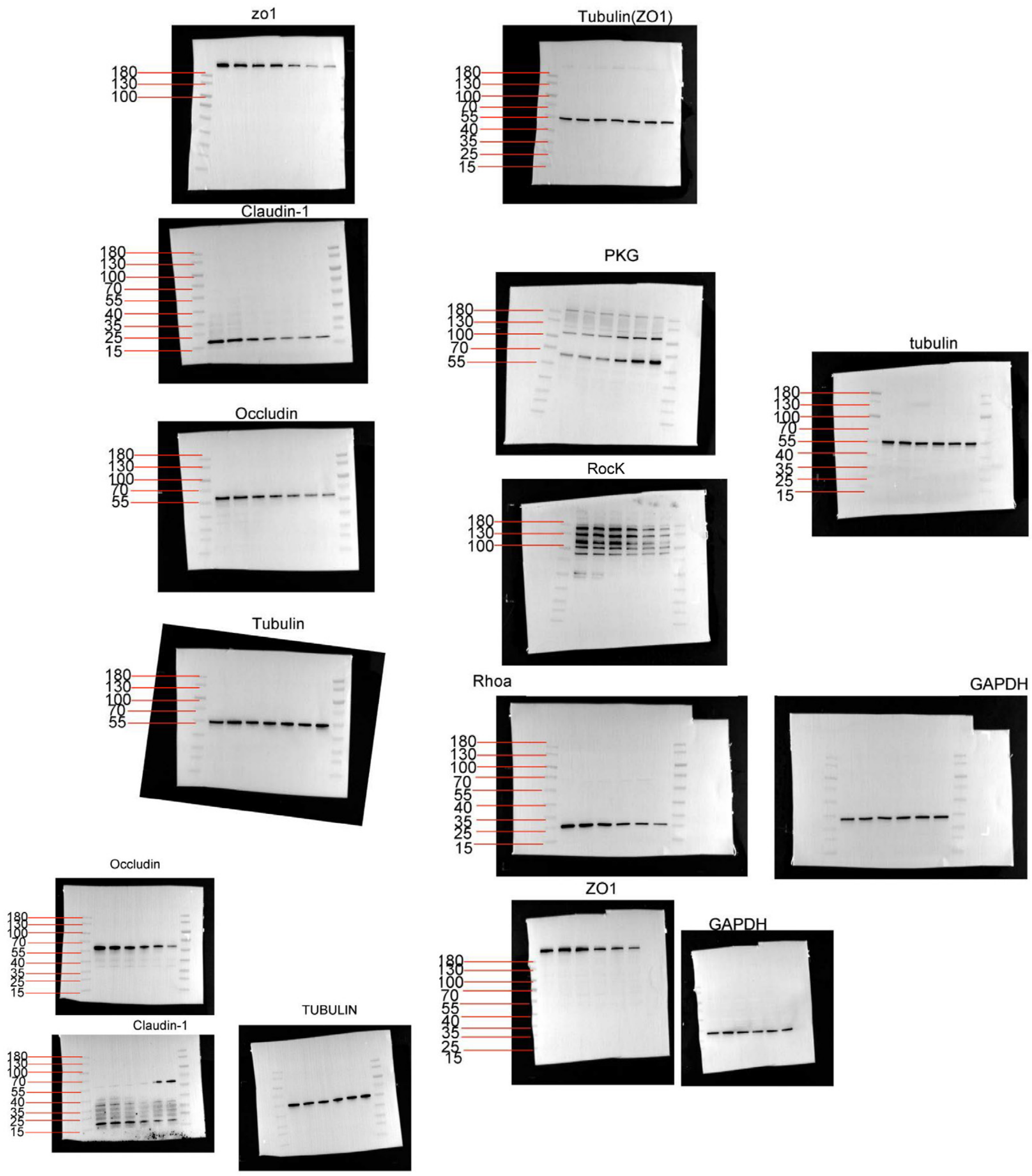

Supplementary Figure 36. Uncropped scans of all blots in Figure 4b, 4e.

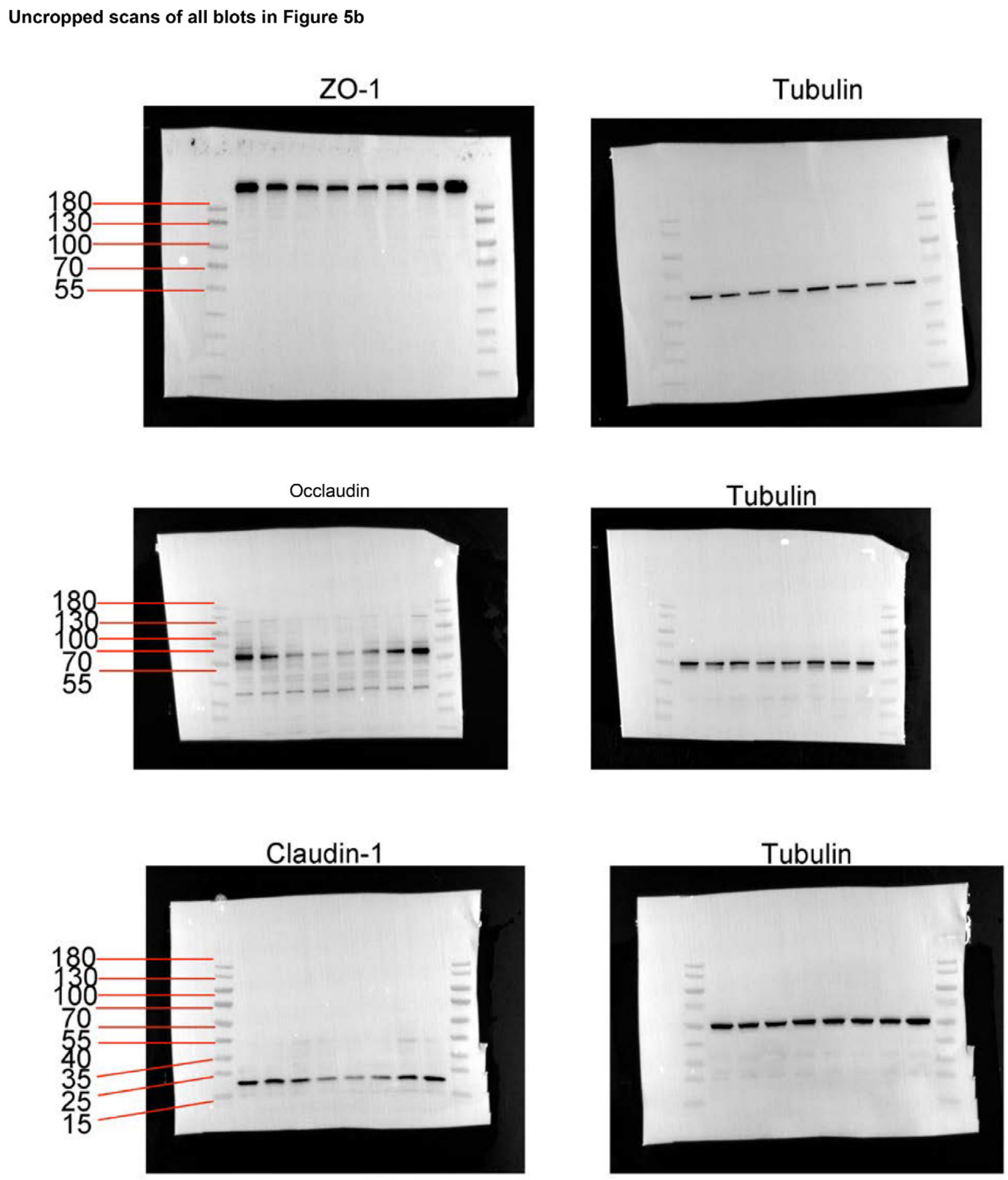

Supplementary Figure 37. Uncropped scans of all blots in Figure 5b.

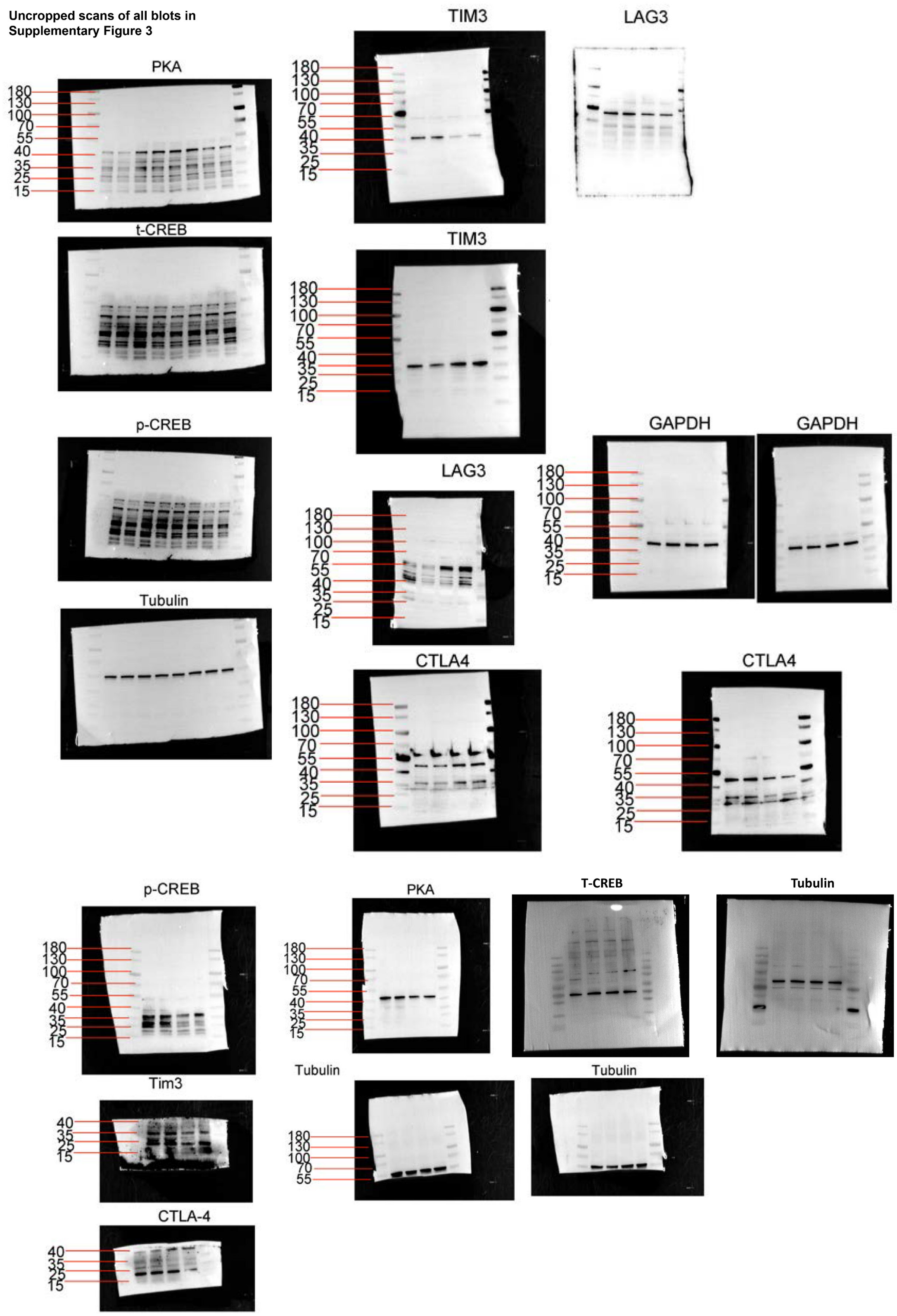

Supplementary Figure 38. Uncropped scans of all blots in Supplementary Figure 3.

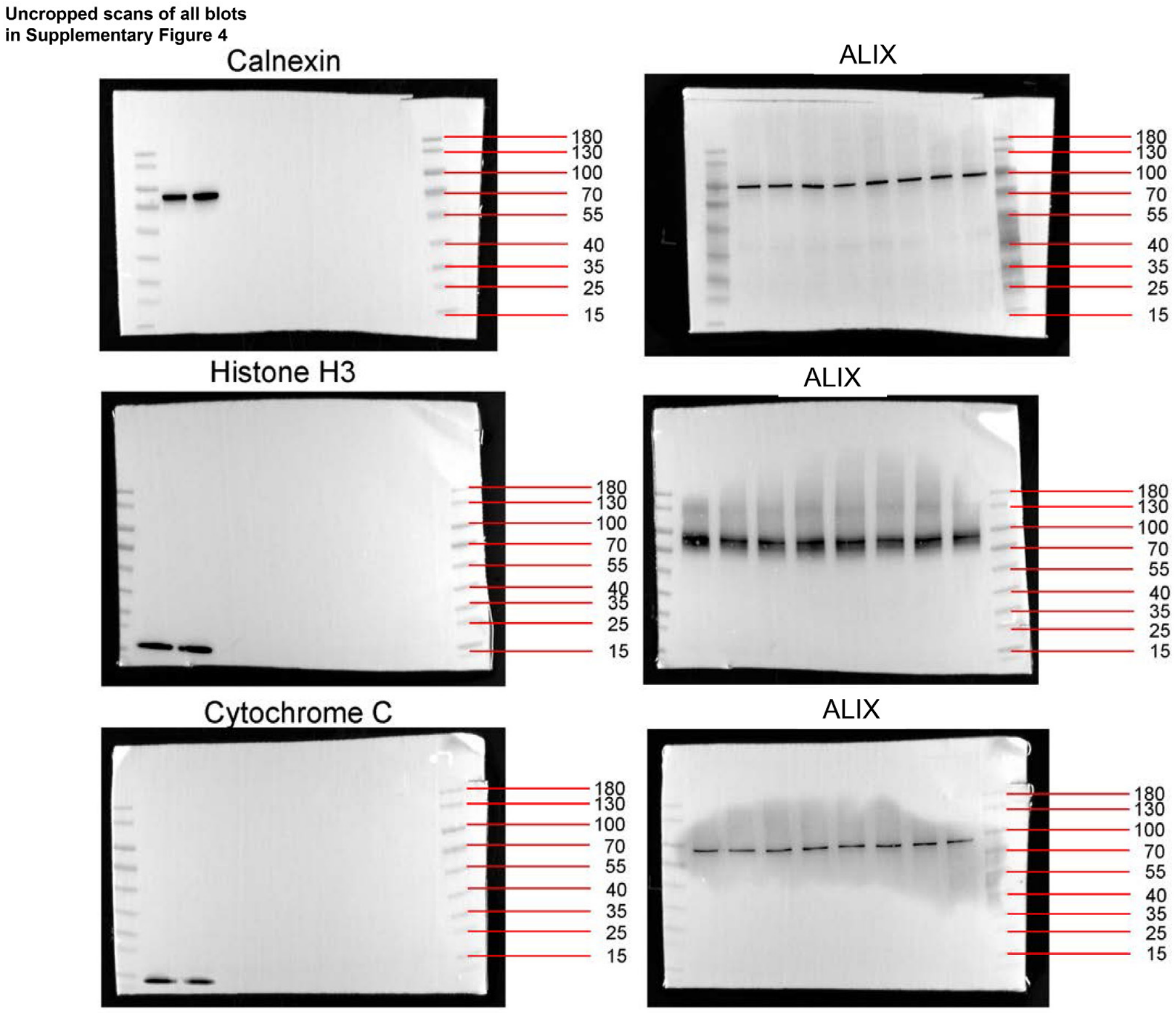

Supplementary Figure 39. Uncropped scans of all blots in Supplementary Figure 4.

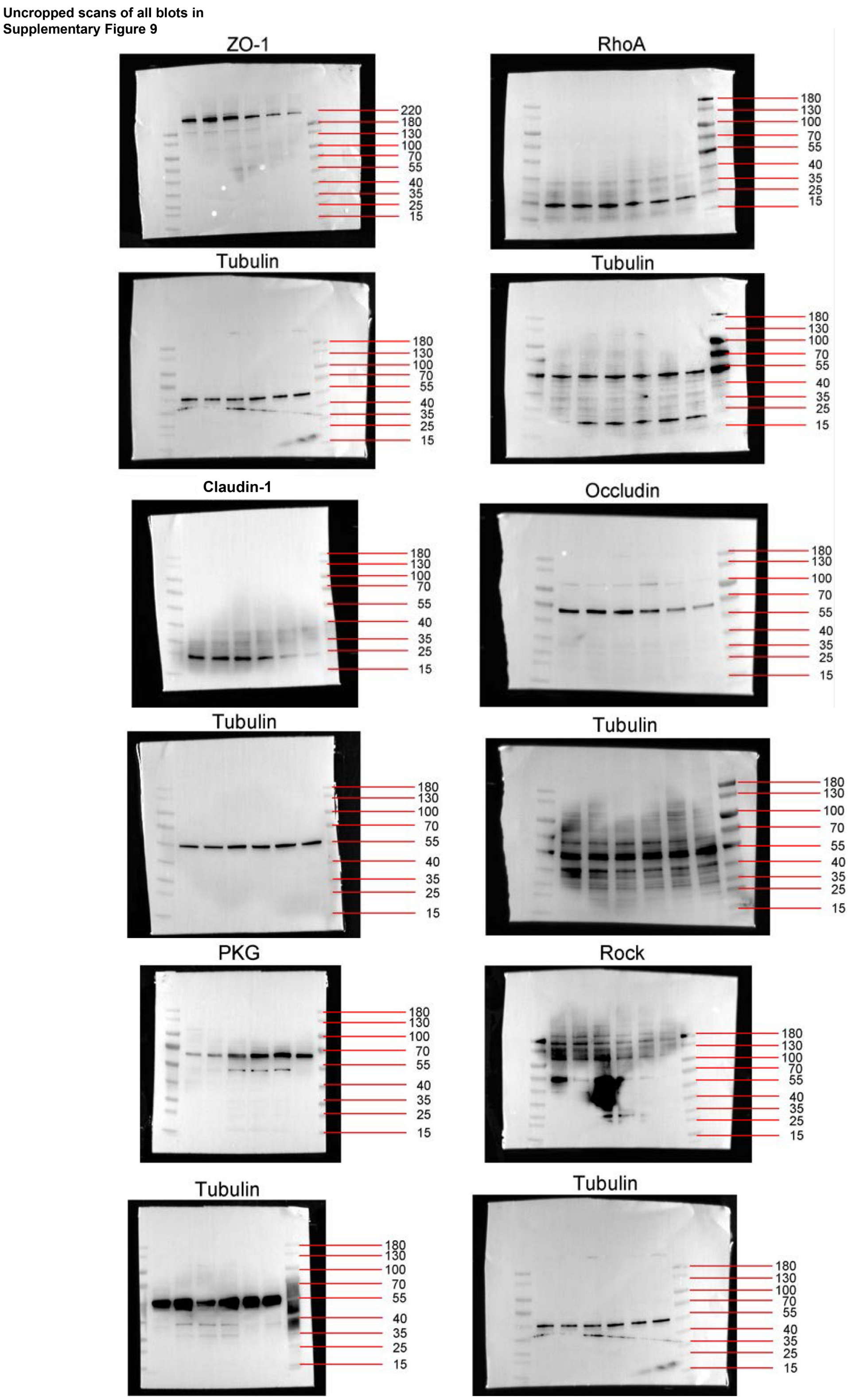

Supplementary Figure 40. Uncropped scans of all blots in Supplementary Figure 9.

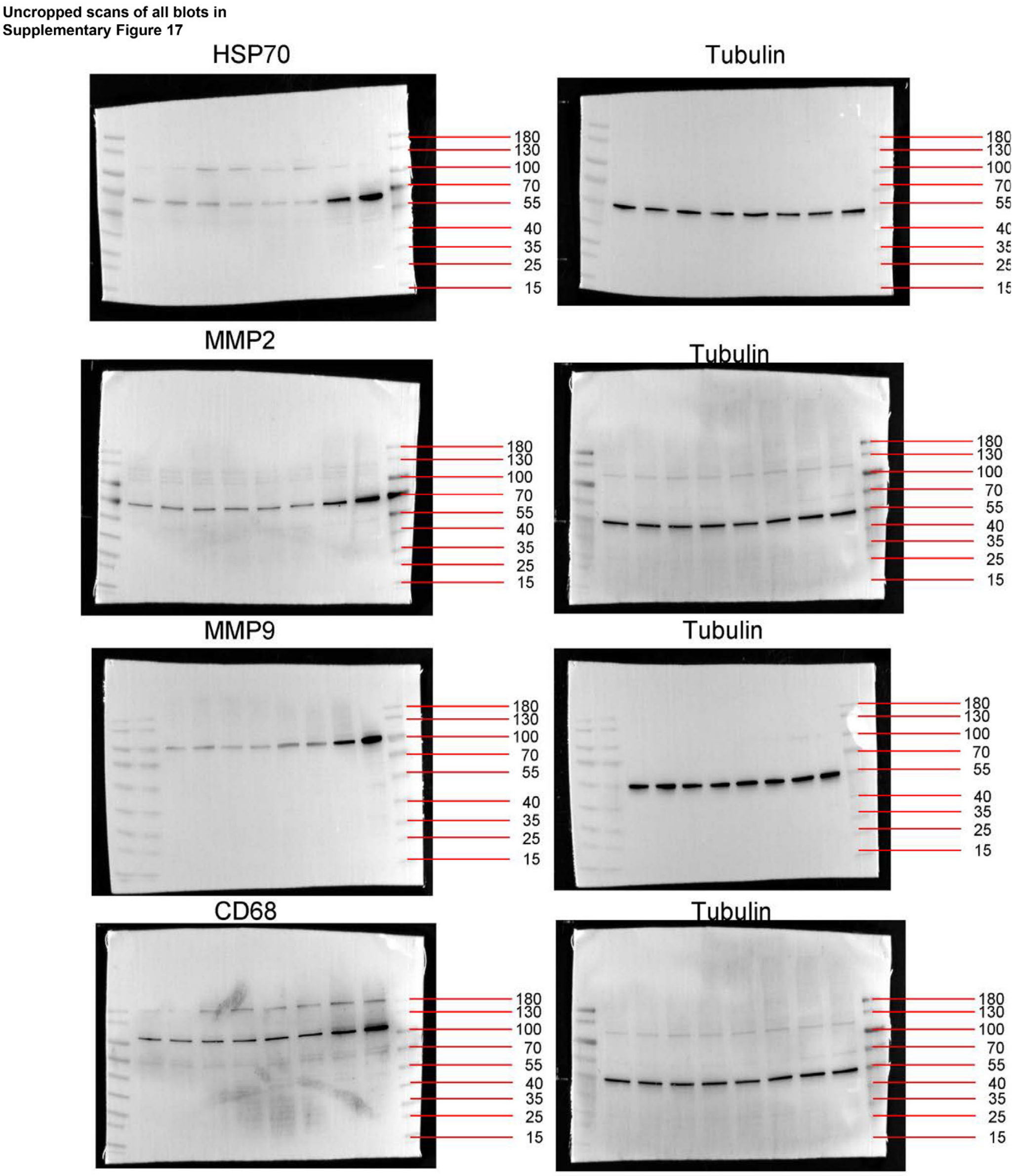

Supplementary Figure 41. Uncropped scans of all blots in Supplementary Figure 17.
