# Supplementary material for: Low-intensity pulsed ultrasound-mediated nose-to-brain co-delivery of β-blockers and aPD-L1 enhances glioblastoma immunotherapy
Source: Nat Commun. 2026 Jul 30;17:9254. doi: 10.1038/s41467-026-76103-4 (PMC13526793; doi:10.1038/s41467-026-76103-4)
Supplement: Supplementary file 2 [file 41467_2026_76103_MOESM2_ESM.pdf]

nature portfolio

Corresponding author(s): Jinbing Xie and Haijun Zhang

Last updated by author(s): Feb 26, 2026

Reporting Summary

Nature Portfolio wishes to improve the reproducibility of the work that we publish. This form provides structure for consistency and transparency in reporting. For further information on Nature Portfolio policies, see our Editorial Policies and the Editorial Policy Checklist.

Statistics

For all statistical analyses, confirm that the following items are present in the figure legend, table legend, main text, or Methods section.

n/a	Confirmed	
☐	☒	The exact sample size (*n*) for each experimental group/condition, given as a discrete number and unit of measurement
☐	☒	A statement on whether measurements were taken from distinct samples or whether the same sample was measured repeatedly
☐	☒	The statistical test(s) used AND whether they are one- or two-sided<br>*Only common tests should be described solely by name; describe more complex techniques in the Methods section.*
☐	☒	A description of all covariates tested
☐	☒	A description of any assumptions or corrections, such as tests of normality and adjustment for multiple comparisons
☐	☒	A full description of the statistical parameters including central tendency (e.g. means) or other basic estimates (e.g. regression coefficient) AND variation (e.g. standard deviation) or associated estimates of uncertainty (e.g. confidence intervals)
☐	☒	For null hypothesis testing, the test statistic (e.g. *F*, *t*, *r*) with confidence intervals, effect sizes, degrees of freedom and *P* value noted<br>*Give P values as exact values whenever suitable.*
☒	☐	For Bayesian analysis, information on the choice of priors and Markov chain Monte Carlo settings
☒	☐	For hierarchical and complex designs, identification of the appropriate level for tests and full reporting of outcomes
☐	☒	Estimates of effect sizes (e.g. Cohen's *d*, Pearson's *r*), indicating how they were calculated

Our web collection on statistics for biologists contains articles on many of the points above.

Software and code

Policy information about availability of computer code

Data collection	The size distribution and zeta potential was evaluated by dynamic light scattering measurements using a ZetaPlus (BeNano 1800 Zeta Pro, Dandong Bettersize™ Instruments Ltd., Dandong, China).Fluorescence intensity was measured using a multifunctional microplate reader (PerkinElmer, Boston, MA, USA). The morphology was observed by transmission electron microscopy (TEM; HITACHI FE-2000, Tokyo, Japan) and atomic force microscopy (AFM; Dimension Icon, Bruker, Billerica, MA, USA; tip model: ScanAsyst-Air). The gel image was taken by Molecular Imager Gel Doc XR+ System (BioRad, California, USA). The flow-cytometry data were obtained by FACS Celesta (Becton, Dickson and Company, USA) or CytoFLEX LX (Beckman Coulter Life Sciences, Indianapolis, USA); A microplate reader (Multiskan Go, Thermo Fisher Scientific, Massachusetts, USA) was used to measure the absorbance at different nm. Fluorescence images were detected by Laser Scanning Confocal Microscopy (Olympus FV3000, Tokyo, Japan) ; IVIS luminescence image was captured by in vivo imaging machine(Tanon ABL-X5, Shanghai, China); In vivo MR was performed using a 7.0-Tesla small animal MR scanner (Bruker Pharmascan, Ettlingen, Germany)
Data analysis	The data was presented by Graph Pad Prism 8.00 (Graph Pad Software Inc, LaJolla, CA, USA).

For manuscripts utilizing custom algorithms or software that are central to the research but not yet described in published literature, software must be made available to editors and reviewers. We strongly encourage code deposition in a community repository (e.g. GitHub). See the Nature Portfolio guidelines for submitting code & software for further information.

Data

Policy information about availability of data

All manuscripts must include a data availability statement. This statement should provide the following information, where applicable:

- Accession codes, unique identifiers, or web links for publicly available datasets
- A description of any restrictions on data availability
- For clinical datasets or third party data, please ensure that the statement adheres to our policy

All data supporting the findings of this study are available within the paper and its Supplementary Information files or from the corresponding author upon reasonable request. Source data are provided within this paper.

Research involving human participants, their data, or biological material

Policy information about studies with human participants or human data. See also policy information about sex, gender (identity/presentation), and sexual orientation and race, ethnicity and racism.

Reporting on sex and gender	In this study, the term sex (biological attribute) is used when referring to the classification of participants, as gender-related data were not collected. Participant sex was determined based on patient self-report at the time of clinical diagnosis. Both male and female patients with glioblastoma (GBM) were included in the study design; however, formal statistical analyses stratified by sex were not performed, as the primary aim was to characterize the tumor immune microenvironment (TIM) and T cell exhaustion profile in relation to carvedilol treatment, rather than to assess sex-specific differences in these outcomes.
Reporting on race, ethnicity, or other socially relevant groupings	Race and ethnicity data were not collected or analyzed in this study. All participants were of Han Chinese ethnicity, consistent with the population served by the participating center. No other socially relevant grouping variables were defined or used as proxies for socioeconomic status or other factors in the analyses. Confounding variables, including age at diagnosis, Karnofsky Performance Status (KPS), and extent of resection, were collected and considered in the study design; however, due to the limited sample size of the retrospective cohort, formal statistical adjustment for these variables was not performed in the primary multiplex immunofluorescence analyses.
Population characteristics	The study population consisted of adult patients (≥18 years of age) with histopathologically confirmed primary glioblastoma (GBM, WHO Grade IV) who underwent surgical resection at the participating center. Key participant characteristics included:Age: Median age at diagnosis was 59 years (range, 34–78 years).Treatment status: Patients were categorized into two groups based on whether they received carvedilol treatment as part of their clinical management prior to sample collection.Clinical covariates: Data on extent of resection (gross total resection vs. subtotal resection), use of standard adjuvant chemoradiotherapy (temozolomide + radiotherapy), and Karnofsky Performance Status (KPS) were collected from medical records.Molecular features: IDH1/2 mutation status and MGMT promoter methylation status were determined from clinical diagnostic reports where available.
Recruitment	This is a retrospective study using formalin-fixed paraffin-embedded (FFPE) glioblastoma (GBM) tumor samples stored in the institutional tissue bank. Participants were identified from a consecutive series of patients who underwent surgical resection at the participating center between 2018 and 2023 and for whom archived FFPE tumor tissue was available in tissue microarray (TMA) format. No additional recruitment procedures were performed. Potential biases include: (1) selection bias: the retrospective design limits the generalizability of findings to patients treated at a single tertiary care center; (2) attrition bias: only patients with available archival tissue were included, which may exclude cases with rapid clinical progression or limited tissue yield; (3) confounding by indication: carvedilol treatment was not randomly assigned, and its use may be correlated with other clinical variables (e.g., cardiovascular comorbidities) that could independently influence the tumor immune microenvironment. These biases were mitigated through detailed documentation of clinical covariates and transparent reporting of the study's limitations in the main manuscript.
Ethics oversight	All procedures involving human participants were approved by the Life Science Ethics Review Board of Zhengzhou University, under protocol numbers ZZUIRB2024-191. The study was conducted in accordance with the Declaration of Helsinki. Patient consent forms were obtained for all participants, or a formal waiver of consent was granted by the ethics committee for the use of anonymized retrospective archival tissue samples. All patient information was anonymized and de-identified prior to analysis to ensure confidentiality.

Note that full information on the approval of the study protocol must also be provided in the manuscript.

Field-specific reporting

Please select the one below that is the best fit for your research. If you are not sure, read the appropriate sections before making your selection.

☒ Life sciences ☐ Behavioural & social sciences ☐ Ecological, evolutionary & environmental sciences

For a reference copy of the document with all sections, see nature.com/documents/nr-reporting-summary-flat.pdf

Life sciences study design

All studies must disclose on these points even when the disclosure is negative.

Sample size	The sample size for each experiment is specified in the figure legend and ranges from 3 to 10.
Data exclusions	no data was excluded
Replication	All attempts at replication were successful
Randomization	All samples and organisms were randomly allocated into the experimental groups
Blinding	The investigators were blinded to group allocation during data collection and/or analysis.

Reporting for specific materials, systems and methods

We require information from authors about some types of materials, experimental systems and methods used in many studies. Here, indicate whether each material, system or method listed is relevant to your study. If you are not sure if a list item applies to your research, read the appropriate section before selecting a response.

Materials & experimental systems

n/a	Involved in the study
☐	☒ Antibodies
☐	☒ Eukaryotic cell lines
☒	☐ Palaeontology and archaeology
☐	☒ Animals and other organisms
☐	☒ Clinical data
☒	☐ Dual use research of concern
☒	☐ Plants

Methods

n/a	Involved in the study
☒	☐ ChIP-seq
☐	☒ Flow cytometry
☒	☐ MRI-based neuroimaging

Antibodies

Antibodies used	Anti-mouse PD-L1 was purchased from Bio X Cell (Cat. No. BE0101, New Hampshire, USA).<br>CD3 monoclonal antibody (Cat. No. 100204) and the antibodies used for flow cytometry were specific for Zombie NIR™ Fixable Viability Kit (Cat. No. 423106), CD8(Cat. No. 100732), IFN-γ(Cat. No. 430807), TNF-α(Cat. No. 986802), PD1(Cat. No. 143705), Tim3(Cat. No. 134011), CD3(Cat. No. 100204), CD4(Cat. No. 100537), CD8(Cat. No. 100732), PD1(Cat. No. 143705), IFN-γ(Cat. No. 430807), CD62L(Cat. No. 104407), CD44(Cat. No. 1030299), Foxp3(Cat. No. 4244011), were purchased from BioLegend (California, USA).<br>GAPDH (Cat. No. 60004-1-Ig) and the antibodies used for western blotting were Tubulin (Cat. No. 80762-1-RR), CREB (Cat. No. 67927-1-lg), phospho-CREB (28792-1-AP), LAG-3 (98167-1-RR), Tim-3 (82836-1-RR), CTLA-4 (30648-1-AP), claudin-1 (28674-1-AP), occludin (27260-1-AP), RhoA (66733-1-Ig), ZO-1 (21773-1-AP), were purchased from Proteintech (Wuhan, China).
Validation	All the antibodies used are from commercial sources and have been validated by the vendors.

Eukaryotic cell lines

Policy information about cell lines and Sex and Gender in Research

Cell line source(s)	The murine macrophage cell line RAW 264.7 (catalog: SCSP-5036), human neuroblastoma cell line SH-SY5Y (catalog: SCSP-5014), mouse brain endothelial cell line bEnd.3 (catalog: SCSP-5267), and mouse neuroblastoma cell line Neuro-2a (catalog: SCSP-5035) were obtained from the Chinese Academy of Science Cell Bank (Shanghai, China). The murine glioma cell line GL261 (catalog: SAc0135) was purchased from Shanghai Fusheng Industrial Co., Ltd. (Shanghai, China). Human nasal epithelial cell line HNEpC (IM?H279, IMMOCELL, Xiamen, Fujian, China); sex not specified. Murine glioma cell line G422 (catalog: IM-M041) were purchased from IMMOCELL (Xiamen, Fujian, China). The rat pheochromocytoma cell line PC12 (catalog: QuiCell-P305) was purchased from QuiCell (Shanghai, China). $CD8^+$ T cells were isolated using the EasySep™ Mouse $CD8^+$ T Cell Isolation Kit (catalog #19853, Stemcell Technologies, Vancouver, Canada). G1261-Luc cell was generated through infecting Lent-EFla-P2A-luciferase-CMV-coGFP-P2A-Purovirus (Banma Biotechnology Co., Ltd. BM2024012201) and selected with 2ug/ml puromycin(Beyotime, ST551-10mg). All cell lines were routinely treated with mycoplasma removing agent (VivaCell, Shanghai, China) and confirmed to be mycoplasma-negative. bEnd.3, GL261, GL261-Luc, Neuro-2a and G422 cells were cultured in high-glucose DMEM supplemented with 10% special grade fetal bovine serum. RAW 264.7, SH-SY5Y and PC12 cells were maintained in RPMI-1640 medium supplemented with 10% special grade fetal bovine serum. HNEpC cells were cultured in dedicated special medium for HNEpC. All cells were incubated at 37 °C in a humidified atmosphere containing 5% CO_2. Cells were cryopreserved using CELLSAVING serum-free cryopreservation solution (catalog: C40100, New Cell&Molecular Biotech, China). Fetal bovine serum (catalog: FBS-CS100) was purchased from Premium, NEWZERUM Ltd. (Shanghai, China).

Authentication	None of the cell lines used were authenticated.
Mycoplasma contamination	all the cell line are mycoplasma negative
Commonly misidentified lines (See ICLAC register)	No misidentified lines were used

Animals and other research organisms

Policy information about studies involving animals; ARRIVE guidelines recommended for reporting animal research, and Sex and Gender in Research

Laboratory animals	Male C57BL/6 mice (6–8 weeks old) were purchased from Jiangsu Huachuang Sino Pharma Tech Co., Ltd.
Wild animals	The study did not involve wild animals.
Reporting on sex	Gender preference analysis was not performed for ethical reasons and the study is gender-neutral
Field-collected samples	The study did not involve samples collected from the field
Ethics oversight	All animal experiments were conducted according to the ethical guidelines of the Animal Care & Welfare Committee of Southeast University, Jiangsu, China (NO. 20220316049).

Note that full information on the approval of the study protocol must also be provided in the manuscript.

Clinical data

Policy information about clinical studies

All manuscripts should comply with the ICMJE guidelines for publication of clinical research and a completed CONSORT checklist must be included with all submissions.

Clinical trial registration	ZZUIRB2024-191
Study protocol	The Life Science Ethics Review Board of Zhengzhou University
Data collection	We included retrospectively collected formalin-fixed paraffin-embedded (FFPE) tumor samples presented in tissue microarray (TMA) format. All tissues were used following approval by the Life Science Ethics Review Board of Zhengzhou University under protocol number ZZUIRB2024-191, which approved patient consent forms or, in certain cases, waiver of consent. This study did not pose additional therapeutic risks. All patient information was kept confidential.
Outcomes	Tumor tissues from GBM patients who were either subjected to or not subjected to carvedilol treatment were processed for multiplex fluorescence staining. Each section was stained with distinct and previously standardized multiplex immunofluorescence panels to profile exhausted T cells, including markers for DAPI, CD3, CD8, and PD-1 (blue: DAPI; green: Alexa Fluor 488-CD8; red: CY3-CD3; yellow: SpRed-PD1).

Plants

Seed stocks	*Report on the source of all seed stocks or other plant material used. If applicable, state the seed stock centre and catalogue number. If plant specimens were collected from the field, describe the collection location, date and sampling procedures.*
Novel plant genotypes	*Describe the methods by which all novel plant genotypes were produced. This includes those generated by transgenic approaches, gene editing, chemical/radiation-based mutagenesis and hybridization. For transgenic lines, describe the transformation method, the number of independent lines analyzed and the generation upon which experiments were performed. For gene-edited lines, describe the editor used, the endogenous sequence targeted for editing, the targeting guide RNA sequence (if applicable) and how the editor was applied.*
Authentication	*Describe any authentication procedures for each seed stock used or novel genotype generated. Describe any experiments used to assess the effect of a mutation and, where applicable, how potential secondary effects (e.g. second site T-DNA insertions, mosiacism, off-target gene editing) were examined.*

Flow Cytometry

Plots

Confirm that:

☒ The axis labels state the marker and fluorochrome used (e.g. CD4-FITC).

☒ The axis scales are clearly visible. Include numbers along axes only for bottom left plot of group (a 'group' is an analysis of identical markers).

☒ All plots are contour plots with outliers or pseudocolor plots.

☒ A numerical value for number of cells or percentage (with statistics) is provided.

Methodology

Sample preparation	For T cell analysis, tumor tissue cell suspensions were isolated using 0.5 μg/mL collagenase D (Roche, Basel, Switzerland), 0.5 μg/mL DNase I (Vazyme Biotec Co., Ltd, China), and 3 μg/mL DNase I (Sigma-Aldrich, USA). Brain-infiltrating immune cells were then separated using lymphocyte isolation solution (Solarbio, China). Cells were stained for 90 minutes with Zombie NIR™ Fixable Viability Kit, anti-CD3, anti-CD4, anti-CD8a, anti-PD1, anti-IFN-γ, anti-CD62L, anti-CD44 (423106, 100204, 100537, 100732, 143705, 430807, 104407, 103029; BioLegend, USA), and anti-Foxp3 antibody (126403; BioLegend, USA). Foxp3 staining was performed according to the BioLegend intracellular staining protocol (424401; BioLegend, USA). Subsequently, stained cells were subjected to flow cytometric analysis to identify $CD3^+CD8^+$ T cells, $PD1^+CD3^+CD8^+$ T cells, $CD3^+IFN\text{-}\gamma^+$ T cells, $CD3^+CD4^+$ T cells, $Foxp3^+CD3^+CD4^+$ T cells, $CD44^+CD62L^+$ T cells, and $CD44^+CD62L^-$ T cell populations ($2*10^4$ events were collected for analysis). All antibodies used for flow cytometric analysis were purchased from Dakewe Biotech Co., Ltd. Analytical grade chemicals were obtained from Sigma-Aldrich (USA).<br>Multiplex immunofluorescence staining was performed on tumor tissue sections. Quadruple staining (CD3, CD8, IFN-γ, and DAPI for nuclear labeling) was used to evaluate the activity and infiltration of $CD3^+CD8^+$ T cells. Triple staining (CD8, PD1, and DAPI for nuclear labeling) was applied to assess T cell exhaustion. Quadruple staining (CD3, CD4, Foxp3, and DAPI for nuclear labeling) was employed to evaluate Treg cell infiltration. Another quadruple staining (CD3, CD8, CD44, and DAPI for nuclear labeling) was conducted to assess memory T cell infiltration. Technical support for multicolor immunofluorescence was provided by Wuhan Ruisaiqi Biotechnology Co., Ltd.
Instrument	FACS Celesta, Becton, Dickinson and Company, New Jersey, USA
Software	FlowJo_v10.8.1
Cell population abundance	mouse tumor infiltrating tissue lymphocyte extraction kit was used during sample preparation, no sorting was performed
Gating strategy	As illustrated in Supplementary Figure 14, we first utilized FSC-A and SSC-A to gate the cells, followed by FSC-A and FSC-H for single-cell gating. Next, we applied live-dead gating to identify viable cells. For markers without obvious positive and negative population boundaries, a Fluorescence Minus One (FMO) control was employed to establish the boundaries between positive and negative populations.

☒ Tick this box to confirm that a figure exemplifying the gating strategy is provided in the Supplementary Information.
