# Supplementary material for: Low-intensity pulsed ultrasound-mediated nose-to-brain co-delivery of β-blockers and aPD-L1 enhances glioblastoma immunotherapy
Source: Nat Commun. 2026 Jul 30;17:9254. doi: 10.1038/s41467-026-76103-4 (PMC13526793; doi:10.1038/s41467-026-76103-4)
Supplement: Supplementary file 3 [file 41467_2026_76103_MOESM3_ESM.pdf]

nature portfolio

Peer Review File

Low-intensity pulsed ultrasound-mediated nose-to-brain co-delivery of β-blockers and aPD-L1 enhances glioblastoma immunotherapy

Corresponding Author: Professor Jinbing Xie

Parts of this Peer Review File have been redacted as indicated to remove third-party material.

This file contains all reviewer reports in order by version, followed by all author rebuttals in order by version.

Version 0:

Reviewer comments:

Reviewer #1

(Remarks to the Author)
In this article, the authors developed a low-frequency ultrasound (LFUS)-mediated intranasal delivery system for targeted glioblastoma (GBM) immunotherapy. A bionic pH-responsive nanovesicle (iMPC) was developed, composed of macrophage-derived extracellular vesicle membranes (EM) co-delivers carvedilol and aPD-L1. Overall, it is an interesting paper. However, some comparison and discussion still lack. Few parts should be further improved as follows.

1. The similarity is quite high (plagiarism check is 30%), the authors should reduce the similarity of this manuscript. As well as, what is the novelty of this work?
2. The authors should provide the pharmacokinetics and clearance data (in main organs) for the drugs post-delivery. What is the in vivo half-life of carvedilol and aPD-L1?
3. Did the authors examine whether activated $CD8^+$ T cells or dendritic cell maturation in lymph node?
4. For the biocompability, the authors should be considered to provide the cytotocixity of these materials in normal cell lines.
5. It seems that only one cell line GL261 was used in this study. The authors should repeat their key discoveries in another GBM cell lines.
6. The authors mentioned about thermal damage for using LFUS, but no quantitative thermal imaging was shown to exclude temperature-induced damage. Please provide the in vitro and in vivo images for thermal inducing at different irradiation power and time points. What is the key factor of iMPC that make it response to LFUS?
7. In Figure 5b, it seems that the authors take the different mouse's brain at different time points. It might be unconvinced due to the different responses with strange tissues in each mouse. The authors should be considered to provide the MRI images of live GBM-bearing mice at designated time points (before and after treatment), rather than from different animals.
8. Please add the scale bar for Figure 1e-upper, Figure 4l-n, Supplementary Figure 1k, Supplementary Figure 12a,c, and highlight the words of e to g in Figure 2 legends.
9. Please pay attention to the format of the reference (for example, only the first letter of the title is capitalized in reference 1, while first letters of every words are capitalized reference 3)

Reviewer #2

(Remarks to the Author)
The authors developed a formulation in which aPD-L1 antibody and carvedilol were encapsulated into extracellular vesicles derived from RAW cells, and attempted brain tumor treatment via ultrasound-enhanced nose-to-brain delivery. The study is carefully analyzed with a very sophisticated experimental design. I would like to request revisions on the following points:
• The rationale for using carvedilol in brain tumor therapy should be explained in the Abstract from the perspective of T-cell responses mediated by NE signaling.
• While it is understood that the fabricated nano-iMPCs exhibit sufficient therapeutic effects, encapsulation of aPD-L1 antibodies into nanoparticles is not commonly performed. Although the encapsulation may facilitate brain delivery,

appropriate release afterward is essential. Is there any evidence that release occurs before cellular internalization?
• It is unclear how polyaspartic acid is incorporated into the MPC and how it functions. In addition, while it is stated that nanoparticles disintegrate and release their contents under acidic conditions, the underlying mechanism remains unclear.
• The EVs collected from RAW cells (Fig. 1e) may be smaller than typical ones.
• In Fig. 1f, it is necessary to exclude the possibility of contamination by cellular components using markers such as Calnexin.
• In Fig. 1o, the data for the original EVs should also be included.
• In Fig. 2, high-intensity ultrasound appears to cause cell detachment. This phenomenon is unrelated to the decrease of TJ proteins and should be discussed separately. In other words, applying ultrasound at levels that induce cell detachment is not appropriate for in vitro experiments. Permeability assessment and TJ protein expression should be evaluated under conditions where a certain TEER value is maintained.
• The legend for Fig. 2g is missing.
• In Fig. 3a, the image appears to show the whole mouse body, but it is difficult to identify which regions correspond to which organs. Please modify the figure to make this clear.
• In Fig. 3, why is the permeability reduced at 3 W ultrasound intensity compared to 1.5 W? If TJ protein expression is further decreased, permeability should be more enhanced.
• In Fig. 4j, please explain why Claudin-1 expression decreases in the iMPC-only group.
• The superiority of iRGD peptide modification is not demonstrated in vivo. In addition, the MPC + US group is missing in Fig. S5.
• Fig. S10 appears to be important data showing that the reduction of barrier function is transient and reversible. Since reversibility is also claimed in the Abstract, it is recommended to move this figure to the main text.
• In the Methods section, the irradiation site of the ultrasound should be described in detail. Furthermore, 1 MHz is generally considered a standard ultrasound frequency, whereas “low-frequency ultrasound” usually refers to below 1 MHz. The terminology, including the title, should therefore be revised accordingly.

Reviewer #3

(Remarks to the Author)
Dong et al. and their team developed an immune-activating nanosystem for the intervention of glioblastoma (GBM). This therapeutic system was created by loading extracellular vesicles (EVs) with carvedilol and aPD-L1 through processes of freeze-drying and extrusion, followed by surface modification using the targeting peptide iRGD. The article emphasizes that overcoming the blood-brain barrier (BBB) using low-frequency ultrasound (LFUS) alone marks a significant advancement. This innovation simplifies the procedure and reduces the risks associated with using microbubbles. Below are some suggestions for improving the manuscript.

Majors:
1. There are numerous typos and unclear sentences throughout the text. The authors should conduct another round of proofreading to address these issues. Additionally, the article lacks clarity in scientific writing and requires significant improvement. Specific examples of these issues can be found in the minor comments provided.

2. The current title, ‘Low frequency ultrasound-mediated nose-to-brain drug delivery,’ is very general. The title needs to be modified/elaborated to provide the reader with a more detailed description of the proposed GBM intervention.

3. In the introduction, the authors don't mention the current challenges faced by the LFUS in the NMB application.

4. What is ‘NE is associated with T-cell exhaustion and worsened prognosis in the GBM mice stress model’? How was it established?

5. In the cell viability test (Fig. 2b), is there any result indicating that the lower cell viability was caused by LFUS+iMPC instead of LFUS alone? The authors switch the LFUS? The description of the cell line differs in the experimental section.

6. Fig. 2e and 2g reveal significant accumulation of aPD-L1 in the lower chamber, and Fig. 2h-k, which can be attributed to disruption of the tight junction. But the paper doesn't give mechanistic insights into the data shown in Figure 2. Elucidate more on the phenomenon.

7. The results in Fig. 3a-d exhibit high iMPC accumulation in the tumor and trigeminal nerves for groups treated with 1.5 W/cm2 compared to groups treated with 3 W/cm2, which is conflicting. Elucidate on the same. Moreover, Fig. 4b-k, 4l, 4n, and 4m reveal effective disruption of tight junction at an ultrasound power intensity of 3 W/cm2, but the same dose does not correlate with in vivo fluorescence imaging results (Fig. 3a-d). Elucidate on the contradictory phenomena. Also explain mechanotransduction (Ca^{2+} signaling) and Ca^2+-mediated RhoA/ROCK and myosin light chain kinase (MLCK) activation facilitated by ultrasound in epithelial cells, and cite appropriate scientific references to support your claims. Moreover, it is highly recommended to assess tight junction disruption by measuring trans-endothelial/epithelial electrical resistance (TEER).

8. The authors emphasize low-frequency ultrasound throughout the manuscript. What is the typical range for low-frequency ultrasound? What factors contribute to the varying results observed at different frequency ranges?

9. How did you conclude to start the treatment on the 10th day after inoculation of the cancer cells in mice? On what basis

was the treatment started? Justify with respect to tumor volume.

10. How can immunotherapy alone completely eradicate tumors within 20 days (Fig. 5d and 5k) as GBM is considered to be one of the most robust and recurring ones? Most research articles published so far achieve such high efficiency in GBM therapy only through combination therapy involving chemotherapy and/or hyperthermia and immunotherapy.

11. Why is there a 2-fold and 4-fold increase in TNF-α and IFN-γ for the iMPC+US group upon comparison with the control (Fig. 7c and 7d)? Since iMPC by itself is not toxic to cancer cells, it delivers immunoadjuvants. State the role of CD8+ and CD4+ T-cells in the elevated levels of TNF-α and IFN-γ. Explain with appropriate scientific reasons and cite supporting references.

Minors:

1. For better reading flow, please add the abbreviation for the blood-brain barrier (BBB) in the abstract.

2. In the abstract, you have written iMPC in brackets beside bionic nanovesicles without completely describing the final nanosystem. Include the term iMPC after clearly describing the proposed nanosystem for adequate comprehension of the reader.

3. In the introduction section, you have stated “LFUS may transiently loosen tight junctions of NMB via mechanical vibration, without requiring MBs-enhanced acoustic energy”, making the use of LFUS-mediated biological barrier disruption seem like a possibility rather than an affirmed technique that has been exploited in your research. Moreover, the cited references (3 and 7) do not support the claim of ultrasound-mediated disruption of biological barriers. Reference ‘3’ doesn’t address ultrasound, but reference ‘7’ supports the claim, stating that cavitation is facilitated by a chitosan (primer) solution upon exposure to ultrasound. Either change the statement or include appropriate references to support your claims regarding the disruption/loosening of NMB. Also elucidate in detail the underlying mechanism involved in ultrasound-induced loosening of NMB.

4. At line 66, please cite LFUS specifically for clinical trials related to brain diseases instead of referencing an entire review article.

5. At line 90, "The membrane structure"

6. At line 92, "studies have shown"

7. The mechanism involved in pH-responsive iMPC wasn’t stated to the fullest. Elaborate on the pH-responsive mechanism and clearly state which part of the EVs/Polyaspartic acid (PASP) molecule undergoes protonation under the acidic TME.

8. Figure 1 is not intuitive for readers. It would be helpful to add more labels, such as for epithelial cells, endothelial cells, cilia, and iMPC. The fence structure associated with the epithelial cells may be confusing; providing a similar schematic for Scheme 1, Fig. 1c, and Fig. 4a would be beneficial for readers.

9. Include a scale bar for the insert image in Fig. 1e (TEM of EVs). Additionally, the fig reveals the EVs to be sized at nearly 30 nm, whereas after loading with carvedilol and aPD-L1, there is a drastic increase in the final nanoparticle (iMPC), nearly 100 nm. A minor increase of 10-15nm in size is usually observed. But in your case, the difference is enormous, and the reasons for the same weren't explained. Give a scientific explanation of the same and clearly elucidate the influence posed by PASP, if any.

10. A rapid release of aPD-L1 (more than 50%) could be observed within the first 8 Hrs under acidic pH (Fig. 1l). In the field of drug delivery, nanosystems are designed and intended for a prolonged and sustained release. The proposed therapeutic system iMPC defeats the whole purpose as most of the drug would be cleared via the renal system and the first pass effect posed by the hepatic system. Defend the design of iMPC over rapid clearance and therapeutic efficiency.

11. The surface charge of the proposed nanovesicle system (iMPC) is positive, and the cell membrane of all cells is negatively charged. While cationic nanosystems prove to be effective for nose-to-brain delivery by enhancing retention in nasal mucus and the olfactory region, they can also strongly adhere/bind to the anterior respiratory epithelium, where most of the initial dose is deposited. Adsorption onto the anterior respiratory epithelium would increase the risk of off-targeting and accumulation in other tissues, with clearance by the renal and hepatic systems. Justify the concern about off-targeting by positively charged iMPC, which can reduce therapeutic efficiency with a possible risk of systemic toxicity by complement activation.

12. At line 276, iMPC "possesses"

13. At line 283, there is an incomplete sentence for the chambers.

14. Check the figure captions/legends for Fig. 2 and bold the characters from e through k. Mention the cells utilized in Fig. 2h-k both in the figure legends and in their explanation in the manuscript. Also, at line 316, the statement of the cell activity is not clear.

15. At line 358, do the authors mean 2e-g?

16. At line 383, 1 "hour"

17. In the efficiency section, the mix of delivery efficiency and radiant efficiency is confusing. Additionally, what's the definition of retention?

18. At line 406, is “sound intensities” a correct use?

19. The authors do not need to define abbreviations repeatedly. For instance, at line 477, it is not necessary to spell out the full names of LFUS and NMB, as these have already been explained in earlier sections. Please review the entire manuscript for consistency.

20. At line 417, add a space after a period.

21. At line 699, "ELISA"

22. At line 770, "Statistical" and between "the" two

23. At line 892, "anti-tumor"

24. At line 923, while "minimizing"

25. At line 1106, "," where

Reviewer #4

(Remarks to the Author)
I co-reviewed this manuscript with one of the reviewers who provided the listed reports. This is part of the Nature Communications initiative to facilitate training in peer review and to provide appropriate recognition for Early Career Researchers who co-review manuscripts.

Version 1:

Reviewer comments:

Reviewer #1

(Remarks to the Author)
The authors have properly addressed all concerns with revisions. It is suggested to be accepted for publication.

Reviewer #2

(Remarks to the Author)
The manuscript has been sufficiently revised and is now suitable for publication

Reviewer #3

(Remarks to the Author)
The authors have addressed most of the comments; however, there are still some typographical errors that need revision:

1. The title contains a grammatical error.
2. Please ensure that the terms "claudin-1" and "occludin" are consistently written either in lowercase or capitalized throughout the entire article.
3. In Scheme 1, is it intended to be "LFPUS"?
4. In line 115, "two-hour" should be used in the singular form.
5. There is a grammatical error in line 446.
6. In lines 623 and 630, "ultrasound" should be written with a lowercase "u."
7. In line 739, the authors mention "NMB" twice.

Reviewer #4

(Remarks to the Author)
I co-reviewed this manuscript with one of the reviewers who provided the listed reports. This is part of the Nature Communications initiative to facilitate training in peer review and to provide appropriate recognition for Early Career Researchers who co-review manuscripts.

Open Access This Peer Review File is licensed under a Creative Commons Attribution 4.0 International License, which permits use, sharing, adaptation, distribution and reproduction in any medium or format, as long as you give appropriate credit to the original author(s) and the source, provide a link to the Creative Commons license, and indicate if changes were made.
In cases where reviewers are anonymous, credit should be given to 'Anonymous Referee' and the source.
The images or other third party material in this Peer Review File are included in the article's Creative Commons license, unless indicated otherwise in a credit line to the material. If material is not included in the article's Creative Commons license and your intended use is not permitted by statutory regulation or exceeds the permitted use, you will need to obtain permission directly from the copyright holder.
To view a copy of this license, visit https://creativecommons.org/licenses/by/4.0/

REVIEWER COMMENTS

Reviewer #1 (Remarks to the Author):

In this article, the authors developed a low-frequency ultrasound (LFUS)-mediated intranasal delivery system for targeted glioblastoma (GBM) immunotherapy. A bionic pH-responsive nanovesicle (iMPC) was developed, composed of macrophage-derived extracellular vesicle membranes (EM) co-delivers carvedilol and aPD-L1. Overall, it is an interesting paper. However, some comparison and discussion still lack. Few parts should be further improved as follows.

1. The similarity is quite high (plagiarism check is 30%), the authors should reduce the similarity of this manuscript. As well as, what is the novelty of this work?

Re: Thank you for your suggestions. We have revised the entire manuscript to reduce redundancy.

The primary novelty of this work lies in the following: Unlike previous approaches combining ultrasound (US) with microbubbles (MBs) to open the blood-brain barrier (BBB) and enhance drug penetration into the brain, this study pioneers the use of low-intensity pulsed ultrasound (LIPUS) to open the nasal mucosal barrier (NMB), thereby enhancing nasal-to-brain drug delivery. Nasal-brain drug delivery offers advantages by bypassing the BBB and reducing hepatic and renal toxicity.

References

1. Qiu, Y. et al. The Nasal-Brain Drug Delivery Route: Mechanisms and Applications to Central Nervous System Diseases. MedComm 6, e70213 (2025).

2. Guo, J. et al. Bio-barrier-adaptable biomimetic nanomedicines combined with ultrasound for enhanced cancer therapy. Signal Transduct Target Ther 10, 137 (2025).

3. Chen, Y. et al. Intranasal drug delivery: The interaction between nanoparticles and the nose-to-brain pathway. Adv. Drug Del. Rev. 207, 115196 (2024).

4. Shen, H. et al. Engineered commensals for targeted nose-to-brain drug delivery. Cell 188, 1545-1562.e16 (2025).

5. Wang, J. et al. Ultrasound-mediated blood-brain barrier opening: An effective drug delivery system for theranostics of brain diseases. Adv. Drug Del. Rev. 190, 114539 (2022).

6. Dong, L. et al. Open-Source Throttling of CD8+ T Cells in Brain with Low-Intensity Focused Ultrasound-Guided Sequential Delivery of CXCL10, IL-2, and aPD-L1 for Glioblastoma Immunotherapy. Advanced materials (Deerfield Beach, Fla.) 36, e2407235 (2024).

2. The authors should provide the pharmacokinetics and clearance data (in main organs) for the drugs post-delivery. What is the in vivo half-life of carvedilol and aPD-L1?

Re: Thank you for your valuable suggestions. We have supplemented the pharmacokinetic data following administration and clearance rates in major organs (liver, kidneys, and lungs). The *in vivo* half-lives of carvedilol and aPD-L1 are 6 hours and 36 hours, respectively.

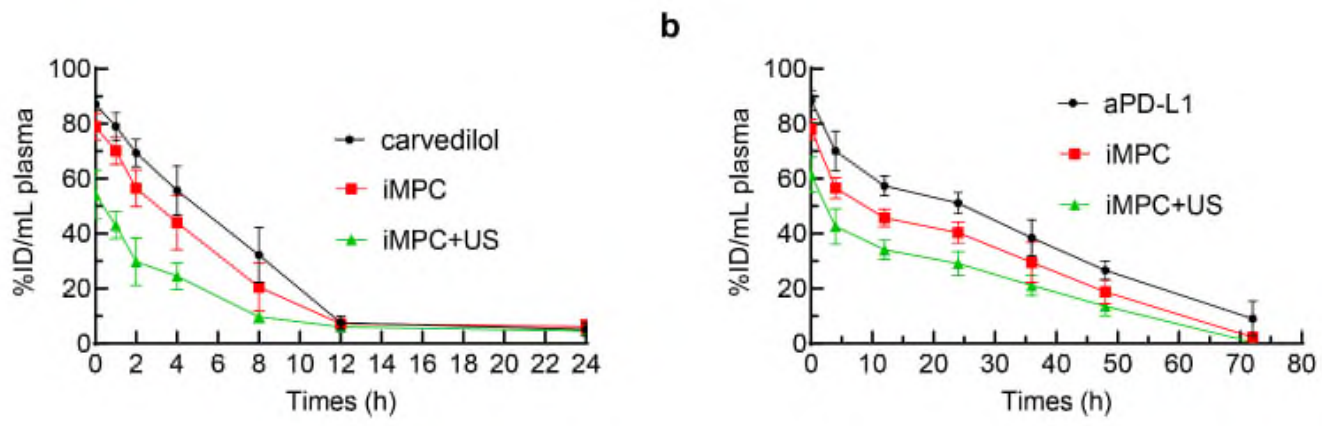

Supplementary Figure 14. Pharmacokinetic monitoring of carvedilol, aPD-L1, and iMPC nano-formulations. a. Percentage change in blood concentration of carvedilol. **b.** Percentage change in blood concentration of aPD-L1. n = 3 biologically independent mice per group for data in **a-b**. Data are expressed as the mean ± s.d.

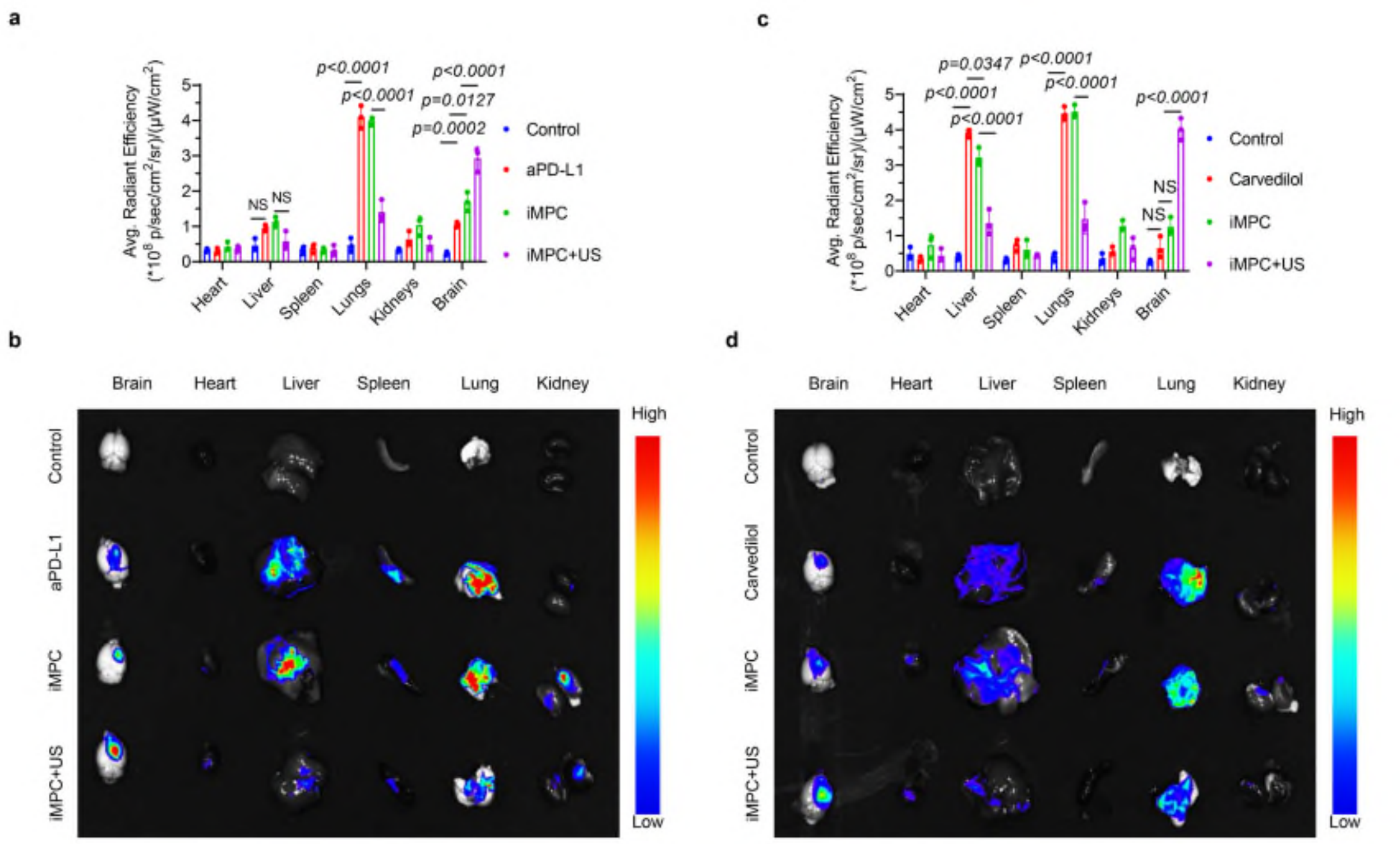

Supplementary Figure 15. Organ distribution of carvedilol, aPD-L1, and iMPC nano-formulations. a-b, Distribution of aPD-L1 and iMPC nano-formulations in heart, liver, spleen, lung,

and kidney. **c-d,** Distribution of carvedilol and iMPC nano-formulations in heart, liver, spleen, lung, and kidney. n = 3 biologically independent mice per group for data in **a** and **c**. Data are expressed as the mean ± s.d. Statistical significances were determined using one-way ANOVA with Dunnett's post hoc test. Comparisons were performed between different groups in **a** and **c**. Not significant (NS) is $P \geq 0.05$, and significant P values are shown.

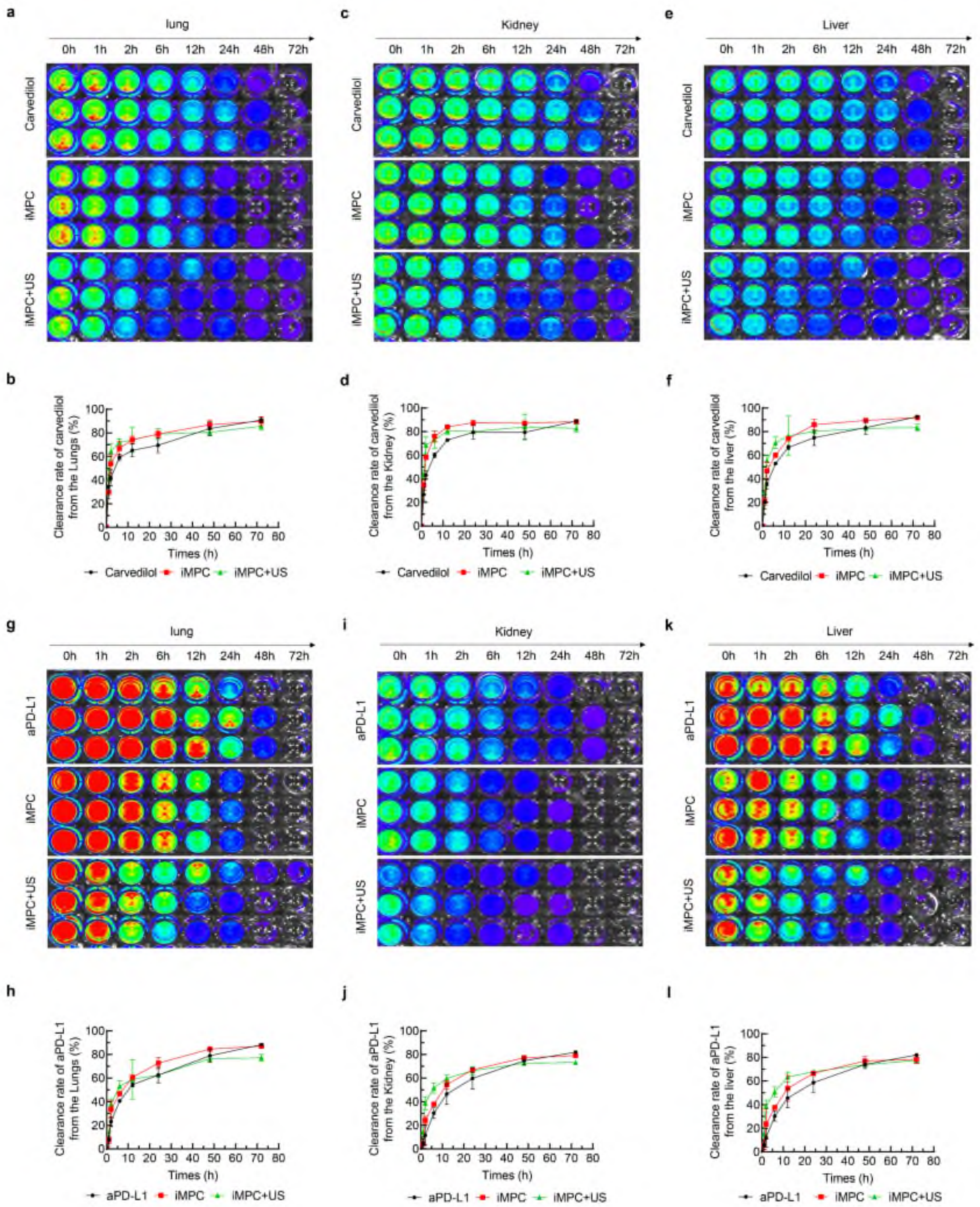

Supplementary Figure 16. Cardiac clearance of carvedilol, aPD-L1, and the iMPC nano-formulations. a-b, Pulmonary clearance of carvedilol and the iMPC nano-formulations. **c-d,** Renal clearance of carvedilol and the iMPC nano-formulations. **e-f,** Hepatic clearance of carvedilol and the iMPC nano-formulations. **g-h,** Pulmonary clearance of aPD-L1 and the iMPC nano-formulations. **i-j,** Renal clearance of aPD-L1 and the iMPC nano-formulations. **k-l,** Hepatic clearance of aPD-L1 and the iMPC nano-formulations. n = 3 biologically independent mice per

group for data in **a-l**. n = 5 biologically independent mice per group for data in **a-l**. Data are expressed as the mean ± s.d.

The description has been added (page 25 line 597-617) as: In contrast, compared with the free carvedilol or iMPC group, the amount of carvedilol entering the bloodstream was significantly reduced in the iMPC+US group (Supplementary Fig. 14a). The *in vivo* half-life of carvedilol was 6 h (Supplementary Fig. 14a). Compared with the free aPD-L1 or iMPC groups, the amount of aPD-L1 entering the bloodstream was significantly reduced in the iMPC+US group (Supplementary Fig. 14b). The *in vivo* half-life of aPD-L1 was 36 h (Supplementary Fig. 14b). These data indicate that carvedilol and aPD-L1 administered intranasally enter the circulation, with aPD-L1 persisting longer than carvedilol.

The distribution of carvedilol and aPD-L1 across the heart, liver, spleen, lungs, kidneys, and brain in various treatment groups showed that the iMPC+US group had significantly lower aPD-L1 levels in the liver and lungs compared to free aPD-L1 or iMPC alone, while the distribution in brain tumors was notably higher (Supplementary Fig. 15a-b). Compared with free carvedilol or iMPC, the iMPC+US group showed significantly reduced carvedilol distribution in the liver and lungs, while brain tumor distribution was significantly increased (Supplementary Fig. 15c-d).

Clearance rate measurements indicated that carvedilol was rapidly cleared from liver, kidney, and lung tissues within 6 hours, while aPD-L1 exhibited a significantly delayed clearance, extending beyond 48 hours (Supplementary Fig. 16a-l). These data indicate that US enhances iMPC brain penetration efficiency by opening the NMB, thereby delivering carvedilol and aPD-L1 to tumor regions. Compared to free carvedilol or the nanoparticle formulation, the clearance rate of free aPD-L1 or the nanoparticle formulation is slower.

The description has been added (page 61 line 1546-1954) as: **Pharmacokinetics of iMPC *in vivo*** The pharmacokinetic behavior of carvedilol, aPD-L1, and various nano-formulations was studied by administering multiple nano-formulations intranasally to healthy C57 mice (n = 3). The administered dosages were 30 μg of aPD-L1 and 4 μg of carvedilol per 20 g of body weight. Ultrasound parameters included a 1.5 w/cm²

intensity, 30% duty cycle, 1 MHz center frequency, and a 60-second duration. Blood samples were collected at 0, 1, 2, 4, 8, 12, 24, 36, 48, and 72 hours following intranasal administration. Serum was extracted and subjected to acid treatment. Carvedilol and aPD-L1 concentrations were measured using a Tecan Safire fluorescence microplate reader (Lyon, France).

Intra-body distribution and clearance rates in major organs of iMPC

To investigate the in vivo distribution of aPD-L1, 6-8-week-old C57 mice (20-22 g) were selected for the experiment, with males and females randomly assigned. The experiment involved nasal administration and was divided into four groups: control, aPD-L1, iMPC, and iMPC+US. LIPUS targeted the olfactory epithelium within the nasal cavity. aPD-L1 was labeled with Cy5-NHS (Starleaf Biological Technology Co, Nanjing, China). The aPD-L1 dosage was administered at 30 μg per 20 g of body weight. IVIS imaging, conducted six hours later, identified fluorescent signals in the heart, liver, spleen, lungs, kidneys, and brain across all four groups. Fluorescence signal values were statistically analyzed using ImageJ software (ImageJ Software Inc., USA).

To investigate the in vivo distribution of aPD-L1, 6-8-week-old C57 mice (20-22 g) were selected for the experiment, with males and females randomly assigned. The experiment involved nasal administration and was divided into four groups: control, aPD-L1, iMPC, and iMPC+US. LIPUS targeted the olfactory epithelium within the nasal cavity. aPD-L1 was labeled with Cy5-NHS (Starleaf Biological Technology Co, Nanjing, China). The aPD-L1 dosage was administered at 30 μg per 20 g of body weight. Six hours later, IVIS imaging revealed fluorescent signals in the heart, liver, spleen, lungs, kidneys, and brain across all four groups. Fluorescence signal values were statistically analyzed using ImageJ software (ImageJ Software Inc., USA).

The in vivo distribution of carvedilol was examined by categorizing the experiment into four groups: control, carvedilol, iMPC, and iMPC+US. Carvedilol was labeled with Cy5-NHS (Starleaf Biological Technology Co., Nanjing, China). Carvedilol was administered at a dosage of 4 μg per 20 g of body weight. Six hours after administration, IVIS imaging revealed fluorescence in the heart, liver, spleen, lungs, kidneys, and brain

of mice in all four groups. Fluorescence signal values were statistically analyzed using ImageJ software (ImageJ Software Inc., USA).

Further evaluate the clearance rates of carvedilol and aPD-L1 in the lungs, kidneys, and liver. Select 6-8-week-old C57 mice (20-22 g) for the experiment, randomly assigning males and females, and administer the drugs intranasally. LIPUS was administered to the olfactory epithelium in the mouse nasal cavity. Experimental groups included: carvedilol group, aPD-L1 group, iMPC group, and iMPC+US group. aPD-L1 was administered at 30 μg per 20 g of body weight, while carvedilol was given at 4 μg per 20 g of body weight. Carvedilol and aPD-L1 were conjugated with Cy5-NHS (Starleaf Biological Technology Co, Nanjing, China). Ultrasound parameters were set to 1.5 W/cm^2 with a 30% duty cycle, a center frequency of 1 MHz, and a duration of 60 seconds. Lungs, kidneys, and livers were collected and homogenized from mice at 0, 1, 2, 6, 12, 24, 48, and 72 hours post-treatment. Fluorescence signal intensity was measured using IVIS, and quantitatively analyzed with a PerkinElmer multifunctional microplate reader (Boston, MA, USA).

3. Did the authors examine whether activated $CD8^+$ T cells or dendritic cell maturation in lymph node?

Re: Thank you for your helpful suggestions. We further examined the activation status of $CD8^+$ T cells and dendritic cells (DCs) in deep cervical lymph nodes.

Concurrently, we assessed DCs activation in axillary lymph nodes. Results demonstrated that LIPUS-mediated iMPC nasal-brain delivery significantly activated CD8+ T cells and DCs in deep cervical lymph nodes. No significant DCs activation was observed in axillary lymph nodes.

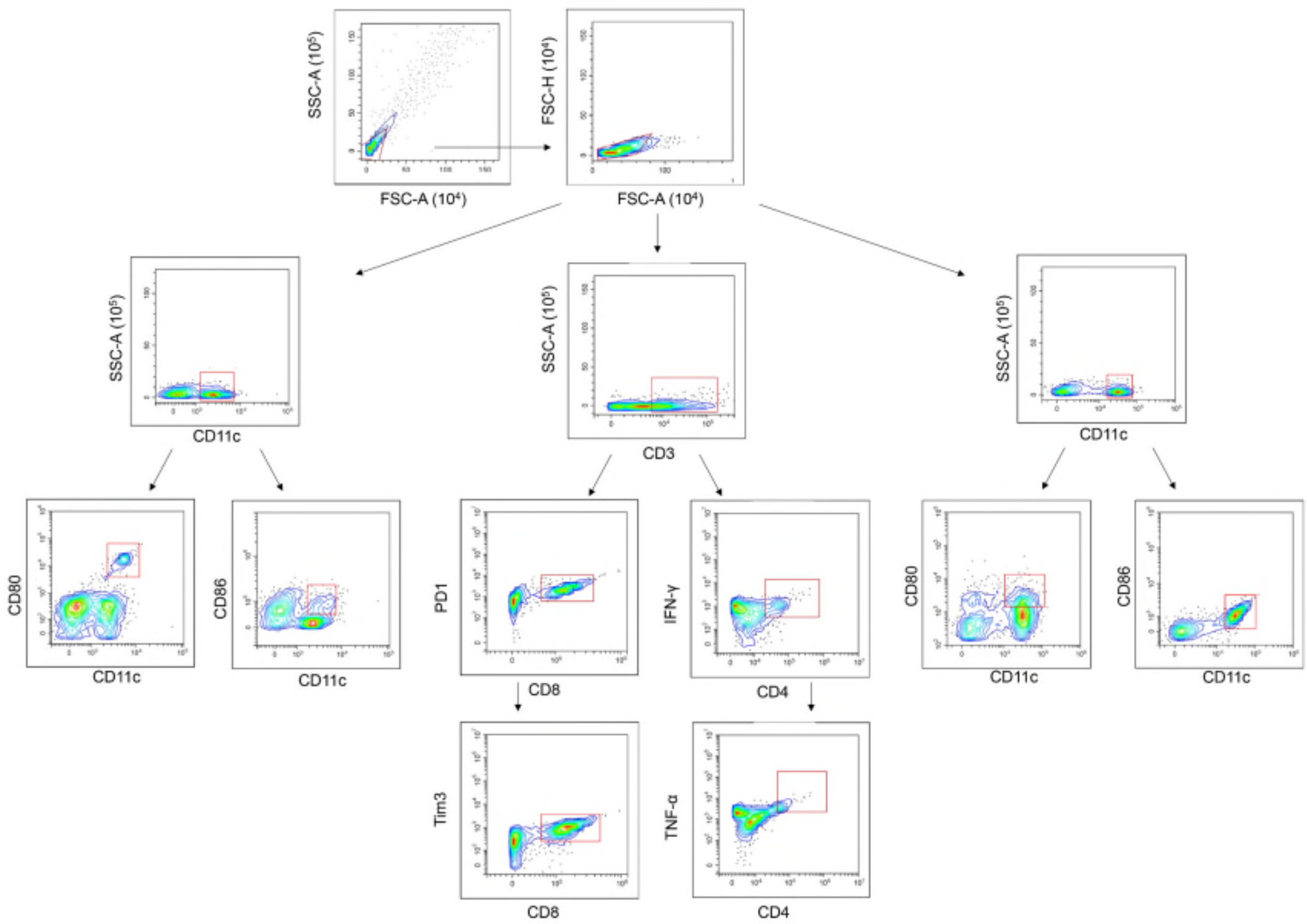

Supplementary Figure 26. Flow cytometry gating logic for dendritic cells (DCs), CD8$^+$ T cells, and CD4$^+$ T cells in cervical deep lymph nodes and axillary lymph nodes.

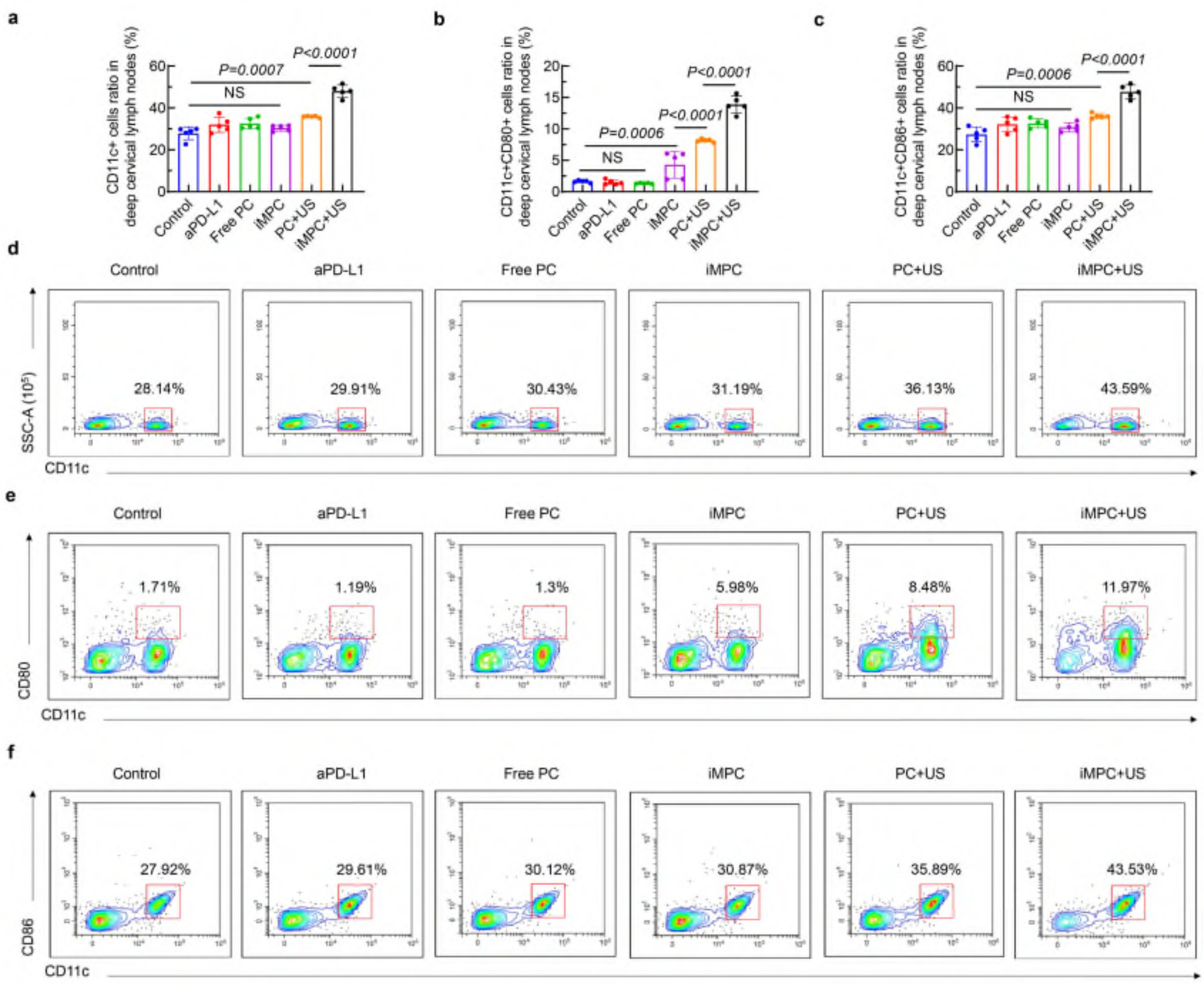

Supplementary Figure 27. The proportion of dendritic cells (DCs) in deep cervical lymph nodes increased, with enhanced activity. a, d, Proportion of DCs in deep cervical lymph nodes. **b, e,** Proportion of DCs highly expressing CD80 in deep cervical lymph nodes. **c, f,** Proportion of DCs highly expressing CD86 in deep cervical lymph nodes. n = 5 biologically independent mice per group for data in c-f and **a-c**. Data are expressed as the mean ± s.d. Statistical significances were determined using one-way ANOVA with Dunnett's post hoc test. Comparisons were performed between different groups in **a-c**. Not significant (NS) is $P \geq 0.05$, and significant P values are shown.

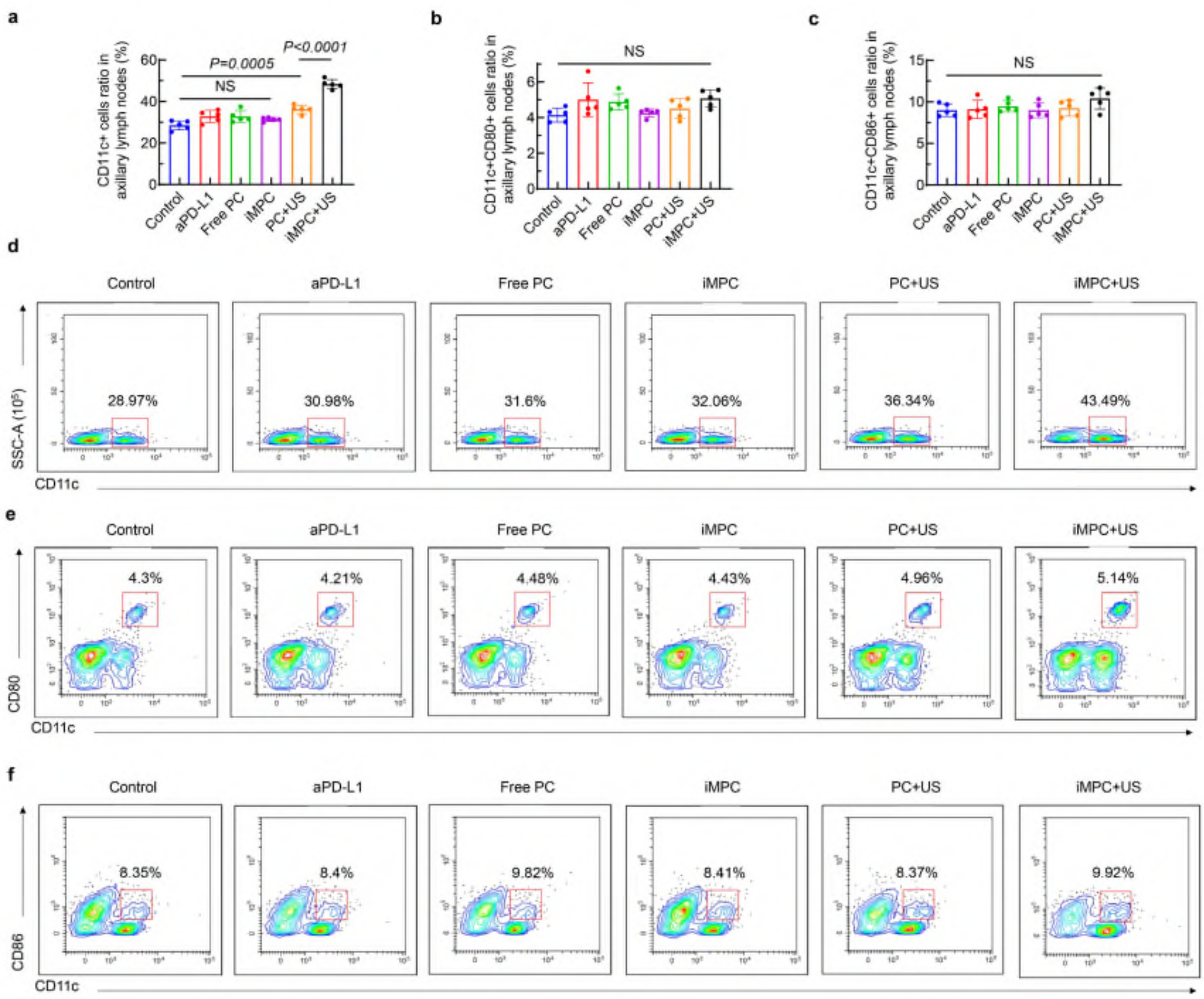

Supplementary Figure 28. The proportion of dendritic cells (DCs) in axillary lymph nodes increased. a, d, Proportion of DCs in axillary lymph nodes. **b, e,** Proportion of DCs highly expressing CD80 in axillary lymph nodes. **c, f,** Proportion of DCs highly expressing CD86 in axillary lymph nodes. n = 5 biologically independent mice per group for data in c-f and **a-c**. Data are expressed as the mean ± s.d. Statistical significances were determined using one-way ANOVA with Dunnett's post hoc test. Comparisons were performed between different groups in **a-c**. Not significant (NS) is $P \geq 0.05$, and significant P values are shown.

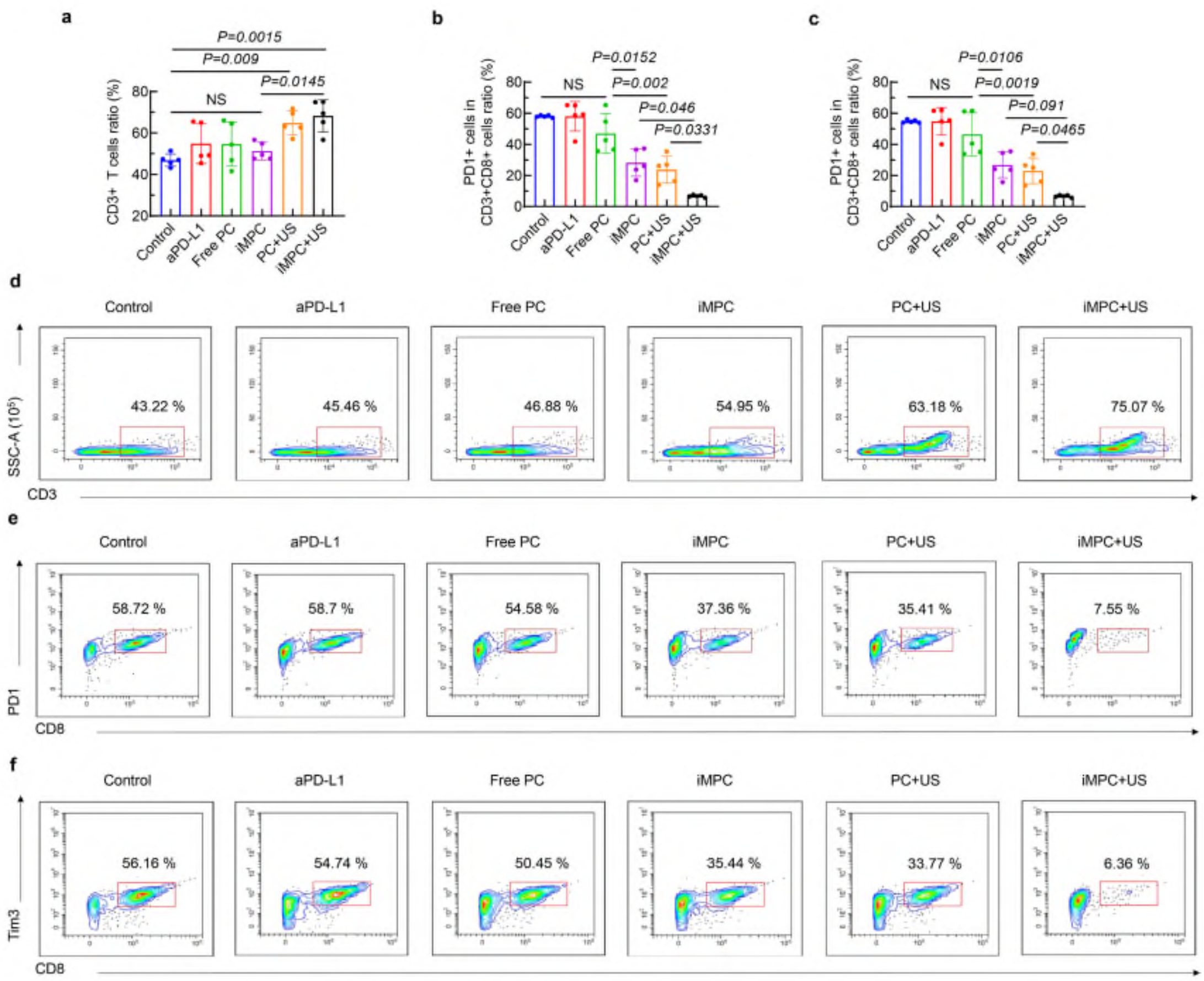

Supplementary Figure 29. The proportion of CD3$^+$CD8$^+$ T cells in deep cervical lymph nodes increased, with enhanced activity. a, d, Proportion of CD3$^+$ T cells in deep cervical lymph nodes. **b, e,** Proportion of PD1-expressing CD3$^+$CD8$^+$ T cells in deep cervical lymph nodes. **c, f,** Proportion of Tim3-expressing CD3$^+$CD8$^+$ T cells in deep cervical lymph nodes. n = 5 biologically independent mice per group for data in c-f and **a-c**. Data are expressed as the mean ± s.d. Statistical significances were determined using one-way ANOVA with Dunnett's post hoc test. Comparisons were performed between different groups in **a-c**. Not significant (NS) is $P \geq 0.05$, and significant P values are shown.

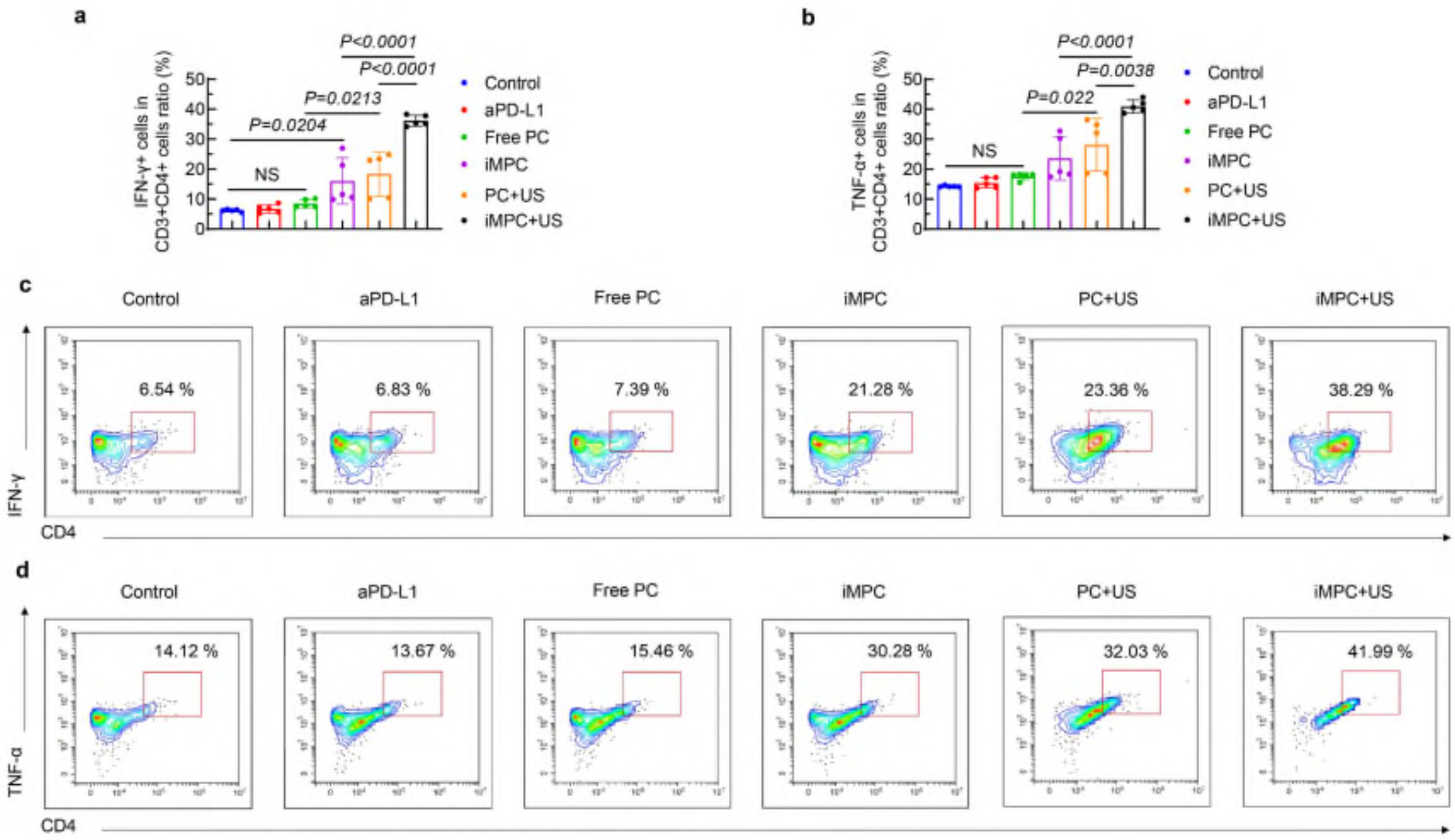

Supplementary Figure 30. The proportion of $CD3^+CD4^+$ T cells in deep cervical lymph nodes increased, with enhanced activity. a, c, Proportion of $CD3^+CD4^+$ T cells expressing IFN-γ in deep cervical lymph nodes. **b, d,** Proportion of $CD3^+CD4^+$ T cells expressing TNF-α in deep cervical lymph nodes. n = 5 biologically independent mice per group for data in c-f and **a-b**. Data are expressed as the mean ± s.d. Statistical significances were determined using one-way ANOVA with Dunnett's post hoc test. Comparisons were performed between different groups in **a-b**. Not significant (NS) is $P \geq 0.05$, and significant P values are shown.

The description has been added (page 38 line 924-973) as: **Enhanced activity of dendritic cells (DCs) and $CD8^+$ T cells in cervical deep lymph nodes.** Additional analysis of DC, $CD4^+$ T cell, and $CD8^+$ T cell activation in lymph nodes is provided in Supplementary Fig. 26. Flow cytometry of deep cervical lymph nodes showed an increase in DC proportions by 128.39% in the PC+US group and 154.90% in the iMPC+US group compared to the control group (Supplementary Fig. 27a and d). The iMPC group exhibited a 3.49-fold increase in the proportion of DCs with high CD80 expression compared to the control group (Supplementary Fig. 27b and e). Compared to the Free PC group, the proportion of DCs highly expressing CD80 increased 6.52-fold in the PC+US group and 9.21-fold in the iMPC+US group (Supplementary Fig. 27b and e). Compared with the iMPC group, the proportion of DCs highly expressing CD80 increased 1.42-fold in the PC+US group and 2.01-fold in the iMPC+US group (Supplementary Fig. 27b and e). The proportion of DCs with high CD86 expression increased by 119.16% in the PC+US group and by 144.52% in the iMPC+US group

compared to the Free PC group (Supplementary Fig. 27c and f). These data indicate that LIPUS-mediated iMPC naso-cerebral delivery induces DCs activation in deep cervical lymph nodes.

Analysis of DCs activity in ipsilateral axillary lymph nodes showed a 1.25-fold increase in the PC+US group and a 1.50-fold increase in the iMPC+US group relative to the control group (Supplementary Fig. 28a and d). Importantly, none of the experimental groups, including iMPC+US, exhibited significantly higher CD86 or CD80 expression on DCs compared to the control group (Supplementary Fig. 28b-c and e-f). This indicates that LIPUS-mediated iMPC naso-cerebral delivery primarily influences local immune regulation within the brain tumor region.

Increased DC activity frequently stimulates T cells, necessitating further assessment of $CD8^+$ and $CD4^+$ T cell activity in deep cervical lymph nodes . The study found that the T cell proportion increased by 146.18% in the PC+US group and by 173.69% in the iMPC+US group compared to the control group (Supplementary Fig. 29a and d). The PC+US group exhibited a 1.66-fold reduction in $PD1^{high}$ $CD8^+$ T cell proportion compared to the control group, while the iMPC+US group showed a 7.78-fold decrease (Supplementary Fig. 29b and e). Compared with the iMPC group, the proportion of $PD1^{high}$ $CD8^+$ T cells decreased 4.95-fold in the iMPC+US group (Supplementary Fig. 29b and e). The PC+US group exhibited a 1.66-fold reduction in PD-1^{high} $CD8^+$ T cells, while the iMPC+US group showed an 8.83-fold decrease, compared to the control group (Supplementary Fig. 29c and f). The iMPC+US group exhibited a 5.57-fold reduction in the proportion of $Tim3^{high}$ $CD8^+$ T cells compared to the iMPC group (Supplementary Fig. 29c and f). These data indicate that LIPUS-mediated iMPC naso-cerebral delivery induces an increased proportion of T cells in deep cervical lymph nodes and enhanced $CD8^+$ T cell activity.

The evaluation of $CD4^+$ T cell activity in deep cervical lymph nodes revealed that the proportion of IFN-γ^{high} $CD4^+$ T cells increased by 3.57 times in the PC+US group and 5.85 times in the iMPC+US group compared to the control group (Supplementary Fig. 30a and c). The iMPC+US group exhibited a 1.79-fold increase in the proportion of IFN-γ^{high} $CD4^+$ T cells compared to the iMPC group (Supplementary Fig. 30a and c).

The proportion of TNF-α^{high} $CD4^+$ T cells increased by 2.27-fold in the PC+US group and 2.97-fold in the iMPC+US group compared to the control group (Supplementary Fig. 30b and d). The iMPC+US group exhibited a 1.39-fold increase in the proportion of IFN-γ^{high} $CD4^+$ T cells compared to the iMPC group (Supplementary Fig. 30b and d). These data indicate that LIPUS-mediated iMPC nasal-brain delivery simultaneously enhances $CD4^+$ T cell activity in deep cervical lymph nodes.

The description has been added (page 73 line 1892-1904) as: Assess the status of DCs and T cells in lymph nodes *via* flow cytometry. 5 days post-treatment, deep cervical and axillary lymph node tissues were harvested from GBM mice. Cell suspensions were isolated using 0.5 μg/mL collagenase D (Roche, Switzerland), 0.5 μg/mL DNase I (Vazyme Biotec, China), and 3 μg/mL DNase I (Sigma-Aldrich, USA). Cells were stained for 90 minutes using the Zombie NIR™ Fixable Viability Kit and antibodies against CD3, CD4, CD8a, CD11c, CD80, CD86, IFN-γ, TNF-α (BioLegend, USA; catalog numbers: 100204, 100537, 100732, 117305, 104714, 105026, 505808, 506307), and PD1 (143705, BioLegend, USA). Antibody staining for anti-IFN-γ, anti-TNF-α, and anti-PD1 was conducted following BioLegend's intracellular staining protocol (424401; BioLegend, USA). Stained cells underwent flow cytometric analysis to identify various cell types, including $CD3^+$ T cells, $PD1^+CD3^+CD8^+$ T cells, $Tim3^+CD3^+CD8^+$ T cells, TNF-α^+CD4^+ T cells, IFN-γ^+CD4^+ T cells, $CD11c^+$ dendritic cells (DCs), $CD86^+CD11c^+$ DCs, and $CD80^+CD11c^+$ DCs, with 20,000 events collected for analysis. Flow cytometric analysis antibodies were sourced from Dakewe Biotech Co., Ltd., while analytical grade chemicals were procured from Sigma-Aldrich (USA).

4. For the biocompability, the authors should be considered to provide the cytotocixity of these materials in normal cell lines.

Re: Thank you for your suggestion. We supplemented the CCK-8 assay to evaluate the effects of iMPC immune-nanoparticles on cell viability in human nasal epithelial cells (HNEpC), mouse brain capillary endothelial cells (bEnd.3), human neuroblastoma cell line (SH-SY5Y), rat pheochromocytoma cell line (PC12), and mouse neuroblastoma

cell line (Neuro-2a). The results demonstrate that iMPC exhibits favorable biosafety as an antitumor immunostimulatory nanomedicine.

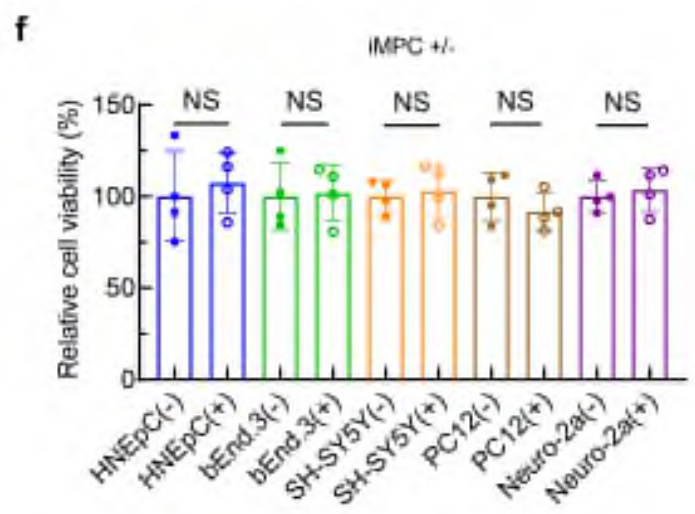

Supplementary Figure 8f, Cell viability of HNEpC, bEnd.3, SH-SY5Y, PC12, and Neuro-2a cells after treatment with iMPC nanoformulation.

The description has been added (page 17 line 426-429) as: Furthermore, compared to the group without iMPC treatment (-), the cell viability of HNEpC, bEnd.3, SH-SY5Y, PC12, and Neuro-2a cells in the iMPC-treated group (+) showed no significant changes (Supplementary Fig. 8f). This suggests that iMPC, as an anti-tumor immune-enhancing nanomedication, exhibits favorable biosafety.

The description has been added (page 59 line 1493-1499) as: Further evaluate the safety of iMPC nanoparticle formulations. HNEpC, bEnd.3 , SH-SY5Y, PC12, and Neuro-2a cells were seeded into 96-well plates at a concentration of 1×10^6 cells/mL. Experimental groups were as follows: HNEpC group, iMPC+HNEpC group, bEnd.3 group, iMPC+bEnd.3 group, SH-SY5Y group, iMPC+SH-SY5Y group, PC12 group, iMPC+PC12 group, Neuro-2a group, and iMPC+Neuro-2a group. The iMPC concentration was 35 μg/mL. Cell viability was evaluated using the CCK-8 assay following a 24-hour conventional culture.

5. It seems that only one cell line GL261 was used in this study. The authors should repeat their key discoveries in another GBM cell lines.

Re: Thank you for your valuable suggestions. We further utilized the mouse GBM cell line G422-Luc to establish GBM mice model in BALB/c mice, thereby validating the antitumor effect of LIPUS-mediated iMPC nasal-to-brain delivery in GBM mice. Results indicate that LIPUS-mediated iMPC nasal-to-brain delivery exhibits broad-

spectrum anti-GBM effects.

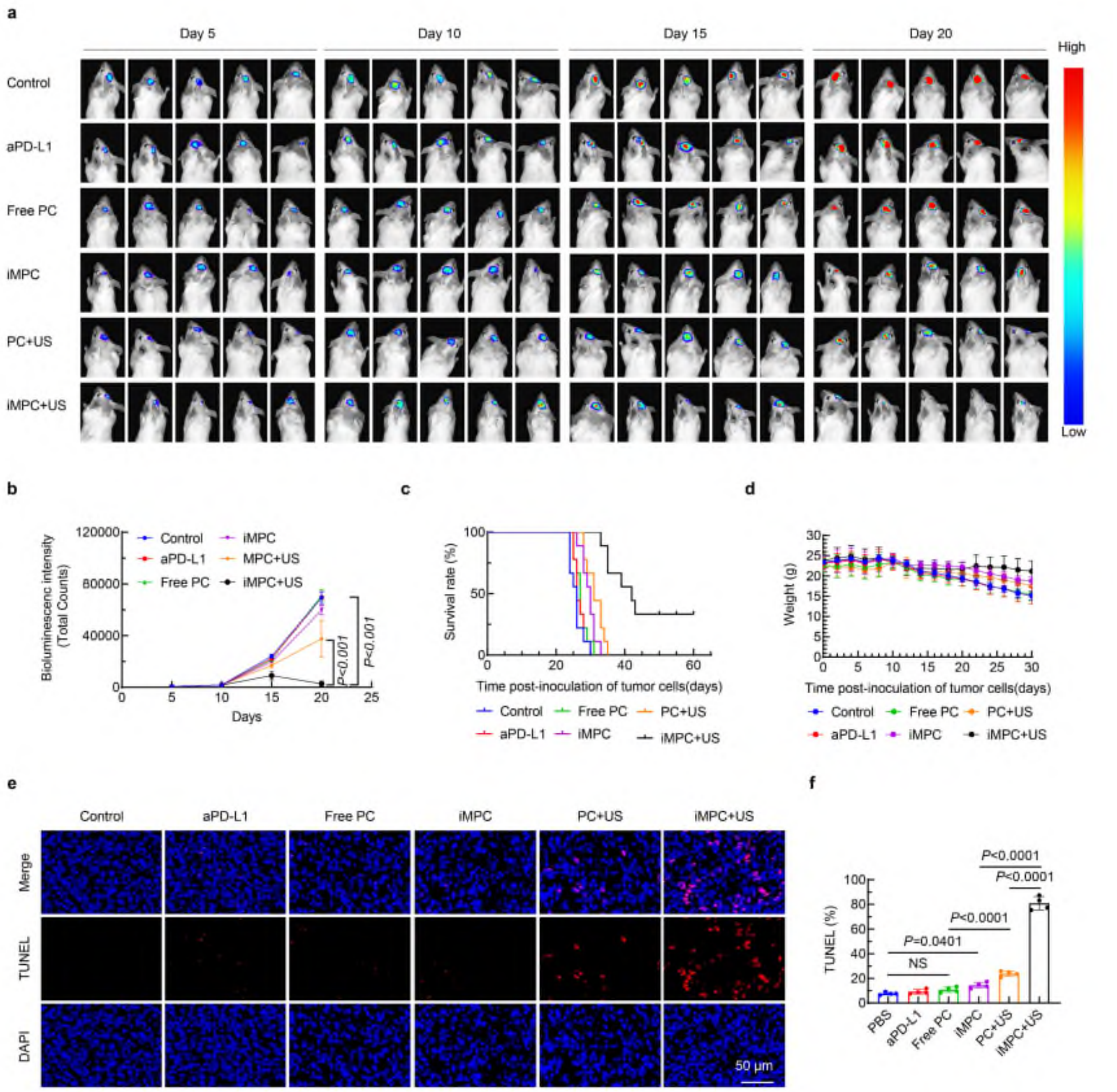

Supplementary Figure 22. Evaluation of antitumor efficacy. a-b, *In vivo* imaging system (*IVIS*) assessment of tumor growth inhibition in G422-Luc cell-implanted in situ glioblastoma (GBM) mouse models across different treatment groups. **c,** Survival curves of GBM tumor-bearing mice in different treatment groups (n = 10). Statistical significance was calculated using the log-rank test. **d,** Body weight changes of GBM mice in different treatment groups (n = 10). **e-f,** Apoptotic proportion in tumor tissues of GBM mice across different treatment groups. n = 5 biologically independent mice per group for data in **a-b**. n = 4 biologically independent mice per group for data in **f**. Data are expressed as the mean ± s.d. Statistical significances were determined using one-way ANOVA with Dunnett's post hoc test. Comparisons were performed between different groups in **a-b** and **f**. Not significant (NS) is $P \geq 0.05$, and significant P values are shown. n = 10 biologically independent mice per group for data in **c-d**. Statistical significance was calculated using the log-rank test.

The description has been added (page 34 line 829-843) as: The G422-Luc cell line-derived GBM mice showed notably reduced tumor growth in the PC+US group relative

to the Free PC group, as evidenced by IVIS antitumor assessment (Supplementary Fig. 22a-b). Tumor growth in the iMPC+US group was further inhibited relative to the PC+US group (Supplementary Fig. 22a-b). The survival period of GBM mice was extended to 35 days in the PC+US group and 60 days in the iMPC+US group, compared to the control group (Supplementary Fig. 22c).

Compared to other groups where body weight of GBM mice began to decrease on day 10, the iMPC+US group showed no significant weight loss (Supplementary Fig. 22d). Tumor tissue apoptosis rates were 2.19 times higher in the PC+US group and 7.47 times higher in the iMPC+US group compared to the control group (Supplementary Fig. 22e-f). The data suggest that LIPUS-enhanced iMPC nasal-to-brain drug delivery significantly suppresses tumor growth in GBM mice.

These results show that combining LIPUS-mediated nose-to-brain delivery with iMPC significantly boosts antitumor effects in GBM-bearing mice by inhibiting tumor growth, inducing apoptosis, extending survival time, and enhancing overall survival.

The description has been added (page 70 line 1794-1803) as: To systematically assess the effectiveness of LIPUS-mediated nasal-to-brain delivery of iMPC for GBM suppression, a GBM mouse model was developed using the G422-Luc cell line and BALB/c mice (6–8 weeks old, 20–22 g), with random assignment of both male and female subjects. The treatment regimen was identical to the previous protocol. Tumor growth progression was monitored via IVIS imaging to evaluate antitumor efficacy. Concurrently, daily measurements of body weight changes and survival status were recorded for mice in different treatment groups. Five days post-treatment, a TUNEL assay kit (Beyotime Biotech, Shanghai, China) was employed for immunofluorescence staining of tumor sections. ImageJ software was utilized for image analysis.

6. The authors mentioned about thermal damage for using LFUS, but no quantitative thermal imaging was shown to exclude temperature-induced damage. Please provide the in vitro and in vivo images for thermal inducing at different irradiation power and time points. What is the key factor of iMPC that make it response to LFUS?

Re: We further supplemented the experiments to explore the "thermal effect" of LIPUS on the nasal mucosa. Results indicated that as ultrasound intensity increased, the temperature of the nasal mucosa rose continuously, posing a risk of causing "thermal injury." Notably, when LIPUS intensity was set at 1.5 w/cm², it did not significantly alter the histological structure of mouse nasal mucosa or induce inflammatory infiltration, and NMB opening was reversible.

Furthermore, the LIPUS-mediated iMPC nasal-to-brain delivery process involves: first applying LIPUS to the olfactory region of the nasal mucosa in GBM mice, followed by intranasal instillation of the iMPC suspension. LIPUS and iMPC were performed sequentially.

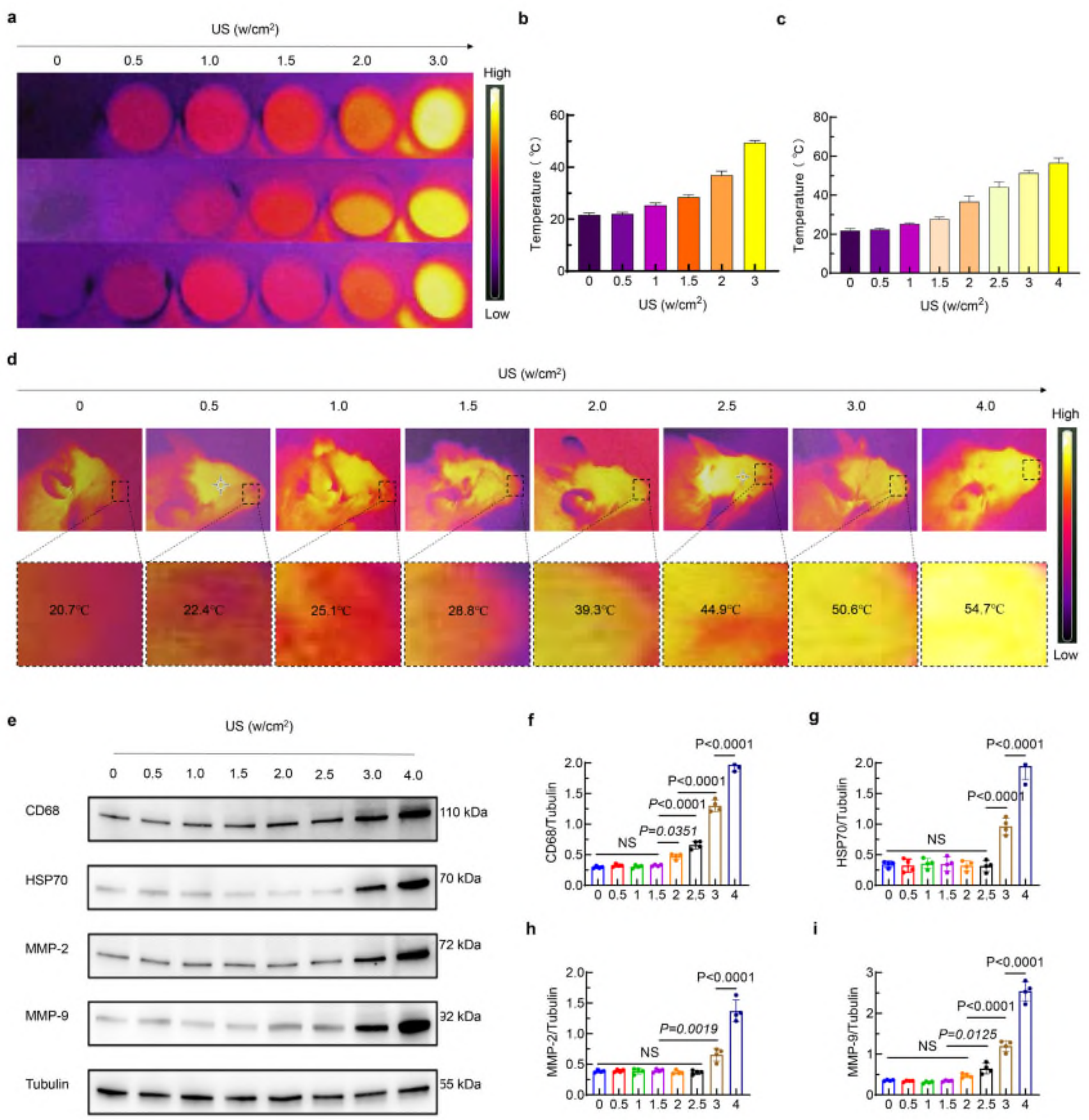

Supplementary Figure 17. Thermal injury of LIPUS on nasal mucosa. a-b, Temperature changes in the nasal mucosal barrier (NMB) model constructed from human nasal epithelial cells (HNEpC). **c-d,** Regional temperature changes in mouse nasal mucosa. **e-h,** Expression levels of HSP70, CD68, and MMP-9. Gray-scale analysis was performed using ImageJ software. n = 3 biologically independent mice per group for data in **a-c**. n = 4 biologically independent mice per group for data in **f-i**. Data are expressed as the mean ± s.d. Statistical significances were determined using one-way ANOVA with Dunnett's post hoc test. Comparisons were performed between different groups in **a-c** and **f-i**. Not significant (NS) is $P \geq 0.05$, and significant P values are shown.

The description has been added (page 26 line 619-648) as: Sustained LIPUS exposure often induces varying degrees of "thermal effects." Thermal imaging results indicate that the temperature of the NMB model increases with rising US intensity (Supplementary Fig. 17a-b). The NMB model attained a temperature of 29.3°C after 60 seconds of continuous ultrasound exposure at an intensity of 1.5 w/cm² (Supplementary Fig. 17a-b). At an US intensity of 3.0 w/cm² with 60 seconds of continuous exposure, the NMB model reached a temperature of 50.1°C (Supplementary Fig. 17a-b).

In vivo experiments demonstrated that the temperature in the mouse nasal mucosa region gradually increased with rising US intensity (Supplementary Fig. 17c-d). Specifically, when US intensity reached 3.0 w/cm² and was applied continuously for 60 seconds, the temperature in the mouse nasal mucosa region reached 50.6°C (Supplementary Fig. 17c-d). At an US intensity of 4.0 w/cm² with a 60-second exposure duration, the temperature in the mouse nasal mucosa region reached 54.7°C (Supplementary Fig. 17c-d). These data indicate that sustained exposure to US at certain intensities may cause thermal injury to nasal mucosal tissue.

WB analysis indicated that CD68 levels in the nasal mucosal tissue of mice exposed to 1.5 w/cm² US were comparable to the control group (0 w/cm²), whereas exposure to 3.0 w/cm² US resulted in a 4.41-fold increase in CD68 levels (Supplementary Fig. 17e-f). HSP70 levels in the nasal mucosa of mice exposed to 1.5 w/cm² ultrasound showed no significant change compared to the control group (0 w/cm²), whereas exposure to 3.0 w/cm² resulted in a 2.83-fold increase (Supplementary Fig. 17g).

The US group (1.5 w/cm²) exhibited no significant differences in matrix metalloproteinase-2 (MMP-2) and matrix metalloproteinase-9 (MMP-9) levels in nasal mucosal tissue compared to the control group (0 w/cm²) (Supplementary Fig. 17h-i).

MMP-2 and MMP-9 levels in the nasal mucosal tissue of the US (3.0 w/cm²) group rose by 1.71-fold and 3.38-fold, respectively (Supplementary Fig. 17h-i). These data indicate that US treatment at an intensity of 3.0 w/cm² induces "thermal injury" and inflammatory infiltration in the nasal mucosal tissue region of mice. LIPUS at 1.5 w/cm² intensity did not significantly alter mouse nasal mucosal tissue structure or cause inflammatory infiltration.

The description has been added (page 65 line 1661-1678) as: **LIPUS-induced thermal injury to the nasal mucosa** Further evaluation of the thermal effect of LIPUS on NMB. HNEpCs were seeded at $1*10^6$ cells/mL in 500 μL into the upper chamber of a 24-well Transwell® migration insert (400 nm pore size; Millipore, MA, USA). After conventional culture for 5 days, LIPUS treatment was applied at intensities of 0, 0.5, 1.0, 1.5, 2.0, and 3.0 w/cm². The other US parameters were as follows: duty cycle 30%, center frequency 1 MHz, and duration 60 s. Subsequently, temperature changes in the NMB were assessed using an infrared thermal imager (TIS65, FLUKE, USA) and statistically analyzed.

The olfactory epithelium in the nasal cavity of 6-8-week-old C57 mice (20-22 g) underwent LIPUS treatment at ultrasound intensities of 0, 0.5, 1.0, 1.5, 2.0, 2.5, 3.0, and 4.0 w/cm², with male and female mice randomly assigned. The US parameters included a 30% duty cycle, 1 MHz center frequency, and 60-second duration. Temperature changes in the nasal cavity's olfactory region were then measured with an infrared thermal imager (TIS65, FLUKE, USA) and analyzed statistically. Western blot analysis was conducted to assess the expression of HSP70, CD68, MMP-2, MMP-9, and Tubulin in the nasal mucosa of the olfactory epithelium in C57 mice following LIPUS treatment at varying ultrasound intensities, using antibodies from Abcam, UK. Statistical analysis of gray values was conducted using ImageJ software (ImageJ Software Inc., USA).

References

1. Ma, Z. et al. Controlled tough bioadhesion mediated by ultrasound. Science 377, 751-755 (2022).

2. Schoellhammer, C. M. et al. Applicability and safety of dual-frequency ultrasonic treatment for the transdermal delivery of drugs. J Control Release 202, 93-100 (2015).

3. Yu, C. C. et al. A Conformable Ultrasound Patch for Cavitation-Enhanced Transdermal Cosmeceutical Delivery. Advanced materials (Deerfield Beach, Fla.) 35, e2300066 (2023).

7. In Figure 5b, it seems that the authors take the different mouse' s brain at different time points. It might be unconvinced due to the different responses with strange tissues in each mouse. The authors should be considered to provide the MRI images of live GBM-bearing mice at designated time points (before and after treatment), rather than from different animals.

Re: Thank you for your critical suggestion. We have revised Figure 6b to ensure that MRI images of live GBM mice at specified time points (before and after treatment) are derived from the same animal.

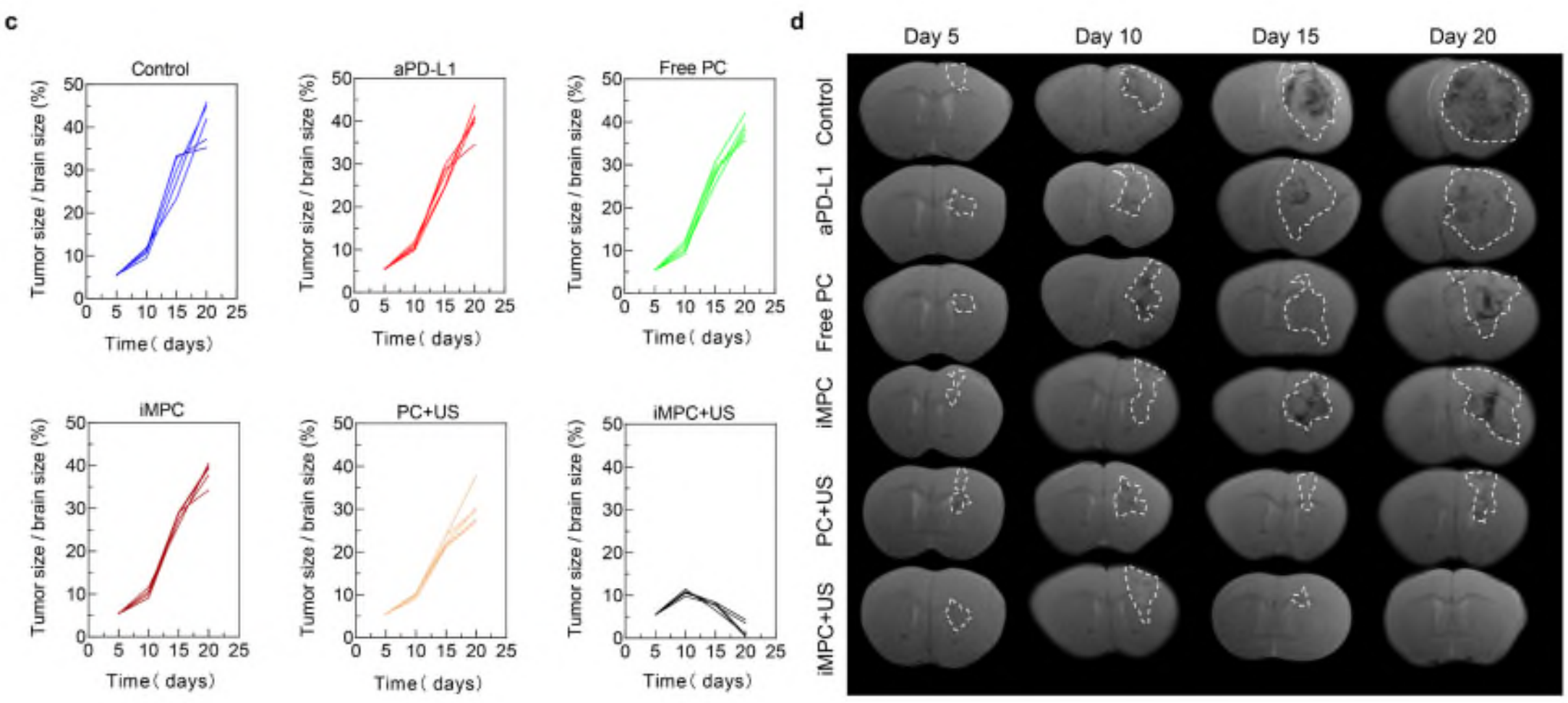

Figure 6. c-d, Brain images of each group of mice obtained by T2 MRI with different treatments, where tumors are circled in white dashed lines.

8. Please add the scale bar for Figure 1e-upper, Figure 4l-n, Supplementary Figure 1k, Supplementary Figure 12a,c, and highlight the words of e to g in Figure 2 legends.

Re: Thank you for your careful suggestions. We have revised these errors.

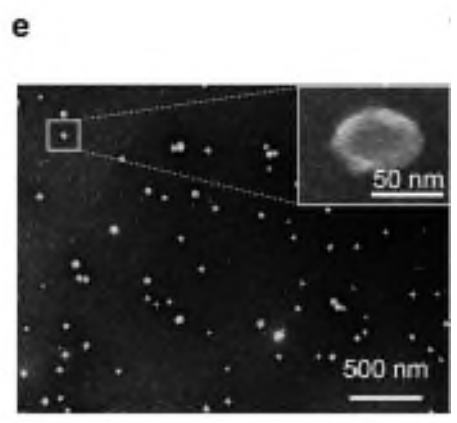

Figure 1e, Transmission electron microscopy image of extracellular vesicles from the macrophage cell line RAW 264.7.

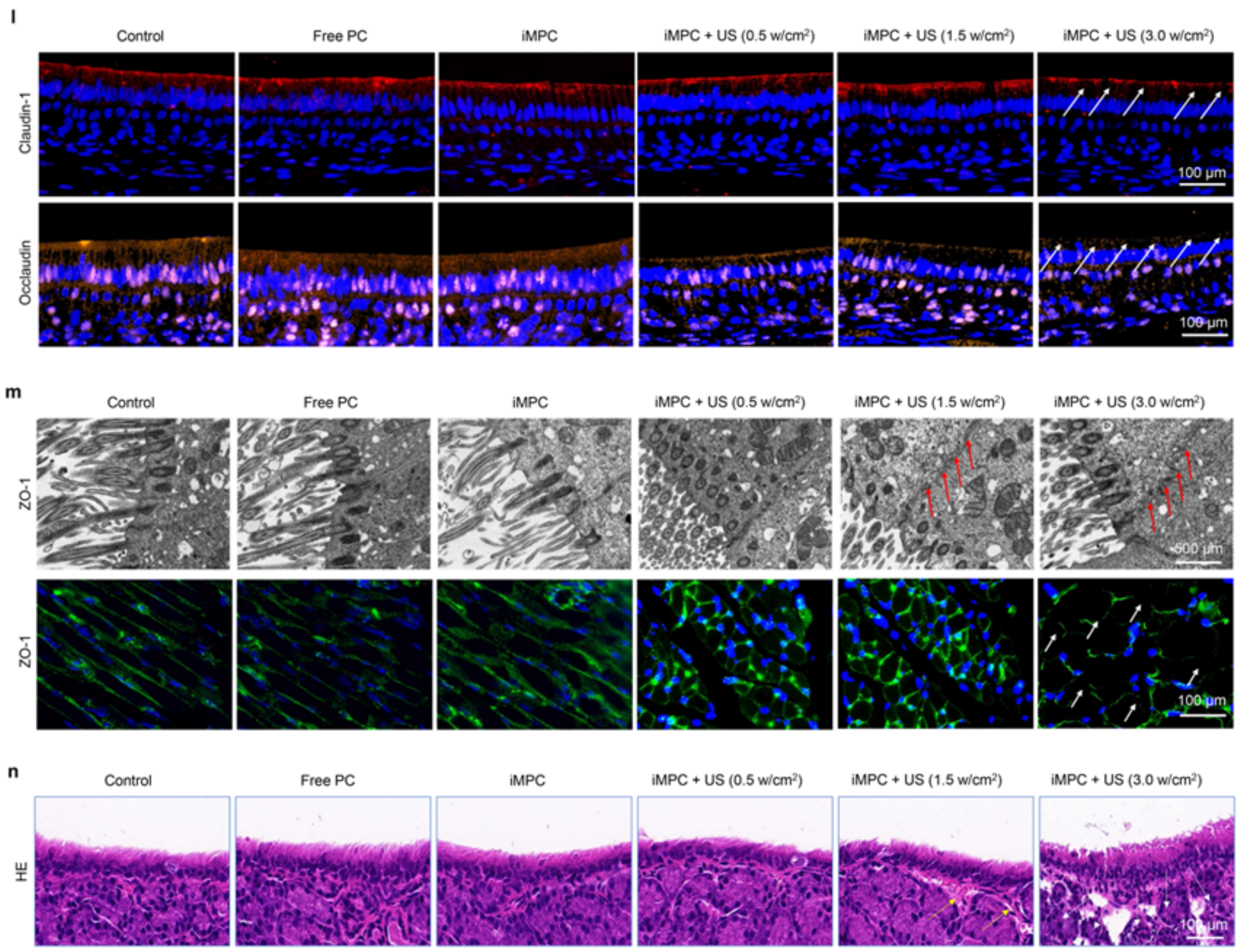

Figure 4l, Changes in tight junction proteins Occludin and Claudin-1 (blue: DAPI; red: CY3-Claudin-1; yellow: SpRed-Occludin). **m**, Alterations in tight junction proteins of the paracellular pathway (blue: DAPI; green: Alexa Fluor 488-ZO-1). **n**, Inflammatory cell infiltration after various treatments.

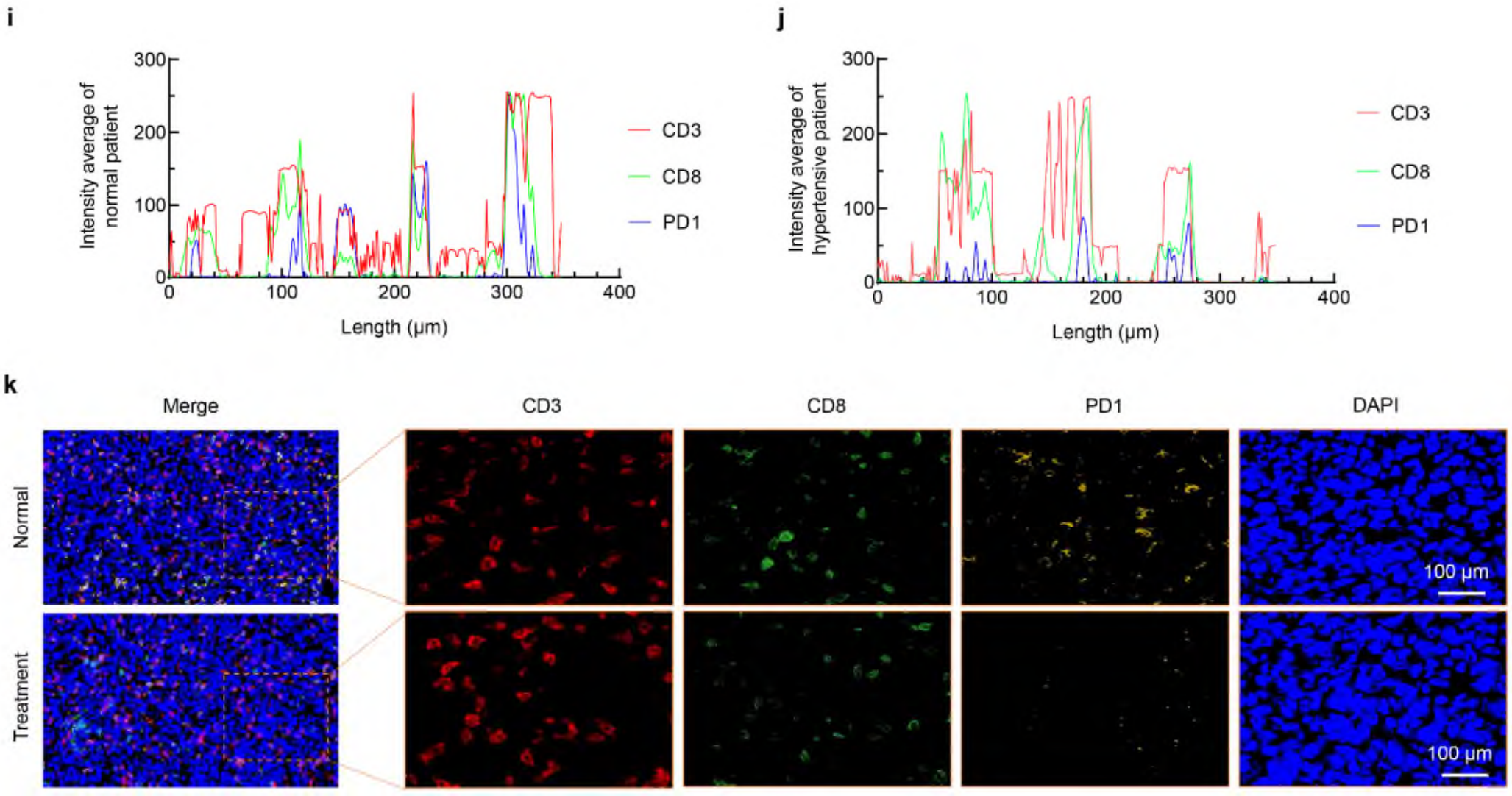

Supplementary Figure 1i-k, Immunofluorescence triple staining was performed on tumor tissues from GBM patients who did (j) and did not (i) receive norepinephrine inhibitors (blue: DAPI; green: Alexa fluor 488-CD8; red: CY3-CD3; yellow: SpRed-PD1). Fluorescence co-localization analysis by ImageJ software.

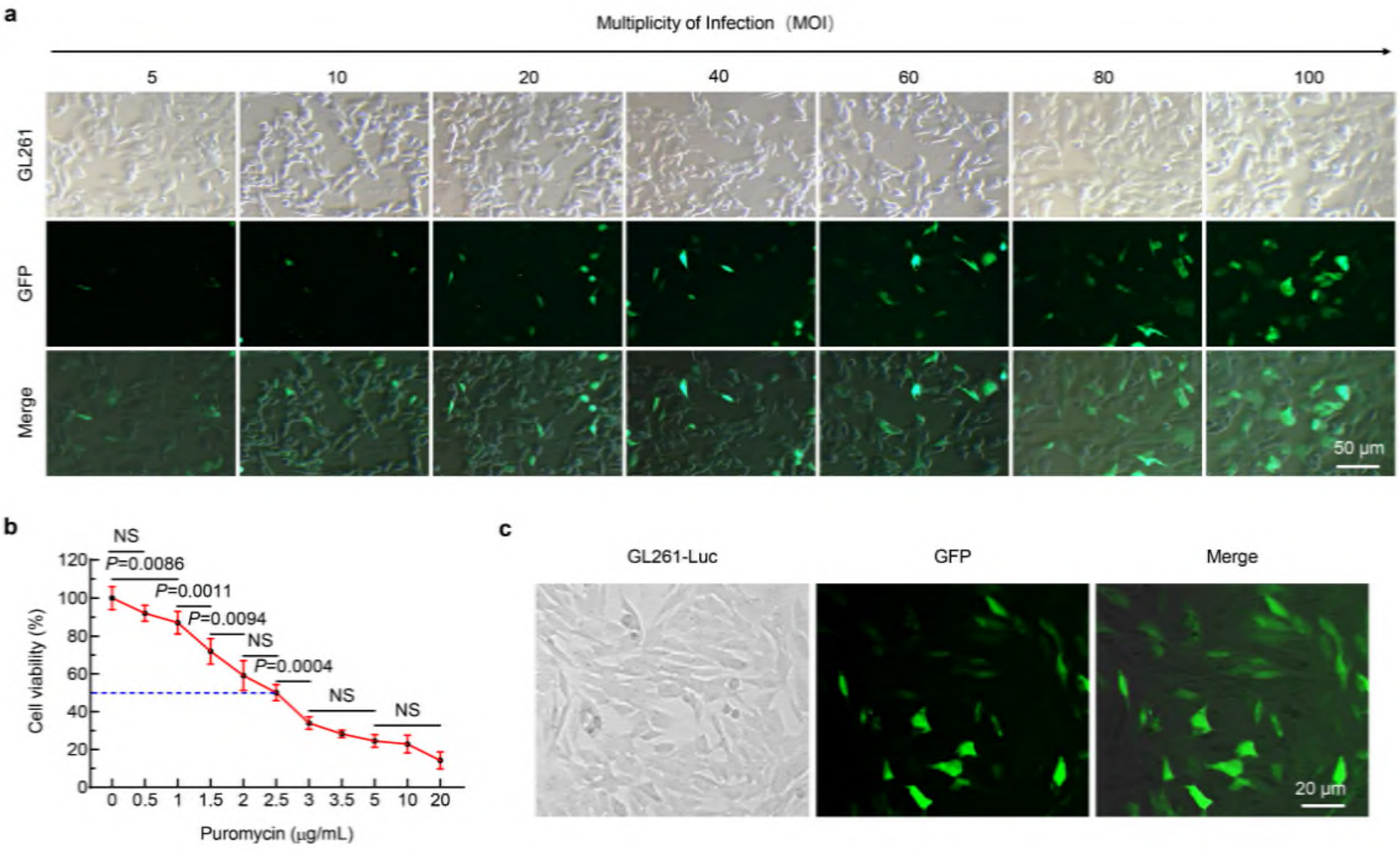

Supplementary Figure 21a, The transfection efficiency of GL261 cells at different multiplicity of infection (MOI). **b**, The effect of different concentrations of puromycin on the viability of GL261 cells, with an IC50 of 2.5 μg/mL. **c**, Stable GFP-expressing GL261-Luc cell line.

9. Please pay attention to the format of the reference (for example, only the first letter of the title is capitalized in reference 1, while first letters of every words are capitalized reference 3).

Re: Thank you for your careful suggestions. We have completed the revisions to the references format.

The description has been added (page 76 line 1974-2096) as:

References

1. Zhao, X., Ye, Y., Ge, S., Sun, P. & Yu, P. Cellular and molecular targeted drug delivery in central nervous system cancers: advances in targeting strategies. Curr. Top. Med. Chem. 20, 2762-2776 (2020).

2. Nance, E., Pun, S. H., Saigal, R. & Sellers, D. L. Drug delivery to the central nervous system. Nature reviews Materials 7, 314-331 (2022).

3. Shen, H. et al. Engineered commensals for targeted nose-to-brain drug delivery. Cell 188, 1545-1562.e16 (2025).

4. Qiu, Y. et al. The nasal-brain drug delivery route: mechanisms and applications to central nervous system diseases. MedComm 6, e70213 (2025).

5. Long, J. M. & Holtzman, D. M. Alzheimerzheimer disease: an update disease: an update on pathobiology and treatment strategies. Cell 179, 312-339 (2019).

6. Liu, Y. et al. Customized intranasal hydrogel delivering methylene blue ameliorates cognitive dysfunction against Alzheimer's disease. Advanced materials (Deerfield Beach, Fla.) 36, e2307081 (2024).

7. Ruan, S. et al. Microneedle-mediated nose-to-brain drug delivery for improved Alzheimer's disease treatment. J Control Release 366, 712-731 (2024).

8. Ma, Z. et al. Controlled tough bioadhesion mediated by ultrasound. Science 377, 751-755 (2022).

9. Schoellhammer, C. M. et al. Applicability and safety of dual-frequency ultrasonic treatment for the transdermal delivery of drugs. J Control Release 202, 93-100 (2015).

10. Guo, J. et al. Bio-barrier-adaptable biomimetic nanomedicines combined with ultrasound for enhanced cancer therapy. Signal Transduct Target Ther 10, 137 (2025).

11. Yu, C. C. et al. A conformable ultrasound patch for cavitation-enhanced transdermal cosmeceutical delivery. Advanced materials (Deerfield Beach, Fla.) 35, e2300066 (2023).

12. Sonabend, A. M. et al. Repeated blood-brain barrier opening with an implantable ultrasound device for delivery of albumin-bound paclitaxel in patients with recurrent glioblastoma: a phase 1 trial. Lancet Oncol. 24, 509-522 (2023).

13. Dong, L. et al. Open-source throttling of CD8+ T cells in the brain with low-intensity focused ultrasound-guided sequential delivery of CXCL10, IL-2, and aPD-L1 for glioblastoma immunotherapy. Advanced materials (Deerfield Beach, Fla.) 36, e2407235 (2024).

14. Chen, Y. et al. Intranasal drug delivery: The interaction between nanoparticles and the nose-to-brain pathway. Adv. Drug Del. Rev. 207, 115196 (2024).

15. Wang, G. et al. Tailored borneol-modified lipid nanoparticles nasal spray for enhanced nose-to-brain delivery to central nervous system diseases. ACS Nano 18, 23684-23701 (2024).

16. Wang, J. et al. Ultrasound-mediated blood-brain barrier opening: An effective drug delivery system for theranostics of brain diseases. Adv. Drug Del. Rev. 190, 114539 (2022).

17. Bausart, M., Préat, V. & Malfanti, A. Immunotherapy for glioblastoma: the promise of combination strategies. JOURNAL OF EXPERIMENTAL & CLINICAL CANCER RESEARCH 41, 35 (2022).

18. Bikfalvi, A. et al. Challenges in glioblastoma research: focus on the tumor microenvironment. Trends Cancer 9, 9-27 (2023).

19. Kang, J. H. & Zappasodi, R. Modulating Treg stability to improve cancer immunotherapy. Trends Cancer 9, 911-927 (2023).

20. Liston, A., Pasciuto, E., Fitzgerald, D. C. & Yshii, L. Brain regulatory T cells. Nat. Rev. Immunol. 24, 326-337 (2024).

21. Globig, A. M. et al. The β1-adrenergic receptor links sympathetic nerves to T cell exhaustion. Nature 622, 383-392 (2023).

22. Zebley, C. C. & Youngblood, B. Mechanisms of T cell exhaustion guiding next-generation immunotherapy. Trends Cancer 8, 726-734 (2022).

23. Vu, Y. & Amit, M. Glioblastoma hijacks cholinergic networks. Cancer Cell 43, 1982-1984 (2025).

24. Ge, K. et al. Engineering EVs-mediated mRNA delivery regulates microglia function and alleviates depressive-like behaviors. Advanced materials (Deerfield Beach, Fla.) 37, e2418872 (2025).

25. Du, W. et al. A combined "eat me/don't eat me" strategy based on exosome for acute liver injury treatment. Cell Reports Medicine 6, 102033 (2025).

26. Pandey, N. et al. Nanotherapeutic treatment of the invasive glioblastoma tumor microenvironment. Adv. Drug Del. Rev. 188, 114415 (2022).

27. Pan, Y. et al. Glycoengineering-based anti-PD-1-iRGD peptide conjugate boosts antitumor efficacy through T cell engagement. Cell Reports Medicine 5, 101590 (2024).

28. Ding, N. et al. iRGD synergizes with PD-1 knockout immunotherapy by enhancing lymphocyte infiltration in gastric cancer. Nat Commun 10, 1336 (2019).

29. Li, Q. et al. Poly(aspartic acid)-based pH-responsive targeting co-delivery nanoparticles. J. Biomater. Appl. 36, 579-591 (2021).

30. Németh, C., Gyarmati, B., Abdullin, T., László, K. & Szilágyi, A. Poly(aspartic acid) with adjustable pH-dependent solubility. Acta Biomater. 49, 486-494 (2017).

31. Gyenes, T., Torma, V., Gyarmati, B. & Zrínyi, M. Synthesis and swelling properties of novel pH-sensitive poly(aspartic acid) gels. Acta Biomater. 4, 733-744 (2008).

32. Hakeem, A. et al. Polyaspartic acid-anchored mesoporous silica nanoparticles for pH-responsive doxorubicin release. Int J Nanomedicine 13, 1029-1040 (2018).

33. Vega-Chacón, J., Arbeláez, M., Jorge, J. H., Marques, R. & Jafelicci, M. pH-responsive poly(aspartic acid) hydrogel-coated magnetite nanoparticles for biomedical applications. Mater Sci Eng C Mater Biol Appl 77, 366-373 (2017).

34. Dong, L. et al. A gambogic acid-loaded delivery system mediated by ultrasound-targeted microbubble destruction: A promising therapy method for malignant cerebral glioma. Int J Nanomedicine 17, 2001-2017 (2022).

35. Dong, Y. et al. Intracavitary spraying of nanoregulator-encased hydrogel modulates cholesterol metabolism of glioma-supportive macrophages for postoperative glioblastoma immunotherapy. Advanced materials (Deerfield Beach, Fla.) 36, e2311109 (2024).

36. Ma, Y. & Kroemer, G. The cancer-immune dialogue in the context of stress. Nat. Rev. Immunol. 24, 264-281 (2024).

37. Xu, X. et al. Recruiting T-cells toward the brain for enhanced glioblastoma immunotherapeutic efficacy by co-delivery of cytokines and immune checkpoint antibodies with macrophage-membrane-camouflaged nanovesicles. Advanced materials (Deerfield Beach, Fla.) 35, e2209785 (2023).

38. Xu, B. et al. Acidity-actuated polymer/calcium phosphate hybrid nanomotor (PCaPmotor) for penetrating drug delivery and synergistic anticancer immunotherapy. Nano Lett. 24, 10724-10733 (2024).

39. Pedder, J. H. et al. Crossing the blood-brain barrier: emerging therapeutic strategies for neurological disease. Lancet Neurol 24, 246-260 (2025).

40. Dong, L. et al. Recruiting T-cells toward the brain for enhanced glioblastoma chemo-immunotherapy efficacy by co-delivery of cytokines and temozolomide via ultrasound-gated redox-responsive extracellular vesicles. J Nanobiotechnology 23, 766 (2025).

41. Wang, Z. H. et al. Bioactive nanomotor enabling efficient intestinal barrier penetration for colorectal cancer therapy. Nat Commun 16, 1678 (2025).

42. Xin, G. et al. Single-cell RNA sequencing unveils an IL-10-producing helper subset that sustains humoral immunity during persistent infection. Nat Commun 9, 5037 (2018).

43. Hoekstra, M. E. et al. Long-distance modulation of bystander tumor cells by CD8+ T cell-secreted IFNγ. Nat Cancer 1, 291-301 (2020).

44. Jia, W. et al. Low-intensity pulsed ultrasound responsive scaffold promotes intramembranous and endochondral ossification via ultrasonic, thermal, and electrical stimulation. ACS Nano 19, 4422-4439 (2025).

45. Venturini, V. et al. The nucleus measures shape changes for cellular proprioception to control

dynamic cell behavior. Science 370, (2020).

46. Franklin, J. M., Ghosh, R. P., Shi, Q., Reddick, M. P. & Liphardt, J. T. Concerted localization-resets precede YAP-dependent transcription. Nat Commun 11, 4581 (2020).

47. Dong, Z. et al. TRPM-mediated mechanoreception regulates myosin oscillation during tissue elongation. Curr. Biol. 35, 5793-5807.e4 (2025).

48. Jadhav, K. et al. Trans-nasal brain delivery of anti-TB drugs by methyl-β-cyclodextrin microparticles show efficient mycobacterial clearance from central nervous system. J Control Release 378, 671-686 (2025).

49. Ma, F. et al. Tumor extracellular vesicle-derived PD-L1 promotes T cell senescence through lipid metabolism reprogramming. Sci. Transl. Med. 17, eadm7269 (2025).

50. Chegini, Z., Noei, M., Hemmati, J., Arabestani, M. R. & Shariati, A. The destruction of mucosal barriers, epithelial remodeling, and impaired mucociliary clearance: possible pathogenic mechanisms of Pseudomonas aeruginosa and Staphylococcus aureus in chronic rhinosinusitis. Cell Communication and Signaling 21, 306 (2023).

51. Moreno Ayala, M. A. et al. CXCR3 expression in regulatory T cells drives interactions with type I dendritic cells in tumors to restrict CD8+ T cell antitumor immunity. Immunity 56, 1613-1630.e5 (2023).

52. Tansey, M. G., Wallings, R. L., Houser, M. C., Herrick, M. K., Keating, C. E. & Joers, V. Inflammation and immune dysfunction in parkinson disease. Nat. Rev. Immunol. 22, 657-673 (2022).

53. Wang, H. et al. Scientific discovery in the age of artificial intelligence. Nature 620, 47-60 (2023).

54. Zhu, P., Simon, I., Kokalari, I., Kohane, D. S. & Rwei, A. Y. Miniaturized therapeutic systems for ultrasound-modulated drug delivery to the central and peripheral nervous system. Adv. Drug Del. Rev. 208, 115275 (2024).

55. Mruk, D. D. & Cheng, C. Y. The mammalian blood-testis barrier: its biology and regulation. Endocr. Rev. 36, 564-591 (2015).

56. Hori, T. et al. Trophoblast stem cell-based organoid models of the human placental barrier. Nat Commun 15, 962 (2024).

57. Dujardin, C., Habeler, W., Monville, C., Letourneur, D. & Simon-Yarza, T. Advances in the engineering of the outer blood-retina barrier: From in-vitro modelling to cellular therapy. Bioact Mater 31, 151-177 (2024).

Reviewer #2 (Remarks to the Author):

The authors developed a formulation in which aPD-L1 antibody and carvedilol were encapsulated into extracellular vesicles derived from RAW cells, and attempted brain tumor treatment via ultrasound-enhanced nose-to-brain delivery. The study is carefully analyzed with a very sophisticated experimental design. I would like to request revisions on the following points:

1 The rationale for using carvedilol in brain tumor therapy should be explained in the Abstract from the perspective of T-cell responses mediated by NE signaling.

Re: Thank you for your suggestion. NE promotes T cell exhaustion by binding to β receptors on the surface of T cells. Therefore, β-blockers can delay T cell exhaustion and enhance T cell antitumor activity.

The description has been added (page 2 line 38-43) as: A bionic nanovesicle (iRGD-anti-programmed cell death ligand 1 (aPD-L1) & carvedilol (β-blocker) @ macrophage-derived extracellular vesicles, iMPC) was designed to co-deliver carvedilol for β-receptor blockade to reduce T-cell exhaustion, and aPD-L1 to enhance T-cell anti-tumor activity in orthotopic glioblastoma (GBM) mice during the two-hour window for NMB opening.

2 While it is understood that the fabricated nano-iMPCs exhibit sufficient therapeutic effects, encapsulation of aPD-L1 antibodies into nanoparticles is not commonly performed. Although the encapsulation may facilitate brain delivery, appropriate release afterward is essential. Is there any evidence that release occurs before cellular internalization? It is unclear how polyaspartic acid is incorporated into the MPC and how it functions. In addition, while it is stated that nanoparticles disintegrate and release their contents under acidic conditions, the underlying mechanism remains unclear.

Re: Thank you for your comments. In previous studies, we successfully encapsulated anti-PD-L1 antibodies within nanoparticles (Advanced materials 36, e2407235 (2024); Advanced materials 35, e2209785 (2023)).

We have further supplemented our experiments. Results indicate that drug release

from iMPCs occurs prior to cellular internalization.

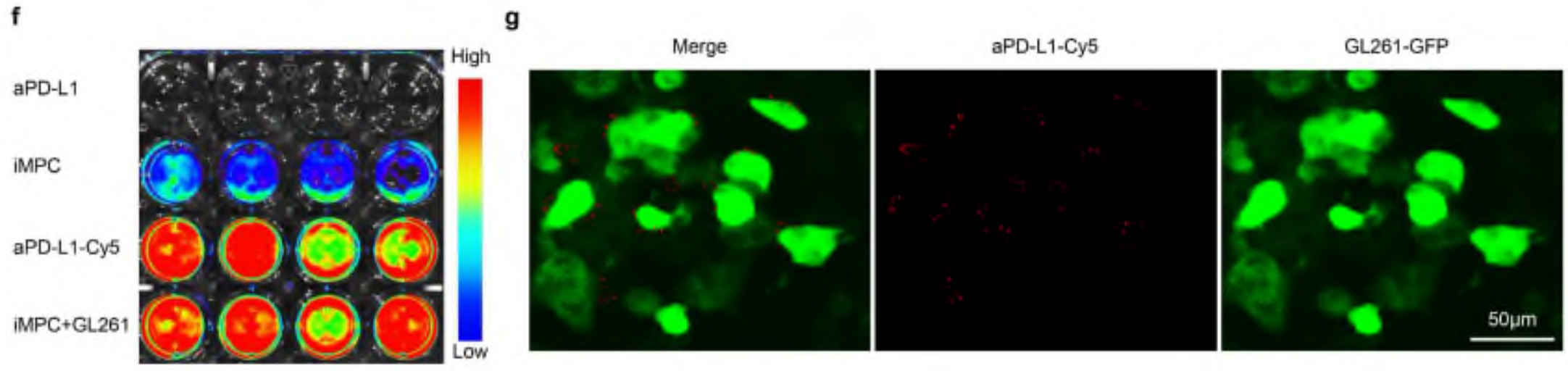

Supplementary Figure 4f, Extracellular aPD-L1-Cy5 fluorescence signal in GL261 cells. **g,** iMPCs release aPD-L1-Cy5 into the intercellular space of GL261-GFP cells.

The description has been added (page 13 line 320-325) as: Analysis of extracellular solutions from GL261 cells revealed enhanced fluorescence signals in the iMPC+GL261 group compared to the iMPC group, consistent with free aPD-L1-Cy5 fluorescence signals (Supplementary Fig. 4f). Confocal microscopy revealed that iMPC-released aPD-L1-Cy5 accumulated in the intercellular spaces of GL261-GFP cells (Supplementary Fig. 4g). These results indicate that iMPC release of aPD-L1 occurs in the intercellular spaces of tumor cells rather than inside tumor cells.

The description has been added (page 61 line 1535-1545) as: To confirm that iMPCs can deliver drugs into the intercellular spaces of GL261 cells, GL261-GFP cells were suspended at a density of 1×10^6 cells/mL and seeded into 48-well plates. After 24 h of routine culture, the experiment was divided into four groups: the aPD-L1 group (without GL261-GFP cells), the iMPC group (loaded with aPD-L1-Cy5, without GL261-GFP cells), aPD-L1-Cy5 group (without GL261-GFP cells), and iMPC+GL261-GFP group (loaded with aPD-L1-Cy5). The concentration of aPD-L1 was kept at 10 μg/mL. After 24 h of conventional culture, the four cell suspensions were removed and dialyzed (200 kDa, Acmec, Shanghai, China) for 12 h. The dialyzed fluid was collected and analyzed for fluorescence signals using IVIS imaging. Confocal microscopy was employed to examine the intercellular distribution of aPD-L1-Cy5 in the iMPC+GL261-GFP group.

We have expanded the description of how polyaspartic acid is incorporated into MPCs and how it functions.

The description has been added (page 4 line 104-110) as: Polyaspartic acid (PASP) is a typical *pH*-sensitive polymer that primarily responds to environmental pH changes

through the ionization properties of its side chains. Under acidic conditions (pH 5.5–6.5), PASP's negative charge rises due to the protonation of its side-chain carboxyl groups (-COOH/-COO^-). This causes polymer chain swelling, inducing structural changes in the nanocarrier and releasing the loaded drug. Conversely, at normal pH levels (such as pH 7.4, PASP maintains a relatively compact structure, inhibiting drug release.

The description has been added (page 55 line 1365-1378) as: The mass ratios of M-EVM to PASP were set at 10:1, 5:1, 3:1, 2:1, and 1:1, respectively. PASP was conjugated to M-EVM through a reaction with EDC/NHS to activate carboxyl groups, followed by a 4–6 hour incubation at room temperature. The resulting PASP-M-EVM complex was purified by dialysis to remove unreacted PASP and reagents. The PASP-M-EVM complex, aPD-L1, and carvedilol underwent six rounds of ultrasonication on ice. Ultrasonication parameters were set as follows: output power 35%, trigger interval 5 seconds, duration 1 minute, duty cycle 30%, frequency 20 kHz, on ice. The mixture was extruded using a membrane extruder (Avanti Polar Lipids, USA) to form MPCs, which were then extracted and purified. During extrusion, sodium cholate (1.5%), cholesterol (0.02%), leupeptin (0.2 μg/mL), and NaCl (500 mM) were added to the reaction system to improve flexibility and minimize membrane protein degradation. The MPCs were dissolved in a non-controlled rate cell cryopreservation medium (Cyagen Biosciences, China) to preserve protein functionality and then underwent freeze-thaw cycles to enhance loading efficiency.

References

1. Li, Q. et al. Poly(aspartic acid)-based pH-responsive targeting co-delivery nanoparticles. J. Biomater. Appl. 36, 579-591 (2021).
2. Németh, C., Gyarmati, B., Abdullin, T., László, K. & Szilágyi, A. Poly(aspartic acid) with adjustable pH-dependent solubility. Acta Biomater. 49, 486-494 (2017).
3. Gyenes, T., Torma, V., Gyarmati, B. & Zrínyi, M. Synthesis and swelling properties of novel pH-sensitive poly(aspartic acid) gels. Acta Biomater. 4, 733-744 (2008).
4. Hakeem, A. et al. Polyaspartic acid-anchored mesoporous silica nanoparticles for pH-responsive doxorubicin release. Int J Nanomedicine 13, 1029-1040 (2018).
5. Vega-Chacón, J., Arbeláez, M., Jorge, J. H., Marques, R. & Jafelicci, M. pH-responsive poly(aspartic acid) hydrogel-coated magnetite nanoparticles for biomedical applications. Mater Sci Eng C Mater Biol Appl 77, 366-373 (2017).

3 The EVs collected from RAW cells (Fig. 1e) may be smaller than typical ones.

Re: Thank you for your professional comments. We have revised the data. Transmission electron microscopy (TEM) shows that M-EVs exhibit a uniformly dispersed “cup-shaped” morphology with a particle size of about 50 nm (Fig. 1e). Particle size analysis revealed that M-EVs exhibited an average diameter of 51.34 ± 3.47 nm (Supplementary Fig. 4a).

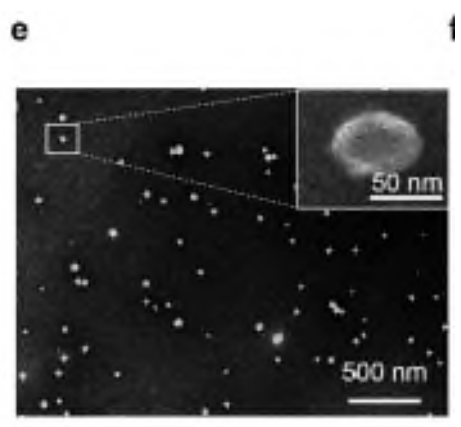

Figure 1e, Transmission electron microscopy image of extracellular vesicles from the macrophage cell line RAW 264.7.

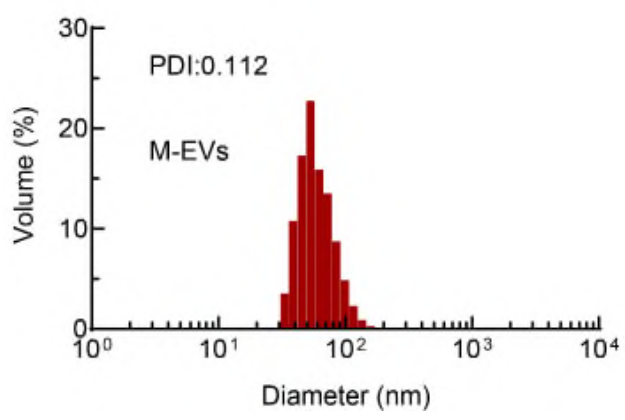

Supplementary Figure 4a, Particle size distribution of macrophage extracellular vesicles (M-EVs).

The description has been added (page 9 line 231-234) as: Transmission electron microscopy (TEM) shows that M-EVs exhibit a uniformly dispersed “cup-shaped” morphology with a particle size of about 50 nm (Fig. 1e). Particle size analysis revealed that M-EVs exhibited an average diameter of 51.34 ± 3.47 nm (Supplementary Fig. 4a).

4 In Fig. 1f, it is necessary to exclude the possibility of contamination by cellular components using markers such as Calnexin.

Re: Thank you for your professional comments. We have further supplemented the experiments to rule out the possibility of contamination from cellular components such as calnexin (endoplasmic reticulum), histone H3 (nucleus), and cytochrome C (chromosomes).

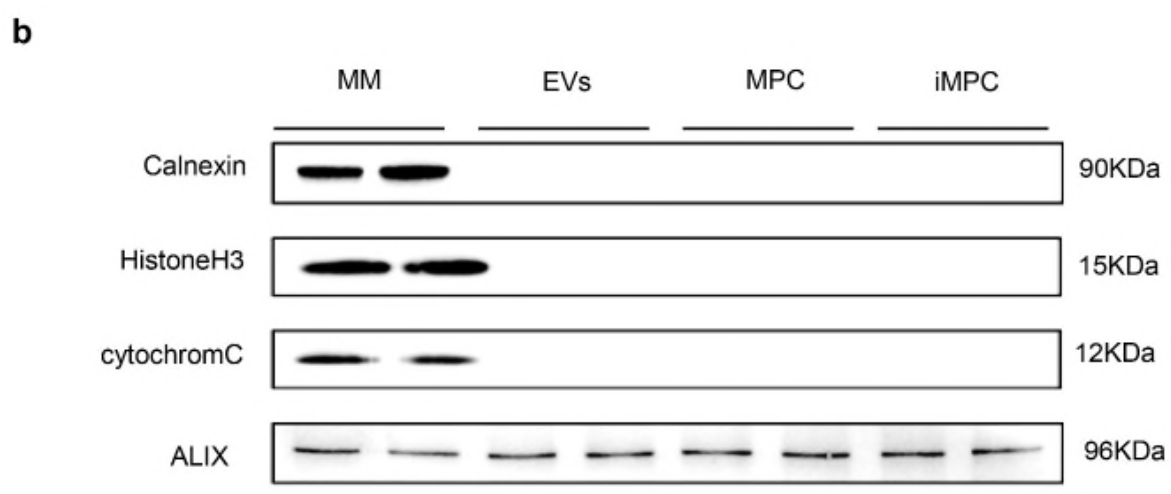

Supplementary Figure 4b, Expression levels of calnexin, histone H3, cytochrome C, and ALIX in macrophage membranes (MM), M-EVs, M-EVs loaded with aPD-L1 and carvedilol (MPC), and iRGD-modified MPC (iMPC).

The description has been added (page 10 line 238-245) as: Calnexin, Histone H3, and cytochrome C levels were significantly lower in the M-EVs, MPC, and iMPC groups compared to the MM group (Supplementary Fig. 4b). These markers ruled out the possibility of contamination from other cellular components such as the endoplasmic reticulum, cell nucleus, and chromosomes.

The description has been added (page 54 line 1346-1350) as: Mastersizer 3000 (Malvern Instruments, UK) was used to determine the average particle size distribution of M-EVs. Western blot analysis was conducted to identify M-EV markers, including TSG101, CD9, Calnexin, Histone H3, cytochrome C, and ALIX, using antibodies from Abcam, UK, confirming the successful isolation of M-EVs.

5 In Fig. 1o, the data for the original EVs should also be included.

Re: Thank you for your suggestion. In Fig. 1o, the data for the original EVs have been added.

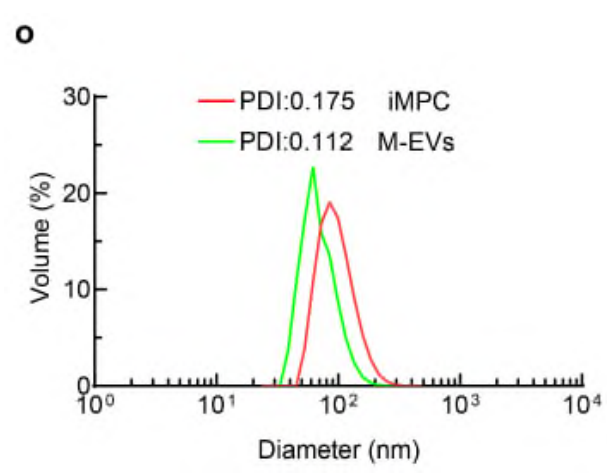

Figure 1o, The average particle size of M-EVs is 51.34±3.47 nm. The average particle size of iMPC is 102±4.54 nm.

6 In Fig. 2, high-intensity ultrasound appears to cause cell detachment. This phenomenon is unrelated to the decrease of TJ proteins and should be discussed

separately. In other words, applying ultrasound at levels that induce cell detachment is not appropriate for in vitro experiments. Permeability assessment and TJ protein expression should be evaluated under conditions where a certain TEER value is maintained.

Re: Thank you for your professional comments. We further examined the transendothelial electrical resistance (TEER) values to assess the impact of LIPUS on the permeability of the NMB model.

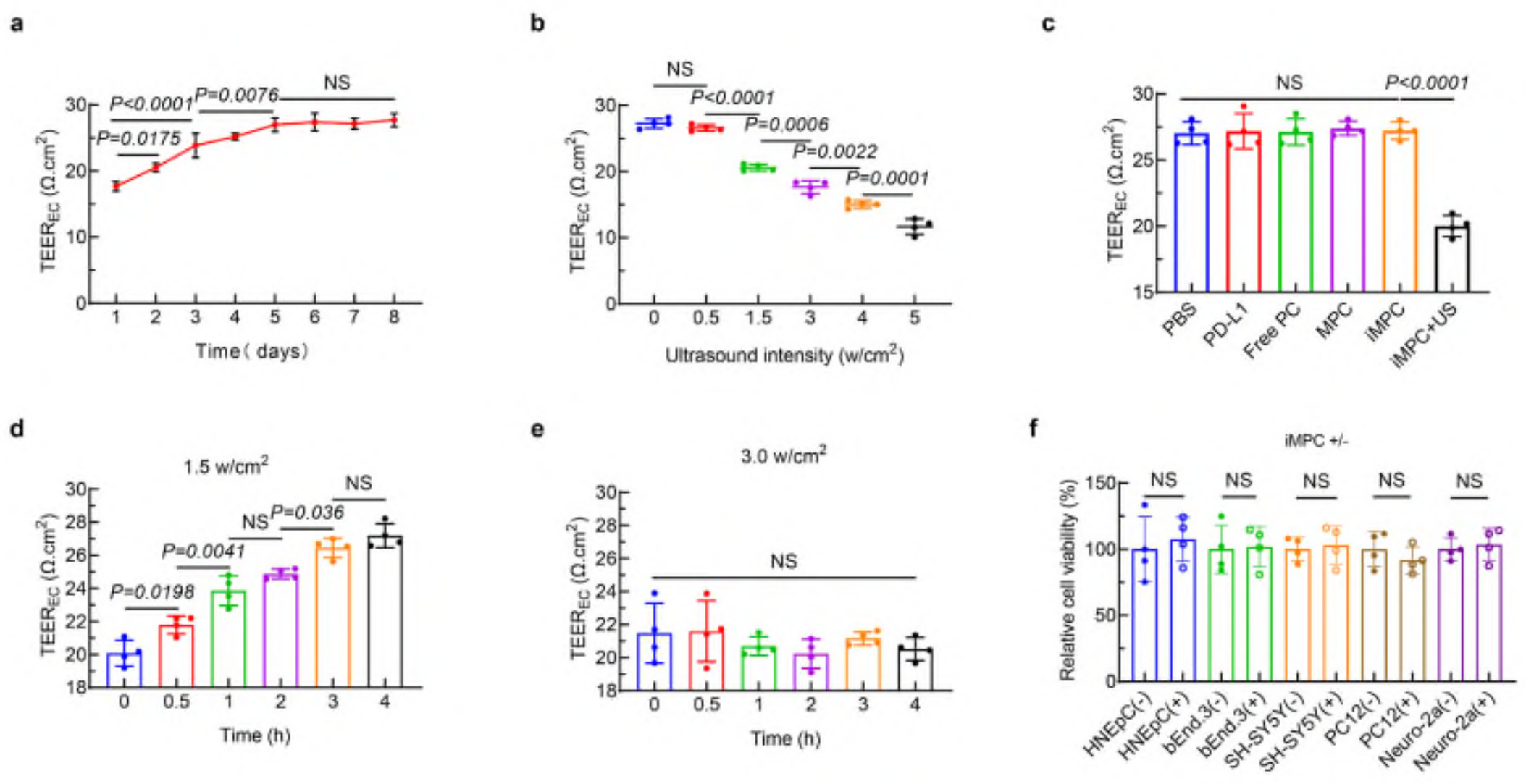

Supplementary Figure 8. Trans-epithelial electrical resistance (TEER) values in the nasal mucosal barrier (NMB) model. a, Changes in TEER values of the human nasal epithelial cell (HNEpC)-based NMB model (over 8 days). **b,** Changes in TEER values of the NMB model following treatment with different ultrasound intensities. **c,** Changes in TEER values of the NMB model after six different treatments. **d,** Ultrasonic intensity of 1.5 w/cm² was applied to NMB models, with changes in TEER values monitored over 4 hours. **e,** Ultrasonic intensity of 3.0 w/cm² was applied to NMB models, with changes in TEER values monitored over 4 hours. **f,** Cell viability of HNEpC, bEnd.3, SH-SY5Y, PC12, and Neuro-2a cells after treatment with iMPC nanoformulation. n = 4 biologically independent mice per group for data in **a-f**. Data are expressed as the mean ± s.d. Statistical significances were determined using one-way ANOVA with Dunnett's post hoc test. Comparisons were performed between different groups in **a-f**. Not significant (NS) is $P \geq 0.05$, and significant P values are shown.

The description has been added (page 16 line 395-429) as: The transendothelial electrical resistance (TEER) values in the NMB model stabilized by the fifth day, as shown in Supplementary Fig. 8a. An *in vivo* imaging system (*IVIS*) assessment demonstrated that the aPD-L1 content in the lower chamber progressively rose with increased US intensity (Fig. 2e-g). This conclusion was further validated by quantitative

measurements using a microplate reader (Fig. 2e-g). TEER measurement results indicate that the TEER value of the NMB model gradually decreases with increasing US intensity (Supplementary Fig. 8b). The findings suggest a significant correlation between the extent of NMB disruption and ultrasonic energy, highlighting the need to identify a safe ultrasound intensity range to ensure NMB damage reversibility.

We further investigate changes in tight junctions in the NMB model via immunofluorescence. The study found that US+iMPC treatment in the NMB model led to the disruption of tight junction proteins claudin-1 and occludin (Fig. 2h). Compared to other groups without US treatment, the TEER values for the NMB model in the US+iMPC group were significantly reduced (Supplementary Fig. 8c). The Free aPD-L1, P&C, MPC, and iMPC-treated groups showed no significant changes in the tight junction proteins claudin-1 and occludin, indicating that LIPUS, when not applied, does not significantly affect NMB (Fig. 2h). When other parameters (time 60s, duty cycle 50%, frequency 1MHz) remained constant, the disruption of claudin-1 and occludin became more pronounced with increasing US intensity (Fig. 2i). This suggests that the degree of NMB disruption gradually increases as the ultrasonic energy is enhanced (Fig. 2i).

To further assess the reversibility of NMB disruption by LIPUS, immunofluorescence experiments and TEER test were performed. The results demonstrated that when the LIPUS intensity was 1.5 w/cm^2, the NMB model could recover within 4 h (Fig. 2j and Supplementary Fig. 8d). In contrast, at a higher LIPUS intensity of 3.0 w/cm^2, the disruption of tight junction proteins in the NMB model was irreversible (Fig. 2k and Supplementary Fig. 8e). These dates demonstrates that LIPUS at 1.5 w/cm^2 intensity can safely and reversibly disrupt NMB tight junction proteins, allowing iMPC to penetrate the NMB, reduce T-cell exhaustion, and improve the inhibition of GL261 cells.

Furthermore, compared to the group without iMPC treatment (-), the cell viability of HNEpC, bEnd.3, SH-SY5Y, PC12, and Neuro-2a cells in the iMPC-treated group (+) showed no significant changes (Supplementary Fig. 8f). This suggests that iMPC, as an anti-tumor immune-enhancing nanomedication, exhibits favorable biosafety.

The description has been added (page 59 line 1478-1486) as: The NMB model derived from HNEpCs tracked trans endothelial electrical resistance (TEER) values over an 8-day period using a commercial ohmmeter (EVOM, WPI, USA). The TEER value for each well was calculated using the formula: TEER(cells) = (TEER(total) − TEER(blank)) / culture area.

Cy5-labeled iMPC were placed in the upper chamber of the NMB model migration apparatus. The fluorescence signal intensity in the lower chamber was measured and statistically analyzed using an *IVIS* imaging system after 60 seconds of LIPUS exposure at varying acoustic intensities (0, 0.5, 1.5, 3.0, 4.0, and 5.0 w/cm²). TEER values were measured using a commercial ohmmeter (EVOM, WPI, USA).

7 The legend for Fig. 2g is missing.

Re: Thank you for your reminder. The legend for Fig. 2g has been added.

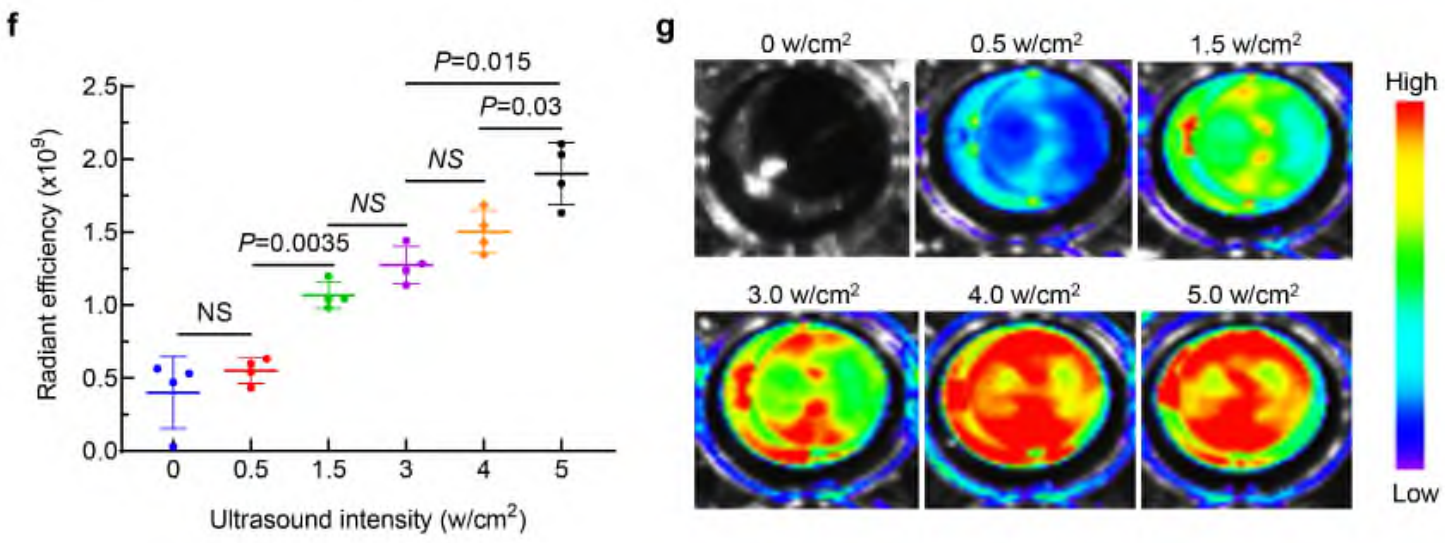

Figure 2f-g, *In vivo* imaging system evaluation of the amount of iMPC penetration through the nasal mucosal barrier model after 60 seconds of LFUS exposure at intensities of 0 w/cm², 0.5 w/cm², 1.5 w/cm², 3.0 w/cm², 4.0 w/cm², and 5.0 w/cm².

8 In Fig. 3a, the image appears to show the whole mouse body, but it is difficult to identify which regions correspond to which organs. Please modify the figure to make this clear.

Re: Thank you for your comment. We have revised Fig. 3a.

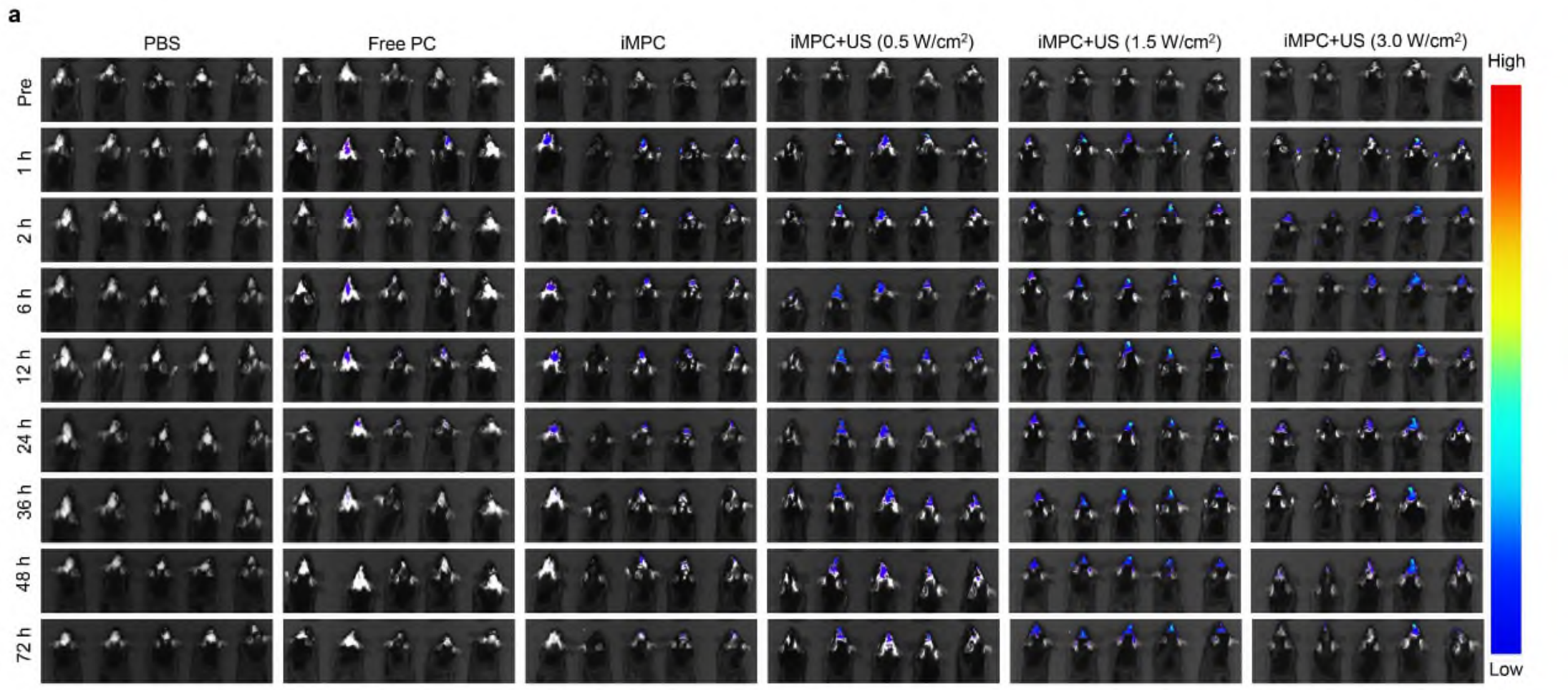

Figure 3. LIPUS improves iMPC nose-to-brain delivery efficiency *in vivo*. a, The distribution of Cy5 NHS-labeled aPD-L1 after various treatments.

9 In Fig. 3, why is the permeability reduced at 3 W ultrasound intensity compared to 1.5 W? If TJ protein expression is further decreased, permeability should be more enhanced.

Re: Thank you for your comments. Unlike the LIPUS at 1.5 w/cm² treatment that safely reverses open NMB, the LIPUS at 3.0 w/cm² applied to mouse nasal mucosa induces significant "thermal effects" and "inflammatory responses," leading to irreversible damage to the nasal mucosa. This limitation restricts the efficiency of nasal-to-brain delivery for iMPC.

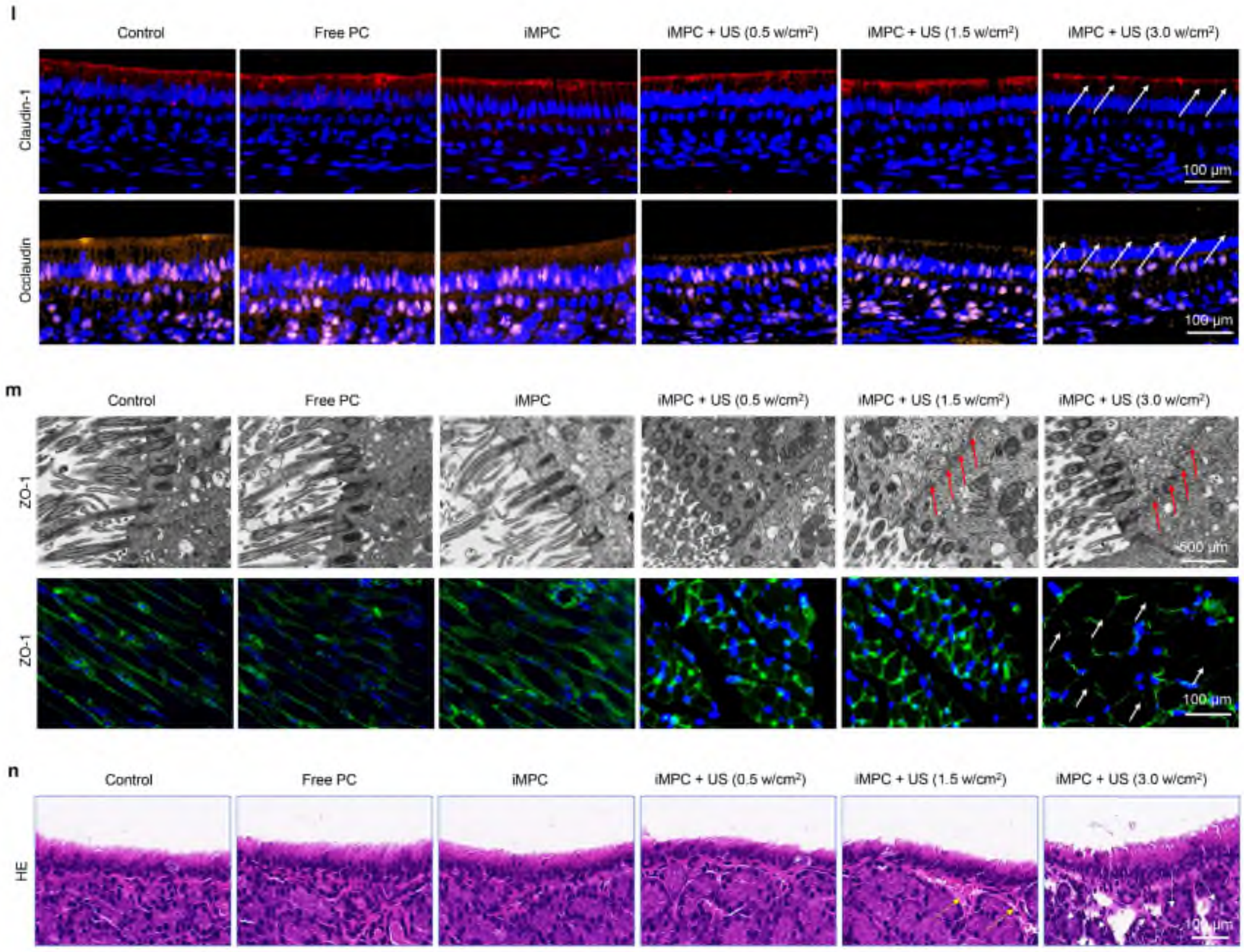

Figure 4l, Changes in tight junction proteins Occludin and Claudin-1 (blue: DAPI; red: CY3-Claudin-1; yellow: SpRed-Occludin). **m**, Alterations in tight junction proteins of the paracellular pathway (blue: DAPI; green: Alexa Fluor 488-ZO-1). **n**, Inflammatory cell infiltration after various treatments.

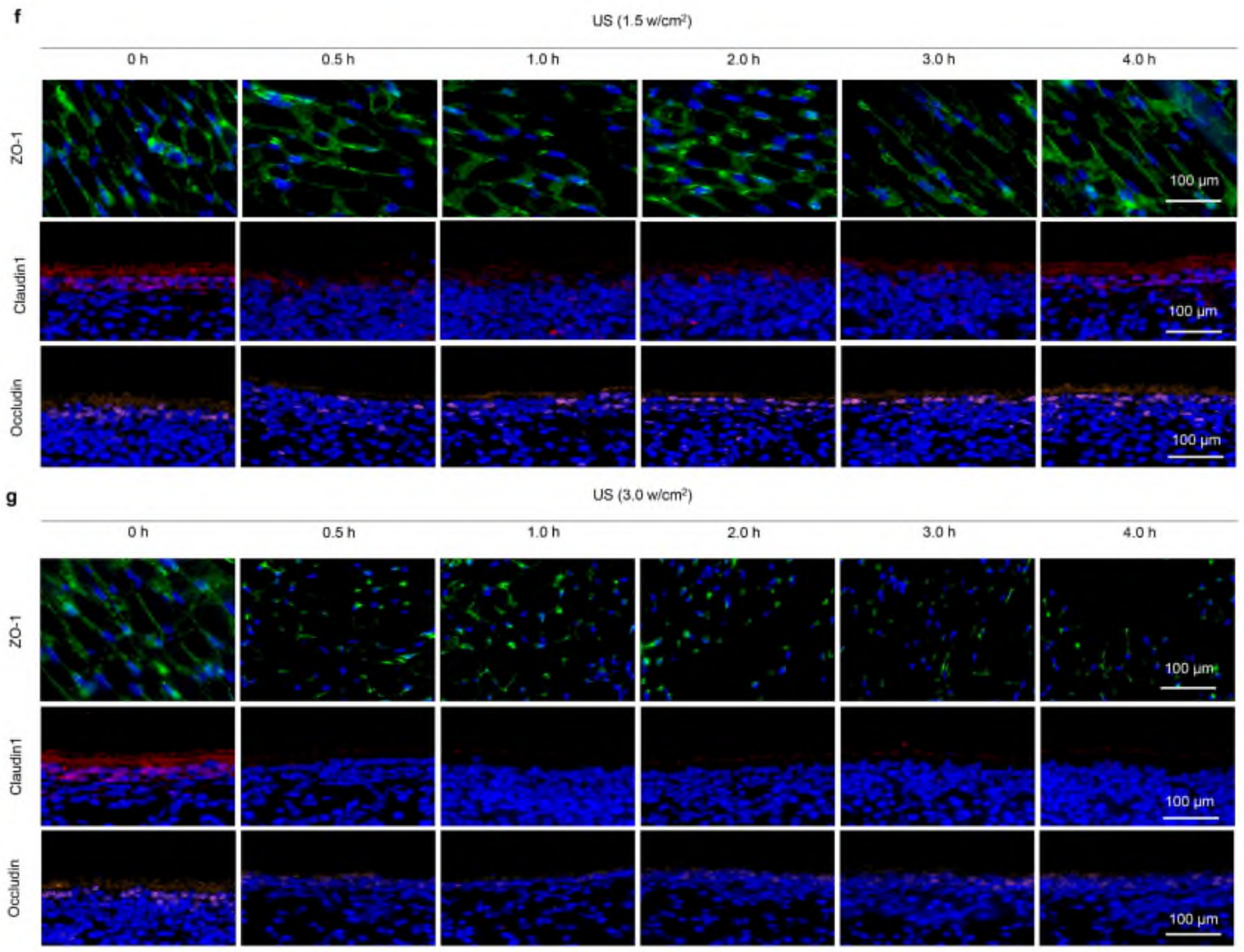

Figure 5f-g, Occludin, Claudin-1, and ZO-1 changes within 4 hours following LFUS exposure at 1.5 w/cm² or 3.0 w/cm² for 60 seconds (blue: DAPI; red: CY3-Claudin-1; yellow: SpRed-Occludin).

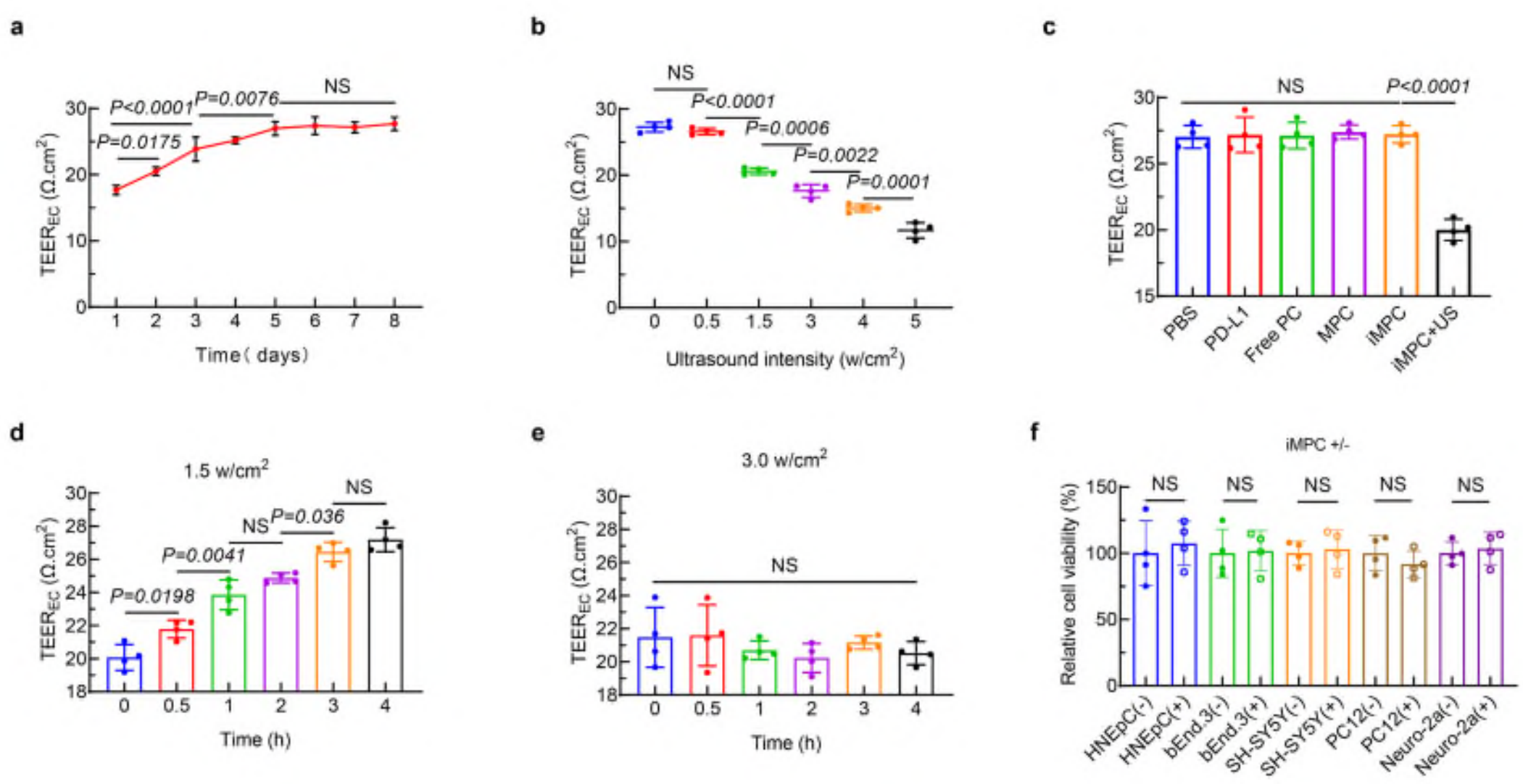

Supplementary Figure 8. Trans-epithelial electrical resistance (TEER) values in the nasal mucosal barrier (NMB) model. a, Changes in TEER values of the human nasal epithelial cell (HNEpC)-based NMB model (over 8 days). **b,** Changes in TEER values of the NMB model following treatment with different ultrasound intensities. **c,** Changes in TEER values of the NMB model after six different treatments. **d,** Ultrasonic intensity of 1.5 w/cm² was applied to NMB models, with changes in TEER values monitored over 4 hours. **e,** Ultrasonic intensity of 3.0 w/cm² was applied to NMB models, with changes in TEER values monitored over 4 hours. **f,** Cell viability

of HNEpC, bEnd.3, SH-SY5Y, PC12, and Neuro-2a cells after treatment with iMPC nanoformulation. n = 4 biologically independent mice per group for data in **a-f**. Data are expressed as the mean ± s.d. Statistical significances were determined using one-way ANOVA with Dunnett's post hoc test. Comparisons were performed between different groups in **a-f**. Not significant (NS) is $P \geq 0.05$, and significant P values are shown.

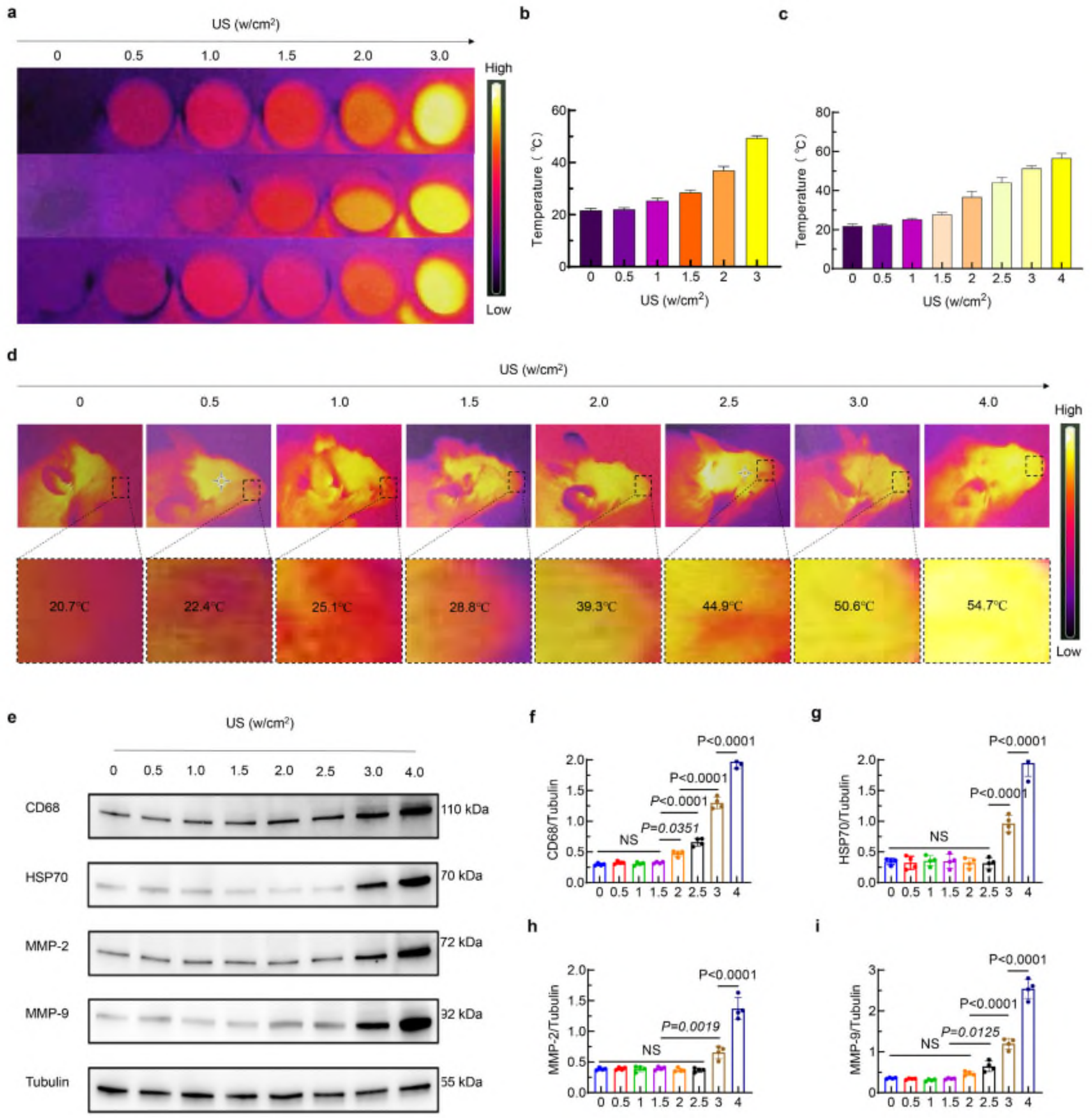

Supplementary Figure 17. Thermal injury of LIPUS on nasal mucosa. a-b, Temperature changes in the nasal mucosal barrier (NMB) model constructed from human nasal epithelial cells (HNEpC). **c-d,** Regional temperature changes in mouse nasal mucosa. **e-h,** Expression levels of HSP70, CD68, and MMP-9. Gray-scale analysis was performed using ImageJ software. n = 3 biologically independent mice per group for data in **a-c**. n = 4 biologically independent mice per group for data in **f-i**. Data are expressed as the mean ± s.d. Statistical significances were determined using one-way ANOVA with Dunnett's post hoc test. Comparisons were performed between different groups in **a-c** and **f-i**. Not significant (NS) is $P \geq 0.05$, and significant P values are shown.

The description has been added (page 28 line 675-679) as: Hematoxylin and eosin (H&E) staining of the nasal mucosa revealed significant inflammatory cell infiltration

when LIPUS at 3.0 w/cm² was combined with iMPC (Fig. 4n). LIPUS at an intensity of 3.0 w/cm² may excessively disrupt the NMB, heightening the risk of inflammatory cell infiltration. It highlights the primary reason for the greater efficiency of LIPUS in nose-to-brain delivery at 1.5 w/cm² rather than 3.0 w/cm².

The description has been added (page 28 line 711-718) as: We evaluated the impact of LIPUS at intensities of 1.5 w/cm² and 3.0 w/cm² on NMB-associated proteins to identify the optimal US intensity range for safely and reversibly opening the NMB. The study found that LIPUS at 1.5 w/cm² intensity disrupted ZO-1, Claudin1, and occludin, but these effects were reversible within 4 hours. (Fig. 5a-f). After LIPUS treatment at an intensity of 3.0 w/cm², the disruption of ZO-1, Claudin1, and occludin was not restored (Figure 5g). This may be due to the fact that LIPUS with too high ultrasound intensity caused significant structural disruption of the NMB and was unable to restore the normal structure.

The description has been added (page 16 line 395-404) as: The transendothelial electrical resistance (TEER) values in the NMB model stabilized by the fifth day, as shown in Supplementary Fig. 8a. An *in vivo* imaging system (*IVIS*) assessment demonstrated that the aPD-L1 content in the lower chamber progressively rose with increased US intensity (Fig. 2e-g). This conclusion was further validated by quantitative measurements using a microplate reader (Fig. 2e-g). TEER measurement results indicate that the TEER value of the NMB model gradually decreases with increasing US intensity (Supplementary Fig. 8b). The findings suggest a significant correlation between the extent of NMB disruption and ultrasonic energy, highlighting the need to identify a safe ultrasound intensity range to ensure NMB damage reversibility.

The description has been added (page 26 line 619-648) as: Sustained LIPUS exposure often induces varying degrees of "thermal effects."[8,9,11] Thermal imaging results indicate that the temperature of the NMB model increases with rising US intensity (Supplementary Fig. 17a-b). The NMB model attained a temperature of 29.3°C after 60 seconds of continuous ultrasound exposure at an intensity of 1.5 w/cm² (Supplementary Fig. 17a-b). At an US intensity of 3.0 w/cm² with 60 seconds of continuous exposure, the NMB model reached a temperature of 50.1°C (Supplementary

Fig. 17a-b).

In vivo experiments demonstrated that the temperature in the mouse nasal mucosa region gradually increased with rising US intensity (Supplementary Fig. 17c-d). Specifically, when US intensity reached 3.0 w/cm² and was applied continuously for 60 seconds, the temperature in the mouse nasal mucosa region reached 50.6°C (Supplementary Fig. 17c-d). At an US intensity of 4.0 w/cm² with a 60-second exposure duration, the temperature in the mouse nasal mucosa region reached 54.7°C (Supplementary Fig. 17c-d). These data indicate that sustained exposure to US at certain intensities may cause thermal injury to nasal mucosal tissue.

WB analysis indicated that CD68 levels in the nasal mucosal tissue of mice exposed to 1.5 w/cm² US were comparable to the control group (0 w/cm²), whereas exposure to 3.0 w/cm² US resulted in a 4.41-fold increase in CD68 levels (Supplementary Fig. 17e-f). HSP70 levels in the nasal mucosa of mice exposed to 1.5 w/cm² ultrasound showed no significant change compared to the control group (0 w/cm²), whereas exposure to 3.0 w/cm² resulted in a 2.83-fold increase (Supplementary Fig. 17g).

The US group (1.5 w/cm²) exhibited no significant differences in matrix metalloproteinase-2 (MMP-2) and matrix metalloproteinase-9 (MMP-9) levels in nasal mucosal tissue compared to the control group (0 w/cm²) (Supplementary Fig. 17h-i). MMP-2 and MMP-9 levels in the nasal mucosal tissue of the US (3.0 w/cm²) group rose by 1.71-fold and 3.38-fold, respectively (Supplementary Fig. 17h-i). These data indicate that US treatment at an intensity of 3.0 w/cm² induces “thermal injury” and inflammatory infiltration in the nasal mucosal tissue region of mice. LIPUS at 1.5 w/cm² intensity did not significantly alter mouse nasal mucosal tissue structure or cause inflammatory infiltration.

10 In Fig. 4j, please explain why Claudin-1 expression decreases in the iMPC-only group.

Re: Thank you for your comments. The positively charged iMPC exhibits a certain degree of suppression on Claudin-1 expression, but LIPUS is the key factor influencing the levels of NMB tight junction proteins.

11 The superiority of iRGD peptide modification is not demonstrated in vivo. In addition, the MPC + US group is missing in Fig. S5.

Re: Thank you for your valuable comments. By establishing GBM mice model, we demonstrated that LIPUS-mediated iMPC (iRGD-peptide-modified MPC) nasal-to-brain delivery significantly enhances delivery efficiency compared to LIPUS-mediated MPC nasal-to-brain delivery. In addition, the MPC + US group has been added in Fig. S5.

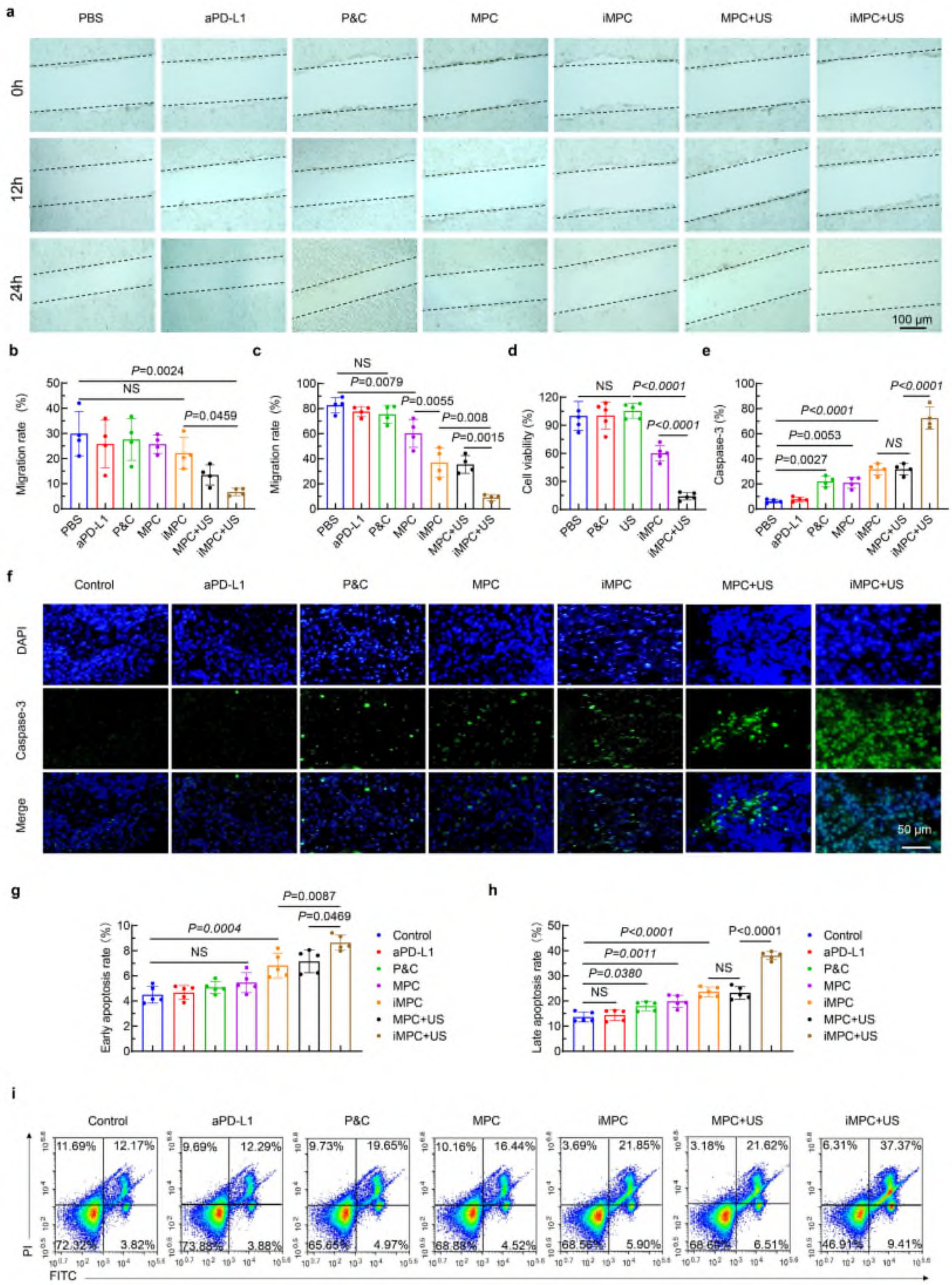

Supplementary Figure 5. Low-intensity pulsed ultrasound (LIPUS)-triggered nasal mucosal barrier opening combined with iMPC inhibits GL261 cell growth *in vitro*. a-c, Evaluation of the invasiveness of GL261 cells using a scratch assay after different treatments in the upper chamber of the Transwell. **d,** Effect of LIPUS on GL261 cell viability. **e-f,** Analysis of apoptosis in GL261 cells using the caspase-3 apoptosis fluorescence assay. **g-i**, The early apoptosis rate, late apoptosis rate, and total apoptosis rate of GL261 cells after 24 hours of different treatments. n = 4 biologically independent mice per group for data in **b, c,** and **e**. n = 5 biologically independent mice per group for data in **d** and **g-h**. Data are expressed as the mean ± s.d. Statistical significances were determined using one-way ANOVA with Dunnett's post hoc test. Comparisons were performed between

different groups in **b-e** and **g-h**. Not significant (NS) is $P \geq 0.05$, and significant P values are shown.

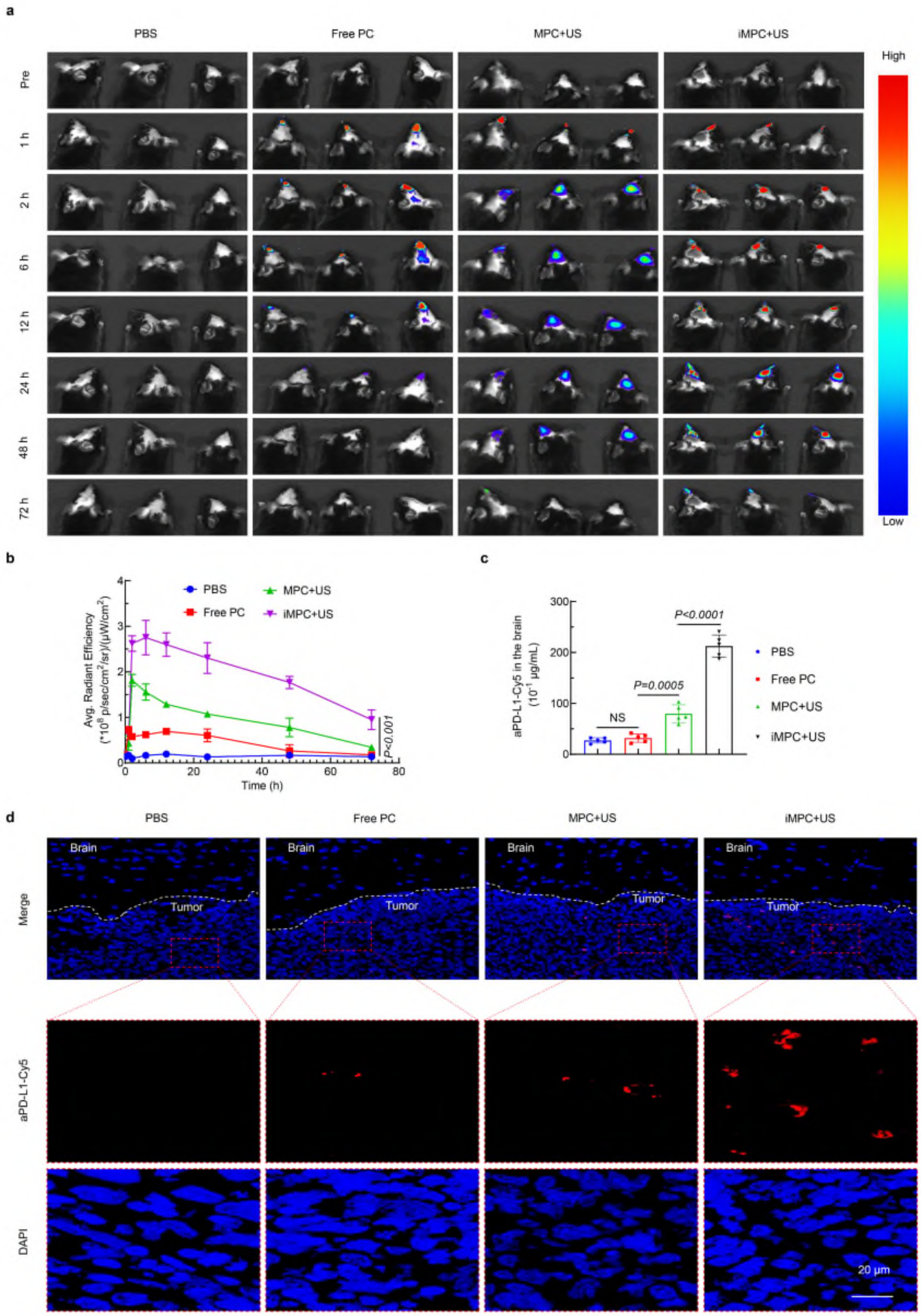

Supplementary Figure 19. iRGD-modified MPC enhances its targeted tumor delivery efficiency. a, *In vivo* imaging of GBM mice subjected to four different treatments. **b,** Quantification of fluorescence signals. **c,** Fluorescence intensity of aPD-L1 in brain tissues, aPD-L1 labeled with Cy5 NHS. **d,** Fluorescence signals of aPD-L1 in tumor tissues of GBM mice. n = 3 biologically independent mice per group for data in **a-b**. n = 5 biologically independent mice per group for data

in **c**. Data are expressed as the mean ± s.d. Statistical significances were determined using one-way ANOVA with Dunnett's post hoc test. Comparisons were performed between different groups in **b-c**. Not significant (NS) is $P \geq 0.05$, and significant P values are shown.

The description has been added (page 13 line 326-379) as: The impact of US+iMPC on GL261 cells was further assessed using scratch tests, flow-through apoptosis assays, and immunofluorescence assays. Scratch test results showed that, compared with free iMPC treatment, US+iMPC treatment resulted in a 4.05-fold decrease in GL261 cell migration rate (Supplementary Fig. 5a-c). GL261 cell migration remained largely unchanged in both the free aPD-L1 and P&C (free aPD-L1 & carvedilol) groups relative to the control group, primarily due to NMB hindering drug permeation (Supplementary Fig. 5a-c).

Additional research indicated that GL261 cell viability remained unchanged in the LIPUS-only group relative to the PBS group (Supplementary Fig. 5d). Compared to the Free P&C group, GL261 cell viability decreased by 59.93% in the iMPC group and by 85.92% in the iMPC+US group (Supplementary Fig. 5d). Compared with the LIPUS group, GL261 cell viability decreased by 87.48% in the iMPC+US group (Supplementary Fig. 5d). The data suggest that LIPUS at an intensity of 1.5 w/cm² does not impact the viability of GL261 cells.

Caspase-3 immunofluorescence and flow cytometry analyses revealed a 2 (Fig. 29-fold increase in the apoptosis rate of GL261 cells following US+iMPC treatment compared to free iMPC treatment (Supplementary Fig. 5d-e). Likewise, caspase-3 expression and apoptosis rate in GL261 cells remained unchanged in both the free aPD-L1 and P&C groups compared to the control group (Supplementary Fig. 5d-f).

Flow cytometry analysis indicated that the early apoptosis rate of GL261 cells increased by 112.95% in the free PC group and by 192.17% in the iMPC+US group compared to the control group (Fig. 2d and Supplementary Fig. 5g-i). The late apoptosis rate of GL261 cells increased by 131.87% in the free PC group and by 279.32% in the iMPC+US group compared to the control group (Fig. 2d and Supplementary Fig. 5g-i). The total apoptosis rate of GL261 cells increased by 134.81% in the iMPC group and by 202.46% in the iMPC+US group compared to the free PC group (Fig. 2d and Supplementary Fig. 5g-i).

The description has been added (page 58 line 1467-1477) as: Seven treatment conditions were applied: control group, aPD-L1 group, P&C group, MPC group, iMPC group, MPC+US group and iMPC+US group. The aPD-L1 concentration was kept at 10 μg/mL, with carvedilol administered at 10 μM. Adherent GL261 cells were digested with 0.25% trypsin (BioChannel Biological Technology Co., Ltd., Nanjing, China), and digestion was halted using culture medium. All cell lines were confirmed free of mycoplasma contamination and regularly treated with a mycoplasma removal agent (VivaCell, Shanghai, China). An Annexin V-FITC/PI apoptosis detection kit (abs50001, Absin, Shanghai, China) facilitated the analysis. Caspase-3 (Abcam) was detected via immunofluorescence staining. The cell migration rate was determined using the formula: Cell Migration Rate (%) = [(Initial Scratch Area − Current Scratch Area) / Initial Scratch Area]*100%.

The description has been added (page 28 line 700-710) as: Further comparison of GBM targeting efficiency after iRGD-modified MPC revealed significantly enhanced tumor region signals in iMPC+US group compared to the MPC+US group (Supplementary Fig. 19a-b). Quantitative analysis using a multifunctional microplate reader revealed that aPD-L1 levels in the GBM mouse tumor region were 2.49 times higher in the MPC+US group compared to the Free PC group (Supplementary Fig. 19c). The iMPC+US group exhibited a 2.67-fold increase in aPD-L1 levels in the GBM mouse tumor region compared to the MPC+US group (Supplementary Fig. 19c). Confocal microscopy revealed significantly increased aPD-L1 distribution in tumor regions of iMPC+US GBM mice compared to MPC+US mice (Supplementary Fig. 19d). These findings demonstrate that iRGD-modified MPCs significantly enhance their targeting efficiency to GBM regions.

The description has been added (page 28 line 1648-1660) as: Further evaluation of tumor targeting by iRGD-modified iMPCs in vivo. C57 mice (6–8 weeks old, 20–22 g) were randomly assigned by sex for intranasal administration in the experiment. The experiments comprised four groups: PBS control, Free PC, MPC+US, and iMPC+US. aPD-L1 was labeled with Cy5-NHS (Starleaf Biological Technology Co, Nanjing, China). The dosage of aPD-L1 was administered at 30 μg for every 20 g of body weight.

LIPUS was directed at the olfactory epithelium within the mouse nasal cavity. Ultrasound parameters included a 1.5 w/cm² intensity, 30% duty cycle, 1 MHz center frequency, and a 60-second duration. The efficiency of nasal-to-brain drug delivery was evaluated using *IVIS* imaging at intervals of 0, 1, 2, 6, 12, 24, 48, and 72 hours post-treatment for all groups. Fluorescence signals were statistically analyzed using ImageJ software (ImageJ Software Inc., USA). Six hours after treatment, frozen sections were prepared, stained with DAPI, and imaged using a Zeiss LSM 510 laser scanning fluorescence confocal microscope to observe aPD-L1-Cy5 distribution.

12 Fig. S10 appears to be important data showing that the reduction of barrier function is transient and reversible. Since reversibility is also claimed in the Abstract, it is recommended to move this figure to the main text.

Re: Thank you for your valuable suggestion. We have moved “Fig. S10” to the main text as “Figure 5.”

[Figure Redacted]

Figure 5. LIPUS induces reversible opening of nasal mucosal barrier (NMB). a, Diagram of LFUS-mediated NMB opening. **b-e,** Occludin, Claudin-1, and ZO-1 variations within 4 hours after exposure to LFUS at an intensity of 1.5 w/cm² for 60 seconds. **f-g,** Occludin, Claudin-1, and ZO-1 changes within 4 hours following LFUS exposure at 1.5 w/cm² or 3.0 w/cm² for 60 seconds (blue: DAPI; red: CY3-Claudin-1; yellow: SpRed-Occludin). n = 3 biologically independent mice per group for data in **c-e**. Data are expressed as the mean ± s.d. Statistical significances were determined using one-way ANOVA with Dunnett's post hoc test. Comparisons were performed between different groups in **c-e**. Not significant (NS) is $P \geq 0.05$, and significant P val ues are shown.

13 In the Methods section, the irradiation site of the ultrasound should be described in detail. Furthermore, 1 MHz is generally considered a standard ultrasound frequency, whereas "low-frequency ultrasound" usually refers to below 1 MHz. The terminology, including the title, should therefore be revised accordingly.

Re: Thank you for your professional suggestions. In the Methods section, we have further detailed the ultrasound irradiation site (olfactory epithelium within the nasal cavity). "Low-frequency ultrasound (LFUS)" has been corrected to "low-intensity pulsed ultrasound (LIPUS)."

The description has been added (page 62 line 1557-1574) as: To investigate the in vivo distribution of aPD-L1, 6-8-week-old C57 mice (20-22 g) were selected for the experiment, with males and females randomly assigned. The experiment involved nasal administration and was divided into four groups: control, aPD-L1, iMPC, and iMPC+US. LIPUS targeted the olfactory epithelium within the nasal cavity. aPD-L1 was labeled with Cy5-NHS (Starleaf Biological Technology Co, Nanjing, China). The aPD-L1 dosage was administered at 30 μg per 20 g of body weight. *IVIS* imaging, conducted six hours later, identified fluorescent signals in the heart, liver, spleen, lungs, kidneys, and brain across all four groups. Fluorescence signal values were statistically analyzed using ImageJ software (ImageJ Software Inc., USA).

To investigate the distribution of aPD-L1 *in vivo*, 6-8-week-old C57 mice (20-22 g) were selected for the experiment, with males and females randomly assigned. The experiment involved nasal administration and was divided into four groups: control, aPD-L1, iMPC, and iMPC+US. LIPUS targeted the olfactory epithelium within the nasal cavity. aPD-L1 was labeled with Cy5-NHS (Starleaf Biological Technology Co, Nanjing, China). The aPD-L1 dosage was administered at 30 μg per 20 g of body weight. Six hours later, *IVIS* imaging revealed fluorescent signals in the heart, liver, spleen, lungs, kidneys, and brain across all four groups. Fluorescence signal values were statistically analyzed using ImageJ software (ImageJ Software Inc., USA).

The description has been added (page 62 line 1582-1594) as: Further evaluate the clearance rates of carvedilol and aPD-L1 in the lungs, kidneys, and liver. Select 6-8-week-old C57 mice (20-22 g) for the experiment, randomly assigning males and

females, and administer the drugs intranasally. LIPUS was administered to the olfactory epithelium in the mouse nasal cavity. Experimental groups included: carvedilol group, aPD-L1 group, iMPC group, and iMPC+US group. aPD-L1 was administered at 30 μg per 20 g of body weight, while carvedilol was given at 4 μg per 20 g of body weight. Carvedilol and aPD-L1 were conjugated with Cy5-NHS (Starleaf Biological Technology Co, Nanjing, China). Ultrasound parameters were set to 1.5 w/cm^2 with a 30% duty cycle, a center frequency of 1 MHz, and a duration of 60 seconds. Lungs, kidneys, and livers were collected and homogenized from mice at 0, 1, 2, 6, 12, 24, 48, and 72 hours post-treatment. Fluorescence signal intensity was measured using *IVIS*, and quantitatively analyzed with a PerkinElmer multifunctional microplate reader (Boston, MA, USA).

The description has been added (page 65 line 1648-1678) as: Further evaluation of tumor targeting by iRGD-modified iMPCs in vivo. C57 mice (6–8 weeks old, 20–22 g) were randomly assigned by sex for intranasal administration in the experiment. The experiments comprised four groups: PBS control, Free PC, MPC+US, and iMPC+US. aPD-L1 was labeled with Cy5-NHS (Starleaf Biological Technology Co, Nanjing, China). The dosage of aPD-L1 was administered at 30 μg for every 20 g of body weight. LIPUS was directed at the olfactory epithelium within the mouse nasal cavity. Ultrasound parameters included a 1.5 w/cm^2 intensity, 30% duty cycle, 1 MHz center frequency, and a 60-second duration. The efficiency of nasal-to-brain drug delivery was evaluated using *IVIS* imaging at intervals of 0, 1, 2, 6, 12, 24, 48, and 72 hours post-treatment for all groups. Fluorescence signals were statistically analyzed using ImageJ software (ImageJ Software Inc., USA). Six hours after treatment, frozen sections were prepared, stained with DAPI, and imaged using a Zeiss LSM 510 laser scanning fluorescence confocal microscope to observe aPD-L1-Cy5 distribution.

LIPUS-induced thermal injury to the nasal mucosa

Further evaluation of the thermal effect of LIPUS on NMB. HNEpCs were seeded at $1*10^6$ cells/mL in 500 μL into the upper chamber of a 24-well Transwell® migration insert (400 nm pore size; Millipore, MA, USA). After conventional culture for 5 days, LIPUS treatment was applied at intensities of 0, 0.5, 1.0, 1.5, 2.0, and 3.0 w/cm^2. The

other US parameters were as follows: duty cycle 30%, center frequency 1 MHz, and duration 60 s. Subsequently, temperature changes in the NMB were assessed using an infrared thermal imager (TIS65, FLUKE, USA) and statistically analyzed.

The olfactory epithelium in the nasal cavity of 6-8-week-old C57 mice (20-22 g) underwent LIPUS treatment at ultrasound intensities of 0, 0.5, 1.0, 1.5, 2.0, 2.5, 3.0, and 4.0 w/cm^2, with male and female mice randomly assigned. The US parameters included a 30% duty cycle, 1 MHz center frequency, and 60-second duration. Temperature changes in the nasal cavity's olfactory region were then measured with an infrared thermal imager (TIS65, FLUKE, USA) and analyzed statistically. Western blot analysis was conducted to assess the expression of HSP70, CD68, MMP-2, MMP-9, and Tubulin in the nasal mucosa of the olfactory epithelium in C57 mice following LIPUS treatment at varying ultrasound intensities, using antibodies from Abcam, UK. Statistical analysis of gray values was conducted using ImageJ software (ImageJ Software Inc., USA).

Reviewer #3 (Remarks to the Author):

Dong et al. and their team developed an immune-activating nanosystem for the intervention of glioblastoma (GBM). This therapeutic system was created by loading extracellular vesicles (EVs) with carvedilol and aPD-L1 through processes of freeze-drying and extrusion, followed by surface modification using the targeting peptide iRGD. The article emphasizes that overcoming the blood-brain barrier (BBB) using low-frequency ultrasound (LFUS) alone marks a significant advancement. This innovation simplifies the procedure and reduces the risks associated with using microbubbles. Below are some suggestions for improving the manuscript.

Majors:

1. There are numerous typos and unclear sentences throughout the text. The authors should conduct another round of proofreading to address these issues. Additionally, the article lacks clarity in scientific writing and requires significant improvement. Specific examples of these issues can be found in the minor comments provided.

Re: Thank you for your careful suggestions. We have thoroughly revised the manuscript and completed another round of proofreading.

2. The current title, ‘Low frequency ultrasound-mediated nose-to-brain drug delivery,’ is very general. The title needs to be modified/elaborated to provide the reader with a more detailed description of the proposed GBM intervention.

Re: Thank you for your comment. The title has been corrected from “Low frequency ultrasound-mediated nose-to-brain drug delivery” to “Low-intensity pulsed ultrasound-mediated nose-to-brain co-delivery of β-blockers and immune checkpoint inhibitors reduces T cells exhaustion to enhance glioblastoma immunotherapy”.

3. In the introduction, the authors don't mention the current challenges faced by the LFUS in the NMB application.

Re: Thank you for your suggestions. The description regarding “the current challenges faced by the LFUS in the NMB application” has been added to the “Introduction” section.

The description has been added (page 3 line 64-79) as: Low-intensity pulsed ultrasound (LIPUS), a non-invasive physical energy technique, has shown the ability to temporarily open biological barriers[7]. Ultrasonic mechanical effects are generated by producing an electrical signal first, which is then amplified and fed to the ultrasonic horn[7-9]. Upon arriving at the horn, this electrical signal is transformed into mechanical waves by piezoelectric crystals via the transducer tip, and the waves are thereafter propagated to the target medium[8,9]. LIPUS can penetrate biological barriers due to its mechanical effects, including cavitation, viscous stress, and acoustic radiation[7-11]. For instance, drug permeation through the stratum corneum can be effectively enhanced under the acoustic energy of LIPUS[11]. Moreover, LIPUS has a promising therapeutic efficacy in clinical trials for brain diseases[12]. Microbubbles (MBs) amplify the acoustic energy of LIPUS to overcome 70-90% attenuation caused by the skull, enabling safe and reversible BBB opening[13]. Notably, NMB arises from the direct exposure of the nasal mucosa to the nasal cavity, devoid of physical skull barriers[14,15]. Currently, there are no studies documenting the direct use of LIPUS in NMB[7-9,15,16]. This study found that LIPUS can transiently loosen tight junctions of NMB via mechanical vibration, without requiring MBs-enhanced acoustic energy.

References

7. Ruan, S. et al. Microneedle-mediated nose-to-brain drug delivery for improved Alzheimer's disease treatment. *J Control Release* **366**, 712-731 (2024).
8. Ma, Z. et al. Controlled tough bioadhesion mediated by ultrasound. *Science* **377**, 751-755 (2022).
9. Schoellhammer, C. M. et al. Applicability and safety of dual-frequency ultrasonic treatment for the transdermal delivery of drugs. *J Control Release* **202**, 93-100 (2015).
10. Guo, J. et al. Bio-barrier-adaptable biomimetic nanomedicines combined with ultrasound for enhanced cancer therapy. *Signal Transduct Target Ther* **10**, 137 (2025).
11. Yu, C. C. et al. A conformable ultrasound patch for cavitation-enhanced transdermal cosmeceutical delivery. *Advanced materials (Deerfield Beach, Fla.)* **35**, e2300066 (2023).
12. Sonabend, A. M. et al. Repeated blood-brain barrier opening with an implantable ultrasound device for delivery of albumin-bound paclitaxel in patients with recurrent glioblastoma: a phase 1 trial. *Lancet Oncol.* **24**, 509-522 (2023).
13. Dong, L. et al. Open-source throttling of $CD8^+$ T cells in the brain with low-intensity focused ultrasound-guided sequential delivery of CXCL10, IL-2, and aPD-L1 for glioblastoma immunotherapy. *Advanced materials (Deerfield Beach, Fla.)* **36**, e2407235 (2024).
14. Chen, Y. et al. Intranasal drug delivery: The interaction between nanoparticles and the nose-to-brain pathway. *Adv. Drug Del. Rev.* **207**, 115196 (2024).

15. Wang, G. et al. Tailored borneol-modified lipid nanoparticles nasal spray for enhanced nose-to-brain delivery to central nervous system diseases. *ACS Nano* **18**, 23684-23701 (2024).
16. Wang, J. et al. Ultrasound-mediated blood-brain barrier opening: An effective drug delivery system for theranostics of brain diseases. *Adv. Drug Del. Rev.* **190**, 114539 (2022).

4. What is ‘NE is associated with T-cell exhaustion and worsened prognosis in the GBM mice stress model’? How was it established?

Re: Thank you for your comment. “NE is associated with T-cell exhaustion and worsened prognosis in the GBM mice stress model” has been revised to “Elevated NE levels in the TME of GBM stress model mice.” We have further added the description of the GBM mice stress model.

The description has been added (page 68 line 1743-1749) as: **Chronic restraint stress (CRs)** C57BL6 GBM mice were randomly allocated into control and stress groups, wherein mice in the stress group were subjected to daily restraint stress by being placed in well-venticated plastic restrainers that limited movement without causing compression for 6 h per day, to minimize non-experimental stress, all handling procedures were performed with extreme care, and potentially stress-inducing procedures (including injections and blood collection) were conducted under mild isoflurane anesthesia.

5. In the cell viability test (Fig. 2b), is there any result indicating that the lower cell viability was caused by LFUS+iMPC instead of LFUS alone? The authors switch the LFUS? The description of the cell line differs in the experimental section.

Re: Thank you for your critical comments. We have added relevant experiments, which demonstrate that LIPUS alone has no significant effect on GL261 cell viability compared to the control group. Depending on the specific experimental requirements, we selected GL261, GL261-GFP (a GL261 cell line expressing GFP), and GL261-Luc (a GL261 cell line expressing luciferase) respectively.

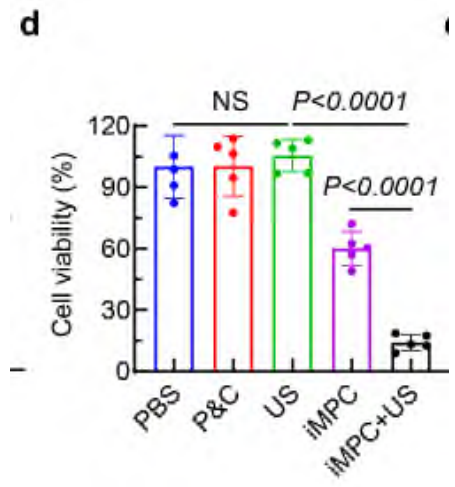

Supplementary Figure 5d, Effect of LIPUS on GL261 cell viability.

The description has been added (page 15 line 360-366) as: Additional research indicated that GL261 cell viability remained unchanged in the LIPUS-only group relative to the PBS group (Supplementary Fig. 5d). Compared to the Free P&C group, GL261 cell viability decreased by 59.93% in the iMPC group and by 85.92% in the iMPC+US group (Supplementary Fig. 5d). Compared with the LIPUS group, GL261 cell viability decreased by 87.48% in the iMPC+US group (Supplementary Fig. 5d). The data suggest that LIPUS at an intensity of 1.5 w/cm² does not impact the viability of GL261 cells.

The description has been added (page 58 line 1458-1466) as: Evaluate the effect of LIPUS alone on GL261 cell viability. HNEpCs ($1*10^6$ cells/mL) were seeded in the upper chamber of a 24-well Transwell® migration insert (400 nm pore size; Millipore, MA, USA), and an equal volume of GL261 cells (IMMOCELL, Xiamen, China) at the same density was placed in the lower chamber. The experiment was divided into five groups: PBS group, P&C (aPD-L1+carvedilol) group, US (1.5 w/cm²) group, iMPC group, and iMPC+US group. The aPD-L1 concentration was kept at 10 µg/mL, with carvedilol administered at 10 µM. The ultrasound parameters included a 30% duty cycle, a center frequency of 1 MHz, and a duration of 60 seconds. GL261 cell viability was evaluated using the CCK-8 assay.

6. Fig. 2e and 2g reveal significant accumulation of aPD-L1 in the lower chamber, and Fig. 2h-k, which can be attributed to disruption of the tight junction. But the paper doesn't give mechanistic insights into the data shown in Figure 2. Elucidate more on the phenomenon.

Re: Thank you for your comments. We further supplemented our *in vitro* experiments, which demonstrated that LIPUS not only disrupts NMB-associated tight junction

proteins through mechanical force but also modulates their levels by activating the cGMP/PKG/RhoA/RocK signaling pathway.

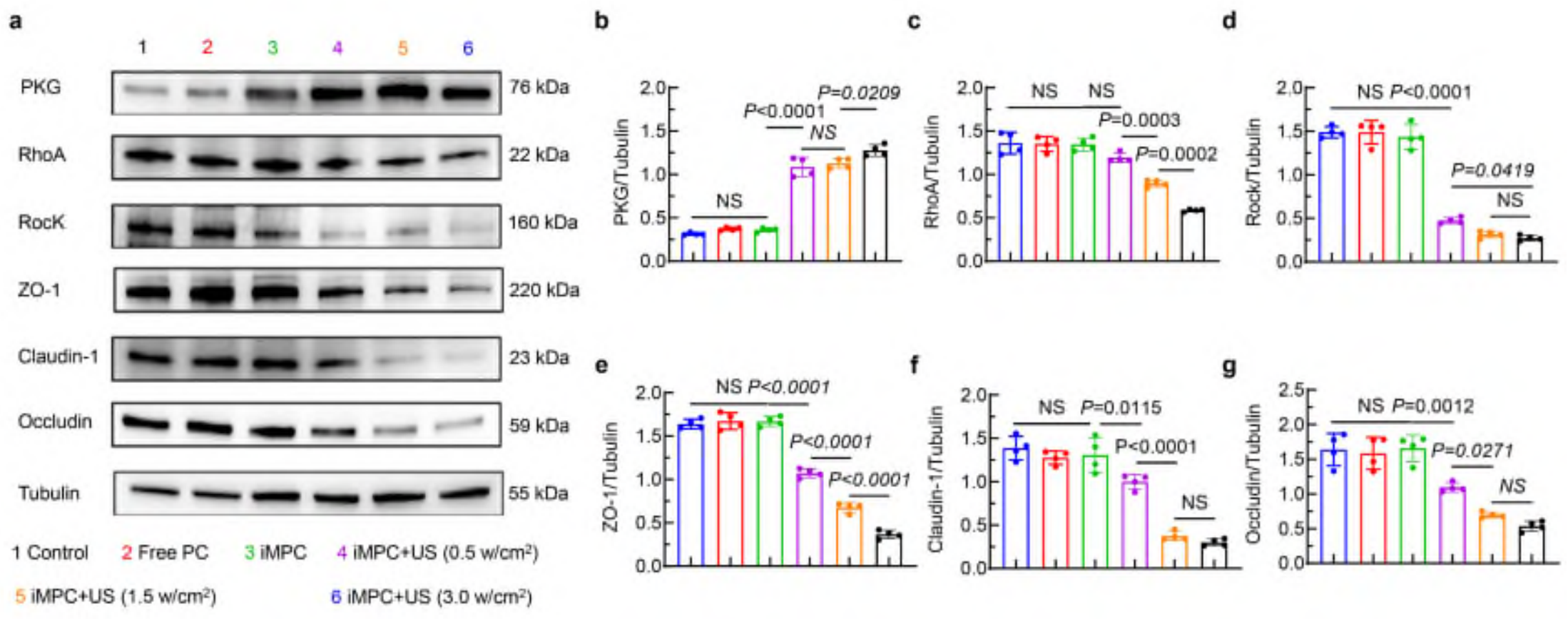

Supplementary Figure 9. Low-intensity pulsed ultrasound (LIPUS) disrupts tight junction proteins in the nasal mucosal barrier (NMB) model by activating the cGMP/PKG signaling pathway. a-g, The effects of different treatments on the cGMP/PKG signaling pathway and tight junction proteins. The gray value analysis was performed by using ImageJ software. n = 3 biologically independent mice per group for data in **b-g**. Data are expressed as the mean ± s.d. Statistical significances were determined using one-way ANOVA with Dunnett's post hoc test. Comparisons were performed between different groups in **b-g**. Not significant (NS) is $P \geq 0.05$, and significant P values are shown.

The description has been added (page 17 line 430-446) as: Research has shown a strong association between the cGMP/PKG signaling pathway and tight junction protein levels. WB analysis indicated a significant elevation in PKG protein levels in the US-treated group compared to the untreated group, with this increase showing a positive correlation with ultrasound intensity (Supplementary Fig. 9a-b). PKG protein levels in the iMPC+US (1.5 w/cm²) group were 3.59 times higher than those in the control group (Supplementary Fig. 9a-b). In the US-treated group, RhoA and Rock protein levels were significantly lower compared to the non-US-treated group, showing a negative correlation with US intensity (Supplementary Fig. 9c-d).

In the iMPC+US (1.5 w/cm²) group, RhoA and Rock protein levels were reduced by 1.52-fold and 4.72-fold, respectively, compared to the control group (Supplementary Fig. 9c-d). The US-treated group showed a significant decrease in tight junction proteins (ZO-1, Claudin-1, and Occludin) compared to the non-US-treated group, with these reductions negatively correlating with US intensity (Supplementary Fig. 9e-g). The iMPC+US (1.5 w/cm²) group showed a reduction in protein levels of ZO-1,

Claudin-1, and Occludin by 2.43-fold, 3.71-fold, and 2.35-fold, respectively, compared to the control group (Supplementary Fig. 9e-g). The findings indicate that US could interfere with NMB by triggering the cGMP/PKG signaling pathway.

The description has been added (page 58 line 1450-1457) as: The NMB model established using HNEpCs underwent six treatment conditions: control group (routine culture), Free PC group, iMPC group, iMPC+US (0.5 w/cm²) group, iMPC+US (1.5 w/cm²) group, and iMPC+US (3.0 w/cm²) group. The ultrasound parameters included a duty cycle of 30%, a center frequency of 1 MHz, and a duration of 60 seconds. Two hours later, Western blot analysis was conducted to detect the levels of PKG (AB199438), RhoA (AB187027), Rock (AB45171), ZO-1 (AB307799), Claudin-1 (AB211737), Occludin (AB216327), and GAPDH (AB181602), all sourced from Abcam, UK.

References

1. Wang, Z. H. et al. Bioactive nanomotor enabling efficient intestinal barrier penetration for colorectal cancer therapy. *Nat Commun* **16**, 1678 (2025).
2. Dong, L. et al. Open-source throttling of CD8+ T cells in the brain with low-intensity focused ultrasound-guided sequential delivery of CXCL10, IL-2, and aPD-L1 for glioblastoma immunotherapy. Advanced materials (Deerfield Beach, Fla.) 36, e2407235 (2024).
3. Liu, Y. et al. Customized intranasal hydrogel delivering methylene blue ameliorates cognitive dysfunction against Alzheimer's disease. Advanced materials (Deerfield Beach, Fla.) 36, e2307081 (2024).
4. Ruan, S. et al. Microneedle-mediated nose-to-brain drug delivery for improved Alzheimer's disease treatment. J Control Release 366, 712-731 (2024).
5. Ma, Z. et al. Controlled tough bioadhesion mediated by ultrasound. Science 377, 751-755 (2022).
6. Schoellhammer, C. M. et al. Applicability and safety of dual-frequency ultrasonic treatment for the transdermal delivery of drugs. J Control Release 202, 93-100 (2015).
7. Guo, J. et al. Bio-barrier-adaptable biomimetic nanomedicines combined with ultrasound for enhanced cancer therapy. Signal Transduct Target Ther 10, 137 (2025).
8. Yu, C. C. et al. A conformable ultrasound patch for cavitation-enhanced transdermal cosmeceutical delivery. Advanced materials (Deerfield Beach, Fla.) 35, e2300066 (2023).
9. Sonabend, A. M. et al. Repeated blood-brain barrier opening with an implantable ultrasound device for delivery of albumin-bound paclitaxel in patients with recurrent glioblastoma: a phase 1 trial. Lancet Oncol. 24, 509-522 (2023).

7. The results in Fig. 3a-d exhibit high iMPC accumulation in the tumor and trigeminal nerves for groups treated with 1.5 W/cm2 compared to groups treated with 3 W/cm2, which is conflicting. Elucidate on the same. Moreover, Fig. 4b-k, 4l, 4n, and 4m reveal

effective disruption of tight junction at an ultrasound power intensity of 3 W/cm2, but the same dose does not correlate with in vivo fluorescence imaging results (Fig. 3a-d). Elucidate on the contradictory phenomena. Also explain mechanotransduction (Ca^{2+} signaling) and Ca^{2+}-mediated RhoA/ROCK and myosin light chain kinase (MLCK) activation facilitated by ultrasound in epithelial cells, and cite appropriate scientific references to support your claims. Moreover, it is highly recommended to assess tight junction disruption by measuring trans-endothelial/epithelial electrical resistance (TEER).

Re: Thank you for your comments. Unlike the LIPUS at 1.5 w/cm² treatment that safely reverses open NMB, the LIPUS at 3.0 w/cm² applied to mouse nasal mucosa induces significant "thermal effects" and "inflammatory responses," leading to irreversible damage to the nasal mucosa. This limitation restricts the efficiency of nasal-to-brain delivery for iMPC.

Research indicates that the Piezo1 ion channel responds to mechanical stimulation by converting LIPUS signals into intracellular calcium ion currents. Enhanced calcium signaling stimulates the small GTPase RhoA and its downstream effector, Rho-associated protein kinase (ROCK). ROCK is essential for cellular functions including contraction, morphological alterations, and migration. Ca^{2+} activates specific calmodulins, thereby activating the small GTPase RhoA, which leads to ROCK activation. Ca^{2+} also enhances actin contractility by activating myosin light chain kinase (MLCK).

Additionally, we added experiments to assess tight junction disruption by measuring trans-endothelial/epithelial electrical resistance (TEER).

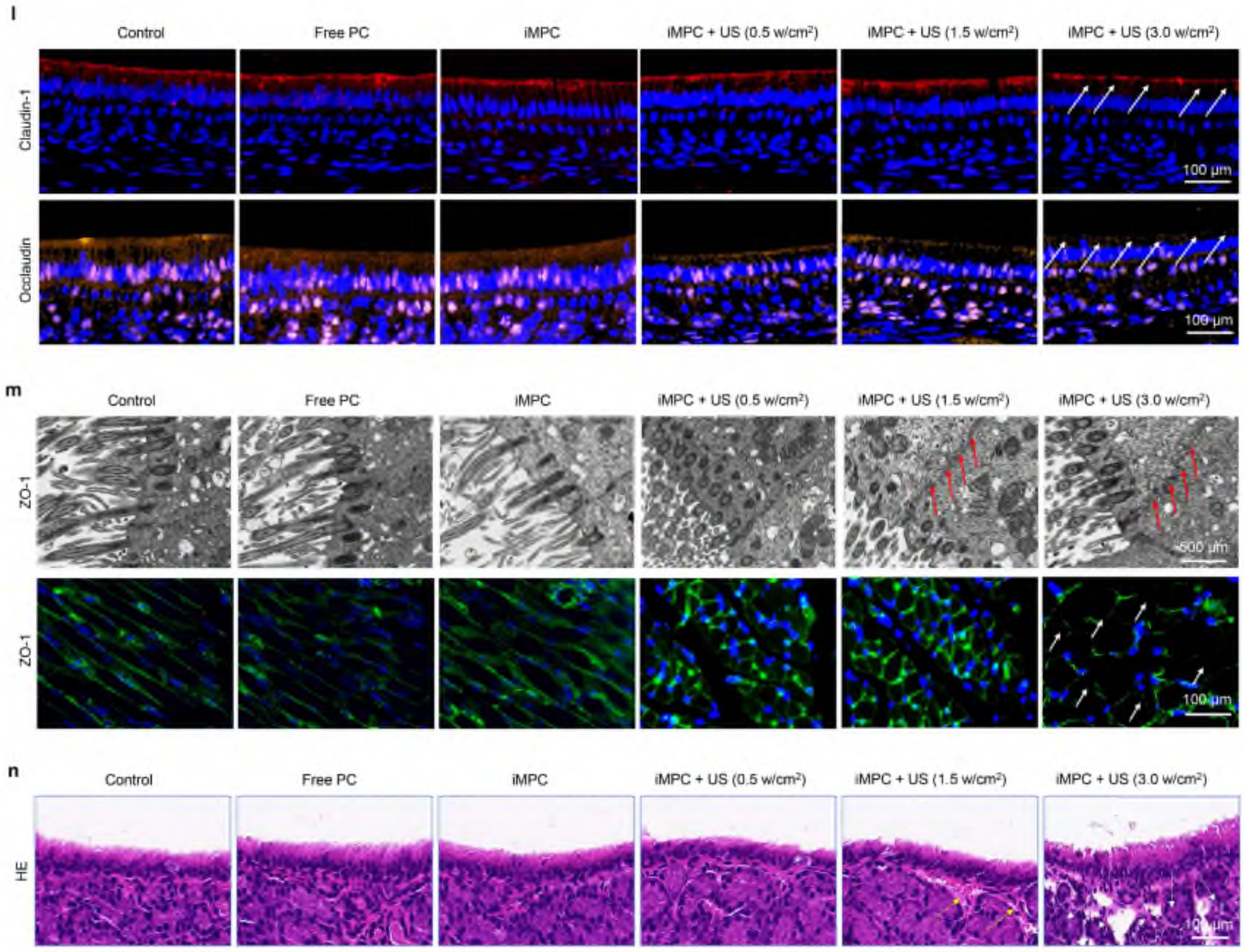

Figure 4l, Changes in tight junction proteins Occludin and Claudin-1 (blue: DAPI; red: CY3-Claudin-1; yellow: SpRed-Occludin). **m**, Alterations in tight junction proteins of the paracellular pathway (blue: DAPI; green: Alexa Fluor 488-ZO-1). **n**, Inflammatory cell infiltration after various treatments.

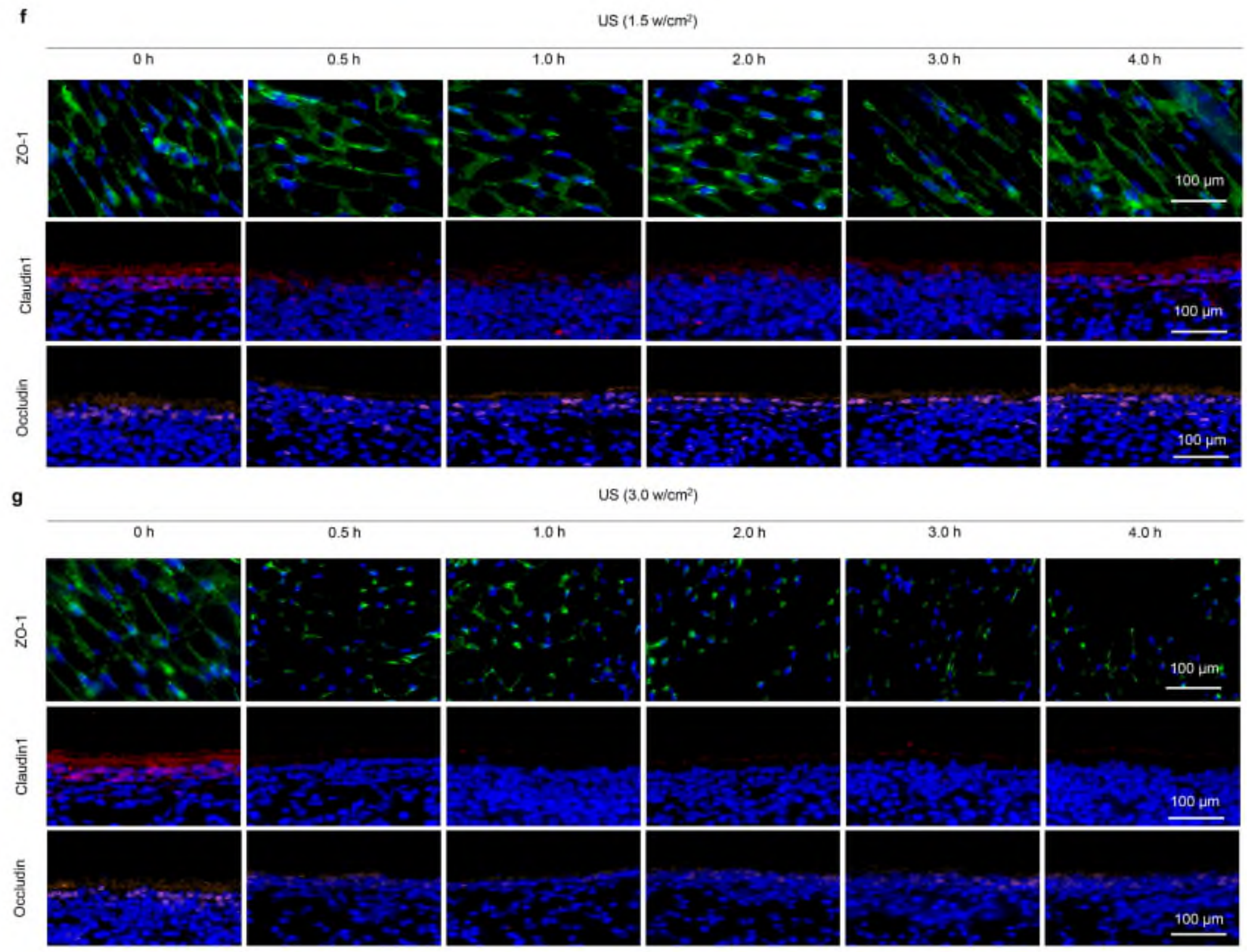

Figure 5f-g, Occludin, Claudin-1, and ZO-1 changes within 4 hours following LFUS exposure at 1.5 w/cm² or 3.0 w/cm² for 60 seconds (blue: DAPI; red: CY3-Claudin-1; yellow: SpRed-Occludin).

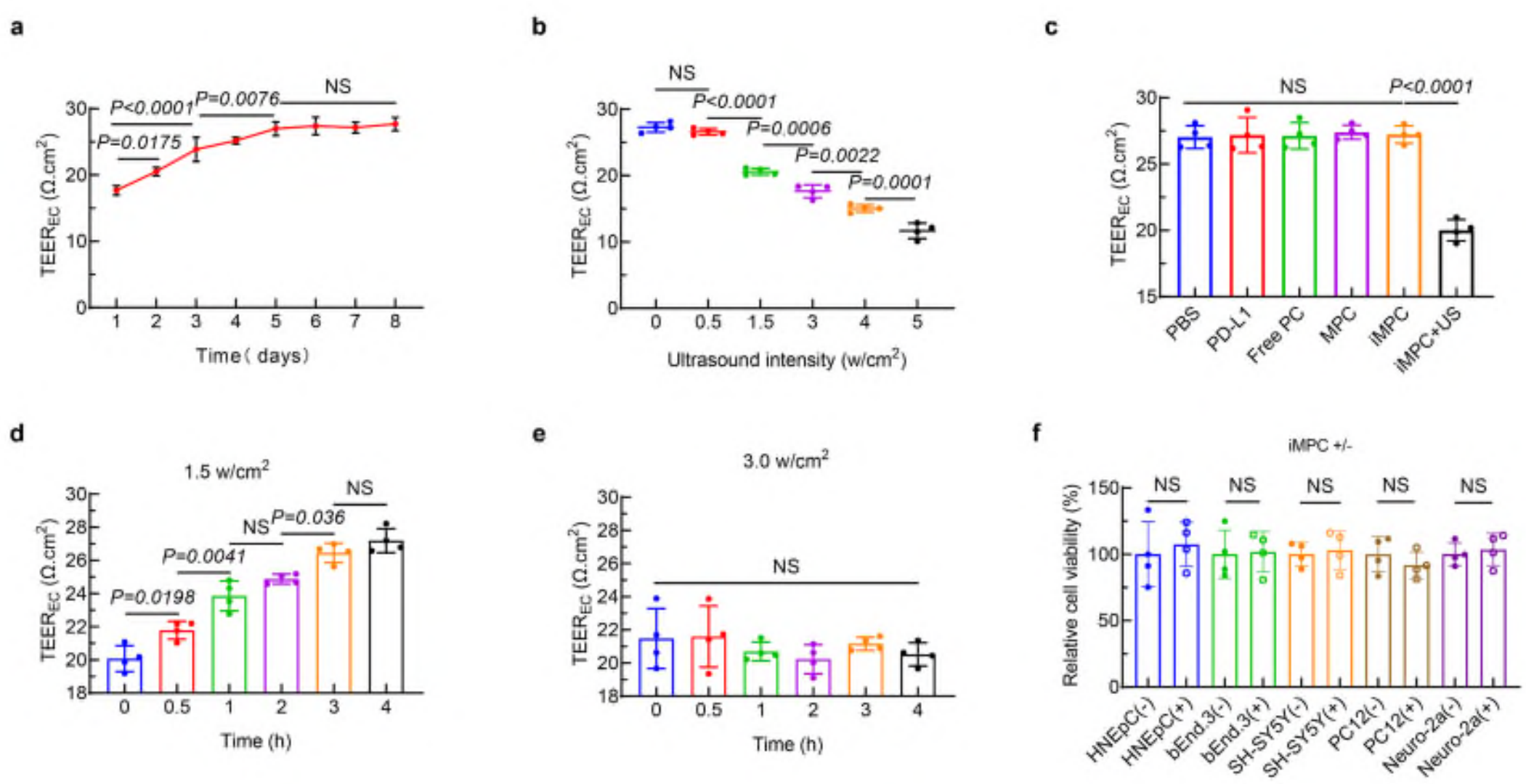

Supplementary Figure 8. Trans-epithelial electrical resistance (TEER) values in the nasal mucosal barrier (NMB) model. a, Changes in TEER values of the human nasal epithelial cell (HNEpC)-based NMB model (over 8 days). **b,** Changes in TEER values of the NMB model following treatment with different ultrasound intensities. **c,** Changes in TEER values of the NMB model after six different treatments. **d,** Ultrasonic intensity of 1.5 w/cm² was applied to NMB models, with changes in TEER values monitored over 4 hours. **e,** Ultrasonic intensity of 3.0 w/cm² was applied to NMB models, with changes in TEER values monitored over 4 hours. **f,** Cell viability

of HNEpC, bEnd.3, SH-SY5Y, PC12, and Neuro-2a cells after treatment with iMPC nanoformulation. n = 4 biologically independent mice per group for data in **a-f**. Data are expressed as the mean ± s.d. Statistical significances were determined using one-way ANOVA with Dunnett's post hoc test. Comparisons were performed between different groups in **a-f**. Not significant (NS) is $P \geq 0.05$, and significant P values are shown.

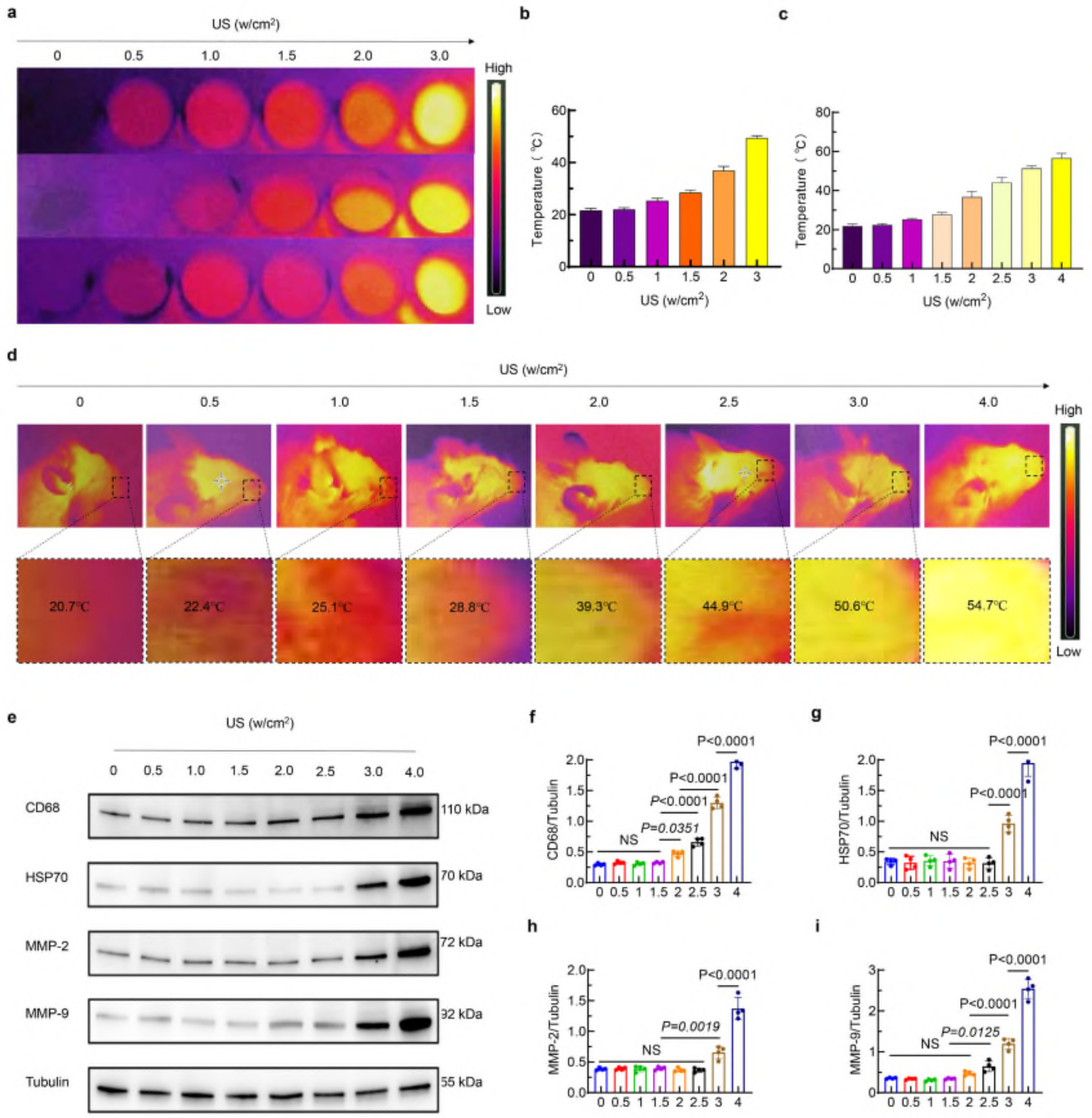

Supplementary Figure 17. Thermal injury of LIPUS on nasal mucosa. a-b, Temperature changes in the nasal mucosal barrier (NMB) model constructed from human nasal epithelial cells (HNEpC). **c-d,** Regional temperature changes in mouse nasal mucosa. **e-h,** Expression levels of HSP70, CD68, and MMP-9. Gray-scale analysis was performed using ImageJ software. n = 3 biologically independent mice per group for data in **a-c**. n = 4 biologically independent mice per group for data in **f-i**. Data are expressed as the mean ± s.d. Statistical significances were determined using one-way ANOVA with Dunnett's post hoc test. Comparisons were performed between different groups in **a-c** and **f-i**. Not significant (NS) is $P \geq 0.05$, and significant P values are shown.

The description has been added (page 28 line 675-679) as: Hematoxylin and eosin (H&E) staining of the nasal mucosa revealed significant inflammatory cell infiltration

when LIPUS at 3.0 w/cm² was combined with iMPC (Fig. 4n). LIPUS at an intensity of 3.0 w/cm² may excessively disrupt the NMB, heightening the risk of inflammatory cell infiltration. It highlights the primary reason for the greater efficiency of LIPUS in nose-to-brain delivery at 1.5 w/cm² rather than 3.0 w/cm².

The description has been added (page 28 line 711-718) as: We evaluated the impact of LIPUS at intensities of 1.5 w/cm² and 3.0 w/cm² on NMB-associated proteins to identify the optimal US intensity range for safely and reversibly opening the NMB. The study found that LIPUS at 1.5 w/cm² intensity disrupted ZO-1, Claudin1, and occludin, but these effects were reversible within 4 hours. (Fig. 5a-f). After LIPUS treatment at an intensity of 3.0 w/cm², the disruption of ZO-1, Claudin1, and occludin was not restored (Figure 5g). This may be due to the fact that LIPUS with too high ultrasound intensity caused significant structural disruption of the NMB and was unable to restore the normal structure.

The description has been added (page 16 line 395-404) as: The transendothelial electrical resistance (TEER) values in the NMB model stabilized by the fifth day, as shown in Supplementary Fig. 8a. An *in vivo* imaging system (*IVIS*) assessment demonstrated that the aPD-L1 content in the lower chamber progressively rose with increased US intensity (Fig. 2e-g). This conclusion was further validated by quantitative measurements using a microplate reader (Fig. 2e-g). TEER measurement results indicate that the TEER value of the NMB model gradually decreases with increasing US intensity (Supplementary Fig. 8b). The findings suggest a significant correlation between the extent of NMB disruption and ultrasonic energy, highlighting the need to identify a safe ultrasound intensity range to ensure NMB damage reversibility.

The description has been added (page 26 line 619-648) as: Sustained LIPUS exposure often induces varying degrees of “thermal effects.”[8,9,11] Thermal imaging results indicate that the temperature of the NMB model increases with rising US intensity (Supplementary Fig. 17a-b). The NMB model attained a temperature of 29.3°C after 60 seconds of continuous ultrasound exposure at an intensity of 1.5 w/cm² (Supplementary Fig. 17a-b). At an US intensity of 3.0 w/cm² with 60 seconds of continuous exposure, the NMB model reached a temperature of 50.1°C (Supplementary

Fig. 17a-b).

In vivo experiments demonstrated that the temperature in the mouse nasal mucosa region gradually increased with rising US intensity (Supplementary Fig. 17c-d). Specifically, when US intensity reached 3.0 w/cm² and was applied continuously for 60 seconds, the temperature in the mouse nasal mucosa region reached 50.6°C (Supplementary Fig. 17c-d). At an US intensity of 4.0 w/cm² with a 60-second exposure duration, the temperature in the mouse nasal mucosa region reached 54.7°C (Supplementary Fig. 17c-d). These data indicate that sustained exposure to US at certain intensities may cause thermal injury to nasal mucosal tissue.

WB analysis indicated that CD68 levels in the nasal mucosal tissue of mice exposed to 1.5 w/cm² US were comparable to the control group (0 w/cm²), whereas exposure to 3.0 w/cm² US resulted in a 4.41-fold increase in CD68 levels (Supplementary Fig. 17e-f). HSP70 levels in the nasal mucosa of mice exposed to 1.5 w/cm² ultrasound showed no significant change compared to the control group (0 w/cm²), whereas exposure to 3.0 w/cm² resulted in a 2.83-fold increase (Supplementary Fig. 17g).

The US group (1.5 w/cm²) exhibited no significant differences in matrix metalloproteinase-2 (MMP-2) and matrix metalloproteinase-9 (MMP-9) levels in nasal mucosal tissue compared to the control group (0 w/cm²) (Supplementary Fig. 17h-i). MMP-2 and MMP-9 levels in the nasal mucosal tissue of the US (3.0 w/cm²) group rose by 1.71-fold and 3.38-fold, respectively (Supplementary Fig. 17h-i). These data indicate that US treatment at an intensity of 3.0 w/cm² induces "thermal injury" and inflammatory infiltration in the nasal mucosal tissue region of mice. LIPUS at 1.5 w/cm² intensity did not significantly alter mouse nasal mucosal tissue structure or cause inflammatory infiltration.

References

1. Ruan, S. et al. Microneedle-mediated nose-to-brain drug delivery for improved Alzheimer's disease treatment. *J Control Release* **366**, 712-731 (2024).
2. Guo, J. et al. Bio-barrier-adaptable biomimetic nanomedicines combined with ultrasound for enhanced cancer therapy. *Signal Transduct Target Ther* **10**, 137 (2025).
3. Hoekstra, M. E. et al. Long-distance modulation of bystander tumor cells by $CD8^{+}$ T cell-secreted IFNγ. *Nat Cancer* **1**, 291-301 (2020).
4. Jia, W. et al. Low-intensity pulsed ultrasound responsive scaffold promotes intramembranous and

endochondral ossification via ultrasonic, thermal, and electrical stimulation. *ACS Nano* **19**, 4422-4439 (2025).
5. Venturini, V. et al. The nucleus measures shape changes for cellular proprioception to control dynamic cell behavior. *Science* **370**, (2020).
6. Franklin, J. M., Ghosh, R. P., Shi, Q., Reddick, M. P. & Liphardt, J. T. Concerted localization-resets precede YAP-dependent transcription. *Nat Commun* **11**, 4581 (2020).
7. Dong, Z. et al. TRPM-mediated mechanoreception regulates myosin oscillation during tissue elongation. *Curr. Biol.* **35**, 5793-5807.e4 (2025).

8. The authors emphasize low-frequency ultrasound throughout the manuscript. What is the typical range for low-frequency ultrasound? What factors contribute to the varying results observed at different frequency ranges?

Re: Thank you for your comment. The frequency of ultrasound determines its penetration depth; lower frequencies penetrate deeper. Currently, there is no consensus on the exact range for low-frequency ultrasound. Common classifications include: low-frequency ultrasound (20 kHz–1 MHz), mid-to-high-frequency ultrasound (1–20 MHz), and high-frequency/ultra-high-frequency ultrasound (20 MHz–1 GHz). To avoid ambiguity, we have corrected the term "low-frequency ultrasound (LFUS)" in the manuscript to "low-intensity pulsed ultrasound (LIPUS)."

The description has been added (page 2 line 36-38) as: Here, low-intensity pulsed ultrasound (LIPUS) without depending on the microbubbles to amplify energy, is directly used to reversibly open the NMB by disrupting tight junction proteins.

The description has been added (page 2 line 43-49) as: Consequently, compared to free aPD-L1, up to a 33.38-fold increase of aPD-L1 in the GBM region was obtained with LIPUS-mediated intranasal delivery of iMPC. Reactivating T cells significantly enhances immunotherapy, leading to a 40% tumor reduction, extended survival, and long-term immune memory in orthotopic GBM mice. Overall, the LIPUS-mediated NMB opening strategy notably enhances nose-to-brain drug delivery efficiency, offering a promising platform for treating brain diseases.

The description has been added (page 3 line 64-79) as: Low-intensity pulsed ultrasound (LIPUS), a non-invasive physical energy technique, has shown the ability to temporarily open biological barriers[7]. Ultrasonic mechanical effects are generated by producing an electrical signal first, which is then amplified and fed to the ultrasonic

horn[7-9]. Upon arriving at the horn, this electrical signal is transformed into mechanical waves by piezoelectric crystals via the transducer tip, and the waves are thereafter propagated to the target medium[8,9]. LIPUS can penetrate biological barriers due to its mechanical effects, including cavitation, viscous stress, and acoustic radiation[7-11]. For instance, drug permeation through the stratum corneum can be effectively enhanced under the acoustic energy of LIPUS[11]. Moreover, LIPUS has a promising therapeutic efficacy in clinical trials for brain diseases[12]. Microbubbles (MBs) amplify the acoustic energy of LIPUS to overcome 70-90% attenuation caused by the skull, enabling safe and reversible BBB opening[13]. Notably, NMB arises from the direct exposure of the nasal mucosa to the nasal cavity, devoid of physical skull barriers[14,15]. Currently, there are no studies documenting the direct use of LIPUS in NMB[7-9,15,16]. This study found that LIPUS can transiently loosen tight junctions of NMB via mechanical vibration, without requiring MBs-enhanced acoustic energy.

The description has been added (page 5 line 114-126) as: Based on these facts, a strategy using LIPUS was designed to safely and reversibly create a two-hours NMB opening window (Scheme 1). Notably, this approach employs LIPUS independently without MBs-mediated energy amplification, thereby avoiding vascular damage and other side effects associated with conventional LIPUS/MBs combination therapy for BBB disruption[13,34]. Subsequently, a pH-responsive bionic nanovesicle (iRGD-aPD-L1&carvedilol@EM, iMPC) system based on EM was developed to co-deliver carvedilol (a β-adrenergic receptor inhibitor that mitigates T-cell exhaustion) and aPD-L1, working synergistically with LIPUS-mediated NMB opening (Fig. 1a-d). Reactivating T cells significantly improved immunotherapy, achieving a 40% tumor reduction, extended survival, and long-term immune memory in orthotopic GBM mice. LIPUS efficiently and temporarily opens the NMB, significantly improving nose-to-brain drug delivery and providing a promising method for treating brain diseases.

9. How did you conclude to start the treatment on the 10th day after inoculation of the cancer cells in mice? On what basis was the treatment started? Justify with respect to tumor volume.

Re: Thank you for your comment. In the first few days after GBM cell inoculation, tumor volume may maintain a slower growth rate. However, as tumor cells proliferate, tumor volume typically enters an accelerated growth phase around day 10. The 10-day treatment window is designed to avoid initiating therapy too early, which could yield suboptimal results, while also preventing delayed treatment that might miss the optimal intervention window. In a previous study, we similarly administered treatment on day 10 after establishing the GBM mouse model (Advanced Materials 36, e2407235 (2024)).

10. How can immunotherapy alone completely eradicate tumors within 20 days (Fig. 5d and 5k) as GBM is considered to be one of the most robust and recurring ones? Most research articles published so far achieve such high efficiency in GBM therapy only through combination therapy involving chemotherapy and/or hyperthermia and immunotherapy.
Re: Thank you for your comments. In our previous studies, we found that enhancing T-cell activity in GBM regions and reversing the immunosuppressive tumor microenvironment, combined with aPD-L1, holds promise for achieving partial tumor clearance in GBM mouse models (Advanced Materials 35, e2209785 (2023); Advanced Materials 36, e2407235 (2024)). Of course, this may represent a short-term effect, and long-term observation is needed to determine whether tumors will recur.

11. Why is there a 2-fold and 4-fold increase in TNF-α and IFN-γ for the iMPC+US group upon comparison with the control (Fig. 7c and 7d)? Since iMPC by itself is not toxic to cancer cells, it delivers immunoadjuvants. State the role of CD8+ and CD4+ T-cells in the elevated levels of TNF-α and IFN-γ. Explain with appropriate scientific reasons and cite supporting references.
Re: Thank you for your insightful comments. $CD8^+$ T cells and $CD4^+$ T cells play crucial roles in immune responses by secreting TNF-α and IFN-γ. $CD8^+$ T cells primarily kill target cells directly and activate other immune cells through these cytokines, while $CD4^+$ T cells (particularly the Th1 subset) support $CD8^+$ T cell

function and enhance the activity of macrophages and other immune cells by secreting TNF-α and IFN-γ. Through mutual cooperation, both not only amplify immune responses but also play vital roles in immune processes such as antitumor and antiviral defense.

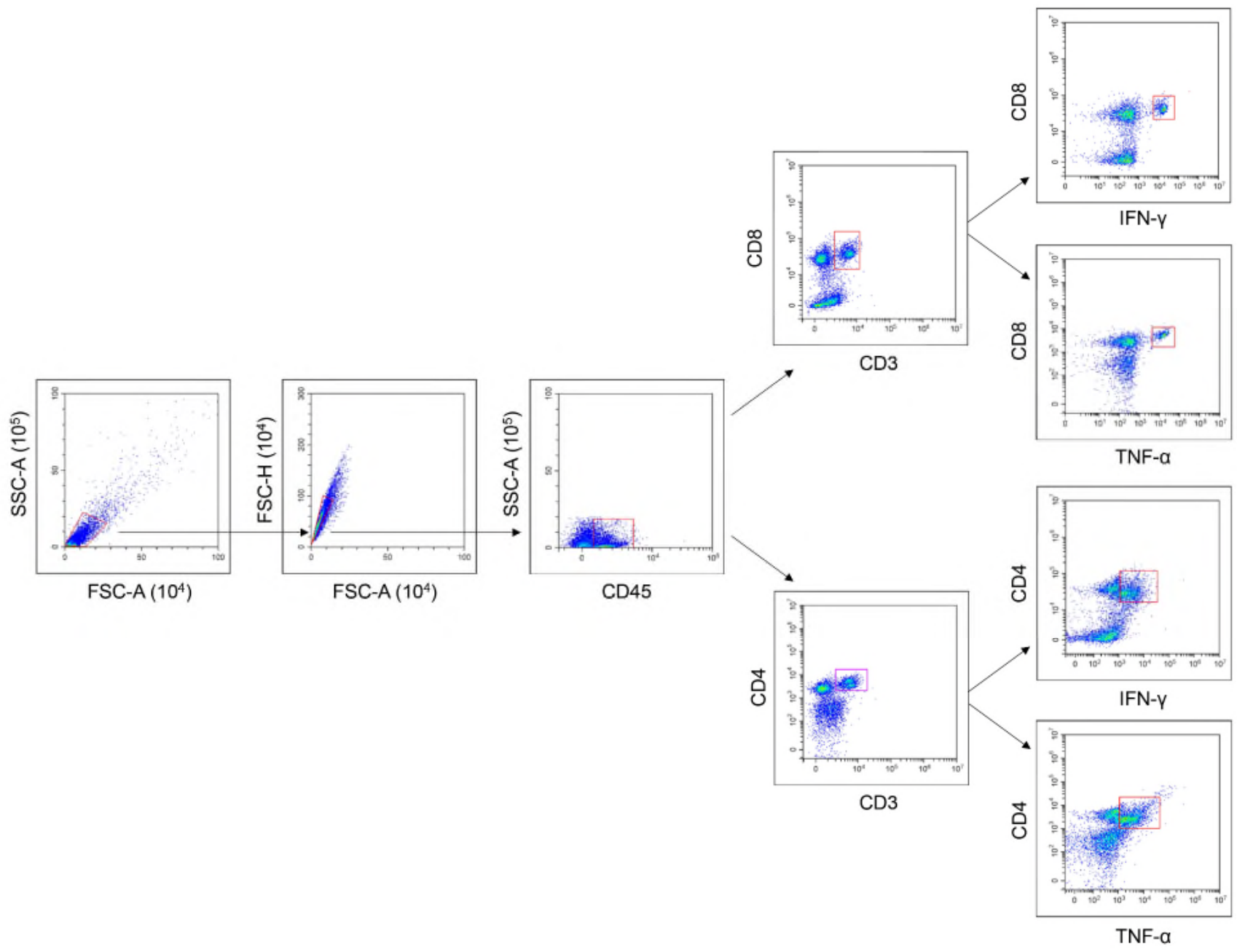

Supplementary Figure 10. Flow cytometry gating logic for $CD3^+CD8^+$ T cells and $CD3^+CD4^+$ T cells.

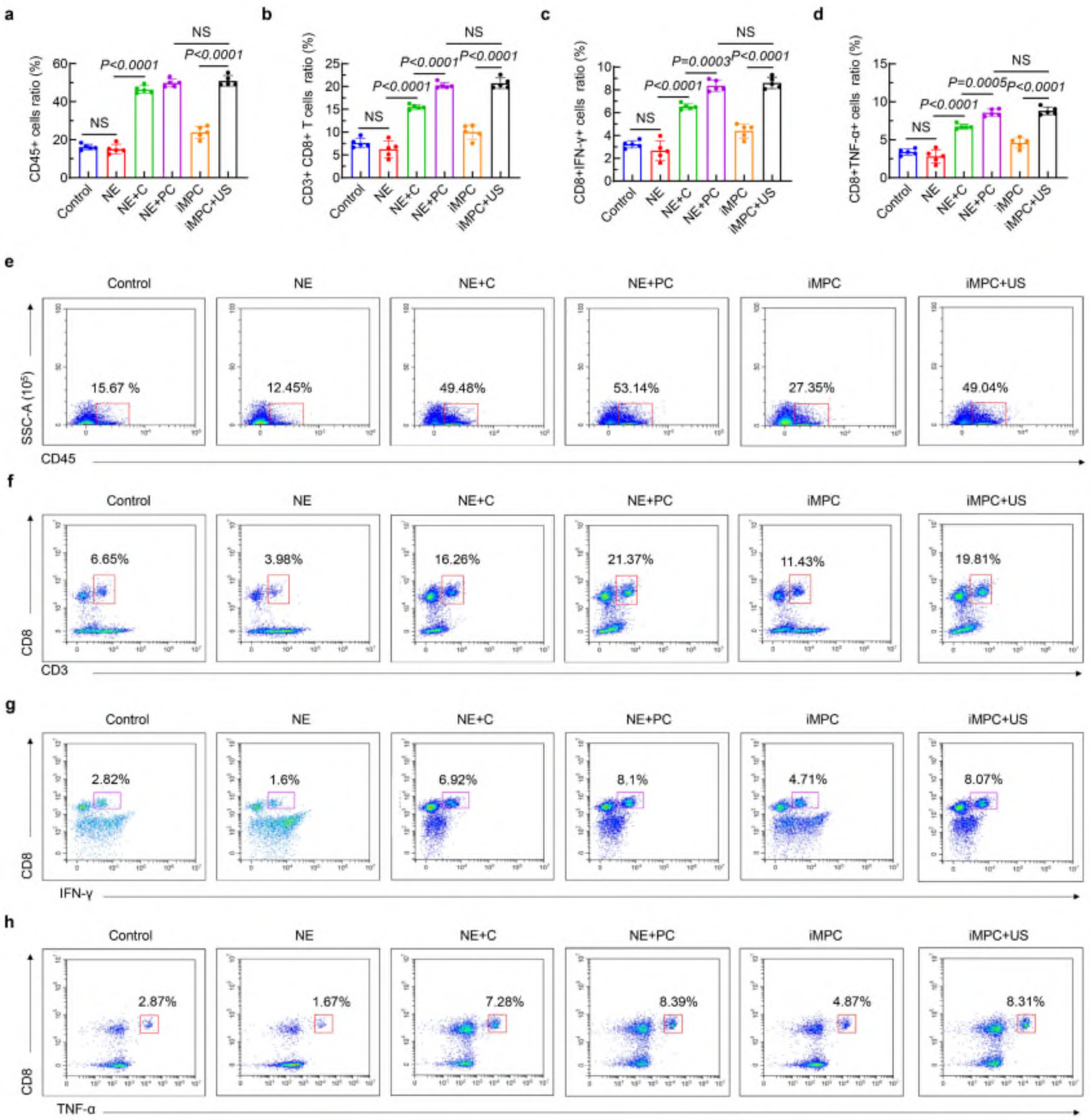

Supplementary Figure 11. β-adrenergic receptor blockade combined with aPD-L1 enhances the antitumor activity of $CD3^+CD8^+$ T cells. a, e, Proportion of $CD45^+$ lymphocytes after different treatments. **b, f,** Proportion of $CD3^+CD8^+$ T cells after different treatments. **c, g,** Proportion of IFN-γ^+CD8^+ T cells after different treatments. **d, h,** Proportion of TNF-α^+CD8^+ T cells after different treatments. n = 5 biologically independent mice per group for data in **a-d**. Data are expressed as the mean ± s.d. Statistical significances were determined using one-way ANOVA with Dunnett's post hoc test. Comparisons were performed between different groups in **a-d**. Not significant (NS) is $P \geq 0.05$, and significant P values are shown.

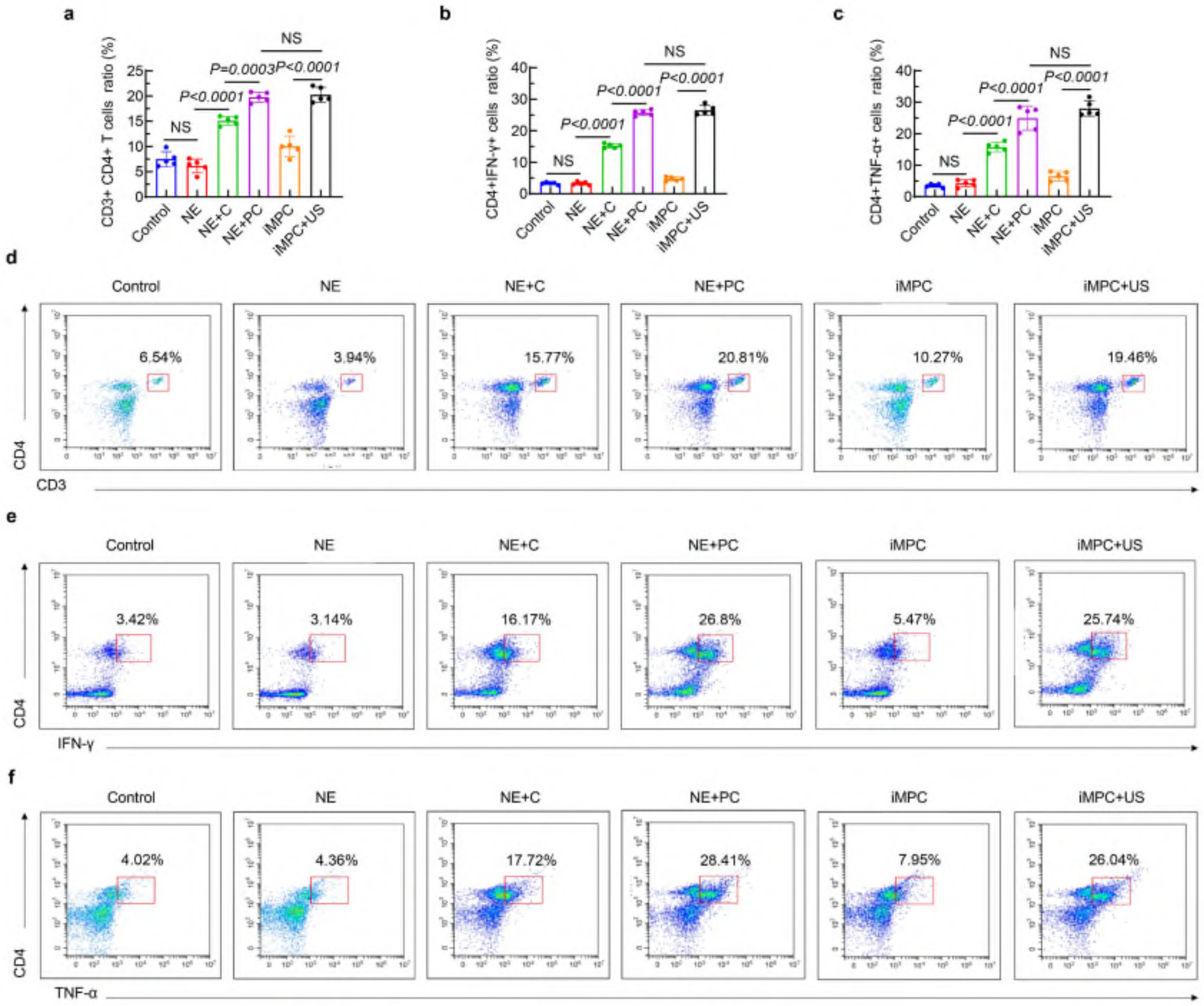

Supplementary Figure 12. β-adrenergic receptor blockade combined with aPD-L1 enhances CD3$^+$CD4$^+$ T cell activity. a, d, Proportion of CD3$^+$CD4$^+$ T cells after different treatments. **b, e,** Proportion of IFN-γ$^+$CD4$^+$ T cells after different treatments. **c, f,** Proportion of TNF-α$^+$CD4$^+$ T cells after different treatments. n = 5 biologically independent mice per group for data in **a-c**. Data are expressed as the mean ± s.d. Statistical significances were determined using one-way ANOVA with Dunnett's post hoc test. Comparisons were performed between different groups in **a-c**. Not significant (NS) is $P \geq 0.05$, and significant P values are shown.

The description has been added (page 18 line 451-490) as: CD4$^+$ T cells can secrete IFN-γ and TNF-α in tumor environments[42,43]. IFN-γ and TNF-α enhance antigen-presenting cell activation and promote the proliferation and activation of cytotoxic CD8$^+$ T cells[42,43]. We subsequently assessed the impact of combining β-blocker with aPD-L1 on the activity of CD4$^+$ and CD8$^+$ T cells using flow cytometry (Supplementary Fig. 10).

Results indicated a 3.97-fold increase in the proportion of CD45$^+$ lymphocytes in the lower chamber of the transwell in the NE+PC group compared to the NE group (Supplementary Fig. 11a and e). The iMPC+US group showed no significant change in the proportion of CD45$^+$ lymphocytes in the transwell's lower chamber compared to the

NE+PC group (Supplementary Fig. 11a and e). The iMPC+US group exhibited a 1.79-fold increase in the proportion of $CD45^+$ lymphocytes in the lower chamber of the transwell compared to the iMPC group (Supplementary Fig. 11a and e).

Detection of $CD3^+CD8^+$ T cell proportions in the lower chamber section revealed a 3.08-fold increase in the NE+PC group compared to the NE group (Supplementary Fig. 11b and f), a 4.33-fold rise in $IFN\gamma^+CD8^+$ T cell proportion (Supplementary Fig. 11c and g) demonstrate a 4.36-fold increase in the proportion of TNF-α^+CD8^+ T cells (Supplementary Fig. 11d and h). The iMPC+US group did not exhibit significant changes in the proportions of $CD3^+CD8^+$ T cells, $IFN\gamma^+CD8^+$ T cells, and TNF-α^+CD8^+ T cells in the lower chamber compared to the NE+PC group (Supplementary Fig. 11d and f-h). The iMPC+US group exhibited a 1.73-fold higher proportion of $CD3^+CD8^+$ T cells compared to the iMPC group (Supplementary Fig. 11b and f), a 1.71-fold rise in the percentage of $IFN\gamma^+CD8^+$ T cells (refer to Supplementary (Supplementary Fig. 11c and g), and 1.72-fold rise in the percentage of TNF-α^+CD8^+ T cells (Supplementary Fig. 11d and h).

Analysis of $CD3^+CD4^+$ T cell ratios in the lower chamber showed a 4.01-fold increase in the NE+PC group (Supplementary Fig. 12a and d), a 5.15-fold increase in $IFN\gamma^+CD4^+$ T cell ratio (Supplementary Fig. 12b and e), with a 4.06-fold increase in the proportion of TNF-α^+ $CD4^+$ T cells (Supplementary Fig. 12c and f), compared to the NE group. The iMPC+US group did not exhibit significant changes in the proportions of $CD3^+CD4^+$, IFN-γ^+CD4^+, and TNF-α^+CD4^+ T cells in the lower chamber compared to the NE+PC group (Supplementary Fig. 12a-f). The iMPC+US group showed a 1.89-fold higher proportion of $CD3^+CD4^+$ T cells compared to the iMPC group (Supplementary Fig. 12a and d), a 4.71-fold increase in the proportion of IFN-γ^+CD4^+ T cells (Supplementary Fig. 12a and d), a 3.28-fold higher proportion of TNF-α^+ $CD4^+$ T cells (Supplementary Fig. 12b and e, 12c and f).

These results indicate that combined β-blocker and aPD-L1 therapy enhances T cell antitumor activity. The proportion of $CD4^+$ and $CD8^+$ T cells with elevated TNF-α and IFN-γ secretion significantly increased. LIPUS-mediated NMB opening facilitates iMPC migration to the lower chamber, and release carvedilol and aPD-L1 to boost T

cell antitumor activity. This effect closely resembles the direct administration of β-blockers and aPD-L1 to the lower chamber.

The description has been added (page 60 line 1507-1523) as: Place the extracted lymphocytes in the lower chamber. The experiment comprised six groups: control group, NE group (NE placed in the lower chamber at 10 μM), NE+C (10 μM NE and 10 μM carvedilol placed in the lower chamber), NE+PC (10 μM NE, 10 μg/mL aPD-L1, and 10 μM carvedilol placed in the lower chamber), iMPC (35 μg/mL, placed in the upper chamber), and iMPC+US (35 μg/mL iMPC placed in the upper chamber, 1.5 w/cm^2). Other US parameters were as follows: duty cycle 30%, center frequency 1 MHz, and duration 60 s. After 24 hours, the cell suspension in the lower chamber was collected and cells were extracted.

Cells were stained for 90 minutes using antibodies against CD45, CD3, CD4, CD8a, TNF-α (catalog numbers: 103112, 100204, 100537, 100732, 506307; BioLegend, USA), and IFN-γ (catalog number: 505807; BioLegend, USA). The staining for anti-TNF-α and anti-IFN-γ was conducted following the BioLegend intracellular staining protocol (424401; BioLegend, USA). Flow cytometric analysis was performed on stained cells to identify populations of $CD3^+CD8^+$ and $CD3^+CD4^+$ T cells, including those expressing TNF-α and IFN-γ, with 20,000 events collected for analysis. Flow cytometric analysis antibodies were sourced from Dakewe Biotech Co., Ltd., while analytical grade chemicals were procured from Sigma-Aldrich (USA).

References

1. Hoekstra, M. E. et al. Long-distance modulation of bystander tumor cells by CD8+ T cell-secreted IFNγ. *Nat Cancer* **1**, 291-301 (2020).
2. Wherry, E. J. T cell exhaustion. *Nat. Immunol.* **12**, 492-499 (2011).
3. Crawford, A. et al. Molecular and transcriptional basis of $CD4^+$ T cell dysfunction during chronic infection. *Immunity* **40**, 289-302 (2014).
4. Globig, A. M. et al. The β1-adrenergic receptor links sympathetic nerves to T cell exhaustion. *Nature* **622**, 383-392 (2023).
5. Dong, L. et al. Open-Source Throttling of CD8+ T Cells in Brain with Low-Intensity Focused Ultrasound-Guided Sequential Delivery of CXCL10, IL-2, and aPD-L1 for Glioblastoma Immunotherapy. *Advanced materials (Deerfield Beach, Fla.)* **36**, e2407235 (2024).
6. Xu, X. et al. Recruiting T-Cells toward the Brain for Enhanced Glioblastoma Immunotherapeutic Efficacy by Co-Delivery of Cytokines and Immune Checkpoint

Antibodies with Macrophage-Membrane-Camouflaged Nanovesicles. *Advanced materials (Deerfield Beach, Fla.)* **35**, e2209785 (2023).

Minors:

1. For better reading flow, please add the abbreviation for the blood-brain barrier (BBB) in the abstract.

Re: Thank you for your comment. We have added the blood-brain barrier (BBB) in the abstract.

The description has been added (page 2 line 34-36) as: Intranasal delivery offers a direct route to the brain, circumventing the blood-brain barrier (BBB) and minimizing systemic toxicity. However, its efficiency is mainly limited by the nasal mucosal barrier (NMB).

2. In the abstract, you have written iMPC in brackets beside bionic nanovesicles without completely describing the final nanosystem. Include the term iMPC after clearly describing the proposed nanosystem for adequate comprehension of the reader.

Re: Thank you for your suggestions. We have revised it.

The description has been added (page 2 line 38-43) as: A bionic nanovesicle (iRGD-anti-programmed cell death ligand 1 (aPD-L1) & carvedilol (β-blocker) @ macrophage-derived extracellular vesicles, iMPC) was designed to co-deliver carvedilol for β-receptor blockade to reduce T-cell exhaustion, and aPD-L1 to enhance T-cell anti-tumor activity in orthotopic glioblastoma (GBM) mice during the two-hour window for NMB opening.

3. In the introduction section, you have stated “LFUS may transiently loosen tight junctions of NMB via mechanical vibration, without requiring MBs-enhanced acoustic energy”, making the use of LFUS-mediated biological barrier disruption seem like a possibility rather than an affirmed technique that has been exploited in your research. Moreover, the cited references (3 and 7) do not support the claim of ultrasound-mediated disruption of biological barriers. Reference ‘3’ doesn’t address ultrasound,

but reference ‘7’ supports the claim, stating that cavitation is facilitated by a chitosan (primer) solution upon exposure to ultrasound. Either change the statement or include appropriate references to support your claims regarding the disruption/loosening of NMB. Also elucidate in detail the underlying mechanism involved in ultrasound-induced loosening of NMB.

Re: Thank you for your suggestions. We have revised it.

The description has been added (page 3 line 64-79) as: Low-intensity pulsed ultrasound (LIPUS), a non-invasive physical energy technique, has shown the ability to temporarily open biological barriers[7]. Ultrasonic mechanical effects are generated by producing an electrical signal first, which is then amplified and fed to the ultrasonic horn[7-9]. Upon arriving at the horn, this electrical signal is transformed into mechanical waves by piezoelectric crystals via the transducer tip, and the waves are thereafter propagated to the target medium[8,9]. LIPUS can penetrate biological barriers due to its mechanical effects, including cavitation, viscous stress, and acoustic radiation[7-11]. For instance, drug permeation through the stratum corneum can be effectively enhanced under the acoustic energy of LIPUS[11]. Moreover, LIPUS has a promising therapeutic efficacy in clinical trials for brain diseases[12]. Microbubbles (MBs) amplify the acoustic energy of LIPUS to overcome 70-90% attenuation caused by the skull, enabling safe and reversible BBB opening[13]. Notably, NMB arises from the direct exposure of the nasal mucosa to the nasal cavity, devoid of physical skull barriers[14,15]. Currently, there are no studies documenting the direct use of LIPUS in NMB[7-9,15,16]. This study found that LIPUS can transiently loosen tight junctions of NMB via mechanical vibration, without requiring MBs-enhanced acoustic energy.

References

7. Ruan, S. et al. Microneedle-mediated nose-to-brain drug delivery for improved Alzheimer's disease treatment. *J Control Release* **366**, 712-731 (2024).
8. Ma, Z. et al. Controlled tough bioadhesion mediated by ultrasound. *Science* **377**, 751-755 (2022).
9. Schoellhammer, C. M. et al. Applicability and safety of dual-frequency ultrasonic treatment for the transdermal delivery of drugs. *J Control Release* **202**, 93-100 (2015).
10. Guo, J. et al. Bio-barrier-adaptable biomimetic nanomedicines combined with ultrasound for enhanced cancer therapy. *Signal Transduct Target Ther* **10**, 137 (2025).

11. Yu, C. C. et al. A conformable ultrasound patch for cavitation-enhanced transdermal cosmeceutical delivery. *Advanced materials (Deerfield Beach, Fla.)* **35**, e2300066 (2023).
12. Sonabend, A. M. et al. Repeated blood-brain barrier opening with an implantable ultrasound device for delivery of albumin-bound paclitaxel in patients with recurrent glioblastoma: a phase 1 trial. *Lancet Oncol.* **24**, 509-522 (2023).
13. Dong, L. et al. Open-source throttling of $CD8^+$ T cells in the brain with low-intensity focused ultrasound-guided sequential delivery of CXCL10, IL-2, and aPD-L1 for glioblastoma immunotherapy. *Advanced materials (Deerfield Beach, Fla.)* **36**, e2407235 (2024).
14. Chen, Y. et al. Intranasal drug delivery: The interaction between nanoparticles and the nose-to-brain pathway. *Adv. Drug Del. Rev.* **207**, 115196 (2024).
15. Wang, G. et al. Tailored borneol-modified lipid nanoparticles nasal spray for enhanced nose-to-brain delivery to central nervous system diseases. *ACS Nano* **18**, 23684-23701 (2024).
16. Wang, J. et al. Ultrasound-mediated blood-brain barrier opening: An effective drug delivery system for theranostics of brain diseases. *Adv. Drug Del. Rev.* **190**, 114539 (2022).

4. At line 66, please cite LFUS specifically for clinical trials related to brain diseases instead of referencing an entire review article.

Re: Thank you for your comment. We have revised it.

5. At line 90, "The membrane structure"

Re: Thank you for your comment. We have revised it.

6. At line 92, "studies have shown"

Re: Thank you for your comment. We have revised it.

7. The mechanism involved in pH-responsive iMPC wasn't stated to the fullest. Elaborate on the pH-responsive mechanism and clearly state which part of the EVs/Polyaspartic acid (PASP) molecule undergoes protonation under the acidic TME.

Re: Thank you for your comment. We have revised it.

The description has been added (page 4 line 104-110) as: Polyaspartic acid (PASP) is a typical pH-sensitive polymer that primarily responds to environmental pH changes through the ionization properties of its side chains[29,30]. Under acidic conditions (pH 5.5–6.5), PASP's negative charge rises due to the protonation of its side-chain carboxyl groups ($-COOH/-COO^-$). This causes polymer chain swelling, inducing structural

changes in the nanocarrier and releasing the loaded drug[31-33]. Conversely, at normal pH levels (such as pH 7.4, PASP maintains a relatively compact structure, inhibiting drug release[31].

References

29. Li, Q. et al. Poly(aspartic acid)-based pH-responsive targeting co-delivery nanoparticles. *J. Biomater. Appl.* **36**, 579-591 (2021).
30. Németh, C., Gyarmati, B., Abdullin, T., László, K. & Szilágyi, A. Poly(aspartic acid) with adjustable pH-dependent solubility. *Acta Biomater.* **49**, 486-494 (2017).
31. Gyenes, T., Torma, V., Gyarmati, B. & Zrínyi, M. Synthesis and swelling properties of novel pH-sensitive poly(aspartic acid) gels. *Acta Biomater.* **4**, 733-744 (2008).
32. Hakeem, A. et al. Polyaspartic acid-anchored mesoporous silica nanoparticles for pH-responsive doxorubicin release. *Int J Nanomedicine* **13**, 1029-1040 (2018).
33. Vega-Chacón, J., Arbeláez, M., Jorge, J. H., Marques, R. & Jafelicci, M. pH-responsive poly(aspartic acid) hydrogel-coated magnetite nanoparticles for biomedical applications. *Mater Sci Eng C Mater Biol Appl* **77**, 366-373 (2017).

8. Figure 1 is not intuitive for readers. It would be helpful to add more labels, such as for epithelial cells, endothelial cells, cilia, and iMPC. The fence structure associated with the epithelial cells may be confusing; providing a similar schematic for Scheme 1, Fig. 1c, and Fig. 4a would be beneficial for readers.

Re: Thank you for your comment. We have revised it.

[Figure Redacted]

Scheme 1. Low-frequency ultrasound enhances nose-to-brain drug delivery by opening the nasal mucosal barrier.

9. Include a scale bar for the insert image in Fig. 1e (TEM of EVs). Additionally, the fig reveals the EVs to be sized at nearly 30 nm, whereas after loading with carvedilol and aPD-L1, there is a drastic increase in the final nanoparticle (iMPC), nearly 100 nm. A minor increase of 10-15nm in size is usually observed. But in your case, the difference is enormous, and the reasons for the same weren't explained. Give a scientific explanation of the same and clearly elucidate the influence posed by PASP, if any.

Re: Thank you for your comment. We have revised it.

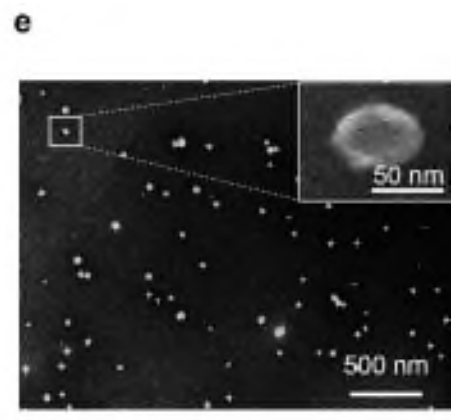

Figure 1e, Transmission electron microscopy image of extracellular vesicles from the macrophage cell line RAW 264.7.

The particle size of iMPC nanoparticles increased significantly from 50 nm in M-EVs to 100 nm due to a hierarchical nanostructure formed by multi-step modification and loading[29,31,40]. Ultrasonic permeabilization converts M-EVs into hollow M-EVMs. The conjugated PASP molecular chains unfurl in aqueous environments, increasing steric hindrance and hydrodynamic diameter[29-32]. This transformation alters the vesicles from flexible native membranes to rigid polymer-membrane composites, raising the baseline particle size[31-33]. When aPD-L1 and carvedilol are ultrasonically loaded, they occupy the hollow lumen and bind to the inner membrane or PASP chains through

hydrogen bonds and hydrophobic interactions, supporting the vesicle structure and further expanding particle size. Additionally, the iRGD peptide chains create a steric hydration layer in solution, significantly enhancing the nanoparticle's hydrodynamic radius[27,28].

References

27. Pan, Y. et al. Glycoengineering-based anti-PD-1-iRGD peptide conjugate boosts antitumor efficacy through T cell engagement. *Cell Reports Medicine* **5**, 101590 (2024).
28. Ding, N. et al. iRGD synergizes with PD-1 knockout immunotherapy by enhancing lymphocyte infiltration in gastric cancer. *Nat Commun* **10**, 1336 (2019).
29. Li, Q. et al. Poly(aspartic acid)-based pH-responsive targeting co-delivery nanoparticles. *J. Biomater. Appl.* **36**, 579-591 (2021).
30. Németh, C., Gyarmati, B., Abdullin, T., László, K. & Szilágyi, A. Poly(aspartic acid) with adjustable pH-dependent solubility. *Acta Biomater.* **49**, 486-494 (2017).
31. Gyenes, T., Torma, V., Gyarmati, B. & Zrínyi, M. Synthesis and swelling properties of novel pH-sensitive poly(aspartic acid) gels. *Acta Biomater.* **4**, 733-744 (2008).
32. Hakeem, A. et al. Polyaspartic acid-anchored mesoporous silica nanoparticles for pH-responsive doxorubicin release. *Int J Nanomedicine* **13**, 1029-1040 (2018).
33. Vega-Chacón, J., Arbeláez, M., Jorge, J. H., Marques, R. & Jafelicci, M. pH-responsive poly(aspartic acid) hydrogel-coated magnetite nanoparticles for biomedical applications. *Mater Sci Eng C Mater Biol Appl* **77**, 366-373 (2017).
40. Dong, L. et al. Recruiting T-cells toward the brain for enhanced glioblastoma chemo-immunotherapy efficacy by co-delivery of cytokines and temozolomide via ultrasound-gated redox-responsive extracellular vesicles. *J Nanobiotechnology* **23**, 766 (2025).

10. A rapid release of aPD-L1 (more than 50%) could be observed within the first 8 Hrs under acidic pH (Fig. 1l). In the field of drug delivery, nanosystems are designed and intended for a prolonged and sustained release. The proposed therapeutic system iMPC defeats the whole purpose as most of the drug would be cleared via the renal system and the first pass effect posed by the hepatic system. Defend the design of iMPC over rapid clearance and therapeutic efficiency.

Re: Thank you for your comments. In the field of drug delivery, when administered intravenously, nanosystems are designed for prolonged and sustained release, avoiding clearance by the first-pass effect generated by the renal and hepatic systems—a significant advantage of nanomedicines.

However, the primary focus of this study is to enhance the nasal-to-brain delivery efficiency of iMPCs by safely and reversibly opening the nasal mucosal barrier (NMB)

via LIPUS, thereby achieving the goal of boosting local anti-tumor immune responses at the GBM. The key advantage lies in nasal-to-brain delivery.

The description has been added (page 10 line 256-264) as: The efficiency of aPD-L1 release from iMPC was further compared at pH 7.4, 6.5, and 6.0. The results demonstrated that, compared to pH 7.4, the amount of aPD-L1 released from iMPC increased by 2.37-fold at pH 6.5 and by 2.66-fold at pH 6.0 (Fig. 1l). However, under acidic pH conditions, aPD-L1 was rapidly released (over 50%) within the 8 h (Fig. 1l). In the field of drug delivery, nanosystems are engineered for prolonged and sustained release[14,39]. Consequently, the iMPC drug-loaded nanosystem can be further designed and optimized to extend release duration, circumvent first-pass effects in the renal and hepatic systems, and thereby accommodate diverse administration routes[32,33].

References

1. Chen, Y. et al. Intranasal drug delivery: The interaction between nanoparticles and the nose-to-brain pathway. *Adv. Drug Del. Rev.* **207**, 115196 (2024).
2. Hakeem, A. et al. Polyaspartic acid-anchored mesoporous silica nanoparticles for pH-responsive doxorubicin release. *Int J Nanomedicine* **13**, 1029-1040 (2018).
3. Vega-Chacón, J., Arbeláez, M., Jorge, J. H., Marques, R. & Jafelicci, M. pH-responsive poly(aspartic acid) hydrogel-coated magnetite nanoparticles for biomedical applications. *Mater Sci Eng C Mater Biol Appl* **77**, 366-373 (2017).
4. Pedder, J. H. et al. Crossing the blood-brain barrier: emerging therapeutic strategies for neurological disease. *Lancet Neurol* **24**, 246-260 (2025).

11. The surface charge of the proposed nanovesicle system (iMPC) is positive, and the cell membrane of all cells is negatively charged. While cationic nanosystems prove to be effective for nose-to-brain delivery by enhancing retention in nasal mucus and the olfactory region, they can also strongly adhere/bind to the anterior respiratory epithelium, where most of the initial dose is deposited. Adsorption onto the anterior respiratory epithelium would increase the risk of off-targeting and accumulation in other tissues, with clearance by the renal and hepatic systems. Justify the concern about off-targeting by positively charged iMPC, which can reduce therapeutic efficiency with a possible risk of systemic toxicity by complement activation.

Re: Thank you for your comments. We have added the experiments, and the results indicate that following intranasal administration of the iMPC suspension, a portion of

the iMPCs enter the circulation. However, comprehensive assessments—including blood biochemistry, complete blood count, pharmacokinetics, clearance rates in major organs (liver, kidney, and lung), and organ distribution—demonstrate that LIPUS-mediated iMPC nasal-to-brain delivery represents a safe therapeutic strategy for GBM.

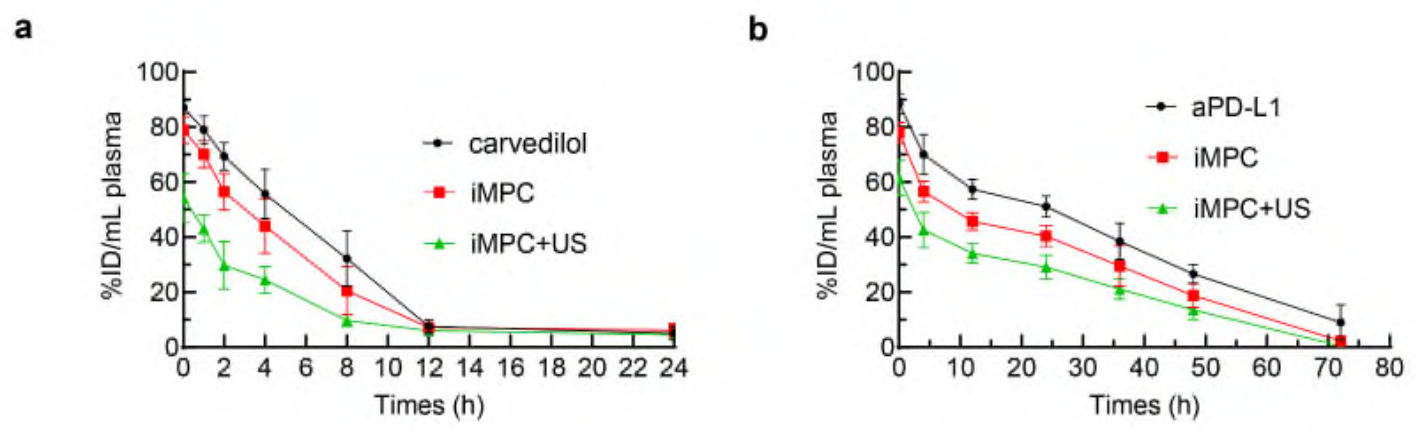

Supplementary Figure 14. Pharmacokinetic monitoring of carvedilol, aPD-L1, and iMPC nano-formulations. a. Percentage change in blood concentration of carvedilol. **b.** Percentage change in blood concentration of aPD-L1. n = 3 biologically independent mice per group for data in **a-b**. Data are expressed as the mean ± s.d.

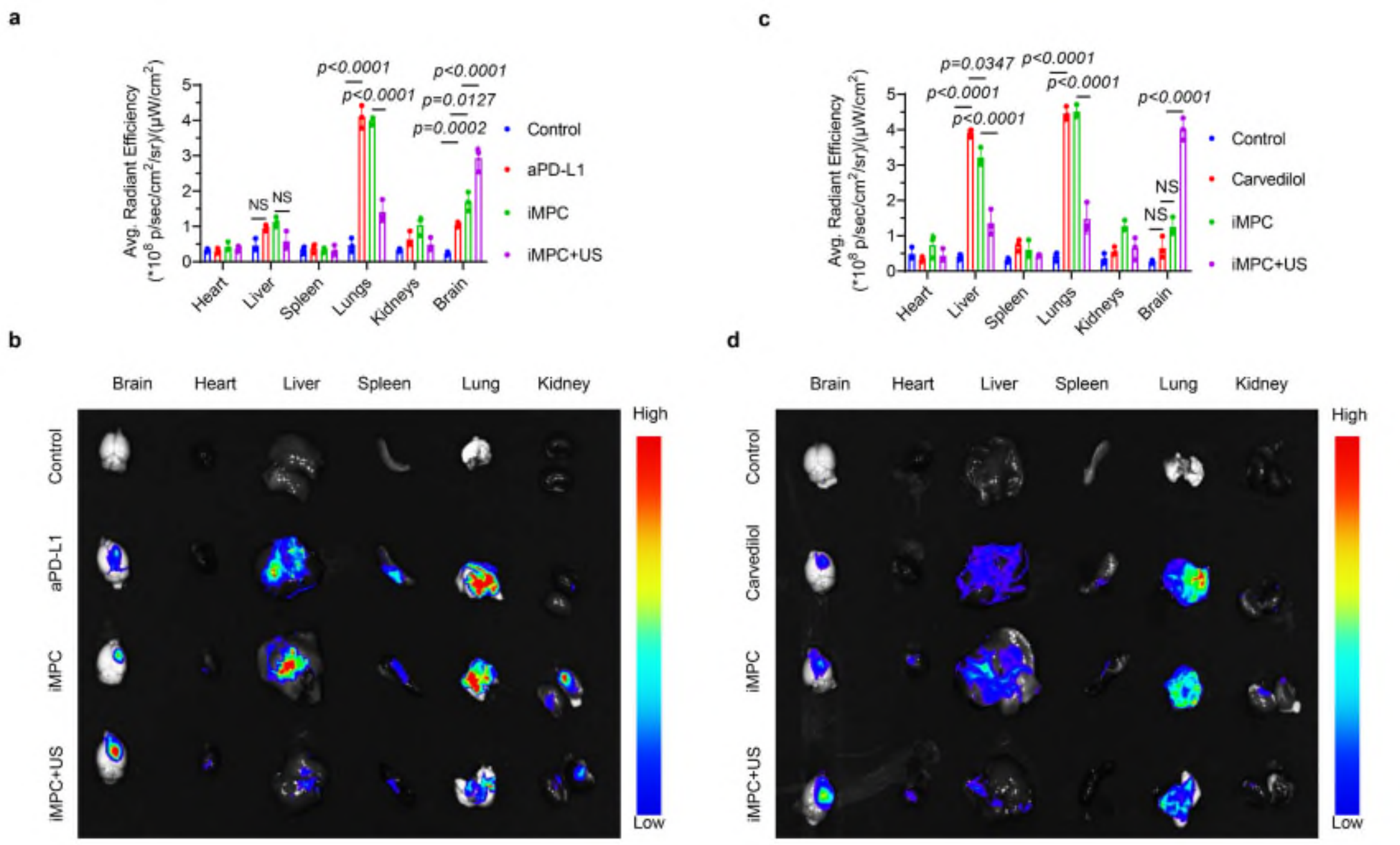

Supplementary Figure 15. Organ distribution of carvedilol, aPD-L1, and iMPC nano-formulations. a-b, Distribution of aPD-L1 and iMPC nano-formulations in heart, liver, spleen, lung, and kidney. **c-d,** Distribution of carvedilol and iMPC nano-formulations in heart, liver, spleen, lung, and kidney. n = 3 biologically independent mice per group for data in **a** and **c**. Data are expressed as the mean ± s.d. Statistical significances were determined using one-way ANOVA with Dunnett's post hoc test. Comparisons were performed between different groups in **a** and **c**. Not significant (NS) is $P \geq 0.05$, and significant P values are shown.

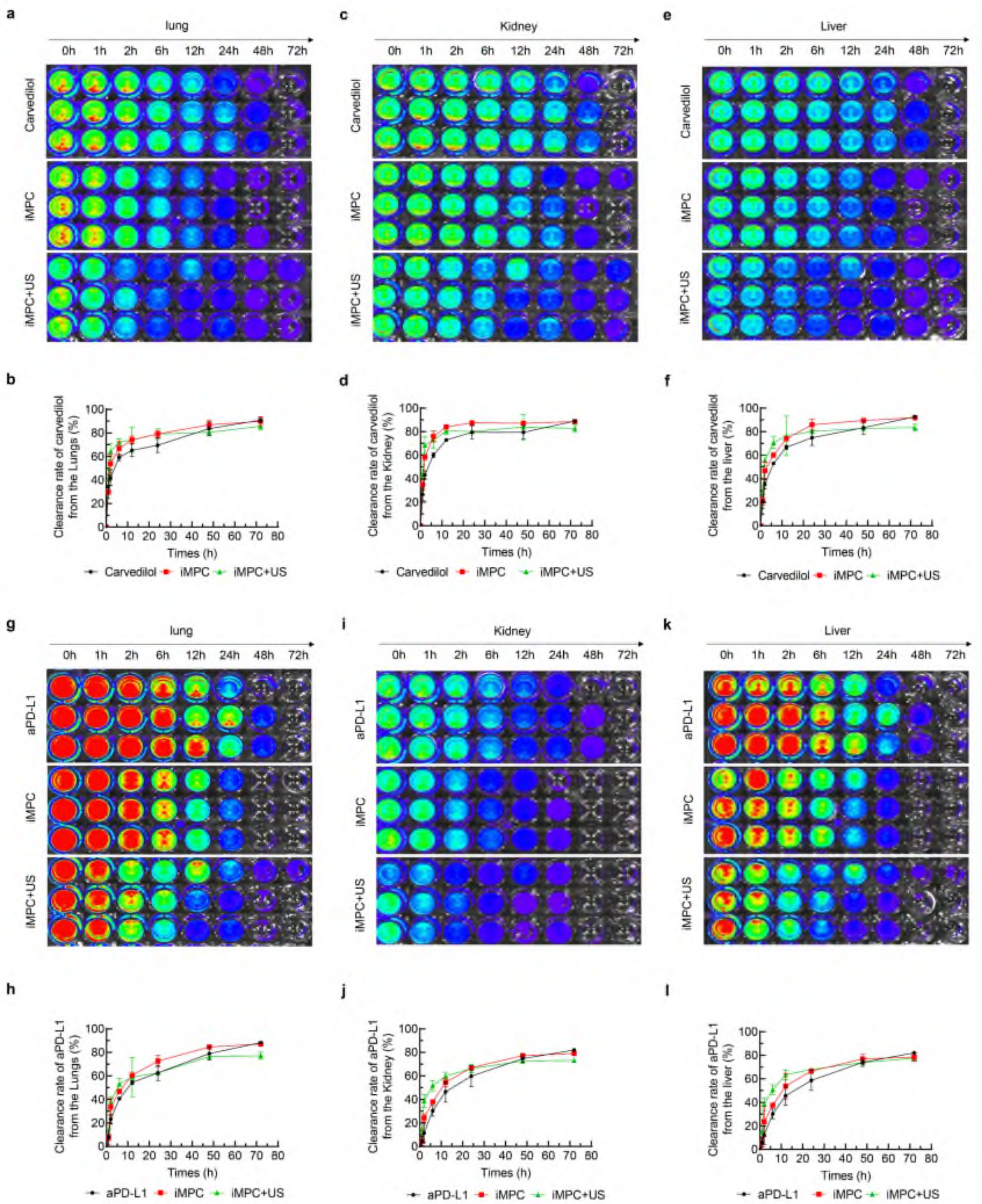

Supplementary Figure 16. Cardiac clearance of carvedilol, aPD-L1, and the iMPC nano-formulations. a-b, Pulmonary clearance of carvedilol and the iMPC nano-formulations. **c-d,** Renal clearance of carvedilol and the iMPC nano-formulations. **e-f,** Hepatic clearance of carvedilol and the iMPC nano-formulations. **g-h,** Pulmonary clearance of aPD-L1 and the iMPC nano-formulations. **i-j,** Renal clearance of aPD-L1 and the iMPC nano-formulations. **k-l,** Hepatic clearance of aPD-L1 and the iMPC nano-formulations. n = 3 biologically independent mice per group for data in **a-l**. n = 5 biologically independent mice per group for data in **a-l**. Data are expressed as the mean ± s.d.

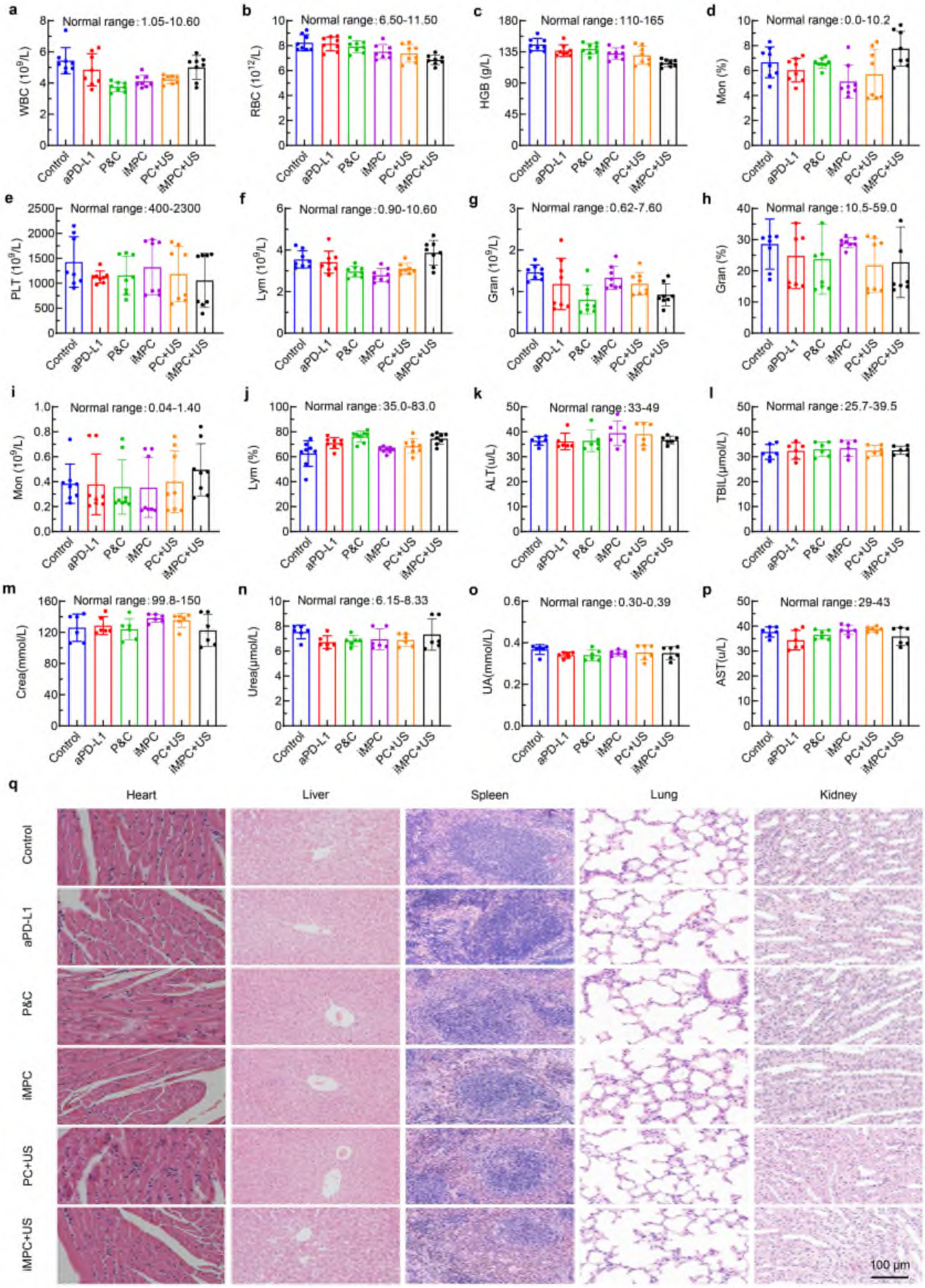

Supplementary Figure 33. Safety evaluation. a-j, Evaluation of blood counts. **k-p**, Blood biochemistry evaluation. **q**, HE staining of heart, liver, spleen, lung, and kidney tissues. n = 6 biologically independent mice per group for data in **a-p**. Data are expressed as the mean ± s.d. Statistical significances were determined using one-way ANOVA with Dunnett's post hoc test. Comparisons were performed between different groups in **a-p**. Not significant (NS) is $P \geq 0.05$, and significant P values are shown.

The description has been added (page 25 line 597-618) as: In contrast, compared

with the free carvedilol or iMPC group, the amount of carvedilol entering the bloodstream was significantly reduced in the iMPC+US group (Supplementary Fig. 14a). The *in vivo* half-life of carvedilol was 6 h (Supplementary Fig. 14a). Compared with the free aPD-L1 or iMPC groups, the amount of aPD-L1 entering the bloodstream was significantly reduced in the iMPC+US group (Supplementary Fig. 14b). The *in vivo* half-life of aPD-L1 was 36 h (Supplementary Fig. 14b). These data indicate that carvedilol and aPD-L1 administered intranasally enter the circulation, with aPD-L1 persisting longer than carvedilol.

The distribution of carvedilol and aPD-L1 across the heart, liver, spleen, lungs, kidneys, and brain in various treatment groups showed that the iMPC+US group had significantly lower aPD-L1 levels in the liver and lungs compared to free aPD-L1 or iMPC alone, while the distribution in brain tumors was notably higher (Supplementary Fig. 15a-b). Compared with free carvedilol or iMPC, the iMPC+US group showed significantly reduced carvedilol distribution in the liver and lungs, while brain tumor distribution was significantly increased (Supplementary Fig. 15c-d).

Clearance rate measurements indicated that carvedilol was rapidly cleared from liver, kidney, and lung tissues within 6 hours, while aPD-L1 exhibited a significantly delayed clearance, extending beyond 48 hours (Supplementary Fig. 16a-l). These data indicate that US enhances iMPC brain penetration efficiency by opening the NMB, thereby delivering carvedilol and aPD-L1 to tumor regions. Compared to free carvedilol or the nanoparticle formulation, the clearance rate of free aPD-L1 or the nanoparticle formulation is slower.

The description has been added (page 61 line 1546-1954) as: **Pharmacokinetics of iMPC *in vivo*** The pharmacokinetic behavior of carvedilol, aPD-L1, and various nano-formulations was studied by administering multiple nano-formulations intranasally to healthy C57 mice (n = 3). The administered dosages were 30 μg of aPD-L1 and 4 μg of carvedilol per 20 g of body weight. Ultrasound parameters included a 1.5 w/cm² intensity, 30% duty cycle, 1 MHz center frequency, and a 60-second duration. Blood samples were collected at 0, 1, 2, 4, 8, 12, 24, 36, 48, and 72 hours following intranasal

administration. Serum was extracted and subjected to acid treatment. Carvedilol and aPD-L1 concentrations were measured using a Tecan Safire fluorescence microplate reader (Lyon, France).

Intra-body distribution and clearance rates in major organs of iMPC

To investigate the in vivo distribution of aPD-L1, 6-8-week-old C57 mice (20-22 g) were selected for the experiment, with males and females randomly assigned. The experiment involved nasal administration and was divided into four groups: control, aPD-L1, iMPC, and iMPC+US. LIPUS targeted the olfactory epithelium within the nasal cavity. aPD-L1 was labeled with Cy5-NHS (Starleaf Biological Technology Co, Nanjing, China). The aPD-L1 dosage was administered at 30 μg per 20 g of body weight. IVIS imaging, conducted six hours later, identified fluorescent signals in the heart, liver, spleen, lungs, kidneys, and brain across all four groups. Fluorescence signal values were statistically analyzed using ImageJ software (ImageJ Software Inc., USA).

To investigate the in vivo distribution of aPD-L1, 6-8-week-old C57 mice (20-22 g) were selected for the experiment, with males and females randomly assigned. The experiment involved nasal administration and was divided into four groups: control, aPD-L1, iMPC, and iMPC+US. LIPUS targeted the olfactory epithelium within the nasal cavity. aPD-L1 was labeled with Cy5-NHS (Starleaf Biological Technology Co, Nanjing, China). The aPD-L1 dosage was administered at 30 μg per 20 g of body weight. Six hours later, IVIS imaging revealed fluorescent signals in the heart, liver, spleen, lungs, kidneys, and brain across all four groups. Fluorescence signal values were statistically analyzed using ImageJ software (ImageJ Software Inc., USA).

The in vivo distribution of carvedilol was examined by categorizing the experiment into four groups: control, carvedilol, iMPC, and iMPC+US. Carvedilol was labeled with Cy5-NHS (Starleaf Biological Technology Co., Nanjing, China). Carvedilol was administered at a dosage of 4 μg per 20 g of body weight. Six hours after administration, IVIS imaging revealed fluorescence in the heart, liver, spleen, lungs, kidneys, and brain of mice in all four groups. Fluorescence signal values were statistically analyzed using ImageJ software (ImageJ Software Inc., USA).

Further evaluate the clearance rates of carvedilol and aPD-L1 in the lungs, kidneys, and liver. Select 6-8-week-old C57 mice (20-22 g) for the experiment, randomly assigning males and females, and administer the drugs intranasally. LIPUS was administered to the olfactory epithelium in the mouse nasal cavity. Experimental groups included: carvedilol group, aPD-L1 group, iMPC group, and iMPC+US group. aPD-L1 was administered at 30 μg per 20 g of body weight, while carvedilol was given at 4 μg per 20 g of body weight. Carvedilol and aPD-L1 were conjugated with Cy5-NHS (Starleaf Biological Technology Co, Nanjing, China). Ultrasound parameters were set to 1.5 W/cm^2 with a 30% duty cycle, a center frequency of 1 MHz, and a duration of 60 seconds. Lungs, kidneys, and livers were collected and homogenized from mice at 0, 1, 2, 6, 12, 24, 48, and 72 hours post-treatment. Fluorescence signal intensity was measured using IVIS, and quantitatively analyzed with a PerkinElmer multifunctional microplate reader (Boston, MA, USA).

The description has been added (page 43 line 1047-1059) as: **LIPUS-mediated iMPC nose-brain delivery is a safe strategy for GBM** To further assess the safety of LIPUS-combined iMPC therapy, hematological and blood biochemical analyses were performed. The study found no significant changes in blood cell components—white blood cells, red blood cells, platelets, and hemoglobin—in GBM-bearing mice across various treatment groups, with all parameters staying within normal ranges (Supplementary Fig. 33a–j). Plasma levels of alanine aminotransferase (ALT), aspartate aminotransferase (AST), total bilirubin (TBIL), urea, and creatinine (Crea) in GBM mice across different treatment groups remained within normal physiological ranges (Supplementary Fig. 33k–p).

H&E staining of the heart, liver, spleen, lungs, and kidneys showed no significant tissue damage across all treatment groups (Supplementary Fig. 33q). These findings indicate that LIPUS-mediated nasal-to-brain delivery of iMPC represents a safe strategy for enhancing immunotherapy in GBM.

The description has been added (page 75 line 1923-1932) as: **Safety evaluation** Post-experiment assays were conducted to assess the safety effects of different treatments on C57BL/6J mice. Histological changes in the heart, liver, spleen, lungs, and kidneys

were examined using hematoxylin–eosin (HE) staining to evaluate the toxic effects on these major organs. Second, blood biochemical analysis targeted key hepatic and renal function indicators: alanine aminotransferase (ALT), aspartate aminotransferase (AST), total bilirubin (TBIL), serum creatinine (Crea), urea, and uric acid. Routine blood tests, including RBC, WBC, and PLT counts, along with leukocyte subset analysis, were conducted to assess the treatments' effects on immune function and the hematopoietic system.

12. At line 276, iMPC "possesses"

Re: Thank you for your comment. We have revised it.

13. At line 283, there is an incomplete sentence for the chambers.

Re: Thank you for your comment. We have revised it.

14. Check the figure captions/legends for Fig. 2 and bold the characters from e through k. Mention the cells utilized in Fig. 2h-k both in the figure legends and in their explanation in the manuscript. Also, at line 316, the statement of the cell activity is not clear.

Re: Thank you for your comment. We have revised it.

[Figure Redacted]

Figure 2. LIPUS-triggered NMB model opening combined with iMPC inhibits GL261 cell growth *in vitro*. a, Schematic diagram of an in vitro simulation experiment where US enhances the penetration of iMPC through the nasal mucosal barrier, increasing T-cell suppression of GL261 cells. **b**, Assessment of GL261 cell viability following LIPUS-mediated NMB model opening combined with iMPC treatment. **c**, The fluorescence intensity of Cy5 NHS-labeled aPD-L1 in the lower chamber. **d**, The early apoptosis rate, late apoptosis rate, and total apoptosis rate of GL261 cells

after 24 hours of different treatments. **e,** Quantitative analysis of aPD-L1 in the lower chamber of the Transwell using a multifunctional microplate reader after LFUS treatment at different intensities. **f-g,** *In vivo* imaging system evaluation of the amount of iMPC penetration through the nasal mucosal barrier model after 60 seconds of LFUS exposure at intensities of 0 w/cm², 0.5 w/cm², 1.5 w/cm², 3.0 w/cm², 4.0 w/cm², and 5.0 w/cm². **h,** Immunofluorescence of Claudin-1 expression (blue: DAPI; green: Alexa Fluor 488-Claudin-1) after 1 hour of different treatments. Immunofluorescence of Occludin expression (blue: DAPI; green: Alexa Fluor 488-Occludin) after 1 hour of different treatments. **i,** Comparison of the effects of ultrasound on the tight junction proteins Occludin and Claudin-1 at intensities of 0.5 w/cm², 1.5 w/cm², and 3.0 w/cm². **j,** Immunofluorescence staining of Occludin and Claudin-1 was performed at 0, 0.5, 1, 2, 3, and 4 hours after LFUS exposure at 1.5 w/cm² for 60 seconds (blue: DAPI; green: Alexa fluor 488-Occludin or Claudin-1). **k,** Immunofluorescence staining of Occludin and Claudin-1 was conducted at 0, 0.5, 1, 2, 3, and 4 hours after LFUS exposure at 3.0 w/cm² for 60 seconds (blue: DAPI; green: Alexa fluor 488-Occludin or Claudin-1. The area marked with a white arrow in **h-k** indicates the open section of the NMB model. n = 5 biologically independent mice per group for data in **b**. n = 4 biologically independent mice per group for data in **c-d** and **f**. n = 10 biologically independent mice per group for data in **e**. Data are expressed as the mean ± s.d. Comparisons were performed between different groups in **a-f**. Statistical significances were determined using one-way ANOVA with Dunnett's post hoc test. Not significant (NS) is $P \geq 0.05$, and significant P values are shown.

The description has been added (page 13 line 311-425) as: *In vitro* experiments were conducted to mimic the LIPUS open NMB model and improve iMPC permeation efficiency. A transwell-based NMB model was developed and analyzed using Dunnett's post hoc test (Fig. 2a). Compared to the untreated control group, the cell viability of GL261 cells treated with free iMPC decreased by 1.54-fold (Fig. 2b). In comparison with the free iMPC-treated group, the cell viability of GL261 cells in the US+iMPC-treated group was reduced by 7.24-fold (Fig. 2b). Fluorescence microscopy demonstrated a notable increase in aPD-L1 content in the lower chamber for the US+iMPC-treated group compared to the iMPC-treated group (Fig. 2c and Supplementary Fig. 4e).

Analysis of extracellular solutions from GL261 cells revealed enhanced fluorescence signals in the iMPC+GL261 group compared to the iMPC group, consistent with free aPD-L1-Cy5 fluorescence signals (Supplementary Fig. 4f). Confocal microscopy revealed that iMPC-released aPD-L1-Cy5 accumulated in the intercellular spaces of GL261-GFP cells (Supplementary Fig. 4g). These results indicate that iMPC release of aPD-L1 occurs in the intercellular spaces of tumor cells rather than inside tumor cells.

The impact of US+iMPC on GL261 cells was further assessed using scratch tests,

flow-through apoptosis assays, and immunofluorescence assays. Scratch test results showed that, compared with free iMPC treatment, US+iMPC treatment resulted in a 4.05-fold decrease in GL261 cell migration rate (Supplementary Fig. 5a-c). GL261 cell migration remained largely unchanged in both the free aPD-L1 and P&C (free aPD-L1 & carvedilol) groups relative to the control group, primarily due to NMB hindering drug permeation (Supplementary Fig. 5a-c).

Additional research indicated that GL261 cell viability remained unchanged in the LIPUS-only group relative to the PBS group (Supplementary Fig. 5d). Compared to the Free P&C group, GL261 cell viability decreased by 59.93% in the iMPC group and by 85.92% in the iMPC+US group (Supplementary Fig. 5d). Compared with the LIPUS group, GL261 cell viability decreased by 87.48% in the iMPC+US group (Supplementary Fig. 5d). The data suggest that LIPUS at an intensity of 1.5 w/cm^2 does not impact the viability of GL261 cells.

Caspase-3 immunofluorescence and flow cytometry analyses revealed a 2 (Fig. 29-fold increase in the apoptosis rate of GL261 cells following US+iMPC treatment compared to free iMPC treatment (Supplementary Fig. 5d-e). Likewise, caspase-3 expression and apoptosis rate in GL261 cells remained unchanged in both the free aPD-L1 and P&C groups compared to the control group (Supplementary Fig. 5d-f).

Flow cytometry analysis indicated that the early apoptosis rate of GL261 cells increased by 112.95% in the free PC group and by 192.17% in the iMPC+US group compared to the control group (Fig. 2d and Supplementary Fig. 5g-i). The late apoptosis rate of GL261 cells increased by 131.87% in the free PC group and by 279.32% in the iMPC+US group compared to the control group (Fig. 2d and Supplementary Fig. 5g-i). The total apoptosis rate of GL261 cells increased by 134.81% in the iMPC group and by 202.46% in the iMPC+US group compared to the free PC group (Fig. 2d and Supplementary Fig. 5g-i).

Flow cytometry was used to assess the impact of combining LIPUS with iMPC on preventing T cell exhaustion (Supplementary Fig. 6). The US+iMPC treatment group showed a higher T cell proportion in the lower chamber than other groups (Supplementary Fig. 7a-b). The CD3$^+$CD8$^+$ T cell proportion in the lower chamber of

the US+iMPC group was 119.5% higher than in the iMPC-treated group (Supplementary Fig. 7a-b). The US+iMPC group exhibited a significant increase in the proportions of IFN-γ^+CD8$^+$ T cells and TNF-α^+CD8$^+$ T cells in the lower chamber compared to other treatment groups (Supplementary Fig. 7c-d).

Furthermore, in the US+iMPC group, the proportion of IFN-γ^+CD8$^+$ T cells rose by 178.09%, and TNF-α^+CD8$^+$ T cells increased by 194.74% in the lower chamber compared to the iMPC-treated group (Supplementary Fig. 7c-d). In the US+iMPC group, T cells exhibited notably lower expression levels of Tim-3 and PD-1 in the lower chamber compared to the iMPC treatment group alone (Supplementary Fig. 7e-f). The US+iMPC group exhibited an 84.2% reduction in PD-1$^+$Tim-3$^+$CD8$^+$ T cells in the lower chamber compared to the iMPC-treated group (Supplementary Fig. 7g-h).

The transendothelial electrical resistance (TEER) values in the NMB model stabilized by the fifth day, as shown in Supplementary Fig. 8a. An *in vivo* imaging system (*IVIS*) assessment demonstrated that the aPD-L1 content in the lower chamber progressively rose with increased US intensity (Fig. 2e-g). This conclusion was further validated by quantitative measurements using a microplate reader (Fig. 2e-g). TEER measurement results indicate that the TEER value of the NMB model gradually decreases with increasing US intensity (Supplementary Fig. 8b). The findings suggest a significant correlation between the extent of NMB disruption and ultrasonic energy, highlighting the need to identify a safe ultrasound intensity range to ensure NMB damage reversibility.

We further investigate changes in tight junctions in the NMB model via immunofluorescence. The study found that US+iMPC treatment in the NMB model led to the disruption of tight junction proteins claudin-1 and occludin (Fig. 2h). Compared to other groups without US treatment, the TEER values for the NMB model in the US+iMPC group were significantly reduced (Supplementary Fig. 8c). The Free aPD-L1, P&C, MPC, and iMPC-treated groups showed no significant changes in the tight junction proteins claudin-1 and occludin, indicating that LIPUS, when not applied, does not significantly affect NMB (Fig. 2h). When other parameters (time 60s, duty cycle 50%, frequency 1MHz) remained constant, the disruption of claudin-1 and occludin

became more pronounced with increasing US intensity (Fig. 2i). This suggests that the degree of NMB disruption gradually increases as the ultrasonic energy is enhanced (Fig. 2i).

To further assess the reversibility of NMB disruption by LIPUS, immunofluorescence experiments and TEER test were performed. The results demonstrated that when the LIPUS intensity was 1.5 w/cm^2, the NMB model could recover within 4 h (Fig. 2j and Supplementary Fig. 8d). In contrast, at a higher LIPUS intensity of 3.0 w/cm^2, the disruption of tight junction proteins in the NMB model was irreversible (Fig. 2k and Supplementary Fig. 8e). These dates demonstrates that LIPUS at 1.5 w/cm^2 intensity can safely and reversibly disrupt NMB tight junction proteins, allowing iMPC to penetrate the NMB, reduce T-cell exhaustion, and improve the inhibition of GL261 cells.

15. At line 358, do the authors mean 2e-g?

Re: Thank you for your comment. We have revised it.

The description has been added (page 16 line 396-399) as: An *in vivo* imaging system (*IVIS*) assessment demonstrated that the aPD-L1 content in the lower chamber progressively rose with increased US intensity (Fig. 2e-g). This conclusion was further validated by quantitative measurements using a microplate reader (Fig. 2e-g).

16. At line 383, 1 "hour"

Re: Thank you for your comment. We have revised it.

The description has been added (page 21 line 495) as: **c-d,** The distribution of aPD-L1-Cy5 in the trigeminal nerve after 1 hour of treatment.

17. In the efficiency section, the mix of delivery efficiency and radiant efficiency is confusing. Additionally, what's the definition of retention?

Re: Thank you for your comment. We have revised it.

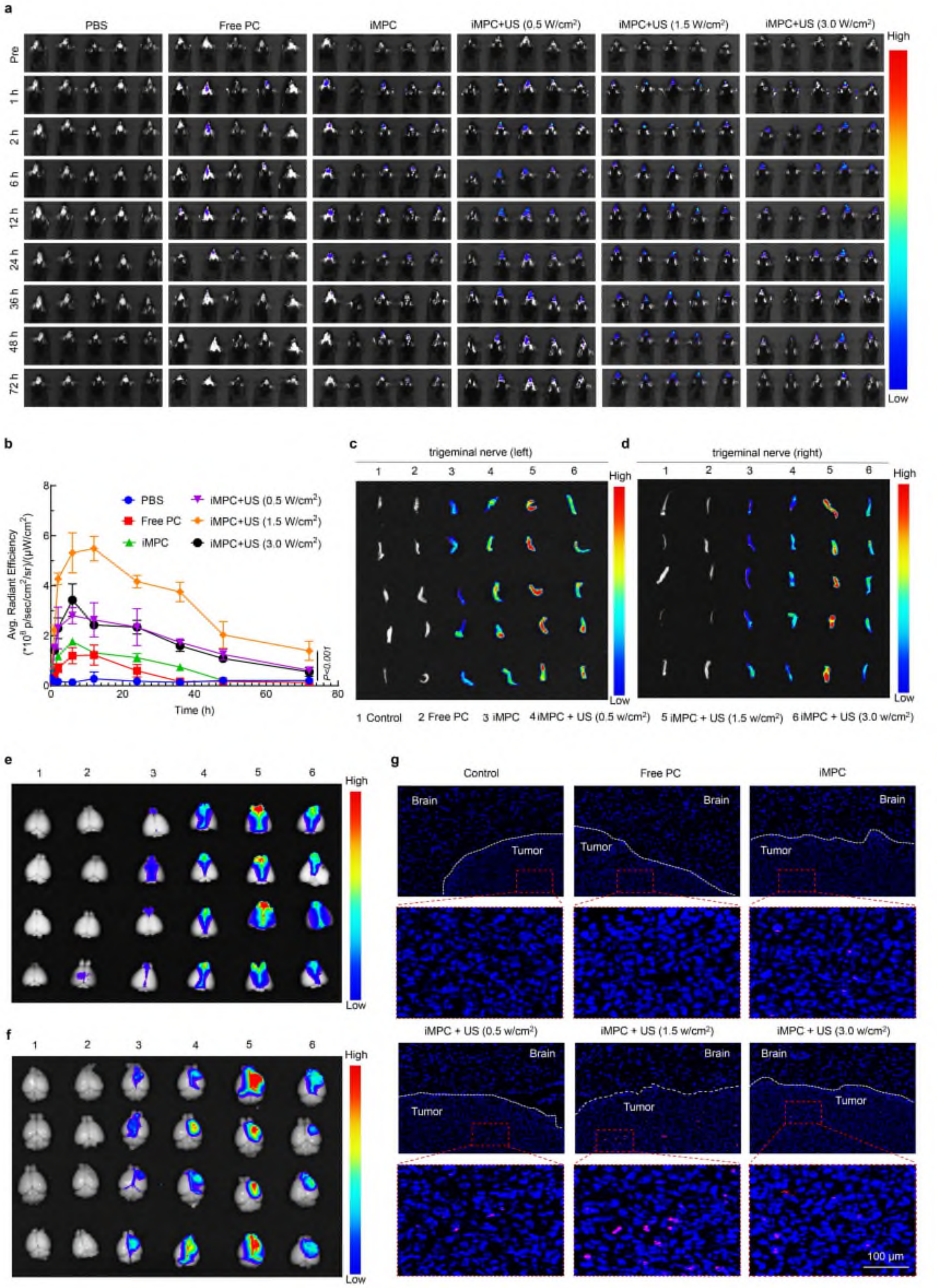

Figure 3. LIPUS improves iMPC nose-to-brain delivery efficiency *in vivo*. a-b, The distribution of Cy5 NHS-labeled aPD-L1 after various treatments. ImageJ software analyzes fluorescence signals. **c-d,** The distribution of aPD-L1-Cy5 in the trigeminal nerve after 1 hour of treatment. **e,** The distribution of aPD-L1-Cy5 in the olfactory bulb after 2 hours of treatment. **f-g,** The distribution of aPD-L1-Cy5 in the brain tumor area after 6 hours of treatment using both the small animal *in vivo* imaging system and frozen sectioning. n = 4 biologically independent mice per group for data in **b**. Data are expressed as the mean ± s.d.

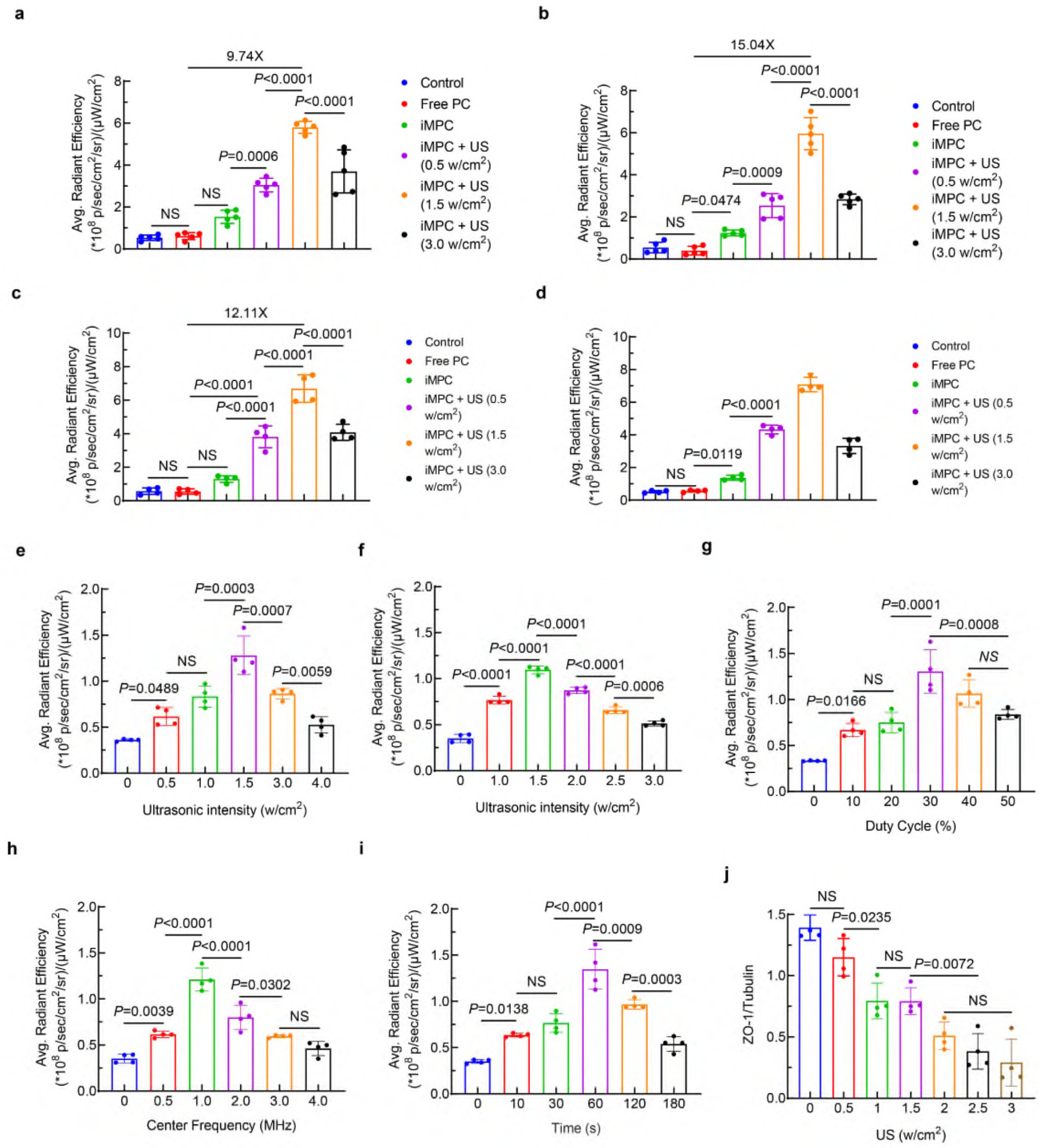

Supplementary Figure 13. *In vivo* imaging system (*IVIS*) for fluorescence signal quantification of aPD-L1-Cy5 in neural bypass, olfactory bulb, and tumor tissue. a-b, Quantitative analysis of aPD-L1-Cy5 fluorescence signal in the trigeminal neural bypass using ImageJ software 1 hour after intranasal administration. **c**, Quantitative analysis of aPD-L1-Cy5 fluorescence signal in the trigeminal neural bypass using ImageJ software 2 hours after intranasal administration. **d**, Quantitative analysis of aPD-L1-Cy5 fluorescence signal in the tumor area using ImageJ software 6 hours after intranasal administration. **e-f**, Fluorescence signals of iMPC-Cy5 in brain tumors after LIPUS treatment at different ultrasound intensities. **g,** Fluorescence signals of iMPC-Cy5 in brain tumors after LFUS treatment with different duty cycles. **h,** Fluorescence signals of iMPC-Cy5 in brain tumors after LFUS treatment at different center frequencies. **i,** Fluorescence signals of iMPC-Cy5 in brain tumors after LFUS treatment for different durations. **j**, ZO-1 reduces with increasing ultrasonic intensity. n = 5 biologically independent mice per group for data in **a-d**. n = 4 biologically independent mice per group for data in **e-j**. Data are expressed as the mean ± s.d. Statistical significances were determined using one-way ANOVA with Dunnett's post hoc test. Comparisons were performed between different groups in **a-j**. Not significant (NS) is $P \geq 0.05$, and significant P values are shown.

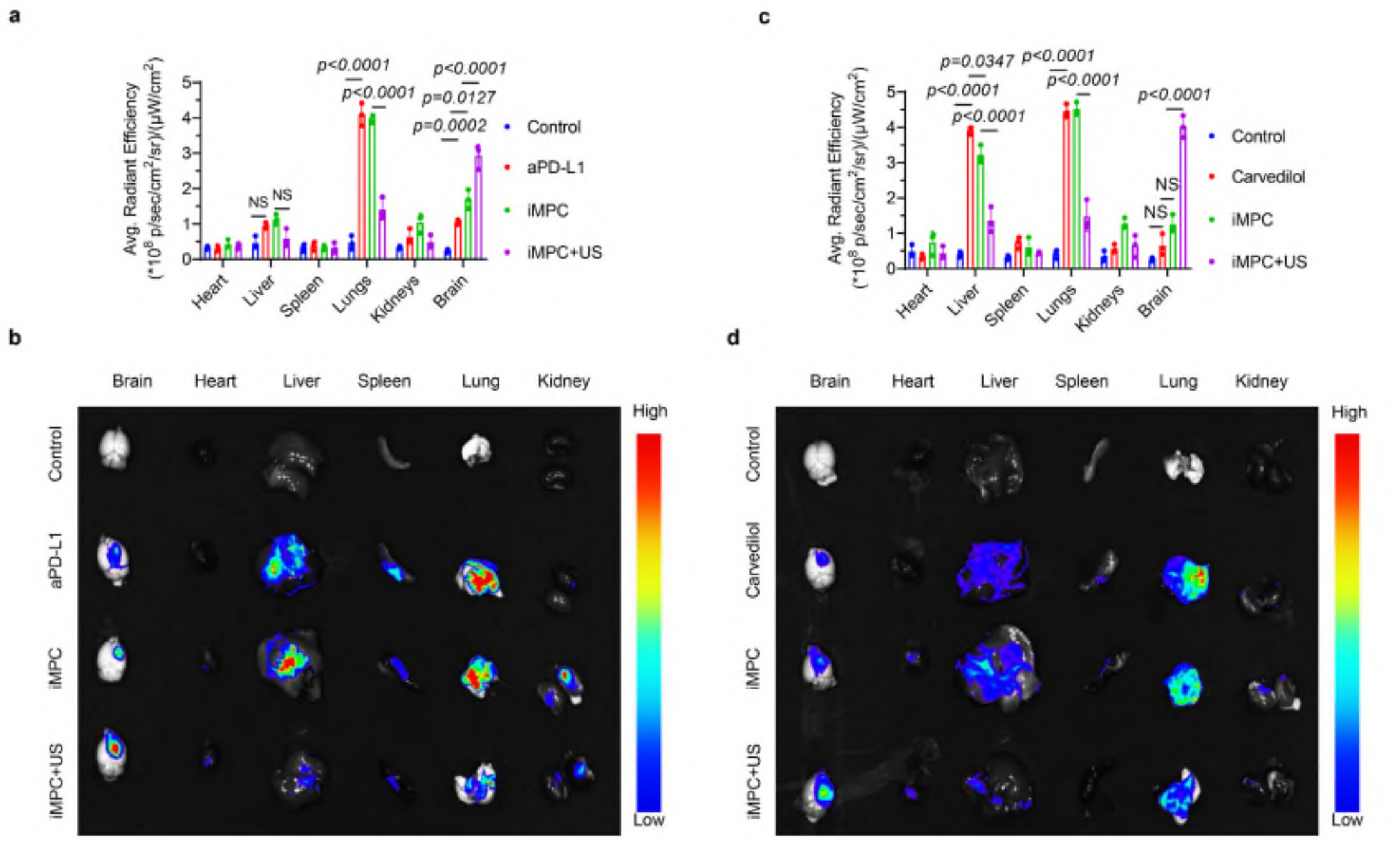

Supplementary Figure 15. Organ distribution of carvedilol, aPD-L1, and iMPC nano-formulations. a-b, Distribution of aPD-L1 and iMPC nano-formulations in heart, liver, spleen, lung, and kidney. **c-d,** Distribution of carvedilol and iMPC nano-formulations in heart, liver, spleen, lung, and kidney. n = 3 biologically independent mice per group for data in **a** and **c**. Data are expressed as the mean ± s.d. Statistical significances were determined using one-way ANOVA with Dunnett's post hoc test. Comparisons were performed between different groups in **a** and **c**. Not significant (NS) is $P \geq 0.05$, and significant P values are shown.

The description has been added (page 21 line 501-558) as: **LIPUS improves iMPC nose-to-brain delivery efficiency *in vivo*** Additional *in vivo* experiments assessed the efficiency of LIPUS-enhanced iMPC penetration across the NMB. *IVIS* imaging analyses demonstrated that the combination of LIPUS and iMPCs significantly improved iMPC penetration across the NMB and extended their retention time in the GBM microenvironment compared to the iMPC treatment alone (Fig. 3a–b). In addition, iMPC had the highest nose-to-brain delivery efficiency when the ultrasound intensity was 1.5 w/cm², compared to ultrasound intensities of 0.5 or 3.0 w/cm² (Fig. 3a–b).

Research indicates that the trigeminal bypass is the primary route for nose-to-brain drug delivery, accessing the olfactory bulb and brain ventricles[2,3,6,14]. *IVIS* observations at 1 hour post-treatment indicated that the LIPUS combined with iMPC treatment group showed enhanced iMPC distribution on the trigeminal nerve surface compared to the iMPC treatment group (Fig. 3c–d and Supplementary Fig. 13a–b).

The LIPUS treatment at an intensity of 1.5 w/cm² resulted in the greatest iMPC

accumulation on the isolated trigeminal nerve surface compared to intensities of 0.5 or 3.0 w/cm² (Fig. 3c–d and Supplementary Fig. 13a–b). The fluorescent signals (aPD-L1-Cy5) distributed on the surface of the right and left branches of the trigeminal nerve increased by 9.74 and 15.04-fold, respectively, in the US+iMPC group (1.5 w/cm²) compared with the free PC (aPD-L1 and carvedilol) group (Supplementary Fig. 13a–b).

Moreover, at 2 h post-treatment, the LIPUS exposure significant enhanced iMPC distribution on the surface of the isolated olfactory bulb compared to iMPC alone (Fig. 3e and Supplementary Fig. 13c). Notably, the LIPUS treatment at 1.5 w/cm² resulted in the highest iMPC content on the olfactory bulb surface (Fig. 3e and Supplementary Fig. 13c). The fluorescence signal (aPD-L1-Cy5) on the surface of the olfactory bulb increased 12.11-fold in the US+iMPC group (1.5 w/cm²) compared to the free PC-treated group (Supplementary Fig. 13c). By 6 h post-treatment, increased iMPC aggregation in the GBM region was observed in US+iMPC group compared with the iMPC treatment group (Fig. 3f and Supplementary Fig. 13d). Moreover, the LIPUS treatment at 1.5 w/cm² demonstrated the highest iMPC content in the GBM region (Fig. 3f and Supplementary Fig. 13d). The fluorescence signal (aPD-L1-Cy5) on the surface of the olfactory bulb increased 12.53-fold in the US+iMPC group (1.5 w/cm²) compared to the free PC-treated group (Supplementary Fig. 13d).

Frozen section analysis of brain tissue indicated that the LIPUS combined with iMPC treatment group showed increased iMPC infiltration in tumor tissue compared to the iMPC treatment group (Fig. 3 g). The LIPUS treatment at 1.5 w/cm² led to the greatest iMPC infiltration in tumor tissue (Fig. 3g). These results indicate that LIPUS can significantly improve the efficiency of iMPC penetration across the NMB into the brain, and iMPC possesses the ability to target and accumulate in GBM.

The study further assessed how different acoustic parameters, such as ultrasound intensity, center frequency, duty cycle, and duration, influence the efficiency of LIPUS-mediated nose-to-brain delivery of iMPC. The findings indicated that the iMPC-Cy5 concentration in tumor tissues of GBM mice subjected to LIPUS at 1.5 w/cm² was 3.55 times greater than in those treated with 0 w/cm² (Supplementary Fig. 13e). Modulating

various LIPUS intensities revealed that 1.5 w/cm² maximized iMPC nose-to-brain delivery efficiency (Supplementary Fig. 13f). The iMPC-Cy5 level in tumor tissues of GBM mice subjected to LIPUS with a 30% duty cycle was 1.95 times greater than in those treated with a 10% duty cycle (Supplementary Fig. 13g). Treatment with LIPUS at a center frequency of 1.0 MHz led to a 1.97-fold increase in iMPC-Cy5 in tumor tissues compared to a frequency of 0.5 MHz (Supplementary Fig. 13h).

Further modulation of the LIPUS exposure duration revealed that the highest iMPC-Cy5 content in tumor tissues was achieved with an LIPUS duration of 60 seconds (Supplementary Fig. 13i). A decline in nose-to-brain delivery efficiency of iMPC was observed when the LIPUS duration was extended to 120 and 180 seconds, which may be attributed to thermal damage of the nasal mucosa induced by prolonged LIPUS exposure[7,11]. The study identifies the optimal acoustic parameters for LIPUS-mediated iMPC nose-to-brain delivery as a center frequency of 1.0 MHz, ultrasound intensity of 1.5 w/cm², a duty cycle of 30%, and a duration of 60 seconds.

The description has been added (page 62 line 1595-1660) as: **Nose-to-brain delivery efficiency** Mice bearing orthotopic GBM models were intranasally administered with six different treatments: PBS, Free PC (aPD-L1 & carvedilol), iMPC, iMPC+US (0.5 w/cm²), iMPC+US (1.5 w/cm²), and iMPC+US (3.0 w/cm²). In the iMPC+US group, nasal mucosa was first subjected to US treatment, followed by intranasal administration of the iMPC suspension. LIPUS was administered to the olfactory epithelium in the mouse nasal cavity. aPD-L1 was administered at 30 µg per 20 g of body weight, while carvedilol was given at 4 µg per 20 g of body weight. Other US parameters were as follows: duty cycle 30%, center frequency 1 MHz, and duration 60 s10,14,16. aPD-L1 was labeled with Cy5. The LIPUS device was provided by the National Key Laboratory of Intelligent Imaging and Interventional Medicine of China.

Fluorescence intensity in the mouse brain was monitored using *IVIS* (ABL X5, Tanon, China) at pre-treatment and at 1, 2, 6, 12, 24, 36, 48, and 72 hours post-treatment across six different treatments. At 2 hours post-treatment, the trigeminal nerves were dissected and analyzed under *IVIS* to evaluate fluorescence intensity. Brain tissues were collected at 6 and 12 hours post-treatment, and fluorescence signal intensity was assessed using

IVIS followed by statistical analysis. Brain tissues were collected 12 hours after treatment, and frozen sections of the tumor area were prepared. Nuclei were stained with DAPI, and aPD-L1-Cy5 distribution was analyzed using a Leica TCS SP confocal fluorescence microscope.

Additionally, US parameters were methodically adjusted to evaluate the efficiency of LIPUS-facilitated nose-to-brain delivery of iMPC. The US parameters were varied as follows: intensity (0 to 4.0 w/cm^2), duty cycle (0% to 50%), center frequency (0 to 4.0 MHz), and application duration (0 to 180 seconds). aPD-L1 was labeled with Cy5. At 12 h post LIPUS-mediated nasal administration of iMPC, brain tumor tissues were harvested, homogenized, and subjected to fluorescence intensity measurement and statistical analysis using *IVIS*.

Comparison of the efficiency of drug delivery to the tumor region between LIPUS-mediated intranasal-to-brain delivery of free PC (aPD-L1 and carvedilol) and iMPC. C57BL/6 mice were allocated into four treatment groups: control, Free PC (aPD-L1 and carvedilol), PC+US (ultrasound-mediated intranasal delivery of aPD-L1 and carvedilol), and iMPC+US (ultrasound-mediated intranasal delivery of iMPC). The administered doses were 30 μg/20 g for aPD-L1 and 4 μg/20 g for carvedilol. The US parameters were configured with an intensity of 1.5 w/cm^2, a duty cycle of 30%, a center frequency of 1 MHz, and a duration of 60 seconds. Additionally, aPD-L1 was labeled with Cy5. Fluorescence signal intensity in the brain was measured using an *IVIS* imaging system at pre-administration and at 2, 6, 12, 24, 48, and 72 hours post-administration. Twelve hours after administration, brain tissues were dissected, and frozen tumor region sections were prepared. Nuclei were stained with DAPI, and aPD-L1-Cy5 distribution in the brain tumor was examined using a Leica TCS SP confocal laser scanning microscope. Brain tumor tissues were homogenized, and aPD-L1-Cy5 concentrations were quantitatively measured using a multifunctional microplate reader (PerkinElmer, Boston, MA, USA).

iMPC nanovesicles with particle sizes of 50, 100, 150, 200, and 300 nm were constructed, and the average particle size distribution of iMPC was measured using a Malvern particle size analyzer. aPD-L1 was labeled with Cy5. In a mouse orthotopic

GBM model, nasal administration was conducted, and brain fluorescence signal intensity was measured using *IVIS* at pre-administration and 2, 6, 12, 24, 48, and 72 hours post-administration. Six hours post-administration, brain tissues were isolated, and fluorescence signal intensity in the brain tumor region was assessed using *IVIS*. Fluorescence signal analysis was conducted using ImageJ software (ImageJ Software Inc., USA). Brain tumor tissues were homogenized for quantitative analysis of aPD-L1-Cy5 using a multifunctional microplate reader (PerkinElmer, Boston, MA, USA).

Further evaluation of tumor targeting by iRGD-modified iMPCs *in vivo*. C57 mice (6–8 weeks old, 20–22 g) were randomly assigned by sex for intranasal administration in the experiment. The experiments comprised four groups: PBS control, Free PC, MPC+US, and iMPC+US. aPD-L1 was labeled with Cy5-NHS (Starleaf Biological Technology Co, Nanjing, China). The dosage of aPD-L1 was administered at 30 μg for every 20 g of body weight. LIPUS was directed at the olfactory epithelium within the mouse nasal cavity. Ultrasound parameters included a 1.5 w/cm² intensity, 30% duty cycle, 1 MHz center frequency, and a 60-second duration. The efficiency of nasal-to-brain drug delivery was evaluated using *IVIS* imaging at intervals of 0, 1, 2, 6, 12, 24, 48, and 72 hours post-treatment for all groups. Fluorescence signals were statistically analyzed using ImageJ software (ImageJ Software Inc., USA). Six hours after treatment, frozen sections were prepared, stained with DAPI, and imaged using a Zeiss LSM 510 laser scanning fluorescence confocal microscope to observe aPD-L1-Cy5 distribution.

18. At line 406, is "sound intensities" a correct use?

Re: Thank you for your comment. We have reviewed the entire manuscript, and the "sound intensities" has been revised to "ultrasound intensities."

19. The authors do not need to define abbreviations repeatedly. For instance, at line 477, it is not necessary to spell out the full names of LFUS and NMB, as these have already been explained in earlier sections. Please review the entire manuscript for consistency.

Re: Thank you for your comment. We have reviewed the entire manuscript, and revised these issues.

20. At line 417, add a space after a period. **20.**

Re: Thank you for your comment. We have revised it.

21. At line 699, "ELISA"

Re: Thank you for your comment. We have revised it.

22. At line 770, "Statistical" and between "the" two

Re: Thank you for your comment. We have revised it.

23. At line 892, "anti-tumor"

Re: Thank you for your comment. We have revised it.

The description has been added (page 47 line 1120-1122) as: The iMPC co-delivers β-blockers (carvedilol) and aPD-L1 to mitigate T-cell exhaustion and improve T-cell antitumor activity

24. At line 923, while "minimizing"

Thank you for your comment. We have revised it.

The description has been added (page 48 line 1151-1154) as: Integrating artificial intelligence (AI) algorithms to optimize ultrasound parameters based on individual patient data could further personalize the treatment, maximizing therapeutic benefits while minimizing side effects.

25. At line 1106, "," where

Thank you for your comment. We have revised it.

The description has been added (page 57 line 1415-1418) as: Cell viability (%) was determined using the formula: (OD experimental – OD blank) / (OD control – OD blank) * 100%. The control group comprised untreated normal cells, while the blank group included only basal medium with 10% CCK-8 reagent.

Reviewer #4 (Remarks to the Author):

I co-reviewed this manuscript with one of the reviewers who provided the listed reports. This is part of the Nature Communications initiative to facilitate training in peer review and to provide appropriate recognition for Early Career Researchers who co-review manuscripts.

REVIEWERS' COMMENTS

Reviewer #1 (Remarks to the Author):

The authors have properly addressed all concerns with revisions. It is suggested to be accepted for publication.

Reviewer #2 (Remarks to the Author):

The manuscript has been sufficiently revised and is now suitable for publication

Reviewer #3 (Remarks to the Author):

The authors have addressed most of the comments; however, there are still some typographical errors that need revision:

1. The title contains a grammatical error.
Re: Thank you for bringing this to our attention. The title has been amended to: 'Low-intensity pulsed ultrasound-mediated nose-to-brain co-delivery of β-blockers and aPD-L1 enhances glioblastoma immunotherapy'.

2. Please ensure that the terms "claudin-1" and "occludin" are consistently written either in lowercase or capitalized throughout the entire article.
Re: Thank you for your comment. Throughout the entire article, the terms have been revised to 'Claudin-1' and 'Occludin'.

3. In Scheme 1, is it intended to be "LFPUS"?
Re: Thank you for your comment. In Scheme 1, 'US' has been amended to 'LIPUS'.

4. In line 115, "two-hour" should be used in the singular form.
Re: Thank you for pointing that out. On line 115, 'two-hours' has been revised to 'two-hour'.

5. There is a grammatical error in line 446.
Re: Thank you for your comment. It has been revised to read: "These findings indicate that US could interfere with NMB by triggering the cGMP/PKG signaling pathway."

6. In lines 623 and 630, "ultrasound" should be written with a lowercase "u."
Re: Thank you for your comment. 'Ultrasound' has been changed to 'ultrasound'.

7. In line 739, the authors mention "NMB" twice.
Re: Thank you for your comment. It has been revised to read 'LIPUS induces reversible opening of NMB'.

Reviewer #4 (Remarks to the Author):

I co-reviewed this manuscript with one of the reviewers who provided the listed reports. This is part of the Nature Communications initiative to facilitate training in peer review and to provide appropriate recognition for Early Career Researchers who co-review manuscripts.
